# Immunogenicity, durability, and safety of an mRNA and three platform-based COVID-19 vaccines as a third dose following two doses of CoronaVac in China: A randomised, double-blinded, placebo-controlled, phase 2 trial

**DOI:** 10.1016/j.eclinm.2022.101680

**Published:** 2022-09-28

**Authors:** Yuemiao Zhang, Xupu Ma, Guanghong Yan, Ying Wu, Yanli Chen, Zumi Zhou, Na Wan, Wei Su, Feng-Wei Liu, Mu-Xian Dai, Mei Yang, Chunmei Li, Xuanjing Yu, Liang Zhang, Zhongfang Wang, Tai-Cheng Zhou, Dingyun You, Jia Wei, Zijie Zhang, Precise-CoVaccine study group

**Affiliations:** a State Key Laboratory for Conservation and Utilization of Bio-resource and School of Life Sciences, Yunnan University, Kunming, Yunnan 650091, China; b Renal Division, Department of Medicine, Peking University First Hospital, Renal Pathology Center, Institute of Nephrology, Peking University, Key Laboratory of Renal Disease, Ministry of Health of China, Key Laboratory of CKD Prevention and Treatment, Ministry of Education of China; Research Units of Diagnosis and Treatment of Immune-Mediated Kidney Diseases, Chinese Academy of Medical Sciences, Beijing 100034, People's Republic of China; c State Key Laboratory of Genetic Resources and Evolution, and Yunnan Laboratory of Molecular Biology of Domestic Animals, Kunming Institute of Zoology, Chinese Academy of Sciences, Kunming 650223 and College of Life Science, University of Chinese Academy of Sciences, Beijing 100049, China; d School of Public Health, Kunming Medical University, Kunming, Yunnan, China; e Guangdong Provincial Key Laboratory of Tropical Disease Research, Department of Biostatistics, School of Public Health, Southern Medical University, Guangdong, China; f Institute of Medical Biology, Chinese Academy of Medical Sciences & Peking Union Medical College, Kunming, Yunnan, People's Republic of China; g Yunnan Genvoo Biotech Ltd., Kunming, Yunnan, People's Republic of China; h Central Lab, Liver Disease Research Center and Department of Infectious Disease, The Affiliated Hospital of Yunnan University, Kunming, Yunnan 650091, China; i State Key Laboratory of Respiratory Disease & National Clinical Research Center for Respiratory Disease, Guangzhou Institute of Respiratory Health, the First Affiliated Hospital of Guangzhou Medical University, Guangzhou Medical University, Guangzhou, China; j Yunnan Key Laboratory of Stem Cell and Regenerative Medicine, Institute of Biomedical Engineering, Kunming Medical University, Kunming 650500, China; k Yunnan Key Laboratory of Stomatology, Kunming Medical University, Kunming, Yunnan 650500, PR China

**Keywords:** COVID-19 mRNA vaccine, Immunogenicity, Safety, Durability

## Abstract

**Background:**

More effective vaccine candidates against variants of concern as a booster dose are needed in people primed with two-dose inactivated COVID-19 vaccines.

**Methods:**

This randomised, double-blinded, investigator-initiated phase 2 trial aims to evaluate immunogenicity, durability, and safety of an mRNA vaccine candidate (RQ3013) and three other platform vaccines (an adenovirus-vectored vaccine candidate [ChAdTS-S], a recombinant protein vaccine candidate [ZR202-CoV], and an inactivated vaccine [CoronaVac]) as a booster. 250 eligible volunteers, who had received a prime two-dose CoronaVac (3 to 5 weeks apart) vaccination 100-270 days before, were randomly assigned in a 1:1:1:1:1 ratio to receive a third dose of RQ3013 (30 μg mRNA per 0.15 mL), ChAdTS-S (5×10^10^ viral particles per 0.5 mL), ZR202-CoV (25 μg prefusion-stabilized Spike ectodomain trimer per 0.5 mL), CoronaVac (3 μg inactivated CN02 strain of SARS-CoV-2 per 0.5 mL) or placebo (0.5 mL of 0.9% sodium chloride solution) via intramuscular injection into the upper arm at a single clinical site in Kunming, China. Participants, investigators, and immunogenicity laboratory were masked to group assignment. The primary immunogenicity outcomes were geometric mean titres (GMTs) of neutralising antibodies against live SARS-CoV-2 (wild-type, delta and omicron) virus at day 0 (before vaccination), day 7, day 14 and day 28 after vaccination, as analysed in a modified intention-to-treat (mITT) population (all participants who completed their booster doses and had at least one post-dose immunogenicity data). Secondary outcomes include T cell responses against the wild-type and omicron SARS-CoV-2 Spike protein. The primary safety outcome was incidence of adverse events within 14 days after the booster vaccination. This trial is registered with ChiCTR.org.cn, ChiCTR2200057758.

**Findings:**

Between January 1, 2022, and February 28, 2022, 235 eligible participants were enrolled and vaccinated, and the primary analysis included 234 participants. At baseline, neutralising antibodies against wild-type virus, the delta, or omicron variants were low or undetectable in all groups. After the booster vaccination, GMTs of neutralising antibodies ranged from 75.4 (95% confidence interval [CI] 61.4-92.5) in CoronaVac to 950.1 (95% CI 785.4-1149.3) in RQ3013 against live wild-type SARS-CoV-2, and from 8.1 (95% CI: 6.1-10.7) in CoronaVac to 247.0 (95% CI 194.1-314.3) in RQ3013 against the omicron variant at day 14. Immunogenicities of all heterologous regimens were superior to that of homologous regimen in neutralisation against all tested SARS-CoV-2 strains, with RQ3013 showing the highest geometric mean ratios (GMRs) of 12.6, 14.7, and 31.3 against the wild-type, the delta variant and the omicron variant compared to CoronaVac at day 14 post-vaccination, respectively. Durability analysis at day 90 showed that >90% of participants in RQ3013 and ZR202-CoV were seropositive for the omicron variant while ZR202-CoV with adjuvants containing CpG showed a slightly better durability than RQ3013. T cell responses specific to the omicron variant were similar to that of the wild-type, with RQ3013 showing the highest boosting effect. Any solicited injection site or systemic adverse events reported within 14 days after vaccination were most commonly observed in RQ3013 (47/47, 100%), followed by ZR202-CoV (46/47, 97.9%) and ChAdTS-S (43/48, 89.6%), and then CoronaVac (37/46, 80.4%) and placebo (21/47, 44.7%). More than 90% of the adverse events were grade 1 (mild) or 2 (moderate) with a typical resolution time of 3 days. No grade 4 adverse events or serious adverse events were reported by study vaccines.

**Interpretation:**

Although all study vaccines boosted neutralising antibodies with no safety concerns, RQ3013 showed much stronger cross-neutralisation and cellular responses, adding more effective vaccine candidates against the omicron variant.

**Funding:**

Yunnan Provincial Science and Technology Department China (202102AA100051 and 202003AC100010), the Double First-class University funding to Yunnan University, National Natural Science Foundation of China (81960116, 82060368 and 82170711), Yunnan Natural Science Foundation (202001AT070085), High-level Health Technical Personnel Project of Yunnan Province (H-2018102) and Spring City Plan: The High-level Talent Promotion and Training Project of Kunming.

**Research in context**

**Evidence before this study**

We searched PubMed for studies published before the date of writing April 15th 2022, using terms “(COVID-19 OR SARS-CoV-2) AND (vaccine) AND (booster OR third dose)” with no language restrictions. Six randomised controlled trials (RCTs) of homologous or heterologous booster vaccinations in participants primed with adenovirus-vector vaccine (AZD1222), mRNA vaccine (BNT162b2) or inactivated vaccine (CoronaVac) were identified. Besides, a case-control study assessed the booster effect of a recombinant protein vaccine (ZF2001) among inactivated-vaccine-primed population. All prime-boost vaccinations elicited humoral and cellular immune responses, but with substantial differences in strength. Among all combinations of vaccine schedules, boosting with mRNA vaccines (BNT162b2 or mRNA1273) appeared to induce higher neutralising antibody titres despite of higher but well-tolerated reactogenicity than other vaccines. However, there has been no systematic clinical study to evaluate heterologous boosting strategies comparing multi-platform vaccines accessible in China, particularly vaccine of novel immunogen design, for the largest inactivated-vaccine-primed population. More importantly, neutralising antibodies against the currently circulating omicron variant were not or only tested in a small subset of participants in previous heterologous booster dose RCTs.

**Added value of this study**

As far as we know, this is the first RCT designed to evaluate the boosting effects of an mRNA COVID-19 vaccine encoding a mutated SARS-CoV-2 Spike protein in head-to-head comparison with three other vaccines representing major COVID-19 vaccine platforms, using comprehensive immunogenicity measures (neutralising antibodies to various live SARS-CoV-2 strains, the clinically accessible neutralising antibody measured by competitive inhibition method as well as T cell responses) at multiple time points. This study revealed varying levels of neutralising antibodies, broadness against variants of concern, T cell responses, durability and reactogenicity profiles of the booster dose by four vaccines in China, with the mRNA vaccine RQ3013 demonstrating the highest neutralising antibodies and T cell responses. Although RQ3013 demonstrated superior immunogenicity than other three COVID-19 vaccines after vaccination, ZR202-CoV with alum/CpG as adjuvants showed a slightly better durability 3 months after the booster dose.

**Implications of all the available evidence**

In this study, we evaluated four COVID-19 vaccines as a third dose. Notably, our clinical evidence demonstrated the value of mRNA vaccine encoding mutated Spike protein design to achieve broader spectrum of neutralisation against variants of concern, adding more omicron-variant-immunogenic vaccines to the booster vaccination campaign toolbox in the world's largest population primed with two-dose inactivated COVID-19 vaccine.

Alt-text: Unlabelled box

## Introduction

Vaccination is one of the most cost-effective strategies to cope with the coronavirus disease 2019 (COVID-19) pandemic. As of July 2022, China has accomplished primary vaccination in over 89% of population, mostly with inactivated virus vaccine from either BBIBP-CorV (China National Pharmaceutical Group Co., Ltd.) or CoronaVac (Sinovac Biotech Ltd.).^1^, ^2^, ^3^ However, waning immunity over time,^4^^,^^5^ enhanced transmissibility and immune escape due to evolving SARS-CoV-2 variants^6^, ^7^, ^8^ resulted in a progressive decline in the vaccine-elicited immunity against SARS-CoV-2. To combat these challenges, booster (third) doses of SARS-CoV-2 vaccine have been administered, beginning with a homologous regimen.^5^, ^6^, ^7^, ^8^, ^9^ Evidence from randomised controlled trials (RCTs) and cohort studies of heterologous booster regimens among population primed with inactivated-vaccine is also accumulating.^10^, ^11^, ^12^, ^13^, ^14^ However, more effective vaccine candidates against variants of concern as a booster dose are needed in people primed with two-dose inactivated COVID-19 vaccines.

With the increasing supply of approved vaccines and maturation of newly developed vaccines, more vaccine candidates became available for the booster vaccination in China. It is necessary to evaluate vaccines of different platforms for strategic optimisation of booster dose regimen given marked differences between regimens with a variety of vaccine platforms.^5^^,^^10^^,^^11^^,^^15^, ^16^, ^17^ Thus we initiated an RCT to compare immune responses between different booster regimens representing four vaccine platforms developed in China as a booster dose (Table 1). Specifically, ChAdTS-S is a recombinant chimpanzee-adenovirus-vectored vaccine encoding the full-length Spike protein of SARS-CoV-2 developed jointly by Tsinghua University and Walvax Biotech Ltd.^18^^,^^19^ RQ3013 is a lipid-nanoparticle-embedded pseudouridine-modified-mRNA-based vaccine encoding a Spike protein containing mutations from both alpha (B.1.1.7) and beta (B.1.351) variants, which is jointly developed by Fudan University, RNAcure Biotech Ltd. and Walvax Biotech Ltd.^20^ ZR202-CoV is a recombinant protein subunit vaccine encoding the ectodomain (Spike without transmembrane domain) of prefusion-stabilized Spike protein with a T4 fibritin trimerization motif developed by Zerun Biotech Ltd. and Walvax Biotech Ltd.^21^ This study reports the magnitude, broadness, durability and kinetics of comprehensive immunogenicity measures for vaccine evaluation, including the neutralising antibodies against multiple variant of concerns using the gold standard live virus test and T-cell responses to the wild-type and omicron Spike peptide pool by a three-analytes FluoroSpot assay.

**Table 1:** Platforms of the four investigational COVID-19 vaccines in this trial.

Vaccine name (platform)	Mechanism of action	Administration	Manufacture
ChAdTS-S (Adenovirus-vectored)	Replication-deficient chimpanzee adenovirus-vectored vaccine, expressing Spike protein of wild-type SARS-CoV-2	5×10^10^ viral particles per 0.5 mL via intramuscular injection	Walvax Biotech Co., Ltd.
RQ3013 (mRNA)	LNP-encapsulated mRNA vaccine encoding a mutated Spike protein harbouring all the mutations derived from the alpha variant with additional K417N, E484K, and A701V mutations found in the beta variant	30 μg mRNA per 0.15 mL via intramuscular injection	Walvax Biotech., Ltd.
ZR202-CoV (Recombinant protein)	Nanoparticle vaccine containing purified prefusion-stabilized Spike ectodomain trimer of wild-type SARS-CoV-2	25 μg Spike ectodomain trimer 500 μg aluminium hydroxide-based adjuvant and 500 μg CpG ODN in 0.5mL via intramuscular injection	Shanghai Zerun Biotech., Ltd.
CoronaVac (Inactivated)	Whole, inactivated CN02 strain (wild-type) of SARS-CoV-2	600 SARS-CoV-2 antigen units per 0.5 mL of aluminium hydroxide adjuvant via intramuscular injection	Sinovac Biotech, Ltd

Note: COVID-19=coronavirus disease 2019, LNP=lipid nanoparticle, SARS-CoV-2=severe acute respiratory syndrome coronavirus 2.

## Methods

### Study design

This is a double-blinded, randomised, placebo-controlled, investigator-initiated phase 2 trial, which was conducted at a single site at the Affiliated Hospital of Yunnan University in Yunnan, China to evaluate the immunogenicity and safety of booster dose vaccination schedules against SARS-CoV-2. This trial was reviewed and approved by the Committee on Human Subject Research and Ethics of The Affiliated Hospital of Yunnan University (ID: 2022026) and is registered with ChiCTR.org.cn (ChiCTR2200057758). The study protocol is provided in Supplementary Appendix 2. The results were reported in compliance with the CONSORT reporting guidelines.

### Participants

Participants who had received a prime two-dose CoronaVac (3 to 5 weeks apart) vaccination 100-270 days before were included in the screening visit. Other key inclusion criteria were 18–59 years old, healthy participants who can comply with the study protocol in the view of the treating physician, and participants who are capable of giving personal signed informed consent. Key exclusion criteria were previously RT-PCR-confirmed SARS-CoV-2 infection, history of allergy to any vaccine ingredient, pregnancy or planned pregnancy, breastfeeding, comorbidities that were considered severe or poorly controlled, or current use of immunosuppressants. Full inclusion and exclusion criteria can be found in the protocol in Supplementary Appendix 2.

### Randomisation and masking

An unblinded statistician created the computer-generated randomisation list. Participants were block-randomised (block size ten; ratio of 1:1:1:1:1) to receive ChAdTS-S, RQ3013, ZR202-CoV, CoronaVac or placebo. Participants, laboratory staff, and the research personnel, including those undertaking adverse event assessments or delivering the vaccines, were masked to treatment allocation. The analysing statisticians remained masked until the statistical analysis plan was signed off. Concealed random group allocations and blinding codes were kept in signed and sealed envelopes.

### Procedures

Participants who met the inclusion and exclusion criteria via the telephone screening were invited to a baseline visit (day 0). Participants who passed the final eligibility assessment and provided written informed consent were randomly assigned to a study group. After obtaining informed consent, participants were screened by medical history and targeted physical examination.

Among four COVID-19 vaccines used for intervention: ChAdTS-S (AdC68-19S) is a replication-deficient chimpanzee adenovirus-vectored vaccine with 5×10^10^ viral particles per 0.5 mL,^18^^,^^19^ RQ3013 is an mRNA vaccine containing 30 μg pseudouridine-modified mRNA encoding a mutated SARS-CoV-2 Spike protein harbouring mutations from both alpha and beta variants per 0.15 mL,^20^ ZR202-CoV is a recombinant protein vaccine with 25 μg prefusion-stabilized Spike ectodomain trimer per 0.5 mL of alum/CpG adjuvants,^21^ and CoronaVac is an inactivated vaccine (Sinovac, inactivated CN02 strain of SARS-CoV-2 with 3 μg per 0.5 mL of aluminium hydroxide adjuvant). Except for CoronaVac that contains the whole-virion as the immunogen, all other three vaccines were designed based on the Spike glycoprotein of SARS-CoV-2 as the immunogens. Specifically, ChAdTS-S and ZR202-CoV use Spike glycoprotein of the wild-type SARS-CoV-2 while RQ3013 uses a variant Spike glycoprotein containing all mutations from the alpha variant and additional K417N, E484K, A701V mutations from the beta variant as the immunogens (Table 1). Placebo consisted of 0.5 mL of 0.9% sodium chloride solution. All the five interventional regimens were administered via intramuscular injection into the upper arm by appropriately trained clinical research nurses at the trial site.

Participants were requested to stay for an additional 45-minute safety observation after vaccination. During the baseline visit, participants were given a thermometer and a tape measure. They were also asked to complete an electronic diary card (e-diary) to record solicited injection-site adverse events (pain, scleroma/swelling and redness), solicited systemic adverse events (acute allergy reaction, nausea/vomiting, joint pain, muscular pain, headache, chill, fatigue and fever) and unsolicited adverse events daily for 14 days. For days 15–28, they were asked to report the mentioned solicited and unsolicited adverse events for once. The study physicians reviewed the e-diary regularly to record adverse events. Serious adverse events and adverse events of special interest were monitored up to 6 months post-vaccination (the monitoring is still ongoing until August 2022). Participants reported the severity of their adverse events as Grade 1-4 as per definitions provided according to China National Medical Products Administration guidelines (https://www.nmpa.gov.cn/xxgk/ggtg/qtggtg/20191231111901460.html). Causality of adverse events was determined as related or unrelated to study treatment based on reasonable possibility, temporal relationship, and alternate cause criteria (see details in Supplementary Appendix 2).

The time points for subsequent visits for immunogenicity blood sampling were at day 0 before the booster dose, and at days 1, 4, 7, 14, 28, 90 and 180 after the booster dose to explore the kinetics and durability of the humoral and cellular responses. Serum samples were tested for the neutralising antibody titres against live SARS-CoV-2 virus, including wild-type strain (Wuhan-1, GenBank: MT123291), delta variant (B.1.617.2, IQTC-IM2175251) and omicron variant (BA.1.1, IQTC-Y216017), with cytopathic effect (CPE)-based microneutralisation assay as described in previous study.^2^ Briefly, serum was serially diluted from 1:8 to 1:5136, in serum free dulbecco's modified eagle medium (DMEM). The neutralising antibody titres were calculated as the reciprocal of the highest serum dilution showing more than 50% of inhibition (IC_50_) and seropositivity was defined as titre ≥ 1:8. Considering the accessibility in clinical setting, we also tested neutralising antibody using a competitive inhibition method measured by magnetic particle chemiluminescence immunoassay (MCLIA, Bioscience Co., China),^22^, ^23^, ^24^ and seropositivity was defined as ≥ 50 international unit (IU)/mL. Missing data was not imputed. Peripheral blood mononuclear cells (PBMCs) were tested for interferon (IFN)-γ, interleukin (IL)-4, and granzyme B-secreting T cells specific to whole Spike protein epitopes designed based on the wild-type SARS-CoV-2 sequence or the omicron variant (B.1.1.529) using a three-analytes FluoroSpot (Mabtech) assay (Figures S1-S2). T cell frequencies were reported as spot forming cells (SFCs) per million PBMCs. Details of immunological assessment methods and related procedures are described in Supplementary Appendix 2.

### Outcomes

The analysis of immunogenicity and safety within 6 months after boosting is planned in the study protocol but not completed yet. Here we report data collected within 90 days after vaccination. The primary immunogenicity outcomes include neutralising antibody titres measured by the live virus test against three variants of concern at four time points. The geometric mean titres (GMTs) of neutralising antibodies against the wild-type, delta and omicron variants of live SARS-CoV-2 at day 0 (pre-vaccination), 7, 14, and 28 after boosting vaccination were all considered as primary outcomes. The primary safety outcome was the occurrence of adverse events within 14 days after the booster vaccination. Immunological secondary outcomes include kinetics and durability of neutralising antibodies and cellular responses (measured by FluoroSpot). Incidence of adverse events and serious adverse events within 7 days, within 28 days, within 3 months and within 6 months were evaluated as secondary safety endpoints. A complete list of outcome measures can be found in the protocol in Supplementary Appendix 2.

### Statistical analysis

The sample size was determined by power analysis and phase 2 clinical trial standards. We powered on the immunogenicity outcome—the GMTs of neutralising antibody titres as measured by live virus assay. We assumed a standard deviation of the GMT on log scale (base 10) to be 0.4 based on the current available data. This study is designed to have 90% power to detect 3-fold difference in the GMTs of neutralising antibodies between each COVID-19 vaccine group with the inactivated vaccine group or to detect 5-fold difference between each vaccine group with the placebo control. Since we made at most four comparisons for each outcome measure when comparing each of four vaccines with the placebo, applying a Bonferroni correction would require to adapt to a significance level of 0.05/4=0.0125. In this study, we focused on the objective of testing the superiority of three heterologous regimens to the homologous regimen, which made three comparisons. To account for multiple testing, a conservative two-sided significance level of 0.01 was used. We inflated the required sample size by 25% to take into account of participants who would be failed for screening or lost to follow-up, recruiting 50 participants per group.

**Figure 1:**
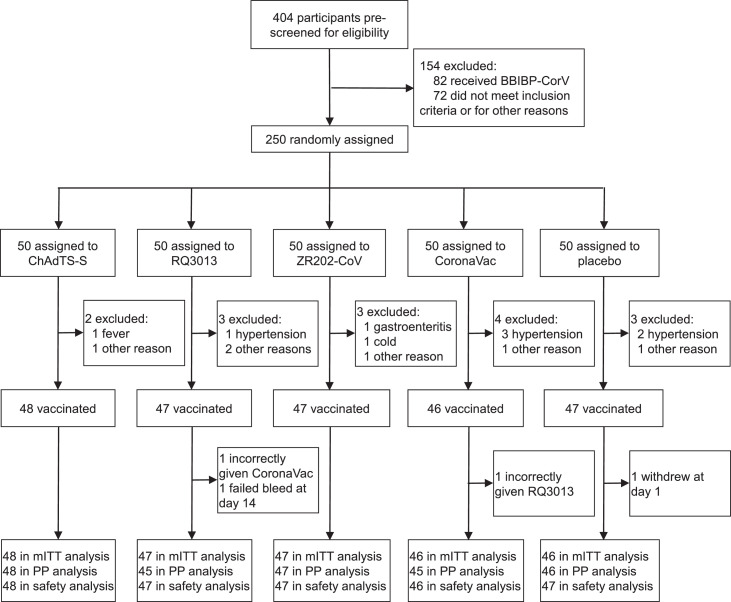
**Trial profile.** mITT=modified intention-to-treat population, placebo=0.9% sodium chloride solution for injection, PP=per-protocol. One participant in placebo group withdrew at day 1 after vaccination because of a serious adverse event. *One participant was randomised to inactivated vaccine group (CoronaVac) but was incorrectly administered an mRNA vaccine (RQ3013) and then reclassified into mRNA group. On the other hand, another participant was randomised to mRNA vaccine group but was incorrectly administered an inactivated vaccine and then reclassified into inactivated vaccine group.

We assessed immunogenicity outcomes in the modified intention-to-treat (mITT) population (Figure 1). This population included all participants who completed their booster doses and had at least one post-dose immunogenicity data. Summaries of baseline characteristics are reported for all enrolled participants in the mITT analysis. The primary immunogenicity outcomes of neutralising antibodies against SARS-CoV-2 (wild-type, delta and omicron) were reported as GMTs and 95% confidence intervals (CIs). We fitted generalized linear model to log-transformed antibody titres at each follow-up time (day 7, 14, 28 and 90) separately to calculate geometric mean ratios (GMRs) between groups by adjusting for baseline antibody titres (at day 0 before vaccination), intervals between the first and second vaccine doses, and intervals between the second and third doses. For subgroup analyses, the interaction terms, the prime-boost time interval and sex, were included in the linear mixed regression model. Spearman non-parametric correlation analysis was applied to humoral and cellular responses.

To test the robustness of the immunogenicity result at day 14 (the peak time point of antibody responses), we repeated the superiority comparison between heterologous regimens and homologous regimen as well as the comparison between all four vaccines and the placebo in the per-protocol (PP) population. This population is compliant with the key procedures of the protocol including completing the vaccination within the time window as required in the protocol and all blood samplings for primary outcomes (day 0, days 7, 14 and 28 after vaccination).

Safety outcomes were assessed in the safety population, which included all participants who received the booster vaccine. The proportion of participants with at least one adverse event was reported by the vaccine schedules. Fisher's exact test was used to compare the differences across study schedules. All statistical analyses were done using R (version 4.2.1). For the primary analyses on immunogenicity (comparing each study vaccine arm with inactivated vaccine or placebo as controls), statistical significance was determined at 2-sided *p*<0.01. The significance level for all the other secondary analyses will be 2-sided 0.05, unless otherwise specified in the analysis section below.

### Role of the funding source

The funders of the study had no role in study design, data collection, data analysis, data interpretation, or writing of the report. Z.Z. and Y.Z. have accessed and verified the data, and Z.Z., J.W., D.Y., T.C.Z. and Z.W. were responsible for the decision to submit for publication.

## Results

Between January 1, 2022 and February 28, 2022, 404 participants were screened and 250 of them were randomised, among whom 235 were enrolled in the study and received a third dose vaccination. 234 were included in the mITT analysis and 231 were included in the PP analysis (Figure 1). The median age of the 234 participants included in the mITT analysis was 28 years (interquartile range [IQR] 24-34), with 53.85% (126/234) females. The median interval was 24 days (IQR 22-27 days) between the first two doses of CoronaVac, and 196 days (IQR 159-199 days) between the primary and booster immunisation. The neutralising antibody titres against the wild-type virus, the delta and omicron variants were low or absent at baseline. By contrast, participants showed high positive rate (83%–100%) of IFN-γ- and granzyme B-secreting cell responses to both the wild-type and the omicron variant of SARS-CoV-2 at baseline. Demographic characteristics and immunogenicity were well balanced across five groups (Table 2).

**Table 2:** Baseline characteristics of primary analysis population.

	ChAdTS-S (*n*=48)	RQ3013 (*n*=47)	ZR202-CoV (*n*=47)	CoronaVac (*n*=46)	Placebo (*n*=46)	*p*
**Age, years**						
mean, SD	28.5±6.1	29.0±6.0	29.7±6.4	29.8±6.1	28.5±6.7	0.1192
median, IQR	27.0 (23.8,31.3)	27.0 (25.0,33.0)	28.0 (24.0,35.0)	29.0 25.0,34.0)	26.0 (23.0,34.0)	0.0583
**Sex, n (%)**						
Male	24.0 (50.0)	21.0 (44.7)	20.0 (42.6)	25.0 (54.3)	18.0 (39.1)	0.6044
Female	24.0 (50.0)	26.0 (55.3)	27.0 (57.4)	21.0 (45.7)	28.0 (60.9)	
**Dose interval, days, median (IQR)**						
The first and second dose interval	24.0 (22.0,26.3)	24.0 (23.0,26.5)	24.0 (22.0,29.0)	24.5 (23.0,29.0)	24.0 (22.0,27.0)	0.0492
The prime-boost dose interval	191.0 (158.8,198.3)	196.0 (163.0,199.0)	192.0 (158.0,198.0)	193.5 (159.0,198.0)	196.0 (158.0,199.0)	0.3453
**Intervals between the second and third doses, n (%)**						
≥180 days	25.0 (52.1)	29.0 (61.7)	25.0 (53.2)	26.0 (56.5)	29.0 (63.0)	0.7612
<180 days	23.0 (47.9)	18.0 (38.3)	22.0 (46.8)	20.0 (43.5)	17.0 (37.0)	
**Neutralising antibodies against live SARS-CoV-2 at day 0 before vaccination, GMT (95% CI)**						
wild-type	4.8 (4.3-5.3)	4.6 (4.1-5.1)	5.1 (4.4-5.9)	4.4 (4.0-4.8)	5.4 (4.7-6.3)	0.1181
delta	4.4 (3.7-5.3)	4.5 (3. 7-5.4)	4.6 (3.9-5.3)	4.2 (4.0-4.4)	4.5 (4.1-5.0)	0.9318
omicron	4.0 (4.0-4.0)	4.0 (4.0-4.0)	4.0 (4.0-4.0)	4.0 (4.0-4.0)	4.0 (4.0-4.0)	-
**Seropositive rate of neutralising antibodies against live SARS-CoV-2 at day 0 before vaccination, n (%)**						
wild-type	10.0 (20.8)	7.0 (14.9)	11.0 (23.4)	5.0 (10.9)	14.0 (30.4)	0.1567
delta	2.0 (4.2)	2.0 (4.3)	3.0 (6.4)	3.0 (6.5)	6.0 (13.0)	0.4291
omicron	0.0 (0.0)	0.0 (0.0)	0.0 (0.0)	0.0 (0.0)	0.0 (0.0)	-
**T cell responses against live wild-type SARS-CoV-2, SFC/million PBMCs, median (positive rate)**						
IFN-γ	282.0 (98%)	248.0 (90%)	232.0 (88%)	200.0 (90%)	318.0 (88%)	0.5327
granzyme B	268.0 (100%)	296.0 (90%)	256.0 (85%)	210.0 (83%)	306.0 (92%)	0.9136
IL-4	0.0 (43%)	0.0 (44%)	0.0 (44%)	0.0 (43%)	20.0 (51%)	0.9758
T cell responses against live omicron SARS-CoV-2, SFC/million PBMCs, median (positive rate) *						
IFN-γ	1008.0 (100%)	346.0 (95%)	528.0 (95%)	360.0 (89%)	640.0 (95%)	0.1455
granzyme B	944.0 (100%)	756.0 (100%)	590.0 (100%)	568.0 (94%)	708.0 (100%)	0.2086
IL-4	24.0 (61%)	0.0 (48%)	10.0 (50%)	0.0 (33%)	0.0 (33%)	0.3895

Note: IQR=interquartile range; PBMC= peripheral blood mononuclear cells; SARS-CoV-2=severe acute respiratory syndrome coronavirus 2; SD=standard deviation; SFC=spot forming cells. Neutralising antibodies against live SARS-CoV-2 virus, including wild-type strain (Wuhan-1, GenBank: MT123291), delta variant (B.1.617.2, IQTC-IM2175251) and omicron variant (BA.1.1, IQTC-Y216017), were tested with cytopathic effect (CPE)-based microneutralisation assay. Seropositivity was defined as titre ≥ 1:8. * T cell response against omicron variant of live SARS-CoV-2 was determined in samples from a random subset of the 234 participants (*n=*23 for ChAdTS-S, *n=*21 for RQ3013, *n=*22 for ZR202-CoV, *n=*21 for CoronaVac, and *n=*21 for placebo).

**Figure 2:**
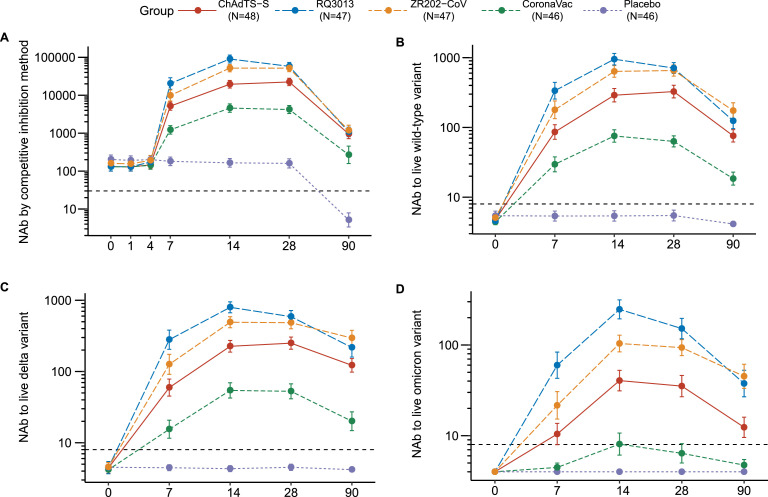
**Kinetics of neutralising antibody responses after the third dose vaccination by booster schedules. (A)** The neutralising antibody titres were measured by competitive inhibition method. The dash line indicates seropositivity cutoff value of ≥ 1:50. **(B-D)** The neutralising antibody titres were determined with cytopathic effect (CPE)-based microneutralisation assay using authentic SARS-CoV-2 virus, including wild-type strain (Wuhan-1, GenBank: MT123291), delta variant (B.1.617.2, IQTC-IM2175251) and omicron variant (BA.1.1, IQTC-Y216017). The dash line indicates seropositivity cutoff value of ≥ 1:8.

**Figure 3:**
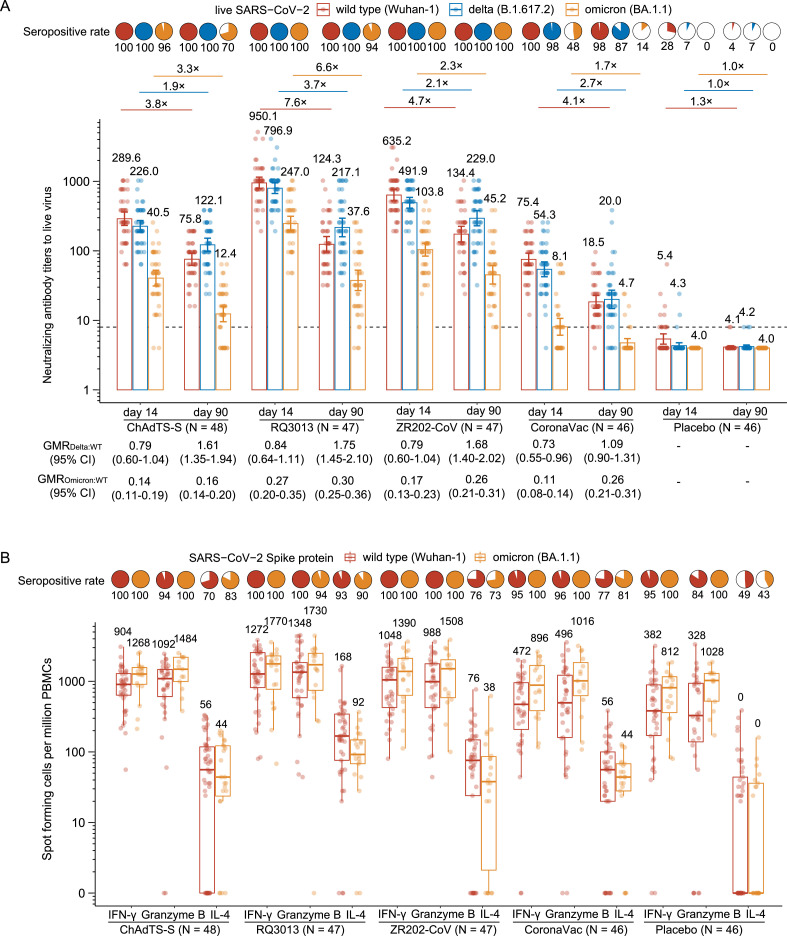
**Humoral as well as cellular responses after the third dose vaccination by booster schedules.** (A) The 50% neutralisation antibody titres against the wild-type, the delta and omicron variants of live SARS-CoV-2 were determined with cytopathic effect (CPE)-based microneutralisation assay using authentic SARS-CoV-2 virus, including wild-type strain (Wuhan-1, GenBank: MT123291), delta variant (B.1.617.2, IQTC-IM2175251) and omicron variant (BA.1.1, IQTC-Y216017). Data are presented as the geometric mean titres with 95% confidence intervals. The titres for individual participants are indicated with datapoints. The geometric mean ratio (GMR) in titres of the delta and omicron variants versus titres against the wild-type of SARS-CoV-2 are shown below the graph. Seropositive rates by booster schedules are also presented. (B) The interferon (IFN)-γ, interleukin (IL)-4 and granzyme B-secreting T cells after stimulating peripheral blood mononuclear cell (PBMCs) with peptides of whole Spike protein epitopes designed based on the wild-type SARS-CoV-2 sequence or the omicron variant (B.1.1.529) were measured by FluoroSpot assay. *T cell response against omicron variant of live SARS-CoV-2 was determined in samples from a random subset of the 234 participants (*n*=23 for ChAdTS-S, *n*=21 for RQ3013, *n*=22 for ZR202-CoV, *n*=21 for CoronaVac, and *n*=21 for placebo). Data are presented as median and interquartile range values.

We first explored the dynamics of antibody responses to the booster dose by measuring the neutralising antibodies using a competitive inhibition method, which is more accessible at clinical laboratory, at day 0 (before the booster dose), days 1, 4, 7, 14, 28 and 90 after the booster dose (Table S1). The baseline neutralising antibodies were close to 50 IU/mL across all groups assessed and remained largely unchanged until day 4. We observed a rapid increase in neutralising antibodies at day 7, a peak at day 14, a slight waning at day 28 and a continued declination at day 90 (Figure 2A). Neutralising antibody titres to live wild-type, delta and omicron variants of SARS-CoV-2 showed similar trends (Figure 2B-D, Table S2). At day 14, in which neutralising antibodies peaked, GMRs between neutralising antibodies against variants and wild-type virus were 0.79, 0.84, 0.79, and 0.73 for the delta variant, but were 0.14, 0.27, 0.17, and 0.11 for the omicron variant in individuals receiving ChAdTS-S, RQ3013, ZR202-CoV and CoronaVac, respectively (Figure 3A). We note that despite of reduced neutralising antibodies against the omicron variant due to immune escape, seropositive rates were near 100% in all heterologous schedules, as compared to 47.8% in the homologous schedule. Durability at day 90 revealed that neutralising antibodies against omicron remained mostly seropositive with titres ≥ 1:8 in heterologous boost schedules while homologous boosting with CoronaVac showed only 14% seropositivity. Interestingly, although mRNA vaccine RQ3013 demonstrated superior immunogenicity than other vaccine types as a booster dose at day 14 after vaccination, the recombinant protein vaccine ZR202-CoV with adjuvants containing CpG showed a slightly better durability against the omicron variant 3 months after the booster dose with a GMR of 3.6, 1.2 and 9.7 comparing to ChAdTS-S, RQ3013 and CoronaVac, respectively.

At day 14 after vaccination when live virus neutralising antibody peaked, GMRs between studied vaccines and placebo control ranged from 15.1 (95%CI 11.5-19.8) in CoronaVac to 190.9 (95%CI 145.8-249.9) in RQ3013 (Table 3). Notably, more remarkable differences across vaccines were observed for GMRs against the omicron variant, with 62.4 (95%CI 45.8-85.1) in RQ3013, followed by 26.0 (95%CI 19.1-35.4) in ZR202-CoV, 10.3 (95%CI 7.6-14.0) in ChAdTS-S, and 2.0 (95%CI 1.5-2.7) in CoronaVac. We observed varying neutralising antibody titres to live wild-type, delta and omicron variants of SARS-CoV-2 across boosting regimens, with RQ3013 exhibiting the highest neutralisation against all three strains. Superiority comparisons between heterologous and homologous schedules showed consistent results as previous studies among population primed with CoronaVac/CoronaVac.^10^^,^^13^ Neutralising antibody responses against all three SARS-CoV-2 strains induced by heterologous booster doses were superior to the homologous booster dose (Table 3). Notably, heterologous boosting with RQ3013 induced a 31.3-fold stronger antibody response against the omicron variant compared with homologous booster with CoronaVac. This is significantly higher than the BNT162b2/CoronaVac GMRs observed in previous studies in population primed with CoronaVac.^10^^,^^13^ The lowered immune escape by the omicron variant in heterologous-booster-elicited antibodies suggests that heterologous boosting, especially the mRNA vaccine RQ3013, can induce broader-spectrum neutralising antibodies against variants of concerns. Similar results were observed in the PP population (Table S3).

**Table 3:** Geometric mean titre and geometric mean ratio of humoral responses at day 14 after the third dose vaccination.

	ChAdTS-S (*n=*48)	RQ3013 (*n=*46)	ZR202-CoV (*n=*47)	CoronaVac (*n=*46)	Placebo (*n=*46)
**Neutralising antibodies against the wild-type of live SARS-CoV-2**					
GMT	289.6	950.1	635.2	75.4	5.4
	(232.2-361.2)	(785.4-1149.3)	(524.0-770.1)	(61.4-92.5)	(4.5-6.4)
GMR1	56.8	190.9	120.8	15.1	Ref
	(43.6-74.1)	(145.8-249.9)	(92.6-157.6)	(11.5-19.8)	-
GMR2	3.8	12.6	8.0	Ref	-
	(2.9-4.9)	(9.6-16.5)	(6.1-10.4)	-	-
p1	<0.0001	<0.0001	<0.0001	<0.0001	Ref
p2	<0.0001	<0.0001	<0.0001	Ref	-
**Neutralising antibodies against the delta variant of live SARS-CoV-2**					
GMT	226.0	796.9	491.9	54.3	4.3
	(187.2-273.0)	(667.7-951.1)	(411.6-587.9)	(42.4-69.4)	(3.9-4.8)
GMR1	52.8	185.0	113.9	12.6	Ref
	(41.0-68.1)	(143.2-239.1)	(88.3-147.0)	(9.7-16.3)	-
GMR2	4.2	14.7	9.1	Ref	-
	(3.3-5.4)	(11.4-19.1)	(7.0-11.7)	-	-
p1	<0.0001	<0.0001	<0.0001	<0.0001	Ref
p2	<0.0001	<0.0001	<0.0001	Ref	-
**Neutralising antibodies against the omicron variant (BA.1.1) of live SARS-CoV-2**					
GMT	40.5	247.0	103.8	8.1	4.0
	(31.2-52.5)	(194.1-314.3)	(84.0-128.3)	(6.1-10.7)	(4.0-4.0)
GMR1	10.3	62.4	26.0	2.0	Ref
	(7.6-14.0)	(45.8-85.1)	(19.1-35.4)	(1.5-2.7)	-
GMR2	5.2	31.3	13.0	Ref	-
	(3.8-7.0)	(22.9-42.7)	(9.6-17.7)	-	-
p1	<0.0001	<0.0001	<0.0001	<0.0001	Ref
p2	<0.0001	<0.0001	<0.0001	Ref	-
**Neutralisation antibodies measured by competitive inhibition method**					
GMT	19512.9	90384.3	51995.0	4588.2	165.2
	(15498.4-24567.1)	(71218.5-114707.9)	(41583.4-65013.4)	(3558.8-5915.5)	(127.2-214.7)
GMR1	138.4	640.6	345.7	33.2	Ref
	(101.8-188.2)	(469.7-873.8)	(254.5-469.6)	(24.3-45.3)	-
GMR2	4.2	19.3	10.4	Ref	-
	(3.1-5.7)	(14.2-26.3)	(7.7-14.2)	-	-
p1	<0.0001	<0.0001	<0.0001	<0.0001	Ref
p2	<0.0001	<0.0001	<0.0001	Ref	-

Note: Geometric mean ratio (GMR) was calculated using linear regression model by adjusting for the baseline neutralising antibody levels at day 0 before the booster dose vaccination, the first and second dose interval, and the second and third dose interval. GMR1 and p1 were calculated by comparing to placebo group, while GMR2 and p2 were calculated by comparing to the CoronaVac group.

We next analysed the influence of 2nd-3rd-dose interval and sex on humoral immune response. Subgroup analyses revealed a higher antibody response for male than female in ChAdTS-S while all other booster regimens had no significant differences by prime-boost interval and sex (Figures S3-S13).

For cellular responses, the fraction of IFN-γ-secreting type 1 helper T (Th1) cells and granzyme B-secreting cytotoxic cells in PBMCs reached peak levels across all vaccine groups at day 7 or day 14 post-vaccination and were detectable in most (94%–100%) individuals (Figure 3B, Tables S4–S5). At day 14 after the booster vaccination, the medians of IFN-γ+ SFC per million PBMCs against wild-type SARS-CoV-2 were 904 (IQR 636-1288), 1272 (IQR 816-2552), 1048 (IQR 426-1586), 472 (IQR 212-956), and 382 (IQR 171-891); and granzyme B+ SFC per million PBMCs were 1092 (IQR 608-1472), 1348 (IQR 586-1844), 988 (IQR 424-1772), 496 (IQR 160-1220), and 328 (IQR 139-938) in individuals receiving ChAdTS-S, RQ3013, ZR202-CoV, CoronaVac, and placebo, respectively. Conversely, RQ3013 induced measurable IL-4+ T cells against wild-type SARS-CoV-2 in >90% individuals, while measurable IL-4+ T cells were detected in relatively fewer participants for other vaccines (70%-80%). The medians of IL4+ SFC per million PBMCs ranged from 56 (IQR 20-100) in CoronaVac to 168 (IQR 76-344) in RQ3013 at day 14 after the booster vaccination. Interestingly, unlike the neutralising immune escape (Figure 3A), cellular responses against the omicron variant of SARS-CoV-2 were largely remained (Figure 3B, Table S6). Superiority comparisons between heterologous and homologous schedules showed that RQ3013 induced the strongest cellular responses against both the wild-type and the omicron variant of SARS-CoV-2 Spike protein (Tables S7–S8). None booster regimens showed significant difference by prime-boost interval and sex (Figures S14-S22).

As expected, we observed strong correlations between neutralising antibodies against the live wild-type SARS-CoV-2 with those against the delta and omicron variants, and neutralising antibodies measured by competitive inhibition method among all vaccines (Figure S23). Similarly, cellular response against the wild-type and omicron variant of SARS-CoV-2 Spike protein showed strong correlations (Figure S24). However, boosting schedules exhibited only weak correlations between humoral and cellular immune responses against the wild-type or omicron variant of SARS-CoV-2 Spike protein (Figures S25–S26).

**Figure 4:**
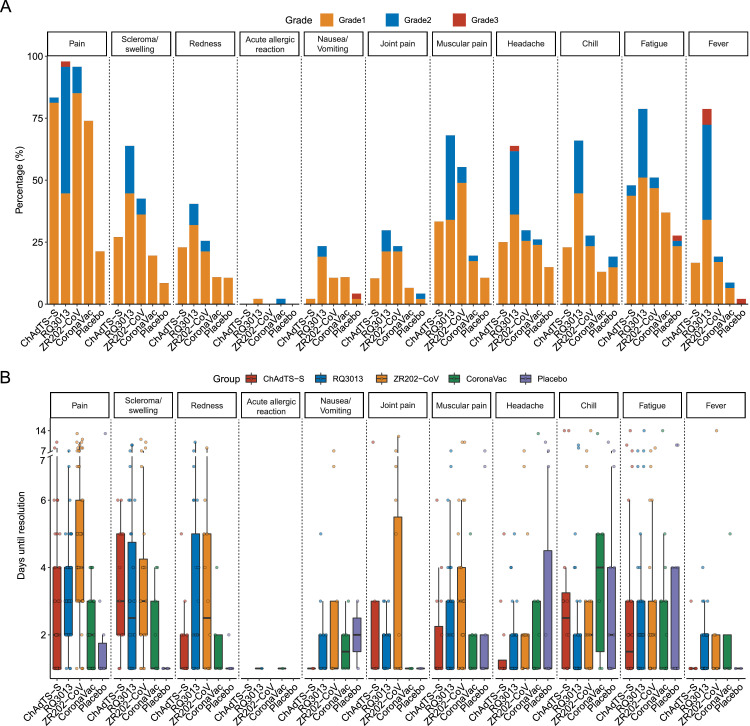
**Incidence of solicited adverse events and days until resolution within 14 days after the third dose vaccination by booster schedules.** (A) The percentage of participants who reported local/systemic adverse events are presented. The maximum severity of respective solicited adverse event recorded for each participant within 14 days after the booster vaccination is shown. (B) The days on resolution of the reported solicited adverse events are presented. Datapoints represent individual participants, and bar and box show median and interquartile range values.

After booster vaccination, the most common local and systemic adverse events after the booster doses were injection site pain and cold like symptoms (muscle pain, headache, chill, fatigue and fever), respectively (Figure 4A). Adverse events were most commonly observed in RQ3013, followed by ZR202-CoV and ChAdTS-S, and then CoronaVac and placebo, most (>90%) of which were mild (grade 1) or moderate (grade 2) (Tables S9-S11) and typically resolved within 2-3 days post-vaccination (Figure 4B and Table S12). Incidences of grade 3 solicited local or systemic adverse events after the booster vaccination were only observed in RQ3013 and placebo groups at 8.5% and 4.3% occurrence rates, respectively (Tables S13–S15). There was no grade 4 solicited local or systemic adverse events or serious adverse events or SARS-CoV-2 infection occurred in all study vaccine groups through the 3-month period after boosting vaccination (Table S16).

## Discussion

This clinical trial describes the immunogenicity, durability and safety of a booster dose of four vaccines developed by different platforms in China, including an mRNA vaccine encoding the Spike protein with mutations in alpha and beta variants reported in an RCT for the first time, in people who had been vaccinated with 2-dose schedule of CoronaVac 4–7 months before the third dose. Consistent with previous studies, we observed very low neutralising antibody GMT against live SARS-CoV-2 wild-type, delta and omicron strains 4–7 months post the two-dose vaccination with CoronaVac.^4^^,^^5^^,^^10^^,^^13^ Our data highlights three key findings. First, we found remarkable differences of boosting effects between vaccine regimens (ChAdTS-S, RQ3013, ZR202-CoV or CoronaVac), with RQ3013 showing the highest levels in the magnitude and broadness of neutralising antibodies as well as cellular responses but ZR202-CoV showing a slightly better durability. Second, neutralisation GMTs didn't increase until 4 days and reached the peak level at 14 days after the booster dose, which then waned at 28 days post-vaccination, but largely retained at 3 months. Third, comparing with homologous booster regimen, reactogenicity was significantly higher in heterologous booster schedules, but was all well-tolerated. Overall, our data informed public and policy makers with immunological, durability and reactogenicity data of four COVID-19 vaccines from various platforms as a booster dose along with homologous schedule with inactivated vaccine in people primed with two doses of CoronaVac. Our clinical evidence added more vaccine candidates that are effective against the omicron variant to the booster vaccination campaign toolbox in China and many countries worldwide, addressing insufficient vaccine-induced immunity for a largest population primed with two-dose inactivated COVID-19 vaccine.

We used both the gold-standard live virus neutralisation test and the clinically more approachable competitive inhibition method (mostly automated) to measure neutralising antibody titres in this study, with good correlation with each other (Figure S23). We observed a consistent baseline antibody levels as previous studies that neutralising antibodies declined to near or below the detection limit after 4–7 months in participants primed with CoronaVac.^4^^,^^5^^,^^10^^,^^13^ All four studied booster schedules demonstrated superior immunogenicities to placebo control. At day 14, neutralisation antibody GMTs against wild-type virus increased to 161 times (RQ3013), 115 times (ZR202-CoV), 57 times (ChAdTS-S), and 15 times (CoronaVac) higher than the placebo control.

Our immunogenicity data showed that heterologous prime-boost regimens were superior to the homologous boost schedule, particularly in neutralisation against the omicron variant. Specifically, the GMRs of neutralising antibodies against the omicron variant between heterologous regimens and the homologous at day 14 were 5.2 for ChAdTS-S (adenovirus-vectored), 31.3 for RQ3013 (mRNA), and 13.0 for ZR202-CoV (recombinant protein), respectively. This is in line with recent heterologous booster dose studies in people primed with inactivated-vaccine that mRNA vaccine (BNT162b2 or mRNA1273) appeared to induce higher neutralising antibody titres than other vaccines as the booster dose.^10^^,^^13^^,^^25^, ^26^, ^27^, ^28^ In addition, consistent with previous reports,^29^^,^^30^ T cell responses against the omicron variant of SARS-CoV-2 are preserved in most individuals after primary vaccination and further enhanced by the booster dose, especially by RQ3013. Consistent with these immunogenicity data, the real-world studies on population primed with inactivated-vaccine showed that a booster dose with mRNA vaccine (BNT162b2) provided a higher rate of protection against symptomatic and severe COVID-19 than inactivated vaccine (CoronaVac).^31^^,^^32^ For example, the real-world study from Brazil, which paid special attention to the period of omicron predominance, revealed protection efficiency of 84.1% (95% CI 83.2–84.9) against severe outcomes at more than 120 days after BNT162b2 booster in participants primed with CoronaVac.^32^ This is in accordance with the booster dose immunogenicity superiority of BNT162b2 over CoronaVac (GMR of neutralising antibodies against live omicron virus = 13.1) among people primed with CoronaVac reported in the RHH-001 study.^10^ Interestingly, superiority comparisons comparing heterologous and homologous boosting schedule revealed a GMR of 23.7 for RQ3013 on neutralisation data against the omicron, which is approximately two times higher than the GMRs of 11.3-13.1 for BNT162b2.^10^^,^^13^ Conversely, GMRs on neutralisation data against the wild-type and delta strains demonstrated an advantage of BNT162b2 over RQ3013 as a booster dose (Table S17). We note that GMRs discussed above were all based on neutralising antibody titres to live virus at 28 days after the booster dose in people primed with 2-dose CoronaVac and hence comparable across studies. We reason that the advantage of RQ3013 over BNT162b2 on the omicron variant and disadvantage on the wild-type and delta variant might be attributed to the design of the immunogen (Spike protein) containing mutations of the alpha and beta variants, which is closer to the omicron variant on the phylogenetic tree than the wild-type Spike in BNT162b2 (Figure S27). Taking together, we can reasonably assume that RQ3013 would likely provide better protection than BNT162b2 during the omicron predominance.

Subgroup analysis of prime-boost interval could inform policy makers about the optimal time for booster vaccination. A longer interval between the prime and the booster has been reported to be associated with higher levels of neutralising antibodies, as the affinity maturation of memory B cells induced by vaccination could take months. For example, prolonged booster interval with 4 months showed higher titres of neutralising antibodies than the 1-month interval of ZF2001.^33^ A third dose of CoronaVac in adults vaccinated 8 months after the second dose induced much higher neutralising antibody than those given 2 months after a second dose (GMT 49.7 *vs*. 143.1).^5^ However, humoral immunity after the two-dose CoronaVac immunisation waned significantly after 3 months. Therefore, the need for a booster dose is as early as 3 months after primary immunisation, while too early booster vaccination may result in a sub-optimal humoral immune response. Our data showed that there was no significant difference in the boosting effect of both humoral and cellular responses from 4 to 7 months after the primary immunisation. This is consistent with previous studies showing equivalent immunogenicity of heterologous boosters given as early as 3 months after primary CoronaVac vaccination to that given at a longer prime-boost interval.^12^^,^^34^^,^^35^

This study included relative dense time points, providing a more comprehensive immune response dynamic to the booster dose. Our data indicates that the effective level of neutralising antibodies can be detected at day 7, but no earlier than day 4 after the booster vaccination. The gain of cellular immune responses was faster as T cell responses peaked at day 7 instead of day 14 for humoral immunity. These results support the rationale for evaluating vaccine effectiveness at least 7 days after receipt of the third dose in real-world studies.^36^, ^37^, ^38^ Our durability analysis at day 90 revealed that although RQ3013 showed the highest level of neutralization to the omicron variant, the recombinant protein vaccine with adjuvants containing CpG showed slightly higher neutralizing antibodies 3 months after the booster dose. We note that all four studied vaccines showed rapid declination of antibody titres 3 months after the booster dose, consistent with previous reports of waning immunity after the primary immunization.^39^^,^^40^ Therefore, a vaccine that can elicit high level and broad-spectrum neutralising antibodies is the key to provide longer protection against infection. Additionally, comparative study of immune memory against SARS-CoV-2 at 3- and 6-month post booster vaccination is needed to determine optimal schedule for further booster doses.

All four study COVID-19 vaccines reported acceptable reactogenicity. Consistent with previous reports, heterologous booster regimens showed more adverse events than homologous regimen with CoronaVac.^41^ Compared with other trials of homologous and heterologous third dose boosters, we observed a systematic shift towards a higher frequency of adverse events. This can be partially explained by the fact that the overall number of younger participants in our study is higher than that in previous studies.^10^^,^^17^

Vaccines from different platforms may have different advantages and disadvantages, but are usually hard to compare with each other across studies. By studying vaccines of multiple platforms in parallel, this study revealed varying levels of neutralising antibodies, broadness of antibodies against variants of concern, T cell responses and reactogenicity profiles of four vaccines available in China (Figure S28). Among them, RQ3013 demonstrated the highest neutralising antibodies and T cell responses, but with the highest reactogenicity. ZR202-CoV and ChAdTS-S showed intermediate immunogenicity, but with a slower decline within 90 days after the booster vaccination, although the longer effects at 6 months remained to be investigated. Homologous booster with CoronaVac showed the lowest immunogenic effect, but reported the lowest incident rate of adverse events. Thus, heterologous boosting with RQ3013 could provide more effective protection for people with higher risk of infection and vulnerability while homologous boosting with CoronaVac could reduce vaccine hesitation for people concerning about side effects of vaccination.

There are several limitations to be mentioned. First, since the pandemic is well contained in China, we cannot assess vaccine efficacy in a real-world study. However, evidence has been accumulating that neutralising antibodies correlate well with protection against symptomatic SARS-CoV-2 infection.^42^, ^43^, ^44^ In this scenario, our results can still provide important comparisons across booster regimens. Second, participants are all Asians aged 18–59 years without older adults, who are often immunocompromised or have underlying medical conditions. Further clinical studies of more races and seniors with or without common chronic diseases are needed to inform the booster strategy in these specific populations. Third, we evaluated the immunogenicity using the omicron BA.1.1 (BA.1 + R346K) in the live virus test while data on the newly emerged BA.2, BA.4/BA.5 and BA.2.75 are still missing. A recent study reported a substantial reduction in cross-neutralisation against BA.4/BA.5 by serum from BA.1 infected patients.^45^ Neutralisation activity against the latest variants will be of great interest in the future studies.

In conclusion, this trial has demonstrated the potential of four different vaccines tested (ChAdTS-S, RQ3013, ZR202-CoV and CoronaVac) to boost immunity following an initial course of CoronaVac/CoronaVac. The mRNA vaccine RQ3013 optimised the magnitude and breadth of immune responses than the others. Although the durability of RQ3013 at day 90 was a bit inferior to that of ChAdTS-S and ZR202-CoV, the neutralizing antibodies against the omicron variant of all the three heterologous boosting regimens were largely retained. Our results demonstrated the advantage of variant immunogen design in mRNA COVID-19 vaccine development and added more effective vaccine candidates against the omicron variant.

## Contributors

Z.Z. and J.W. conceived the trial; Z.Z., Y.Z., D.Y., Y.W. and Y.C. contributed to the protocol and design of the trial; Z.Z., J.W., D.Y. and T.C.Z. supervised the implementation of the trial; Y.Z., G.Y., Z-M.Z., W.S., F.W.L., M.X.D., L.Z., C.L. and X.Y. contributed to the trial implementation; Z.Z., Y.Z., D.Y., X.M., G.Y., Z-M.Z. and Y.W. curated the data; Z.Z. and Y.Z. had access to and verified the study data; D.Y., Y.Z., G.Y. and Y.W. did the statistical analysis; Z.W., X.M., Z-M.Z., N.W., W.S. and M.Y. did the laboratory work; Y.Z. and Z.Z. wrote the original draft. All the authors critically reviewed and approved the final version.

## Data sharing statement

De-identified data are freely available from the corresponding author upon request. All codes associated with data analysis are freely available from the corresponding author upon request.

## Declaration of interests

ZJZ served as a PI in a phase 4 clinical study sponsored by Sinovac Biotech Ltd. All other authors declare no competing interests.

## Appendix Supplementary materials

Image, application 1

Image, application 2
