# Supplementary material for: Immunogenicity, durability, and safety of an mRNA and three platform-based COVID-19 vaccines as a third dose following two doses of CoronaVac in China: A randomised, double-blinded, placebo-controlled, phase 2 trial
Source: eClinicalMedicine. 2022 Sep 28;54:101680. doi: 10.1016/j.eclinm.2022.101680 (PMC9517939; doi:10.1016/j.eclinm.2022.101680)
Supplement: Supplementary file 1 [file mmc1.pdf]

Immunogenicity, durability, and safety of an mRNA and three platform-based COVID-19 vaccines as a third dose following two doses of CoronaVac in China: a randomised, double-blinded, placebo-controlled, phase 2 trial

Appendix 1: Supplementary Tables and Figures

Appendix 2: Trial Protocol and Statistical Analysis Plan

Appendix 3: Precise-CoVaccine study group members

Immunogenicity, durability, and safety of an mRNA and three platform-based COVID-19 vaccines as a third dose following two doses of CoronaVac in China: a randomised, double-blinded, placebo-controlled, phase 2 trial

Appendix 1-

Supplementary Tables and Supplementary Figures

Contents

Supplementary Tables..6

Supplementary Table 1: Kinetics of neutralising antibody measured by competitive inhibition method across booster schedules...................................... 6

Supplementary Table 2: Kinetics of neutralising antibodies against the wild-type, delta and omicron variants of live SARS-CoV-2 across booster schedules...........7

Supplementary Table 3: Geometric mean titre and geometric mean ratio of humoral responses at day 14 after the third dose vaccination analysed in the per-protocol population...9

Supplementary Table 4: Kinetics of T cell responses against the wild-type of SARS-CoV-2 Spike protein across booster schedules.. 10

Supplementary Table 5: Kinetics of T cell responses against the omicron variant of SARS-CoV-2 Spike protein across booster schedules....................................11

Supplementary Table 6: T cell responses against the wild-type and omicron variant of SARS-CoV-2 Spike protein across booster schedules.........................12

Supplementary Table 7 : Comparison of T cell responses against the wild-type of SARS-CoV-2 Spike protein across booster schedules.. 13

Supplementary Table 8 : Comparison of T cell responses against the omicron variant of SARS-CoV-2 Spike protein across booster schedules.........................15

Supplementary Table 9: Local and systemic adverse events reported within 7 days after the third dose vaccination...17

Supplementary Table 10: Local and systemic adverse events reported within 14 days after the third dose vaccination across booster schedules............................19

Supplementary Table 11: Local and systemic adverse events reported within 28 days after the third dose vaccination... 21

Supplementary Table 12: Days until resolution of solicited/unsolicited adverse events reported within 14 days after the third dose vaccination...........................23

Supplementary Table 13: Grade 3 adverse events reported within 7 days after the third dose vaccination..24

Supplementary Table 14: Grade 3 adverse events reported within 14 days after the third dose vaccination...25

Supplementary Table 15: Grade 3 adverse events reported within 28 days after the third dose vaccination...26

Supplementary Table 16: The three serious adverse events reported within 3 months in this study........27
Supplementary Table 17: Booster immunogenicity effects of RQ3013 and BNT162b2 on the basis of two-dose of CoronaVac........28
Supplementary Figures........29
Supplementary Figure 1: An example of interferon (IFN)-γ, interleukin (IL)-4 and granzyme B-secreting T cell responses against SARS-CoV-2 Spike protein measured by FluoroSpot at day 7 after the third dose vaccination........29
Supplementary Figure 2: An example of interferon (IFN)-γ, interleukin (IL)-4 and granzyme B-secreting T cell responses against SARS-CoV-2 Spike protein measured by FluoroSpot at day 14 after the third dose vaccination........31
Supplementary Figure 3: Correlations between neutralising antibody against wild-type SARS-CoV-2 and the prime-boost interval........33
Supplementary Figure 4: Correlations between neutralising antibody against the delta variant of SARS-CoV-2 and the prime-boost interval........35
Supplementary Figure 5: Correlations between neutralising antibody against the omicron variant of SARS-CoV-2 and the prime-boost interval........37
Supplementary Figure 6: Subgroup analysis of neutralising antibodies against SARS-CoV-2 at day 7 after the third dose vaccination stratified by the prime-boost interval........39
Supplementary Figure 7: Subgroup analysis of neutralising antibodies against SARS-CoV-2 at day 14 after the third dose vaccination stratified by the prime-boost interval........41
Supplementary Figure 8: Subgroup analysis of neutralising antibodies against SARS-CoV-2 at day 28 after the third dose vaccination stratified by the prime-boost interval........43
Supplementary Figure 9: Subgroup analysis of neutralising antibodies against SARS-CoV-2 at day 90 after the third dose vaccination stratified by the prime-boost interval........45
Supplementary Figure 10: Subgroup analysis of neutralising antibodies against SARS-CoV-2 at day 7 after the third dose vaccination stratified by sex........47
Supplementary Figure 11: Subgroup analysis of neutralising antibodies against SARS-CoV-2 at day 14 after the third dose vaccination stratified by sex........49

Supplementary Figure 12: Subgroup analysis of neutralising antibodies against SARS-CoV-2 at day 28 after the third dose vaccination stratified by sex............51
Supplementary Figure 13: Subgroup analysis of neutralising antibodies against SARS-CoV-2 at day 90 after the third dose vaccination stratified by sex............53
Supplementary Figure 14: Subgroup analysis of T cell responses against wild-type of SARS-CoV-2 Spike protein at day 7 after the third dose vaccination stratified by the prime-boost interval...55
Supplementary Figure 15: Subgroup analysis of T cell responses against wild-type of SARS-CoV-2 Spike protein at day 14 after the third dose vaccination stratified by the prime-boost interval...57
Supplementary Figure 16: Subgroup analysis of T cell responses against wild-type of SARS-CoV-2 Spike protein at day 28 after the third dose vaccination stratified by the prime-boost interval...59
Supplementary Figure 17: Subgroup analysis of T cell responses against wild-type of SARS-CoV-2 Spike protein at day 7 after the third dose vaccination stratified by sex.. 61
Supplementary Figure 18: Subgroup analysis of T cell responses against wild type of SARS-CoV-2 Spike protein at day 14 after the third dose vaccination stratified by sex.. 63
Supplementary Figure 19: Subgroup analysis of T cell responses against wild-type of SARS-CoV-2 Spike protein at day 28 after the third dose vaccination stratified by sex.. 65
Supplementary Figure 20: Subgroup analysis of T cell responses against omicron variant of SARS-CoV-2 Spike protein at day 7 after the third dose vaccination stratified by sex.. 67
Supplementary Figure 21: Subgroup analysis of T cell responses against omicron variant of SARS-CoV-2 Spike protein at day 14 after the third dose vaccination stratified by sex.. 69
Supplementary Figure 22: Subgroup analysis of T cell responses against omicron variant of SARS-CoV-2 at day 28 after the third dose vaccination stratified by sex.. 71
Supplementary Figure 23: Correlations between neutralising antibodies against SARS-CoV-2 after the third dose vaccination by booster schedules................... 73

Supplementary Figure 24: Correlations between T cell responses against SARS-CoV-2 after the third dose vaccination by booster schedules.............................. 75
Supplementary Figure 25: Correlations between neutralising antibodies and T cell responses against wild-type SARS-CoV-2 after the third dose vaccination by booster schedules...77
Supplementary Figure 26: Correlations between neutralising antibodies against wild-type SARS-CoV-2 and T cell responses against omicron variant of SARS-CoV-2 after the third dose vaccination by booster schedules.............................. 79
Supplementary Figure 27: SARS-CoV-2 evolution and vaccine design..............81
Supplementary Figure 28: Characteristics of neutralisation, T cell response and reactogenicity profiles of the four COVID-19 vaccines and placebo in this trial. 83

Supplementary Tables

Supplementary Table 1: Kinetics of neutralising antibody measured by competitive inhibition method across booster schedules.

Time points	ChAdTS-S (n=48)			RQ3013 (n=47)			ZR202-CoV (n=47)			CoronaVac (n=46)			Placebo (n=46)		
	GMT (95%CI)	GMFR (95% CI)	*P*	GMT (95%CI)	GMFR (95% CI)	*P*	GMT (95%CI)	GMFR (95% CI)	*P*	GMT (95%CI)	GMFR (95% CI)	*P*	GMT (95%CI)	GMFR (95% CI)	*P*
**Neutralisation antibody measured by competitive inhibition method**															
Day 0	131.9	Ref	Ref	131.3	Ref	Ref	160.1	Ref	Ref	129.9	Ref	Ref	203.2	Ref	Ref
	(99.9-174.2)	-	-	(100.4-171.7)	-	-	(114.8-223.2)	-	-	(100.7-167.5)	-	-	(155.6-265.4)	-	-
Day 1	128.8	1.0	0.7332	130.2	1.0	0.7804	155.5	1.0	0.2127	129.9	1.0	0.9975	192.0	0.9	0.0696
	(100.2-165.6)	(0.8-1.1)		(100.8-168.2)	(0.9-1.1)		(113.2-213.5)	(0.9-1.0)		(101.3-166.6)	(1.0-1.0)		(146.6-251.5)	(0.9-1)	
Day 4	146.0	1.1	0.1968	173.3	1.3	0.0001	191.5	1.2	0.0006	142.2	1.1	0.0061	201.1	1.0	0.7284
	(116.2-183.4)	(0.9-1.3)		(132.9-226.1)	(1.2-1.5)		(143.4-255.8)	(1.1-1.3)		(111.9-180.8)	(1.0-1.2)		(156.1-259.1)	(0.9-1.1)	
Day 7	5165.1	39.2	<0.0001	20445.4	155.7	<0.0001	9881.5	61.7	<0.0001	1223.6	9.4	<0.0001	181.1	0.9	<0.0001
	(4030.4-6619.1)	(28.4-54.1)		(14421.5-28985.4)	(111.9-216.8)		(7060.2-13830.2)	(45.8-83.1)		(945.9-1582.9)	(7.1-12.5)		(139.8-234.5)	(0.8-0.9)	
Day 14	19512.9	147.9	<0.0001	90384.3	704.2	<0.0001	51995.0	324.8	<0.0001	4588.2	35.3	<0.0001	165.2	0.8	<0.0001
	(15498.4-24567.1)	(108.1-202.5)		(71218.5-114707.9)	(521.6-950.6)		(41583.4-65013.4)	(234.5-449.9)		(3558.8-5915.5)	(25.7-48.6)		(127.2-214.7)	(0.8-0.9)	
Day 28	22431.3	170.1	<0.0001	58004.8	441.8	<0.0001	51833.7	323.8	<0.0001	4206.5	32.4	<0.0001	159.7	0.8	<0.0001
	(17951.2-28029.5)	(124.0-233.2)		(45870.1-73349.6)	(330.6-590.4)		(41894.3-64131.1)	(234.3-447.4)		(3297.8-5365.5)	(23.7-44.3)		(121.1-210.8)	(0.7-0.9)	
Day90	957.8	7.0	<0.0001	1082.1	8.2	<0.0001	1180.6	7.2	<0.0001	268.8	2.1	0.0084	5.2	0.0	<0.0001
	(716.4-1280.5)	(5.0-9.8)		(812.5-1441.3)	(5.7-11.8)		(858.7-1623.2)	(4.6-11.3)		(158.4-456.2)	(1.2-3.5)		(3.4-7.9)	(0.0-0.0)	

Note: GMT, geometric mean titre; GMFR, geometric mean fold rise.

Supplementary Table 2: Kinetics of neutralising antibodies against the wild-type, delta and omicron variants of live SARS-CoV-2 across booster schedules.

Time points	ChAdTS-S (n=-48)			RQ3013 (n=47)			ZR202-CoV (n=47)			CoronaVac (n=46)			Placebo (n=46)		
	GMT (95% CI)	GMFR (95% CI)	*P*	GMT (95% CI)	GMFR (95% CI)	*P*	GMT (95% CI)	GMFR (95% CI)	*P*	GMT (95% CI)	GMFR (95% CI)	*P*	GMT (95% CI)	GMFR (95% CI)	*P*
**Neutralising antibody against the wild-type of live SARS-CoV-2**															
Day 0	4.8	Ref	Ref	4.6	Ref	Ref	5.1	Ref	Ref	4.4	Ref	Ref	5.4	Ref	Ref
	(4.3-5.3)	-	-	(4.1-5.1)	-	-	(4.4-5.9)	-	-	(4.0-4.8)	-	-	(4.7-6.3)	-	-
Day 7	86.0	18.0	<0.0001	335.7	73.3	<0.0001	178.8	35.1	<0.0001	29.6	6.7	<0.0001	5.4	1.0	0.8425
	(67.6-109.4)	(14.2-2.9)		(254.8-442.2)	(54.9-97.9)		(133.5-239.5)	(26.5-46.5)		(23.1-37.8)	(5.2-8.6)		(4.5-6.3)	(0.9-1.1)	
Day 14	289.6	60.72	<0.0001	950.1	210.0	<0.0001	635.2	124.8	<0.0001	75.4	17.1	<0.0001	5.4	1.0	0.8960
	(232.2-361.2)	(47.8-77.2)		(785.4-1149.3)	(172.0-256.4)		(524.0-770.0)	(102.4-152.0)		(61.4-92.5)	(13.6-21.4)		(4.5-6.4)	(0.9-1.1)	
Day 28	326.3	68.4	<0.0001	709.4	154.9	<0.0001	654.3	128.5	<0.0001	63.1	14.3	<0.0001	5.5	1.0	0.9439
	(264.8-402.0)	(54.3-86.2)		(593.4-848.2)	(128.1-187.4)		(543.6-787.5)	(106.5-155.2)		(52.6-75.7)	(11.6-17.5)		(4.6-6.5)	(0.9-1.2)	
Day 90	75.8	15.7	<0.0001	124.3	27.1	<0.0001	174.1	35.0	<0.0001	18.5	4.2	<0.0001	4.1	0.8	0.0007
	(61.9-92.8)	(12.4-19.9)		(96.1-160.7)	(20.7-35.3)		(134.4-225.5)	(27.7-44.4)		(14.9-22.9)	(3.2-5.3)		(3.9-4.3)	(0.6-0.9)	
**Neutralising antibody against the delta variant of live SARS-CoV-2**															
Day 0	4.4	Ref	Ref	4.5	Ref	Ref	4.6	Ref	Ref	4.2	Ref	Ref	4.5	Ref	Ref
	(3.7-5.3)	-	-	(3.7-5.4)	-	-	(3.9-5.3)	-	-	(4.0-4.4)	-	-	(4.1-5.0)	-	-
Day 7	59.5	13.4	<0.0001	279.5	62.5	<0.0001	126.1	27.7	<0.0001	15.5	3.7	<0.0001	4.5	1.0	0.6597
	(45.2-78.4)	(9.8-18.4)		(205.1-381)	(43.1-90.6)		(91.2-174.2)	(20.7-37.1)		(11.6-20.7)	(2.8-5.0)		(4.0-4.9)	(0.9-1.1)	
Day 14	226.0	51.1	<0.0001	796.9	177.7	<0.0001	491.9	108.1	<0.0001	54.3	13.0	<0.0001	4.3	1.0	0.3683
	(187.2-273)	(39.7-65.7)		(667.7-951.1)	(132.9-237.6)		(411.6-587.9)	(88.7-131.7)		(42.4-69.4)	(10.1-16.6)		(3.9-4.8)	(0.9-1.1)	
Day 28	250.01	56.5	<0.0001	589.3	131.7	<0.0001	484.2	106.4	<0.0001	52.7	12.6	<0.0001	4.5	1.0	1.0000
	(205.3-304.6)	(44.3-72.1)		(483.4-718.3)	(96.7-179.5)		(397.8-589.3)	(86.9-130.3)		(41.4-67.1)	(9.9-16.1)		(4.1-5.0)	(0.9-1.1)	
Day 90	122.1	27.5	<0.0001	217.0	48.3	<0.0001	294.6	64.5	<0.0001	20.0	4.8	<0.0001	4.2	0.9	0.1640
	(98.1-152.0)	(21.5-35.3)		(159.6-295.2)	(32.5-71.8)		(229-378.9)	(49.2-84.6)		(14.8-27.2)	(3.5-6.5)		(4.0-4.4)	(0.9-1.0)	
**Neutralising antibody against the omicron variant of live SARS-CoV-2**															

Day 0	4.0	Ref	Ref	4.0	Ref	Ref	4.0	Ref	Ref	4.0	Ref	Ref	4.0	Ref	Ref
	(4.0-4.0)	-	-	(4.0-4.0)	-	-	(4.0-4.0)	-	-	(4.0-4.0)	-	-	(4.0-4.0)	-	-
Day 7	10.4	2.6	<0.0001	59.9	15.0	<0.0001	21.6	5.4	<0.0001	4.5	1.1	0.0505	4.0	1.0	-
	(8.0-13.7)	(2.0-3.4)		(42.9-83.5)	(10.7-20.9)		(15.2-30.6)	(3.8-7.7)		(4.0-5.0)	(1.0-1.2)		(4.0-4.0)	(1.0-1.0)	
Day 14	40.5	10.1	<0.0001	247.0	61.7	<0.0001	103.8	26.0	<0.0001	8.1	2.0	<0.0001	4.0	1.0	-
	(31.2-52.5)	(7.8-13.1)		(194.1-314.3)	(48.5-78.6)		(84-128.3)	(21.0-32.1)		(6.1-10.7)	(1.5-2.7)		(4.0-4.0)	(1.0-1.0)	
Day 28	35.2	8.8	<0.0001	151.7	37.9	<0.0001	93.5	23.4	<0.0001	6.4	1.6	0.0004	4.0	1.0	-
	(26.9-46.1)	(6.7-11.5)		(117.1-196.6)	(29.3-49.1)		(76.3-114.6)	(19.1-28.6)		(5.0- 8.2)	(1.3-2.0)		(4.0-4.0)	(1.0-1.0)	
Day 90	12.4	3.1	<0.0001	37.6	9.4	<0.0001	45.2	11.3	<0.0001	4.7	1.2	0.0197	4.0	1.0	-
	(9.6-16.0)	(2.4-4,0)		(26.9-52.6)	(6.7-13.2)		(33.3-61.3)	(8.3-15.3)		(4.1-5.5)	(1.0-1.4)		(4.0-4.0)	(1.0-1.0)	

Note: GMT, geometric mean titre; GMFR, geometric mean fold rise.

Supplementary Table 3: Geometric mean titre and geometric mean ratio of humoral responses at day 14 after the third dose vaccination analyzed in the per-protocol population.

	ChAdTS-S (n=48)	RQ3013 (n=46)	ZR202-CoV (n=47)	CoronaVac (n=45)	Placebo (n=46)
**Neutralising antibody against the wild-type of live SARS-CoV-2**					
GMT	289.6	948.5	635.2	75.7	5.4
	(232.2-361.2)	(780.6-1152.4)	(524.0-770.0)	(61.4-93.3)	(4.5-6.4)
GMR1	56.8	190.3	120.8	15.2	Ref
	(43.5-74.2)	(145.0-249.7)	(92.5-157.8)	(11.5-19.9)	-
GMR2	3.7	12.5	8.0	Ref	-
	(2.9-4.9)	(9.6-16.5)	(6.1-10.4)	-	-
*P*1	<0.0001	<0.0001	<0.0001	<0.0001	Ref
*P*2	<0.0001	<0.0001	<0.0001	Ref	-
**Neutralising antibody against the delta variant of live SARS-CoV-2**					
GMT	226.0	792.5	491.9	54.9	4.3
	(187.2-273.0)	(661.5-949.3)	(411.6-587.9)	(42.7-70.6)	(3.9-4.8)
GMR1	52.8	183.9	113.9	12.7	Ref
	(41.0-68.2)	(142.1-238.2)	(88.2-147.1)	(9.8-16.5)	-
GMR2	4.2	14.5	9.0	Ref	-
	(3.2-5.4)	(11.1-18.8)	(6.9-11.6)	-	-
*P*1	<0.0001	<0.0001	<0.0001	<0.0001	Ref
*P*2	<0.0001	<0.0001	<0.0001	Ref	-
**Neutralising antibody against the omicron variant of live SARS-CoV-2**					
GMT	40.5	240.9	103.8	8.2	4.0
	(31.2-52.5)	(189.3-306.6)	(84.0-128.3)	(6.2-10.9)	(4.0-4.0)
GMR1	10.3	60.9	26.0	2.0	Ref
	(7.6-14.0)	(44.6-83)	(19.1-35.3)	(1.5-2.8)	-
GMR2	5.1	30.0	12.8	Ref	-
	(3.7-6.9)	(21.9-41.0)	(9.4-17.4)	-	-
*P*1	<0.0001	<0.0001	<0.0001	<0.0001	Ref
*P*2	<0.0001	<0.0001	<0.0001	Ref	-
**Neutralising antibody measured by competitive inhibition method**					
GMT	19512.9	88322.3	51995.0	4588.2	165.2
	(15498.4-24567.1)	(69536.2-112183.6)	(41583.4-65013.4)	(3538.0-5950.3)	(127.2-214.7)
GMR1	138.7	624.6	346.0	33.0	Ref
	(102.1-188.4)	(457.6-852.5)	(254.9-469.6)	(24.2-45.1)	-
GMR2	4.2	18.9	10.5	Ref	-
	(3.1-5.7)	(13.8-25.8)	(7.7-14.2)	-	-
*P*1	<0.0001	<0.0001	<0.0001	<0.0001	Ref
*P*2	<0.0001	<0.0001	<0.0001	Ref	-

Note: Geometric mean ratio (GMR) was calculated using linear regression model by adjusting for the baseline neutralising antibody levels at day 0 before the booster dose vaccination, the first and second dose interval, and the second and third dose interval. GMR1 and p1 were calculated by regarding placebo as control, while GMR2 and p2 were calculated by regarding CoronaVac as control.

Supplementary Table 4: Kinetics of T cell responses against the wild-type of SARS-CoV-2 Spike protein across booster schedules.

Time points	ChAdTS-S		RQ3013		ZR202-CoV		CoronaVac		Placebo	
	Median (IQR)	*P*	Median (IQR)	*P*	Median (IQR)	*P*	Median (IQR)	*P*	Median (IQR)	*P*
**IFN-γ^{+} SFC per million PBMCs against wild-type of SARS-CoV-2 Spike protein**										
Day 0	282.0(110.0,666.0)	Ref	248.0(84.0,512.0)	Ref	232.0(68.0,384.0)	Ref	200.0(99.0,639.0)	Ref	318.0(133.0,619.0)	Ref
Day 7	1212.0(593.0,1483.0)	<0.0001	2404.0(1512.0,3240.0)	<0.0001	1008.0(516.0,1546.0)	<0.0001	336.0(168.0,852.0)	0.5068	292.0(104.0,856.0)	0.6454
Day 14	904.0(636.0,1288.0)	0.0035	1272.0(816.0,2552.0)	<0.0001	1048.0(426.0,1586.0)	<0.0001	472.0(212.0,956.0)	0.1132	382.0(171.0,891.0)	0.3574
Day 28	366.0(234.0,771.0)	0.6011	628.0(320.0,1052.0)	0.0002	416.0(237.0,795.0)	0.0016	252.0(102.0,558.0)	0.6434	452.0(112.0,760.0)	0.4948
**Granzyme B^{+} SFC per million PBMCs against wild-type of SARS-CoV-2 Spike protein**										
Day 0	268.0(140.0,468.0)	Ref	296.0(86.0,700.0)	Ref	256.0(68.0,516.0)	Ref	210.0(103.0,799.0)	Ref	306.0(159.0,628.0)	Ref
Day 7	994.0(461.0,1359.0)	0.0003	1662.0(1029.0,2583.0)	<0.0001	808.0(340.0,1348.0)	0.0008	416.0(168.0,736.0)	0.4217	432.0(142.0,1036.0)	0.1416
Day 14	1092.0(608.0,1472.0)	0.0018	1348.0(586.0,1844.0)	<0.0001	988.0(424.0,1772.0)	0.0001	496.0(160.0,1220.0)	0.0232	328.0(139.0,938.0)	0.0846
Day 28	354.0(111.0,538.0)	0.4455	548.0(208.0,948.0)	0.0170	600.0(240.0,784.0)	0.0573	210.0(65.0,464.0)	0.2904	372.0(128.0,772.0)	0.4851
**IL-4^{+} SFC per million PBMCs against wild-type of SARS-CoV-2 Spike protein**										
Day 0	0.0(0.0,46.0)	Ref	0.0(0.0,56.0)	Ref	0.0(0.0,44.0)	Ref	0.0(0.0,37.0)	Ref	20.0(0.0,44.0)	Ref
Day 7	74.0(33.0,164.0)	<0.0001	392.0(124.0,631.0)	<0.0001	66.0(23.0,175.0)	<0.0001	20.0(0.0,58.0)	0.4458	0.0(0.0,44.0)	0.7062
Day 14	56.0(0.0,118.0)	<0.0001	168.0(76.0,344.0)	<0.0001	76.0(24.0,148.0)	<0.0001	56.0(20.0,100.0)	0.0045	0.0(0.0,44.0)	1.0000
Day 28	22.0(0.0,58.0)	0.6011	80.0(22.0,139.0)	0.0030	36.0(0.0,54.0)	0.7701	20.0(0.0,46.0)	0.3650	10.0(0.0,41.0)	0.6956

Note: IQR, interquartile range; PBMCs, peripheral blood mononuclear cells; SFC, spot forming cell.

Supplementary Table 5: Kinetics of T cell responses against the omicron variant of SARS-CoV-2 Spike protein across booster schedules.

Time points	ChAdTS-S		RQ3013		ZR202-CoV		CoronaVac		Placebo	
	Median (IQR)	*P*	Median (IQR)	*P*	Median (IQR)	*P*	Median (IQR)	*P*	Median (IQR)	*P*
**IFN-γ⁺ SFC per million PBMCs against omicron variant of SARS-CoV-2 Spike protein**										
Day 0	1008.0(452.0,1434.0)	Ref	346.0(248.0,653.0)	Ref	528.0(220.0,1272.0)	Ref	360.0(164.0,1186.0)	Ref	640.0(270.0,1150.0)	Ref
Day 7	1376.0(889.0,1925.0)	0.0094	2496.0(1112.0,2884.0)	0.0001	880.0(402.0,1550.0)	0.0046	582.0(194.0,1165.0)	0.2633	874.0(316.0,1228.0)	0.1329
Day 14	1268.0(908.0,1568.0)	0.2122	1770.0(795.0,2287.0)	0.0001	1390.0(637.0,2116.0)	0.0026	896.0(390.0,1681.0)	0.0210	812.0(366.0,1162.0)	0.7819
Day 28	1474.0(710.0,2019.0)	0.0353	1024.0(330.0,1246.0)	0.0248	1102.0(555.0,2077.0)	0.0210	570.0(431.0,1016.0)	0.1294	758.0(209.0,947.0)	0.5693
**Granzyme B⁺ SFC per million PBMCs against omicron variant of SARS-CoV-2 Spike protein**										
Day 0	944.0(471.0,1659.0)	Ref	756.0(378.0,1065.0)	Ref	590.0(341.0,1060.0)	Ref	568.0(204.0,832.0)	Ref	708.0(544.0,1840.0)	Ref
Day 7	1392.0(1000.0,1850.0)	0.2633	2134.0(587.0,2273.0)	0.0010	1048.0(472.0,1320.0)	0.0176	668.0(356.0,1144.0)	0.4954	1314.0(542.0,1657.0)	0.4212
Day 14	1484.0(992.0,2192.0)	0.0591	1730.0(749.0,2494.0)	0.0020	1508.0(588.0,2044.0)	0.0067	1016.0(632.0,1858.0)	0.1078	1028.0(523.0,1293.0)	0.6848
Day 28	916.0(704.0,1464.0)	1.0000	1048.0(488.0,1336.0)	0.0771	972.0(356.0,1760.0)	0.1197	314.0(240.0,713.0)	0.4800	846.0(205.0,1257.0)	0.3223
**IL-4⁺ SFC per million PBMCs against omicron variant of SARS-CoV-2 Spike protein**										
Day 0	24.0(0.0,36.0)	Ref	0.0(0.0,24.0)	Ref	10.0(0.0,59.0)	Ref	0.0(0.0,48.0)	Ref	0.0(0.0,28.0)	Ref
Day 7	92.0(40.0,154.0)	0.0006	220.0(144.0,508.0)	0.0001	56.0(15.0,202.0)	0.0035	24.0(0.0,40.0)	0.7835	24.0(0.0,48.0)	0.3261
Day 14	44.0(24.0,122.0)	0.0074	92.0(68.0,148.0)	0.0001	38.0(5.0,86.0)	0.0056	44.0(28.0,68.0)	0.0156	0.0(0.0,36.0)	0.4765
Day 28	38.0(15.0,71.0)	0.0096	32.0(0.0,64.0)	0.0071	22.0(0.0,62.0)	0.9645	0.0(0.0,36.0)	0.3297	0.0(0.0,32.0)	0.7786

Note: IQR, interquartile range; PBMCs, peripheral blood mononuclear cells; SFC, spot forming cell. T cell response against omicron variant of SARS-CoV-2 Spike protein was determined in samples from a random subset of the 234 participants (n=23 for ChAdTS-S, n=21 for RQ3013, n=22 for ZR202-CoV, n=21 for CoronaVac, and n=21 for placebo).

Supplementary Table 6: T cell responses against the wild-type and omicron variant of SARS-CoV-2 Spike protein across booster schedules.

Time points	ChAdTS-S			RQ3013			ZR202-CoV			CoronaVac			Placebo		
	wild-type (n=234)	omicron (n=108)	*P*	wild-type (n=234)	omicron (n=108)	*P*	wild-type (n=234)	omicron (n=108)	*P*	wild-type (n=234)	omicron (n=108)	*P*	wild-type (n=234)	omicron (n=108)	*P*
**IFN-γ^+ SFC per million PBMCs against wild-type or omicron variant of SARS-CoV-2 Spike protein**															
Day 0	282.0 (110.0,666.0)	1008.0 (452.0,1434.0)	0.0009	248.0 (84.0,512.0)	346.0 (248.0,653.0)	0.0491	232.0 (68.0,384.0)	528.0 (220.0,1272.0)	0.0096	200.0 (99.0,639.0)	360.0 (164.0,1186.0)	0.3550	318.0 (133.0,619.0)	640.0 (270.0,1150.0)	0.0783
Day 7	1212.0 (593.0,1483.0)	1376.0 (889.0,1925.0)	0.1007	2404.0 (1512.0,3240.0)	2496.0 (1112.0,2884.0)	0.5420	1008.0 (516.0,1546.0)	880.0 (402.0,1550.0)	0.9043	336.0 (168.0,852.0)	582.0 (194.0,1165.0)	0.3652	292.0 (104.0,856.0)	874.0 (316.0,1228.0)	0.0221
Day 14	904.0 (636.0,1288.0)	1268.0 (908.0,1568.0)	0.0411	1272.0 (816.0,2552.0)	1770.0 (795.0,2287.0)	0.7939	1048.0 (426.0,1586.0)	1390.0 (637.0,2116.0)	0.1979	472.0 (212.0,956.0)	896.0 (390.0,1681.0)	0.0455	382.0 (171.0,891.0)	812.0 (366.0,1162.0)	0.0921
Day 28	366.0 (234.0,771.0)	1474.0 (710.0,2019.0)	0.0001	628.0 (320.0,1052.0)	1024.0 (330.0,1246.0)	0.5197	416.0 (237.0,795.0)	1102.0 (555.0,2077.0)	0.0516	252.0 (102.0,558.0)	570.0 (431.0,1016.0)	0.0198	452.0 (112.0,760.0)	758.0 (209.0,947.0)	0.6265
**Granzyme B^+ SFC per million PBMCs against wild-type or omicron variant of SARS-CoV-2 Spike protein**															
Day 0	268.0 (140.0,468.0)	944.0 (471.0,1659.0)	0.0005	296.0 (86.0,700.0)	756.0 (378.0,1065.0)	0.0102	256.0 (68.0,516.0)	590.0 (341.0,1060.0)	0.0110	210.0 (103.0,799.0)	568.0 (204.0,832.0)	0.1608	306.0 (159.0,628.0)	708.0 (544.0,1840.0)	0.0019
Day 7	994.0 (461.0,1359.0)	1392.0 (1000.0,1850.0)	0.0213	1662.0 (1029.0,2583.0)	2134.0 (587.0,2273.0)	0.8599	808.0 (340.0,1348.0)	1048.0 (472.0,1320.0)	0.3954	416.0 (168.0,736.0)	668.0 (356.0,1144.0)	0.1426	432.0 (142.0,1036.0)	1314.0 (542.0,1657.0)	0.0067
Day 14	1092.0 (608.0,1472.0)	1484.0 (992.0,2192.0)	0.0759	1348.0 (586.0,1844.0)	1730.0 (749.0,2494.0)	0.3766	988.0 (424.0,1772.0)	1508.0 (588.0,2044.0)	0.3373	496.0 (160.0,1220.0)	1016.0 (632.0,1858.0)	0.0525	328.0 (139.0,938.0)	1028.0 (523.0,1293.0)	0.0158
Day 28	354.0 (111.0,538.0)	916.0 (704.0,1464.0)	0.0009	548.0 (208.0,948.0)	1048.0 (488.0,1336.0)	0.0469	600.0 (240.0,784.0)	972.0 (356.0,1760.0)	0.1531	210.0 (65.0,464.0)	314.0 (240.0,713.0)	0.0985	372.0 (128.0,772.0)	846.0 (205.0,1257.0)	0.1476
**IL-4^+ SFC per million PBMCs against wild-type or omicron variant of SARS-CoV-2 Spike protein**															
Day 0	0.0 (0.0,46.0)	24.0 (0.0,36.0)	0.5166	0.0 (0.0,56.0)	0.0 (0.0,24.0)	0.2952	0.0 (0.0,44.0)	10.0 (0.0,59.0)	0.7163	0.0 (0.0,37.0)	0.0 (0.0,48.0)	0.5738	20.0 (0.0,44.0)	0.0 (0.0,28.0)	0.1712
Day 7	74.0 (33.0,164.0)	92.0 (40.0,154.0)	0.9796	392.0 (124.0,631.0)	220.0 (144.0,508.0)	0.2838	66.0 (23.0,175.0)	56.0 (15.0,202.0)	0.7544	20.0 (0.0,58.0)	24.0 (0.0,40.0)	0.9644	0.0 (0.0,44.0)	24.0 (0.0,48.0)	0.7587
Day 14	56.0 (0.0,118.0)	44.0 (24.0,122.0)	1.0000	168.0 (76.0,344.0)	92.0 (68.0,148.0)	0.0252	76.0 (24.0,148.0)	38.0 (5.0,86.0)	0.2056	56.0 (20.0,100.0)	44.0 (28.0,68.0)	0.6300	0.0 (0.0,44.0)	0.0 (0.0,36.0)	0.6117
Day 28	22.0 (0.0,58.0)	38.0 (15.0,71.0)	0.2267	80.0 (22.0,139.0)	32.0 (0.0,64.0)	0.1495	36.0 (0.0,54.0)	22.0 (0.0,62.0)	0.7475	20.0 (0.0,46.0)	0.0 (0.0,36.0)	0.6846	10.0 (0.0,41.0)	0.0 (0.0,32.0)	0.3894

Note: IQR, interquartile range; PBMCs, peripheral blood mononuclear cells; SFC, spot forming cell. T cell responses against omicron variant of SARS-CoV-2 Spike protein were determined in samples from a random subset of the 234 participants (n=23 for ChAdTS-S, n=21 for RQ3013, n=22 for ZR202-CoV, n=21 for CoronaVac, and n=21 for placebo).

Supplementary Table 7 : Comparison of T cell responses against the wild-type of SARS-CoV-2 Spike protein across booster schedules.

Time points	ChAdTS-S	RQ3013	ZR202-CoV	CoronaVac	Placebo
	Median (IQR)	Median (IQR)	Median (IQR)	Median (IQR)	Median (IQR)
**IFN-γ^+ SFC per million PBMCs against wild-type of SARS-CoV-2 Spike protein**					
Day 0	282.0 (110.0,666.0)	248.0 (84.0,512.0)	232.0 (68.0,384.0)	200.0 (99.0,639.0)	318.0 (133.0,619.0)
*P*1	0.9251	0.2307	0.1536	0.6194	Ref
*P*2	0.6196	0.5835	0.5835	Ref	-
Day 7	1212.0 (593.0,1483.0)	2404.0 (1512.0,3240.0)	1008.0 (516.0,1546.0)	336.0 (168.0,852.0)	292.0 (104.0,856.0)
*P*1	<0.0001	<0.0001	<0.0001	0.5251	Ref
*P*2	0.0001	<0.0001	0.0009	Ref	
Day 14	904.0 (636.0,1288.0)	1272.0 (816.0,2552.0)	1048.0 (426.0,1586.0)	472.0 (212.0,956.0)	382.0 (171.0,891.0)
*P*1	0.0014	<0.0001	0.0016	0.4948	Ref
*P*2	0.0099	<0.0001	0.0073	Ref	
Day 28	366.0 (234.0,771.0)	628.0 (320.0,1052.0)	416.0 (237.0,795.0)	252.0 (102.0,558.0)	452.0 (112.0,760.0)
*P*1	0.7074	0.1750	0.6491	0.3655	Ref
*P*2	0.1280	0.0089	0.0847	Ref	
**Granzyme B$^+$ SFC per million PBMCs against wild-type of SARS-CoV-2 Spike protein**					
Day 0	268.0 (140.0,468.0)	296.0 (86.0,700.0)	256.0 (68.0,516.0)	210.0 (103.0,799.0)	306.0 (159.0,628.0)
*P*1	0.8404	0.7251	0.5137	0.4749	Ref
*P*2	0.4833	0.7743	0.9959	Ref	-
Day 7	994.0 (461.0,1359.0)	1662.0 (1029.0,2583.0)	808.0 (340.0,1348.0)	416.0 (168.0,736.0)	432.0 (142.0,1036.0)
*P*1	0.0048	<0.0001	0.0539	0.9077	Ref
*P*2	0.0024	<0.0001	0.0320	Ref	-
Day 14	1092.0 (608.0,1472.0)	1348.0 (586.0,1844.0)	988.0 (424.0,1772.0)	496.0 (160.0,1220.0)	328.0 (139.0,938.0)
*P*1	0.0035	0.0001	0.0070	0.3235	Ref
*P*2	0.0361	0.0023	0.0523	Ref	-
Day 28	354.0 (111.0,538.0)	548.0 (208.0,948.0)	600.0 (240.0,784.0)	210.0 (65.0,464.0)	372.0 (128.0,772.0)
*P*1	0.6880	0.3135	0.1757	0.2189	Ref
*P*2	0.2597	0.0185	0.0048	Ref	-
**IL-4$^+$ SFC per million PBMCs against wild-type of SARS-CoV-2 Spike protein**					
Day 0	0.0 (0.0,46.0)	0.0 (0.0,56.0)	0.0 (0.0,44.0)	0.0 (0.0,37.0)	20.0 (0.0,44.0)

*P*1	0.5937	0.8251	0.5577	0.5530	Ref
*P*2	1.0000	0.8528	0.9819	Ref	-
Day 7	74.0 (33.0,164.0)	392.0 (124.0,631.0)	66.0 (23.0,175.0)	20.0 (0.0,58.0)	0.0 (0.0,44.0)
*P*1	<0.0001	<0.0001	0.0002	0.5586	Ref
*P*2	0.0001	<0.0001	0.0006	Ref	-
Day 14	56.0 (0.0,118.0)	168.0 (76.0,344.0)	76.0 (24.0,148.0)	56.0 (20.0,100.0)	0.0 (0.0,44.0)
*P*1	0.0047	<0.0001	0.0003	0.0074	Ref
*P*2	0.7045	<0.0001	0.1799	Ref	-
Day 28	22.0 (0.0,58.0)	80.0 (22.0,139.0)	36.0 (0.0,54.0)	20.0 (0.0,46.0)	10.0 (0.0,41.0)
*P*1	0.5700	0.0009	0.1841	0.6952	Ref
*P*2	0.8760	0.0068	0.3985	Ref	-

Note: IQR, interquartile range; PBMCs, peripheral blood mononuclear cells; SFC, spot forming cell.

Supplementary Table 8 : Comparison of T cell responses against the omicron variant of SARS-CoV-2 Spike protein across booster schedules.

Time points	ChAdTS-S	RQ3013	ZR202-CoV	CoronaVac	Placebo
	Median (IQR)	Median (IQR)	Median (IQR)	Median (IQR)	Median (IQR)
**IFN-γ⁺ SFC per million PBMCs against omicron variant of SARS-CoV-2**					
Day 0	1008.0 (452.0,1434.0)	346.0 (248.0,653.0)	528.0 (220.0,1272.0)	360.0 (164.0,1186.0)	640.0 (270.0,1150.0)
*P*1	0.1263	0.3837	0.9676	0.5135	Ref
*P*2	0.0473	0.9883	0.4138	Ref	-
Day 7	1376.0 (889.0,1925.0)	2496.0 (1112.0,2884.0)	880.0 (402.0,1550.0)	582.0 (194.0,1165.0)	874.0 (316.0,1228.0)
*P*1	0.0224	0.0011	0.4619	0.4864	Ref
*P*2	0.0025	0.0002	0.1516	Ref	-
Day 14	1268.0 (908.0,1568.0)	1770.0 (795.0,2287.0)	1390.0 (637.0,2116.0)	896.0 (390.0,1681.0)	812.0 (366.0,1162.0)
*P*1	0.0154	0.0106	0.0360	0.2983	Ref
*P*2	0.4079	0.1186	0.2681	Ref	-
Day 28	1474.0 (710.0,2019.0)	1024.0 (330.0,1246.0)	1102.0 (555.0,2077.0)	570.0 (431.0,1016.0)	758.0 (209.0,947.0)
*P*1	0.0108	0.1432	0.0997	0.6297	Ref
*P*2	0.0031	0.6833	0.2189	Ref	-
**Granzyme B⁺ SFC per million PBMCs against omicron variant of SARS-CoV-2**					
Day 0	944.0 (471.0,1659.0)	756.0 (378.0,1065.0)	590.0 (341.0,1060.0)	568.0 (204.0,832.0)	708.0 (544.0,1840.0)
*P*1	0.7413	0.4193	0.2406	0.1222	Ref
*P*2	0.0527	0.2346	0.5833	Ref	-
Day 7	1392.0 (1000.0,1850.0)	2134.0 (587.0,2273.0)	1048.0 (472.0,1320.0)	668.0 (356.0,1144.0)	1314.0 (542.0,1657.0)
*P*1	0.3046	0.1125	0.4426	0.1547	Ref
*P*2	0.0094	0.0091	0.2688	Ref	-
Day 14	1484.0 (992.0,2192.0)	1730.0 (749.0,2494.0)	1508.0 (588.0,2044.0)	1016.0 (632.0,1858.0)	1028.0 (523.0,1293.0)
*P*1	0.0687	0.0461	0.3260	0.6467	Ref
*P*2	0.4610	0.3073	0.7715	Ref	

Day 28	916.0 (704.0,1464.0)	1048.0 (488.0,1336.0)	972.0 (356.0,1760.0)	314.0 (240.0,713.0)	846.0 (205.0,1257.0)
*P*1	0.5490	0.3359	0.2773	0.5306	Ref
*P*2	0.0363	0.0644	0.0817	Ref	-
**$IL\text{-}4^{+}$ SFC per million PBMCs against omicron variant of SARS-CoV-2**					
Day 0	24.0 (0.0,36.0)	0.0 (0.0,24.0)	10.0 (0.0,59.0)	0.0 (0.0,48.0)	0.0 (0.0,28.0)
*P*1	0.1243	0.8968	0.2225	0.7757	Ref
*P*2	0.3674	0.8968	0.4238	Ref	-
Day 7	92.0 (40.0,154.0)	220.0 (144.0,508.0)	56.0 (15.0,202.0)	24.0 (0.0,40.0)	24.0 (0.0,48.0)
*P*1	0.0010	<0.0001	0.0480	0.9892	Ref
*P*2	0.0004	<0.0001	0.0579	Ref	-
Day 14	44.0 (24.0,122.0)	92.0 (68.0,148.0)	38.0 (5.0,86.0)	44.0 (28.0,68.0)	0.0 (0.0,36.0)
*P*1	0.0065	<0.0001	0.0272	0.0129	Ref
*P*2	0.5240	0.0018	0.9318	Ref	-
Day 28	38.0 (15.0,71.0)	32.0 (0.0,64.0)	22.0 (0.0,62.0)	0.0 (0.0,36.0)	0.0 (0.0,32.0)
*P*1	0.0294	0.0520	0.2122	0.6263	Ref
*P*2	0.1832	0.2639	0.7161	Ref	-

Note: IQR, interquartile range; PBMCs, peripheral blood mononuclear cells; SFC, spot forming cell. T cell responses against omicron variant of SARS-CoV-2 Spike protein was determined in samples from a random subset of the 234 participants (n=23 for ChAdTS-S, n=21 for RQ3013, n=22 for ZR202-CoV, n=21 for CoronaVac, and n=21 for placebo).

Supplementary Table 9: Local and systemic adverse events reported within 7 days after the third dose vaccination.

AEs	ChAdTS-S		RQ3013		ZR202-CoV		CoronaVac		Placebo	
n(%)	n=48		n=47		n=47		n=46		n=47	
Causality	Related	Unrelated	Related	Unrelated	Related	Unrelated	Related	Unrelated	Related	Unrelated
**Any solicited AEs**	43(89.6%)	0(0.0%)	47(100.0%)	0(0.0%)	46(97.9%)	0(0.0%)	37(80.4%)	0(0.0%)	21(44.7%)	0(0.0%)
Grade1	41(85.4%)	0(0.0%)	14(29.8%)	0(0.0%)	37(78.7%)	0(0.0%)	34(73.9%)	0(0.0%)	17(36.2%)	0(0.0%)
Grade2	2(4.2%)	0(0.0%)	29(61.7%)	0(0.0%)	9(19.1%)	0(0.0%)	3(6.5%)	0(0.0%)	2(4.3%)	0(0.0%)
Grade3	0(0.0%)	0(0.0%)	4(8.5%)	0(0.0%)	0(0.0%)	0(0.0%)	0(0.0%)	0(0.0%)	2(4.3%)	0(0.0%)
Grade4	0(0.0%)	0(0.0%)	0(0.0%)	0(0.0%)	0(0.0%)	0(0.0%)	0(0.0%)	0(0.0%)	0(0.0%)	0(0.0%)
**Any solicited local AEs**	40(83.3%)	0(0.0%)	46(97.9%)	0(0.0%)	45(95.7%)	0(0.0%)	35(76.1%)	0(0.0%)	11(23.4%)	0(0.0%)
Grade1	39(81.3%)	0(0.0%)	19(40.4%)	0(0.0%)	40(85.1%)	0(0.0%)	35(76.1%)	0(0.0%)	11(23.4%)	0(0.0%)
Grade2	1(2.1%)	0(0.0%)	26(55.3%)	0(0.0%)	5(10.6%)	0(0.0%)	0(0.0%)	0(0.0%)	0(0.0%)	0(0.0%)
Grade3	0(0.0%)	0(0.0%)	1(2.1%)	0(0.0%)	0(0.0%)	0(0.0%)	0(0.0%)	0(0.0%)	0(0.0%)	0(0.0%)
Grade4	0(0.0%)	0(0.0%)	0(0.0%)	0(0.0%)	0(0.0%)	0(0.0%)	0(0.0%)	0(0.0%)	0(0.0%)	0(0.0%)
**Pain**	40(83.3%)	0(0.0%)	46(97.9%)	0(0.0%)	45(95.7%)	0(0.0%)	34(73.9%)	0(0.0%)	10(21.3%)	0(0.0%)
Grade1	39(81.3%)	0(0.0%)	21(44.7%)	0(0.0%)	40(85.1%)	0(0.0%)	34(73.9%)	0(0.0%)	10(21.3%)	0(0.0%)
Grade2	1(2.1%)	0(0.0%)	24(51.1%)	0(0.0%)	5(10.6%)	0(0.0%)	0(0.0%)	0(0.0%)	0(0.0%)	0(0.0%)
Grade3	0(0.0%)	0(0.0%)	1(2.1%)	0(0.0%)	0(0.0%)	0(0.0%)	0(0.0%)	0(0.0%)	0(0.0%)	0(0.0%)
Grade4	0(0.0%)	0(0.0%)	0(0.0%)	0(0.0%)	0(0.0%)	0(0.0%)	0(0.0%)	0(0.0%)	0(0.0%)	0(0.0%)
**Scleroma/Swelling**	13(27.1%)	0(0.0%)	30(63.8%)	0(0.0%)	20(42.6%)	0(0.0%)	9(19.6%)	0(0.0%)	4(8.5%)	0(0.0%)
Grade1	13(27.1%)	0(0.0%)	21(44.7%)	0(0.0%)	17(36.2%)	0(0.0%)	9(19.6%)	0(0.0%)	4(8.5%)	0(0.0%)
Grade2	0(0.0%)	0(0.0%)	9(19.1%)	0(0.0%)	3(6.4%)	0(0.0%)	0(0.0%)	0(0.0%)	0(0.0%)	0(0.0%)
Grade3	0(0.0%)	0(0.0%)	0(0.0%)	0(0.0%)	0(0.0%)	0(0.0%)	0(0.0%)	0(0.0%)	0(0.0%)	0(0.0%)
Grade4	0(0.0%)	0(0.0%)	0(0.0%)	0(0.0%)	0(0.0%)	0(0.0%)	0(0.0%)	0(0.0%)	0(0.0%)	0(0.0%)
**Redness**	11(22.9%)	0(0.0%)	19(40.4%)	0(0.0%)	12(25.5%)	0(0.0%)	5(10.9%)	0(0.0%)	5(10.6%)	0(0.0%)
Grade1	11(22.9%)	0(0.0%)	15(31.9%)	0(0.0%)	10(21.3%)	0(0.0%)	5(10.9%)	0(0.0%)	5(10.6%)	0(0.0%)
Grade2	0(0.0%)	0(0.0%)	4(8.5%)	0(0.0%)	2(4.3%)	0(0.0%)	0(0.0%)	0(0.0%)	0(0.0%)	0(0.0%)
Grade3	0(0.0%)	0(0.0%)	0(0.0%)	0(0.0%)	0(0.0%)	0(0.0%)	0(0.0%)	0(0.0%)	0(0.0%)	0(0.0%)
Grade4	0(0.0%)	0(0.0%)	0(0.0%)	0(0.0%)	0(0.0%)	0(0.0%)	0(0.0%)	0(0.0%)	0(0.0%)	0(0.0%)
**Any solicited systemic AEs**	33(68.8%)	0(0.0%)	44(93.6%)	0(0.0%)	36(76.6%)	0(0.0%)	20(43.5%)	0(0.0%)	18(38.3%)	0(0.0%)
Grade1	32(66.7%)	0(0.0%)	17(36.2%)	0(0.0%)	30(63.8%)	0(0.0%)	17(37.0%)	0(0.0%)	14(29.8%)	0(0.0%)
Grade2	1(2.1%)	0(0.0%)	24(51.1%)	0(0.0%)	6(12.8%)	0(0.0%)	3(6.5%)	0(0.0%)	2(4.3%)	0(0.0%)
Grade3	0(0.0%)	0(0.0%)	3(6.4%)	0(0.0%)	0(0.0%)	0(0.0%)	0(0.0%)	0(0.0%)	2(4.3%)	0(0.0%)
Grade4	0(0.0%)	0(0.0%)	0(0.0%)	0(0.0%)	0(0.0%)	0(0.0%)	0(0.0%)	0(0.0%)	0(0.0%)	0(0.0%)
**Acute allergic reaction**	0(0.0%)	0(0.0%)	1(2.1%)	0(0.0%)	0(0.0%)	0(0.0%)	1(2.2%)	0(0.0%)	0(0.0%)	0(0.0%)
Grade1	0(0.0%)	0(0.0%)	1(2.1%)	0(0.0%)	0(0.0%)	0(0.0%)	0(0.0%)	0(0.0%)	0(0.0%)	0(0.0%)
Grade2	0(0.0%)	0(0.0%)	0(0.0%)	0(0.0%)	0(0.0%)	0(0.0%)	1(2.2%)	0(0.0%)	0(0.0%)	0(0.0%)
Grade3	0(0.0%)	0(0.0%)	0(0.0%)	0(0.0%)	0(0.0%)	0(0.0%)	0(0.0%)	0(0.0%)	0(0.0%)	0(0.0%)
Grade4	0(0.0%)	0(0.0%)	0(0.0%)	0(0.0%)	0(0.0%)	0(0.0%)	0(0.0%)	0(0.0%)	0(0.0%)	0(0.0%)
**Nausea/ Vomiting**	1(2.1%)	0(0.0%)	11(23.4%)	0(0.0%)	5(10.6%)	0(0.0%)	5(10.9%)	0(0.0%)	2(4.3%)	0(0.0%)
Grade1	1(2.1%)	0(0.0%)	9(19.1%)	0(0.0%)	5(10.6%)	0(0.0%)	5(10.9%)	0(0.0%)	1(2.1%)	0(0.0%)
Grade2	0(0.0%)	0(0.0%)	2(4.3%)	0(0.0%)	0(0.0%)	0(0.0%)	0(0.0%)	0(0.0%)	0(0.0%)	0(0.0%)
Grade3	0(0.0%)	0(0.0%)	0(0.0%)	0(0.0%)	0(0.0%)	0(0.0%)	0(0.0%)	0(0.0%)	1(2.1%)	0(0.0%)
Grade4	0(0.0%)	0(0.0%)	0(0.0%)	0(0.0%)	0(0.0%)	0(0.0%)	0(0.0%)	0(0.0%)	0(0.0%)	0(0.0%)
**Joint pain**	5(10.4%)	0(0.0%)	14(29.8%)	0(0.0%)	11(23.4%)	0(0.0%)	3(6.5%)	0(0.0%)	2(4.3%)	0(0.0%)
Grade1	5(10.4%)	0(0.0%)	10(21.3%)	0(0.0%)	10(21.3%)	0(0.0%)	3(6.5%)	0(0.0%)	1(2.1%)	0(0.0%)
Grade2	0(0.0%)	0(0.0%)	4(8.5%)	0(0.0%)	1(2.1%)	0(0.0%)	0(0.0%)	0(0.0%)	1(2.1%)	0(0.0%)
Grade3	0(0.0%)	0(0.0%)	0(0.0%)	0(0.0%)	0(0.0%)	0(0.0%)	0(0.0%)	0(0.0%)	0(0.0%)	0(0.0%)
Grade4	0(0.0%)	0(0.0%)	0(0.0%)	0(0.0%)	0(0.0%)	0(0.0%)	0(0.0%)	0(0.0%)	0(0.0%)	0(0.0%)
**Muscular pain**	16(33.3%)	0(0.0%)	32(68.1%)	0(0.0%)	26(55.3%)	0(0.0%)	9(19.6%)	0(0.0%)	5(10.6%)	0(0.0%)
Grade1	16(33.3%)	0(0.0%)	16(34%)	0(0.0%)	23(48.9%)	0(0.0%)	8(17.4%)	0(0.0%)	5(10.6%)	0(0.0%)
Grade2	0(0.0%)	0(0.0%)	16(34%)	0(0.0%)	3(6.4%)	0(0.0%)	1(2.2%)	0(0.0%)	0(0.0%)	0(0.0%)
Grade3	0(0.0%)	0(0.0%)	0(0.0%)	0(0.0%)	0(0.0%)	0(0.0%)	0(0.0%)	0(0.0%)	0(0.0%)	0(0.0%)
Grade4	0(0.0%)	0(0.0%)	0(0.0%)	0(0.0%)	0(0.0%)	0(0.0%)	0(0.0%)	0(0.0%)	0(0.0%)	0(0.0%)

**Headache**	12(25.0%)	0(0.0%)	30(63.8%)	0(0.0%)	14(29.8%)	0(0.0%)	12(26.1%)	0(0.0%)	7(14.9%)	0(0.0%)
Grade1	12(25.0%)	0(0.0%)	17(36.2%)	0(0.0%)	12(25.5%)	0(0.0%)	11(23.9%)	0(0.0%)	7(14.9%)	0(0.0%)
Grade2	0(0.0%)	0(0.0%)	12(25.5%)	0(0.0%)	2(4.3%)	0(0.0%)	1(2.2%)	0(0.0%)	0(0.0%)	0(0.0%)
Grade3	0(0.0%)	0(0.0%)	1(2.1%)	0(0.0%)	0(0.0%)	0(0.0%)	0(0.0%)	0(0.0%)	0(0.0%)	0(0.0%)
Grade4	0(0.0%)	0(0.0%)	0(0.0%)	0(0.0%)	0(0.0%)	0(0.0%)	0(0.0%)	0(0.0%)	0(0.0%)	0(0.0%)
**Chill**	11(22.9%)	0(0.0%)	31(66.0%)	0(0.0%)	13(27.7%)	0(0.0%)	6(13.0%)	0(0.0%)	9(19.1%)	0(0.0%)
Grade1	11(22.9%)	0(0.0%)	21(44.7%)	0(0.0%)	11(23.4%)	0(0.0%)	6(13.0.%)	0(0.0%)	8(17.0%)	0(0.0%)
Grade2	0(0.0%)	0(0.0%)	10(21.3%)	0(0.0%)	2(4.3%)	0(0.0%)	0(0.0%)	0(0.0%)	1(2.1%)	0(0.0%)
Grade3	0(0.0%)	0(0.0%)	0(0.0%)	0(0.0%)	0(0.0%)	0(0.0%)	0(0.0%)	0(0.0%)	0(0.0%)	0(0.0%)
Grade4	0(0.0%)	0(0.0%)	0(0.0%)	0(0.0%)	0(0.0%)	0(0.0%)	0(0.0%)	0(0.0%)	0(0.0%)	0(0.0%)
**Fatigue**	23(47.9%)	0(0.0%)	37(78.7%)	0(0.0%)	24(51.1%)	0(0.0%)	17(37.0%)	0(0.0%)	13(27.7%)	0(0.0%)
Grade1	22(45.8%)	0(0.0%)	24(51.1%)	0(0.0%)	22(46.8%)	0(0.0%)	17(37.0%)	0(0.0%)	11(23.4%)	0(0.0%)
Grade2	1(2.1%)	0(0.0%)	13(27.7%)	0(0.0%)	2(4.3%)	0(0.0%)	0(0.0%)	0(0.0%)	1(2.1%)	0(0.0%)
Grade3	0(0.0%)	0(0.0%)	0(0.0%)	0(0.0%)	0(0.0%)	0(0.0%)	0(0.0%)	0(0.0%)	1(2.1%)	0(0.0%)
Grade4	0(0.0%)	0(0.0%)	0(0.0%)	0(0.0%)	0(0.0%)	0(0.0%)	0(0.0%)	0(0.0%)	0(0.0%)	0(0.0%)
**Fever**	8(16.7%)	0(0.0%)	37(78.7%)	0(0.0%)	9(19.1%)	0(0.0%)	4(8.7%)	0(0.0%)	1(2.1%)	0(0.0%)
Grade1	8(16.7%)	0(0.0%)	16(34.0%)	0(0.0%)	8(17.0%)	0(0.0%)	3(6.5%)	0(0.0%)	0(0.0%)	0(0.0%)
Grade2	0(0.0%)	0(0.0%)	18(38.3%)	0(0.0%)	1(2.1%)	0(0.0%)	1(2.2%)	0(0.0%)	0(0.0%)	0(0.0%)
Grade3	0(0.0%)	0(0.0%)	3(6.4%)	0(0.0%)	0(0.0%)	0(0.0%)	0(0.0%)	0(0.0%)	1(2.1%)	0(0.0%)
Grade4	0(0.0%)	0(0.0%)	0(0.0%)	0(0.0%)	0(0.0%)	0(0.0%)	0(0.0%)	0(0.0%)	0(0.0%)	0(0.0%)
**Any unsolicited AEs**	0(0.0%)	0(0.0%)	0(0.0%)	0(0.0%)	0(0.0%)	0(0.0%)	0(0.0%)	0(0.0%)	0(0.0%)	0(0.0%)
Grade1	0(0.0%)	0(0.0%)	0(0.0%)	0(0.0%)	0(0.0%)	0(0.0%)	0(0.0%)	0(0.0%)	0(0.0%)	0(0.0%)
Grade2	0(0.0%)	0(0.0%)	0(0.0%)	0(0.0%)	0(0.0%)	0(0.0%)	0(0.0%)	0(0.0%)	0(0.0%)	0(0.0%)
Grade3	0(0.0%)	0(0.0%)	0(0.0%)	0(0.0%)	0(0.0%)	0(0.0%)	0(0.0%)	0(0.0%)	0(0.0%)	0(0.0%)
Grade4	0(0.0%)	0(0.0%)	0(0.0%)	0(0.0%)	0(0.0%)	0(0.0%)	0(0.0%)	0(0.0%)	0(0.0%)	0(0.0%)

Note: AE, adverse event. n=the number of exposed participants who submitted any data for the event in the given time window. Percentages are based on the number of exposed participants who submitted any data for the event.

Supplementary Table 10: Local and systemic adverse events reported within 14 days after the third dose vaccination across booster schedules.

AEs, n(%)	ChAdTS-S		RQ3013		ZR202-CoV		CoronaVac		Placebo	
	n=48		n=47		n=47		n=46		n=47	
**Causality**	**Related**	**Unrelated**	**Related**	**Unrelated**	**Related**	**Unrelated**	**Related**	**Unrelated**	**Related**	**Unrelated**
**Any solicited AEs**	43(89.6%)	2(4.2%)	47(100.0%)	0(0.0%)	46(97.9%)	0(0.0%)	37(80.4%)	1(2.2%)	21(44.7%)	2(4.3%)
Grade1	40(83.3%)	2(4.2%)	14(29.8%)	0(0.0%)	37(78.7%)	0(0.0%)	34(73.9%)	1(2.2%)	17(36.2%)	2(4.3%)
Grade2	3(6.2%)	0(0.0%)	29(61.7%)	0(0.0%)	9(19.1%)	0(0.0%)	3(6.5%)	0(0.0%)	2(4.3%)	0(0.0%)
Grade3	0(0.0%)	0(0.0%)	4(8.5%)	0(0.0%)	0(0.0%)	0(0.0%)	0(0.0%)	0(0.0%)	2(4.3%)	0(0.0%)
Grade4	0(0.0%)	0(0.0%)	0(0.0%)	0(0.0%)	0(0.0%)	0(0.0%)	0(0.0%)	0(0.0%)	0(0.0%)	0(0.0%)
**Any solicited local AEs**	40(83.3%)	0(0.0%)	46(97.9%)	0(0.0%)	45(95.7%)	0(0.0%)	35(76.1%)	0(0.0%)	11(23.4%)	0(0.0%)
Grade1	39(81.2%)	0(0.0%)	19(40.4%)	0(0.0%)	40(85.1%)	0(0.0%)	35(76.1%)	0(0.0%)	11(23.4%)	0(0.0%)
Grade2	1(2.1%)	0(0.0%)	26(55.3%)	0(0.0%)	5(10.6%)	0(0.0%)	0(0.0%)	0(0.0%)	0(0.0%)	0(0.0%)
Grade3	0(0.0%)	0(0.0%)	1(2.1%)	0(0.0%)	0(0.0%)	0(0.0%)	0(0.0%)	0(0.0%)	0(0.0%)	0(0.0%)
Grade4	0(0.0%)	0(0.0%)	0(0.0%)	0(0.0%)	0(0.0%)	0(0.0%)	0(0.0%)	0(0.0%)	0(0.0%)	0(0.0%)
**Pain**	40(83.3%)	0(0.0%)	46(97.9%)	0(0.0%)	45(95.7%)	0(0.0%)	34(73.9%)	0(0.0%)	10(21.3%)	0(0.0%)
Grade1	39(81.2%)	0(0.0%)	21(44.7%)	0(0.0%)	40(85.1%)	0(0.0%)	34(73.9%)	0(0.0%)	10(21.3%)	0(0.0%)
Grade2	1(2.1%)	0(0.0%)	24(51.1%)	0(0.0%)	5(10.6%)	0(0.0%)	0(0.0%)	0(0.0%)	0(0.0%)	0(0.0%)
Grade3	0(0.0%)	0(0.0%)	1(2.1%)	0(0.0%)	0(0.0%)	0(0.0%)	0(0.0%)	0(0.0%)	0(0.0%)	0(0.0%)
Grade4	0(0.0%)	0(0.0%)	0(0.0%)	0(0.0%)	0(0.0%)	0(0.0%)	0(0.0%)	0(0.0%)	0(0.0%)	0(0.0%)
**Scleroma/Swelling**	13(27.1%)	0(0.0%)	30(63.8%)	0(0.0%)	20(42.6%)	0(0.0%)	9(19.6%)	0(0.0%)	4(8.5%)	0(0.0%)
Grade1	13(27.1%)	0(0.0%)	21(44.7%)	0(0.0%)	17(36.2%)	0(0.0%)	9(19.6%)	0(0.0%)	4(8.5%)	0(0.0%)
Grade2	0(0.0%)	0(0.0%)	9(19.1%)	0(0.0%)	3(6.4%)	0(0.0%)	0(0.0%)	0(0.0%)	0(0.0%)	0(0.0%)
Grade3	0(0.0%)	0(0.0%)	0(0.0%)	0(0.0%)	0(0.0%)	0(0.0%)	0(0.0%)	0(0.0%)	0(0.0%)	0(0.0%)
Grade4	0(0.0%)	0(0.0%)	0(0.0%)	0(0.0%)	0(0.0%)	0(0.0%)	0(0.0%)	0(0.0%)	0(0.0%)	0(0.0%)
**Redness**	11(22.9%)	0(0.0%)	19(40.4%)	0(0.0%)	12(25.5%)	0(0.0%)	5(10.9%)	0(0.0%)	5(10.6%)	0(0.0%)
Grade1	11(22.9%)	0(0.0%)	15(31.9%)	0(0.0%)	10(21.3%)	0(0.0%)	5(10.9%)	0(0.0%)	5(10.6%)	0(0.0%)
Grade2	0(0.0%)	0(0.0%)	4(8.5%)	0(0.0%)	2(4.3%)	0(0.0%)	0(0.0%)	0(0.0%)	0(0.0%)	0(0.0%)
Grade3	0(0.0%)	0(0.0%)	0(0.0%)	0(0.0%)	0(0.0%)	0(0.0%)	0(0.0%)	0(0.0%)	0(0.0%)	0(0.0%)
Grade4	0(0.0%)	0(0.0%)	0(0.0%)	0(0.0%)	0(0.0%)	0(0.0%)	0(0.0%)	0(0.0%)	0(0.0%)	0(0.0%)
**Any Solicited systemic AEs**	33(68.8%)	2(4.2%)	44(93.6%)	0(0.0%)	36(76.6%)	0(0.0%)	20(43.5%)	1(2.2%)	18(38.3%)	2(4.3%)
Grade1	31(64.6%)	2(4.2%)	17(36.2%)	0(0.0%)	30(63.8%)	0(0.0%)	17(37%)	1(2.2%)	14(29.8%)	2(4.3%)
Grade2	2(4.2%)	0(0.0%)	24(51.1%)	0(0.0%)	6(12.8%)	0(0.0%)	3(6.5%)	0(0.0%)	2(4.3%)	0(0.0%)
Grade3	0(0.0%)	0(0.0%)	3(6.4%)	0(0.0%)	0(0.0%)	0(0.0%)	0(0.0%)	0(0.0%)	2(4.3%)	0(0.0%)
Grade4	0(0.0%)	0(0.0%)	0(0.0%)	0(0.0%)	0(0.0%)	0(0.0%)	0(0.0%)	0(0.0%)	0(0.0%)	0(0.0%)
**Acute allergic reaction**	0(0.0%)	0(0.0%)	1(2.1%)	0(0.0%)	0(0.0%)	0(0.0%)	1(2.2%)	0(0.0%)	0(0.0%)	0(0.0%)
Grade1	0(0.0%)	0(0.0%)	1(2.1%)	0(0.0%)	0(0.0%)	0(0.0%)	0(0.0%)	0(0.0%)	0(0.0%)	0(0.0%)
Grade2	0(0.0%)	0(0.0%)	0(0.0%)	0(0.0%)	0(0.0%)	0(0.0%)	1(2.2%)	0(0.0%)	0(0.0%)	0(0.0%)
Grade3	0(0.0%)	0(0.0%)	0(0.0%)	0(0.0%)	0(0.0%)	0(0.0%)	0(0.0%)	0(0.0%)	0(0.0%)	0(0.0%)
Grade4	0(0.0%)	0(0.0%)	0(0.0%)	0(0.0%)	0(0.0%)	0(0.0%)	0(0.0%)	0(0.0%)	0(0.0%)	0(0.0%)
**Nausea/Vomiting**	1(2.1%)	1(2.1%)	11(23.4%)	0(0.0%)	5(10.6%)	0(0.0%)	5(10.9%)	1(2.2%)	2(4.3%)	0(0.0%)
Grade1	1(2.1%)	1(2.1%)	9(19.1%)	0(0.0%)	5(10.6%)	0(0.0%)	5(10.9%)	1(2.2%)	1(2.1%)	0(0.0%)
Grade2	0(0.0%)	0(0.0%)	2(4.3%)	0(0.0%)	0(0.0%)	0(0.0%)	0(0.0%)	0(0.0%)	0(0.0%)	0(0.0%)
Grade3	0(0.0%)	0(0.0%)	0(0.0%)	0(0.0%)	0(0.0%)	0(0.0%)	0(0.0%)	0(0.0%)	1(2.1%)	0(0.0%)
Grade4	0(0.0%)	0(0.0%)	0(0.0%)	0(0.0%)	0(0.0%)	0(0.0%)	0(0.0%)	0(0.0%)	0(0.0%)	0(0.0%)
**Joint pain**	5(10.4%)	0(0.0%)	14(29.8%)	0(0.0%)	11(23.4%)	0(0.0%)	3(6.5%)	0(0.0%)	2(4.3%)	0(0.0%)
Grade1	5(10.4%)	0(0.0%)	10(21.3%)	0(0.0%)	10(21.3%)	0(0.0%)	3(6.5%)	0(0.0%)	1(2.1%)	0(0.0%)
Grade2	0(0.0%)	0(0.0%)	4(8.5%)	0(0.0%)	1(2.1%)	0(0.0%)	0(0.0%)	0(0.0%)	1(2.1%)	0(0.0%)
Grade3	0(0.0%)	0(0.0%)	0(0.0%)	0(0.0%)	0(0.0%)	0(0.0%)	0(0.0%)	0(0.0%)	0(0.0%)	0(0.0%)
Grade4	0(0.0%)	0(0.0%)	0(0.0%)	0(0.0%)	0(0.0%)	0(0.0%)	0(0.0%)	0(0.0%)	0(0.0%)	0(0.0%)
**Muscular pain**	16(33.3%)	0(0.0%)	32(68.1%)	0(0.0%)	26(55.3%)	0(0.0%)	9(19.6%)	0(0.0%)	5(10.6%)	0(0.0%)
Grade1	16(33.3%)	0(0.0%)	16(34.0%)	0(0.0%)	23(48.9%)	0(0.0%)	8(17.4%)	0(0.0%)	5(10.6%)	0(0.0%)
Grade2	0(0.0%)	0(0.0%)	16(34.0%)	0(0.0%)	3(6.4%)	0(0.0%)	1(2.2%)	0(0.0%)	0(0.0%)	0(0.0%)
Grade3	0(0.0%)	0(0.0%)	0(0.0%)	0(0.0%)	0(0.0%)	0(0.0%)	0(0.0%)	0(0.0%)	0(0.0%)	0(0.0%)
Grade4	0(0.0%)	0(0.0%)	0(0.0%)	0(0.0%)	0(0.0%)	0(0.0%)	0(0.0%)	0(0.0%)	0(0.0%)	0(0.0%)
**Headache**	12(25.0%)	0(0.0%)	30(63.8%)	0(0.0%)	14(29.8%)	0(0.0%)	12(26.1%)	0(0.0%)	7(14.9%)	0(0.0%)
Grade1	12(25.0%)	0(0.0%)	17(36.2%)	0(0.0%)	12(25.5%)	0(0.0%)	11(23.9%)	0(0.0%)	7(14.9%)	0(0.0%)
Grade2	0(0.0%)	0(0.0%)	12(25.5%)	0(0.0%)	2(4.3%)	0(0.0%)	1(2.2%)	0(0.0%)	0(0.0%)	0(0.0%)
Grade3	0(0.0%)	0(0.0%)	1(2.1%)	0(0.0%)	0(0.0%)	0(0.0%)	0(0.0%)	0(0.0%)	0(0.0%)	0(0.0%)

Grade4	0(0.0%)	0(0.0%)	0(0.0%)	0(0.0%)	0(0.0%)	0(0.0%)	0(0.0%)	0(0.0%)	0(0.0%)	0(0.0%)
**Chill**	11(22.9%)	1(2.1%)	31(66.0%)	0(0.0%)	13(27.7%)	0(0.0%)	6(13.0%)	0(0.0%)	9(19.1%)	1(2.1%)
Grade1	11(22.9%)	1(2.1%)	21(44.7%)	0(0.0%)	11(23.4%)	0(0.0%)	6(13.0%)	0(0.0%)	7(14.9%)	1(2.1%)
Grade2	0(0.0%)	0(0.0%)	10(21.3%)	0(0.0%)	2(4.3%)	0(0.0%)	0(0.0%)	0(0.0%)	2(4.3%)	0(0.0%)
Grade3	0(0.0%)	0(0.0%)	0(0.0%)	0(0.0%)	0(0.0%)	0(0.0%)	0(0.0%)	0(0.0%)	0(0.0%)	0(0.0%)
Grade4	0(0.0%)	0(0.0%)	0(0.0%)	0(0.0%)	0(0.0%)	0(0.0%)	0(0.0%)	0(0.0%)	0(0.0%)	0(0.0%)
**Fatigue**	23(47.9%)	0(0.0%)	37(78.7%)	0(0.0%)	24(51.1%)	0(0.0%)	17(37.0%)	0(0.0%)	13(27.7%)	0(0.0%)
Grade1	21(43.8%)	0(0.0%)	24(51.1%)	0(0.0%)	22(46.8%)	0(0.0%)	17(37.0%)	0(0.0%)	11(23.4%)	0(0.0%)
Grade2	2(4.2%)	0(0.0%)	13(27.7%)	0(0.0%)	2(4.3%)	0(0.0%)	0(0.0%)	0(0.0%)	1(2.1%)	0(0.0%)
Grade3	0(0.0%)	0(0.0%)	0(0.0%)	0(0.0%)	0(0.0%)	0(0.0%)	0(0.0%)	0(0.0%)	1(2.1%)	0(0.0%)
Grade4	0(0.0%)	0(0.0%)	0(0.0%)	0(0.0%)	0(0.0%)	0(0.0%)	0(0.0%)	0(0.0%)	0(0.0%)	0(0.0%)
**Fever**	8(16.7%)	0(0.0%)	37(78.7%)	0(0.0%)	9(19.1%)	0(0.0%)	4(8.7%)	0(0.0%)	1(2.1%)	1(2.1%)
Grade1	8(16.7%)	0(0.0%)	16(34.0%)	0(0.0%)	8(17.0%)	0(0.0%)	3(6.5%)	0(0.0%)	0(0.0%)	1(2.1%)
Grade2	0(0.0%)	0(0.0%)	18(38.3%)	0(0.0%)	1(2.1%)	0(0.0%)	1(2.2%)	0(0.0%)	0(0.0%)	0(0.0%)
Grade3	0(0.0%)	0(0.0%)	3(6.4%)	0(0.0%)	0(0.0%)	0(0.0%)	0(0.0%)	0(0.0%)	1(2.1%)	0(0.0%)
Grade4	0(0.0%)	0(0.0%)	0(0.0%)	0(0.0%)	0(0.0%)	0(0.0%)	0(0.0%)	0(0.0%)	0(0.0%)	0(0.0%)
**Any unsolicited AEs**	0(0.0%)	0(0.0%)	0(0.0%)	1(2.1%)	0(0.0%)	0(0.0%)	0(0.0%)	1(2.2%)	0(0.0%)	0(0.0%)
Grade1	0(0.0%)	0(0.0%)	0(0.0%)	0(0.0%)	0(0.0%)	0(0.0%)	0(0.0%)	0(0.0%)	0(0.0%)	0(0.0%)
Grade2	0(0.0%)	0(0.0%)	0(0.0%)	0(0.0%)	0(0.0%)	0(0.0%)	0(0.0%)	1(2.2%)	0(0.0%)	0(0.0%)
Grade3	0(0.0%)	0(0.0%)	0(0.0%)	1(2.1%)	0(0.0%)	0(0.0%)	0(0.0%)	0(0.0%)	0(0.0%)	0(0.0%)
Grade4	0(0.0%)	0(0.0%)	0(0.0%)	0(0.0%)	0(0.0%)	0(0.0%)	0(0.0%)	0(0.0%)	0(0.0%)	0(0.0%)

Note: AE, adverse event. n=the number of exposed participants who submitted any data for the event in the given time window. Percentages are based on the number of exposed participants who submitted any data for the event.

Supplementary Table 11: Local and systemic adverse events reported within 28 days after the third dose vaccination.

AEs n(%)	ChAdTS-S n=48		RQ3013 n=47		ZR202-CoV n=47		CoronaVac n=46		Placebo n=47	
**Causality**	**Related**	**Unrelated**	**Related**	**Unrelated**	**Related**	**Unrelated**	**Related**	**Unrelated**	**Related**	**Unrelated**
**Any solicited AEs**	43(89.6%)	3(6.2%)	47(100.0%)	0(0.0%)	46(97.9%)	0(0.0%)	37(80.4%)	2(4.3%)	21(44.7%)	2(4.3%)
Grade1	40(83.3%)	2(4.2%)	14(29.8%)	0(0.0%)	37(78.7%)	0(0.0%)	34(73.9%)	2(4.3%)	17(36.2%)	2(4.3%)
Grade2	3(6.2%)	1(2.1%)	29(61.7%)	0(0.0%)	9(19.1%)	0(0.0%)	3(6.5%)	0(0.0%)	2(4.3%)	0(0.0%)
Grade3	0(0.0%)	0(0.0%)	4(8.5%)	0(0.0%)	0(0.0%)	0(0.0%)	0(0.0%)	0(0.0%)	2(4.3%)	0(0.0%)
Grade4	0(0.0%)	0(0.0%)	0(0.0%)	0(0.0%)	0(0.0%)	0(0.0%)	0(0.0%)	0(0.0%)	0(0.0%)	0(0.0%)
**Any solicited local AEs**	40(83.3%)	0(0.0%)	46(97.9%)	0(0.0%)	45(95.7%)	0(0.0%)	35(76.1%)	0(0.0%)	11(23.4%)	0(0.0%)
Grade1	39(81.2%)	0(0.0%)	19(40.4%)	0(0.0%)	40(85.1%)	0(0.0%)	35(76.1%)	0(0.0%)	11(23.4%)	0(0.0%)
Grade2	1(2.1%)	0(0.0%)	26(55.3%)	0(0.0%)	5(10.6%)	0(0.0%)	0(0.0%)	0(0.0%)	0(0.0%)	0(0.0%)
Grade3	0(0.0%)	0(0.0%)	1(2.1%)	0(0.0%)	0(0.0%)	0(0.0%)	0(0.0%)	0(0.0%)	0(0.0%)	0(0.0%)
Grade4	0(0.0%)	0(0.0%)	0(0.0%)	0(0.0%)	0(0.0%)	0(0.0%)	0(0.0%)	0(0.0%)	0(0.0%)	0(0.0%)
**Pain**	40(83.3%)	0(0.0%)	46(97.9%)	0(0.0%)	45(95.7%)	0(0.0%)	34(73.9%)	0(0.0%)	10(21.3%)	0(0.0%)
Grade1	39(81.2%)	0(0.0%)	21(44.7%)	0(0.0%)	40(85.1%)	0(0.0%)	34(73.9%)	0(0.0%)	10(21.3%)	0(0.0%)
Grade2	1(2.1%)	0(0.0%)	24(51.1%)	0(0.0%)	5(10.6%)	0(0.0%)	0(0.0%)	0(0.0%)	0(0.0%)	0(0.0%)
Grade3	0(0.0%)	0(0.0%)	1(2.1%)	0(0.0%)	0(0.0%)	0(0.0%)	0(0.0%)	0(0.0%)	0(0.0%)	0(0.0%)
Grade4	0(0.0%)	0(0.0%)	0(0.0%)	0(0.0%)	0(0.0%)	0(0.0%)	0(0.0%)	0(0.0%)	0(0.0%)	0(0.0%)
**Scleroma/Swelling**	13(27.1%)	0(0.0%)	30(63.8%)	0(0.0%)	20(42.6%)	0(0.0%)	9(19.6%)	0(0.0%)	4(8.5%)	0(0.0%)
Grade1	13(27.1%)	0(0.0%)	21(44.7%)	0(0.0%)	17(36.2%)	0(0.0%)	9(19.6%)	0(0.0%)	4(8.5%)	0(0.0%)
Grade2	0(0.0%)	0(0.0%)	9(19.1%)	0(0.0%)	3(6.4%)	0(0.0%)	0(0.0%)	0(0.0%)	0(0.0%)	0(0.0%)
Grade3	0(0.0%)	0(0.0%)	0(0.0%)	0(0.0%)	0(0.0%)	0(0.0%)	0(0.0%)	0(0.0%)	0(0.0%)	0(0.0%)
Grade4	0(0.0%)	0(0.0%)	0(0.0%)	0(0.0%)	0(0.0%)	0(0.0%)	0(0.0%)	0(0.0%)	0(0.0%)	0(0.0%)
**Redness**	11(22.9%)	0(0.0%)	19(40.4%)	0(0.0%)	12(25.5%)	0(0.0%)	5(10.9%)	0(0.0%)	5(10.6%)	0(0.0%)
Grade1	11(22.9%)	0(0.0%)	15(31.9%)	0(0.0%)	10(21.3%)	0(0.0%)	5(10.9%)	0(0.0%)	5(10.6%)	0(0.0%)
Grade2	0(0.0%)	0(0.0%)	4(8.5%)	0(0.0%)	2(4.3%)	0(0.0%)	0(0.0%)	0(0.0%)	0(0.0%)	0(0.0%)
Grade3	0(0.0%)	0(0.0%)	0(0.0%)	0(0.0%)	0(0.0%)	0(0.0%)	0(0.0%)	0(0.0%)	0(0.0%)	0(0.0%)
Grade4	0(0.0%)	0(0.0%)	0(0.0%)	0(0.0%)	0(0.0%)	0(0.0%)	0(0.0%)	0(0.0%)	0(0.0%)	0(0.0%)
**Any Solicited systemic AEs**	33(68.8%)	3(6.2%)	44(93.6%)	0(0.0%)	36(76.6%)	0(0.0%)	20(43.5%)	2(4.3%)	18(38.3%)	2(4.3%)
Grade1	31(64.6%)	2(4.2%)	17(36.2%)	0(0.0%)	30(63.8%)	0(0.0%)	17(37.0%)	2(4.3%)	14(29.8%)	2(4.3%)
Grade2	2(4.2%)	1(2.1%)	24(51.1%)	0(0.0%)	6(12.8%)	0(0.0%)	3(6.5%)	0(0.0%)	2(4.3%)	0(0.0%)
Grade3	0(0.0%)	0(0.0%)	3(6.4%)	0(0.0%)	0(0.0%)	0(0.0%)	0(0.0%)	0(0.0%)	2(4.3%)	0(0.0%)
Grade4	0(0.0%)	0(0.0%)	0(0.0%)	0(0.0%)	0(0.0%)	0(0.0%)	0(0.0%)	0(0.0%)	0(0.0%)	0(0.0%)
**Acute allergic reaction**	0(0.0%)	1(2.1%)	1(2.1%)	0(0.0%)	0(0.0%)	0(0.0%)	1(2.2%)	0(0.0%)	0(0.0%)	0(0.0%)
Grade1	0(0.0%)	0(0.0%)	1(2.1%)	0(0.0%)	0(0.0%)	0(0.0%)	0(0.0%)	0(0.0%)	0(0.0%)	0(0.0%)
Grade2	0(0.0%)	1(2.1%)	0(0.0%)	0(0.0%)	0(0.0%)	0(0.0%)	1(2.2%)	0(0.0%)	0(0.0%)	0(0.0%)
Grade3	0(0.0%)	0(0.0%)	0(0.0%)	0(0.0%)	0(0.0%)	0(0.0%)	0(0.0%)	0(0.0%)	0(0.0%)	0(0.0%)
Grade4	0(0.0%)	0(0.0%)	0(0.0%)	0(0.0%)	0(0.0%)	0(0.0%)	0(0.0%)	0(0.0%)	0(0.0%)	0(0.0%)
**Nausea/ Vomiting**	1(2.1%)	1(2.1%)	11(23.4%)	0(0.0%)	5(10.6%)	0(0.0%)	5(10.9%)	1(2.2%)	2(4.3%)	0(0.0%)
Grade1	1(2.1%)	1(2.1%)	9(19.1%)	0(0.0%)	5(10.6%)	0(0.0%)	5(10.9%)	1(2.2%)	1(2.1%)	0(0.0%)
Grade2	0(0.0%)	0(0.0%)	2(4.3%)	0(0.0%)	0(0.0%)	0(0.0%)	0(0.0%)	0(0.0%)	0(0.0%)	0(0.0%)
Grade3	0(0.0%)	0(0.0%)	0(0.0%)	0(0.0%)	0(0.0%)	0(0.0%)	0(0.0%)	0(0.0%)	1(2.1%)	0(0.0%)
Grade4	0(0.0%)	0(0.0%)	0(0.0%)	0(0.0%)	0(0.0%)	0(0.0%)	0(0.0%)	0(0.0%)	0(0.0%)	0(0.0%)
**Joint pain**	5(10.4%)	0(0.0%)	14(29.8%)	0(0.0%)	11(23.4%)	0(0.0%)	3(6.5%)	0(0.0%)	2(4.3%)	0(0.0%)
Grade1	5(10.4%)	0(0.0%)	10(21.3%)	0(0.0%)	10(21.3%)	0(0.0%)	3(6.5%)	0(0.0%)	1(2.1%)	0(0.0%)
Grade2	0(0.0%)	0(0.0%)	4(8.5%)	0(0.0%)	1(2.1%)	0(0.0%)	0(0.0%)	0(0.0%)	1(2.1%)	0(0.0%)
Grade3	0(0.0%)	0(0.0%)	0(0.0%)	0(0.0%)	0(0.0%)	0(0.0%)	0(0.0%)	0(0.0%)	0(0.0%)	0(0.0%)
Grade4	0(0.0%)	0(0.0%)	0(0.0%)	0(0.0%)	0(0.0%)	0(0.0%)	0(0.0%)	0(0.0%)	0(0.0%)	0(0.0%)
**Muscular pain**	16(33.3%)	0(0.0%)	32(68.1%)	0(0.0%)	26(55.3%)	0(0.0%)	9(19.6%)	0(0.0%)	5(10.6%)	0(0.0%)
Grade1	16(33.3%)	0(0.0%)	16(34.0%)	0(0.0%)	23(48.9%)	0(0.0%)	8(17.4%)	0(0.0%)	5(10.6%)	0(0.0%)
Grade2	0(0.0%)	0(0.0%)	16(34.0%)	0(0.0%)	3(6.4%)	0(0.0%)	1(2.2%)	0(0.0%)	0(0.0%)	0(0.0%)
Grade3	0(0.0%)	0(0.0%)	0(0.0%)	0(0.0%)	0(0.0%)	0(0.0%)	0(0.0%)	0(0.0%)	0(0.0%)	0(0.0%)

Grade4	0(0.0%)	0(0.0%)	0(0.0%)	0(0.0%)	0(0.0%)	0(0.0%)	0(0.0%)	0(0.0%)	0(0.0%)	0(0.0%)
**Headache**	12(25.0%)	0(0.0%)	30(63.8%)	0(0.0%)	14(29.8%)	0(0.0%)	12(26.1%)	0(0.0%)	7(14.9%)	0(0.0%)
Grade1	12(25.0%)	0(0.0%)	17(36.2%)	0(0.0%)	12(25.5%)	0(0.0%)	11(23.9%)	0(0.0%)	7(14.9%)	0(0.0%)
Grade2	0(0.0%)	0(0.0%)	12(25.5%)	0(0.0%)	2(4.3%)	0(0.0%)	1(2.2%)	0(0.0%)	0(0.0%)	0(0.0%)
Grade3	0(0.0%)	0(0.0%)	1(2.1%)	0(0.0%)	0(0.0%)	0(0.0%)	0(0.0%)	0(0.0%)	0(0.0%)	0(0.0%)
Grade4	0(0.0%)	0(0.0%)	0(0.0%)	0(0.0%)	0(0.0%)	0(0.0%)	0(0.0%)	0(0.0%)	0(0.0%)	0(0.0%)
**Chill**	11(22.9%)	1(2.1%)	31(66.0%)	0(0.0%)	13(27.7%)	0(0.0%)	6(13.0%)	1(2.2%)	9(19.1%)	1(2.1%)
Grade1	10(20.8%)	1(2.1%)	21(44.7%)	0(0.0%)	11(23.4%)	0(0.0%)	6(13.0%)	1(2.2%)	7(14.9%)	1(2.1%)
Grade2	1(2.1%)	0(0.0%)	10(21.3%)	0(0.0%)	2(4.3%)	0(0.0%)	0(0.0%)	0(0.0%)	2(4.3%)	0(0.0%)
Grade3	0(0.0%)	0(0.0%)	0(0.0%)	0(0.0%)	0(0.0%)	0(0.0%)	0(0.0%)	0(0.0%)	0(0.0%)	0(0.0%)
Grade4	0(0.0%)	0(0.0%)	0(0.0%)	0(0.0%)	0(0.0%)	0(0.0%)	0(0.0%)	0(0.0%)	0(0.0%)	0(0.0%)
**Fatigue**	23(47.9%)	0(0.0%)	37(78.7%)	0(0.0%)	24(51.1%)	0(0.0%)	17(37.0%)	0(0.0%)	13(27.7%)	0(0.0%)
Grade1	21(43.8%)	0(0.0%)	24(51.1%)	0(0.0%)	22(46.8%)	0(0.0%)	17(37.0%)	0(0.0%)	11(23.4%)	0(0.0%)
Grade2	2(4.2%)	0(0.0%)	13(27.7%)	0(0.0%)	2(4.3%)	0(0.0%)	0(0.0%)	0(0.0%)	1(2.1%)	0(0.0%)
Grade3	0(0.0%)	0(0.0%)	0(0.0%)	0(0.0%)	0(0.0%)	0(0.0%)	0(0.0%)	0(0.0%)	1(2.1%)	0(0.0%)
Grade4	0(0.0%)	0(0.0%)	0(0.0%)	0(0.0%)	0(0.0%)	0(0.0%)	0(0.0%)	0(0.0%)	0(0.0%)	0(0.0%)
**Fever**	8(16.7%)	0(0.0%)	37(78.7%)	0(0.0%)	9(19.1%)	0(0.0%)	4(8.7%)	0(0.0%)	1(2.1%)	1(2.1%)
Grade1	8(16.7%)	0(0.0%)	16(34.0%)	0(0.0%)	8(17.0%)	0(0.0%)	3(6.5%)	0(0.0%)	0(0.0%)	1(2.1%)
Grade2	0(0.0%)	0(0.0%)	18(38.3%)	0(0.0%)	1(2.1%)	0(0.0%)	1(2.2%)	0(0.0%)	0(0.0%)	0(0.0%)
Grade3	0(0.0%)	0(0.0%)	3(6.4%)	0(0.0%)	0(0.0%)	0(0.0%)	0(0.0%)	0(0.0%)	1(2.1%)	0(0.0%)
Grade4	0(0.0%)	0(0.0%)	0(0.0%)	0(0.0%)	0(0.0%)	0(0.0%)	0(0.0%)	0(0.0%)	0(0.0%)	0(0.0%)
**Any unsolicited AEs**	0(0.0%)	0(0.0%)	0(0.0%)	1(2.1%)	0(0.0%)	0(0.0%)	0(0.0%)	1(2.2%)	0(0.0%)	0(0.0%)
Grade1	0(0.0%)	0(0.0%)	0(0.0%)	0(0.0%)	0(0.0%)	0(0.0%)	0(0.0%)	0(0.0%)	0(0.0%)	0(0.0%)
Grade2	0(0.0%)	0(0.0%)	0(0.0%)	0(0.0%)	0(0.0%)	0(0.0%)	0(0.0%)	1(2.2%)	0(0.0%)	0(0.0%)
Grade3	0(0.0%)	0(0.0%)	0(0.0%)	1(2.1%)	0(0.0%)	0(0.0%)	0(0.0%)	0(0.0%)	0(0.0%)	0(0.0%)
Grade4	0(0.0%)	0(0.0%)	0(0.0%)	0(0.0%)	0(0.0%)	0(0.0%)	0(0.0%)	0(0.0%)	0(0.0%)	0(0.0%)

Note: AE, adverse event. n=the number of exposed participants who submitted any data for the event in the given time window. Percentages are based on the number of exposed participants who submitted any data for the event.

Supplementary Table 12: Days until resolution of solicited/unsolicited adverse events reported within 14 days after the third dose vaccination.

AEs	ChAdTS-S	RQ3013	ZR202-CoV	CoronaVac	Placebo
Any solicited AEs	1.0(1.0,3.0)	2.0(1.0,3.0)	3.0(1.0,5.0)	1.0(1.0,3.0)	1.0(1.0,2.0)
Any solicited local AEs	2.0(1.0,4.0)	3.0(2.0,4.0)	4.0(3.0,5.0)	1.5(1.0,3.0)	1.0(1.0,1.0)
Pain	2.0(1.0,4.0)	3.0(2.0,4.0)	4.0(3.0,6.0)	2.0(1.0,3.0)	1.0(1.0,1.8)
Scleroma/Swelling	3.0(2.0,5.0)	2.5(1.0,4.8)	3.0(2.0,4.3)	1.0(1.0,3.0)	1.0(1.0,1.0)
Redness	1.0(1.0,2.0)	2.0(1.0,5.0)	2.5(1.0,5.0)	1.0(1.0,2.0)	1.0(1.0,1.0)
Any Solicited systemic AEs	1.0(1.0,3.0)	2.0(1.0,2.0)	2.0(1.0,3.0)	1.0(1.0,3.0)	1.0(1.0,4.0)
Acute allergic reaction	-	1.0(-)	-	1.0(-)	-
Nausea/Vomiting	1.0(1.0,1.0)	1.0(1.0,2.0)	1.0(1.0,3.0)	1.5(1.0,2.0)	2.0(1.5,2.5)
Joint pain	1.0(1.0,3.0)	2.0(1.0,2.0)	1.0(1.0,5.5)	1.0(1.0,1.0)	1.0(1.0,1.0)
Muscular pain	1.0(1.0,2.3)	2.0(1.0,3.0)	3.0(1.0,4.0)	1.0(1.0,2.0)	1.0(1.0,2.0)
Headache	1.0(1.0,1.3)	1.0(1.0,2.0)	2.0(1.0,2.0)	1.0(1.0,3.0)	1.0(1.0,4.5)
Chill	2.5(1.0,3.3)	1.0(1.0,2.0)	2.0(1.0,3.0)	4.0(1.5,5.0)	2.0(1.0,4.0)
Fatigue	1.5(1.0,3.0)	2.0(1.0,3.0)	2.0(1.0,3.0)	1.0(1.0,3.0)	1.0(1.0,4.0)
Fever	1.0(1.0,1.0)	1.0(1.0,2.0)	1.0(1.0,2.0)	1.0(1.0,2.0)	1.0(1.0,1.0)
Unsolicited	-	2.0(-)	-	4.0(-)	-

Note: AE, adverse event; Median and interquartile range values are presented.

Supplementary Table 13: Grade 3 adverse events reported within 7 days after the third dose vaccination.

AEs n(%)	ChAdTS-S n=48		RQ3013 n= 47		ZR202-CoV n=47		CoronaVac n=46		Placebo n=47	
**Causality**	**Related**	**Unrelated**	**Related**	**Unrelated**	**Related**	**Unrelated**	**Related**	**Unrelated**	**Related**	**Unrelated**
Any solicited AEs	0(0.0%)	0(0.0%)	4(8.5%)	0(0.0%)	0(0.0%)	0(0.0%)	0(0.0%)	0(0.0%)	2(4.3%)	0(0.0%)
Any solicited local AEs	0(0.0%)	0(0.0%)	1(2.1%)	0(0.0%)	0(0.0%)	0(0.0%)	0(0.0%)	0(0.0%)	0(0.0%)	0(0.0%)
Pain	0(0.0%)	0(0.0%)	1(2.1%)	0(0.0%)	0(0.0%)	0(0.0%)	0(0.0%)	0(0.0%)	0(0.0%)	0(0.0%)
Scleroma/Swelling	0(0.0%)	0(0.0%)	0(0.0%)	0(0.0%)	0(0.0%)	0(0.0%)	0(0.0%)	0(0.0%)	0(0.0%)	0(0.0%)
Redness	0(0.0%)	0(0.0%)	0(0.0%)	0(0.0%)	0(0.0%)	0(0.0%)	0(0.0%)	0(0.0%)	0(0.0%)	0(0.0%)
Any solicited systemic AEs	0(0.0%)	0(0.0%)	3(6.4%)	0(0.0%)	0(0.0%)	0(0.0%)	0(0.0%)	0(0.0%)	2(4.3%)	0(0.0%)
Acute allergic reaction	0(0.0%)	0(0.0%)	0(0.0%)	0(0.0%)	0(0.0%)	0(0.0%)	0(0.0%)	0(0.0%)	0(0.0%)	0(0.0%)
Nausea/ Vomiting	0(0.0%)	0(0.0%)	0(0.0%)	0(0.0%)	0(0.0%)	0(0.0%)	0(0.0%)	0(0.0%)	1(2.1%)	0(0.0%)
Joint pain	0(0.0%)	0(0.0%)	0(0.0%)	0(0.0%)	0(0.0%)	0(0.0%)	0(0.0%)	0(0.0%)	0(0.0%)	0(0.0%)
Muscular pain	0(0.0%)	0(0.0%)	0(0.0%)	0(0.0%)	0(0.0%)	0(0.0%)	0(0.0%)	0(0.0%)	0(0.0%)	0(0.0%)
Headache	0(0.0%)	0(0.0%)	1(2.1%)	0(0.0%)	0(0.0%)	0(0.0%)	0(0.0%)	0(0.0%)	0(0.0%)	0(0.0%)
Chill	0(0.0%)	0(0.0%)	0(0.0%)	0(0.0%)	0(0.0%)	0(0.0%)	0(0.0%)	0(0.0%)	0(0.0%)	0(0.0%)
Fatigue	0(0.0%)	0(0.0%)	0(0.0%)	0(0.0%)	0(0.0%)	0(0.0%)	0(0.0%)	0(0.0%)	1(2.1%)	0(0.0%)
Fever	0(0.0%)	0(0.0%)	3(6.4%)	0(0.0%)	0(0.0%)	0(0.0%)	0(0.0%)	0(0.0%)	1(2.1%)	0(0.0%)
Any unsolicited AEs	0(0.0%)	0(0.0%)	0(0.0%)	0(0.0%)	0(0.0%)	0(0.0%)	0(0.0%)	0(0.0%)	0(0.0%)	0(0.0%)

Note: AE, adverse event.

Supplementary Table 14: Grade 3 adverse events reported within 14 days after the third dose vaccination.

AEs n(%)	ChAdTS-S		RQ3013		ZR202-CoV		CoronaVac		Placebo	
	n=48		n=47		n=47		n=46		n=47	
**Causality**	**Related**	**Unrelated**	**Related**	**Unrelated**	**Related**	**Unrelated**	**Related**	**Unrelated**	**Related**	**Unrelated**
Any solicited AEs	0(0.0%)	0(0.0%)	4(8.5%)	0(0.0%)	0(0.0%)	0(0.0%)	0(0.0%)	0(0.0%)	2(4.3%)	0(0.0%)
Any solicited local AEs	0(0.0%)	0(0.0%)	1(2.1%)	0(0.0%)	0(0.0%)	0(0.0%)	0(0.0%)	0(0.0%)	0(0.0%)	0(0.0%)
Pain	0(0.0%)	0(0.0%)	1(2.1%)	0(0.0%)	0(0.0%)	0(0.0%)	0(0.0%)	0(0.0%)	0(0.0%)	0(0.0%)
Scleroma/Swelling	0(0.0%)	0(0.0%)	0(0.0%)	0(0.0%)	0(0.0%)	0(0.0%)	0(0.0%)	0(0.0%)	0(0.0%)	0(0.0%)
Redness	0(0.0%)	0(0.0%)	0(0.0%)	0(0.0%)	0(0.0%)	0(0.0%)	0(0.0%)	0(0.0%)	0(0.0%)	0(0.0%)
Any Solicited systemic AEs	0(0.0%)	0(0.0%)	3(6.4%)	0(0.0%)	0(0.0%)	0(0.0%)	0(0.0%)	0(0.0%)	2(4.3%)	0(0.0%)
Acute allergic reaction	0(0.0%)	0(0.0%)	0(0.0%)	0(0.0%)	0(0.0%)	0(0.0%)	0(0.0%)	0(0.0%)	0(0.0%)	0(0.0%)
Nausea/Vomiting	0(0.0%)	0(0.0%)	0(0.0%)	0(0.0%)	0(0.0%)	0(0.0%)	0(0.0%)	0(0.0%)	1(2.1%)	0(0.0%)
Joint pain	0(0.0%)	0(0.0%)	0(0.0%)	0(0.0%)	0(0.0%)	0(0.0%)	0(0.0%)	0(0.0%)	0(0.0%)	0(0.0%)
Muscular pain	0(0.0%)	0(0.0%)	0(0.0%)	0(0.0%)	0(0.0%)	0(0.0%)	0(0.0%)	0(0.0%)	0(0.0%)	0(0.0%)
Headache	0(0.0%)	0(0.0%)	1(2.1%)	0(0.0%)	0(0.0%)	0(0.0%)	0(0.0%)	0(0.0%)	0(0.0%)	0(0.0%)
Chill	0(0.0%)	0(0.0%)	0(0.0%)	0(0.0%)	0(0.0%)	0(0.0%)	0(0.0%)	0(0.0%)	0(0.0%)	0(0.0%)
Fatigue	0(0.0%)	0(0.0%)	0(0.0%)	0(0.0%)	0(0.0%)	0(0.0%)	0(0.0%)	0(0.0%)	1(2.1%)	0(0.0%)
Fever	0(0.0%)	0(0.0%)	3(6.4%)	0(0.0%)	0(0.0%)	0(0.0%)	0(0.0%)	0(0.0%)	1(2.1%)	0(0.0%)
Any unsolicited AEs	0(0.0%)	0(0.0%)	0(0.0%)	1(2.1%)	0(0.0%)	0(0.0%)	0(0.0%)	0(0.0%)	0(0.0%)	0(0.0%)

Note: AE, adverse event.

Supplementary Table 15: Grade 3 adverse events reported within 28 days after the third dose vaccination.

AEs n(%)	ChAdTS-S n=48		RQ3013 n= 47		ZR202-CoV n=47		CoronaVac n=46		Placebo n=47	
**Causality**	**Related**	**Unrelated**	**Related**	**Unrelated**	**Related**	**Unrelated**	**Related**	**Unrelated**	**Related**	**Unrelated**
Any solicited AEs	0(0.0%)	0(0.0%)	4(8.5%)	0(0.0%)	0(0.0%)	0(0.0%)	0(0.0%)	0(0.0%)	2(4.3%)	0(0.0%)
Any solicited local AEs	0(0.0%)	0(0.0%)	1(2.1%)	0(0.0%)	0(0.0%)	0(0.0%)	0(0.0%)	0(0.0%)	0(0.0%)	0(0.0%)
Pain	0(0.0%)	0(0.0%)	1(2.1%)	0(0.0%)	0(0.0%)	0(0.0%)	0(0.0%)	0(0.0%)	0(0.0%)	0(0.0%)
Scleroma/Swelling	0(0.0%)	0(0.0%)	0(0.0%)	0(0.0%)	0(0.0%)	0(0.0%)	0(0.0%)	0(0.0%)	0(0.0%)	0(0.0%)
Redness	0(0.0%)	0(0.0%)	0(0.0%)	0(0.0%)	0(0.0%)	0(0.0%)	0(0.0%)	0(0.0%)	0(0.0%)	0(0.0%)
Any Solicited systemic AEs	0(0.0%)	0(0.0%)	3(6.4%)	0(0.0%)	0(0.0%)	0(0.0%)	0(0.0%)	0(0.0%)	2(4.3%)	0(0.0%)
Acute allergic reaction	0(0.0%)	0(0.0%)	0(0.0%)	0(0.0%)	0(0.0%)	0(0.0%)	0(0.0%)	0(0.0%)	0(0.0%)	0(0.0%)
Nausea/ Vomiting	0(0.0%)	0(0.0%)	0(0.0%)	0(0.0%)	0(0.0%)	0(0.0%)	0(0.0%)	0(0.0%)	1(2.1%)	0(0.0%)
Joint pain	0(0.0%)	0(0.0%)	0(0.0%)	0(0.0%)	0(0.0%)	0(0.0%)	0(0.0%)	0(0.0%)	0(0.0%)	0(0.0%)
Muscular pain	0(0.0%)	0(0.0%)	0(0.0%)	0(0.0%)	0(0.0%)	0(0.0%)	0(0.0%)	0(0.0%)	0(0.0%)	0(0.0%)
Headache	0(0.0%)	0(0.0%)	1(2.1%)	0(0.0%)	0(0.0%)	0(0.0%)	0(0.0%)	0(0.0%)	0(0.0%)	0(0.0%)
Chill	0(0.0%)	0(0.0%)	0(0.0%)	0(0.0%)	0(0.0%)	0(0.0%)	0(0.0%)	0(0.0%)	0(0.0%)	0(0.0%)
Fatigue	0(0.0%)	0(0.0%)	0(0.0%)	0(0.0%)	0(0.0%)	0(0.0%)	0(0.0%)	0(0.0%)	1(2.1%)	0(0.0%)
Fever	0(0.0%)	0(0.0%)	3(6.4%)	0(0.0%)	0(0.0%)	0(0.0%)	0(0.0%)	0(0.0%)	1(2.1%)	0(0.0%)
Any unsolicited AEs	0(0.0%)	0(0.0%)	0(0.0%)	1(2.1%)	0(0.0%)	0(0.0%)	0(0.0%)	0(0.0%)	0(0.0%)	0(0.0%)

Note: AE, adverse event.

Supplementary Table 16: The three serious adverse events reported within 3 months in this study.

Study group	Serious adverse event	Days to onset from the booster vaccination	Severity	Serious adverse event type	Causality assessment	Outcome
Placebo	Dizzy and Vomiting	1	Grade 3	Hospitalisation	Injection-related	Recovered after 3 days of hospitalisation showing no abnormal findings of intracranial image or laboratory tests
Placebo	Appendicitis	79	Grade 3	Hospitalisation	Unrelated	Undergone surgery, and recovered and were discharged home after 5 days.
Placebo	Klebsiella pneumoniae pneumonia	83	Grade 4	Hospitalisation	Unrelated	Treated in the intensive cure unit, and recovered and were discharged home after 39 days.

Supplementary Table 17: Booster immunogenicity effects of RQ3013 and BNT162b2 on the basis of two-dose of CoronaVac.

Comparable study	Strain	Measurement method	Antibody test date	BNT162b2/CoronaVac (GMR)	RQ3013/CoronaVac (GMR)
RHH-001 study in Brazil<br>*Lancet* 399(10324): 521-529 (2022)<br>Study design: RCT	wild-type	Pseudovirus titers	28 days	15.6	-
		Live-virus titers		-	11.24
		Competitive Inhibition		-	13.79
	delta	Live-virus titers		27.1	11.1
	**omicron**			**13.1**	**23.7**
A study in Hongkong<br>*Nature Medicine* 28:486-489 (2022)<br>Study design: Cohort	wild-type	Live-virus titers	21-35 days	4.7	-
			28 days	-	11.1
	**omicron**		21-35 days	**6.6**	-
			28 days	-	**23.7**
A study in Thailand<br>*The Journal of Infectious Diseases* jiac092 (2022)<br>Study design: Cohort	delta	Live-virus titers	28 days	18.5	11.1
	**omicron**			**11.3**	**23.7**

Note: All the three published studies as well as this study were designed to evaluate the booster effect of a third dose (including mRNA vaccines) in participants primed with two-dose CoronaVac. GMR, geometric mean ratio; RCT, randomised controlled trial.

Supplementary Figures

Supplementary Figure 1: An example of interferon (IFN)-γ, interleukin (IL)-4 and granzyme B-secreting T cell responses against SARS-CoV-2 Spike protein measured by FluoroSpot at day 7 after the third dose vaccination.

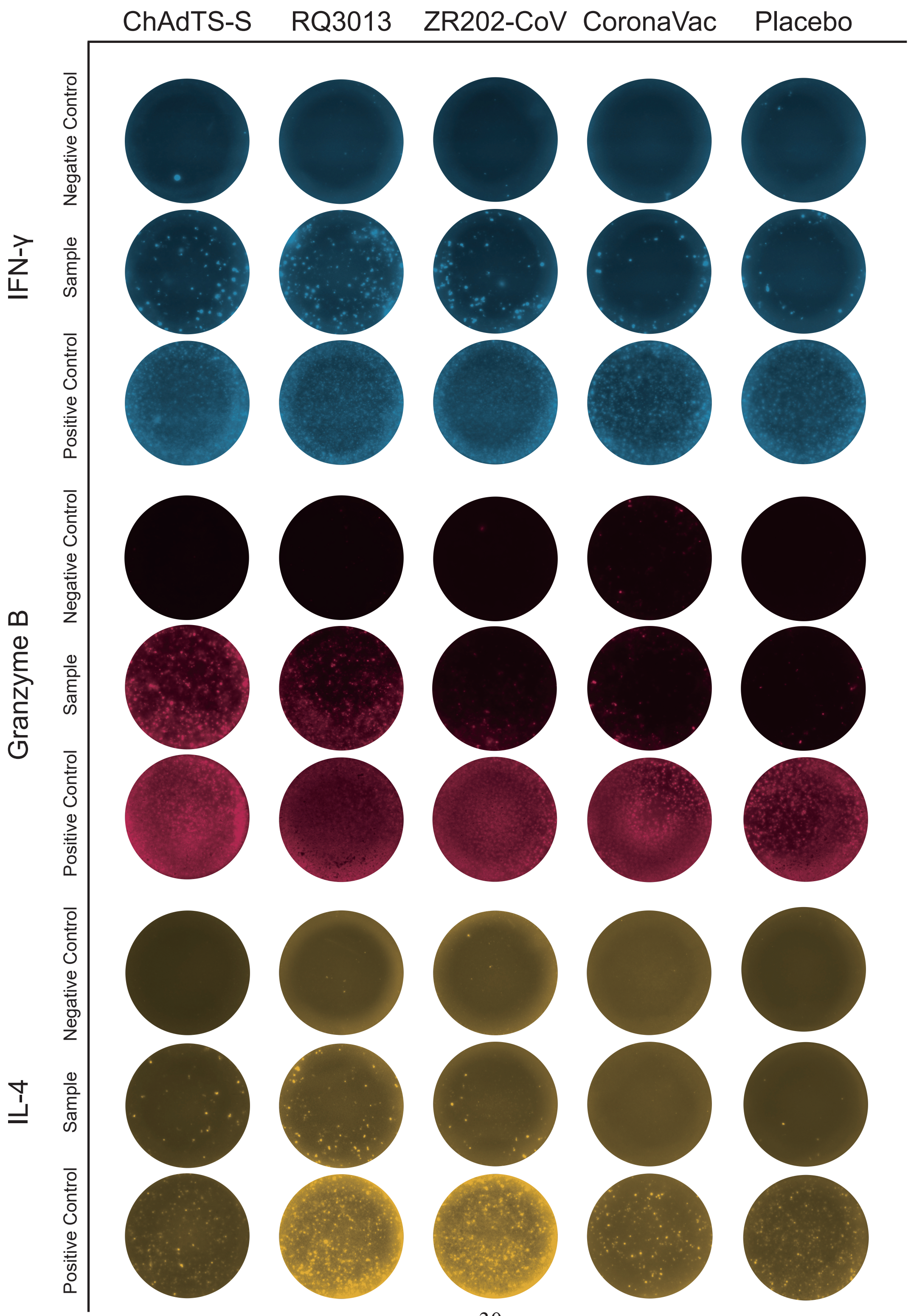
ChAdTS-S
RQ3013
ZR202-CoV
CoronaVac
Placebo
IFN-γ
Granzyme B
IL-4
Negative Control
Sample
Positive Control

Supplementary Figure 2: An example of interferon (IFN)-γ, interleukin (IL)-4 and granzyme B-secreting T cell responses against SARS-CoV-2 Spike protein measured by FluoroSpot at day 14 after the third dose vaccination.

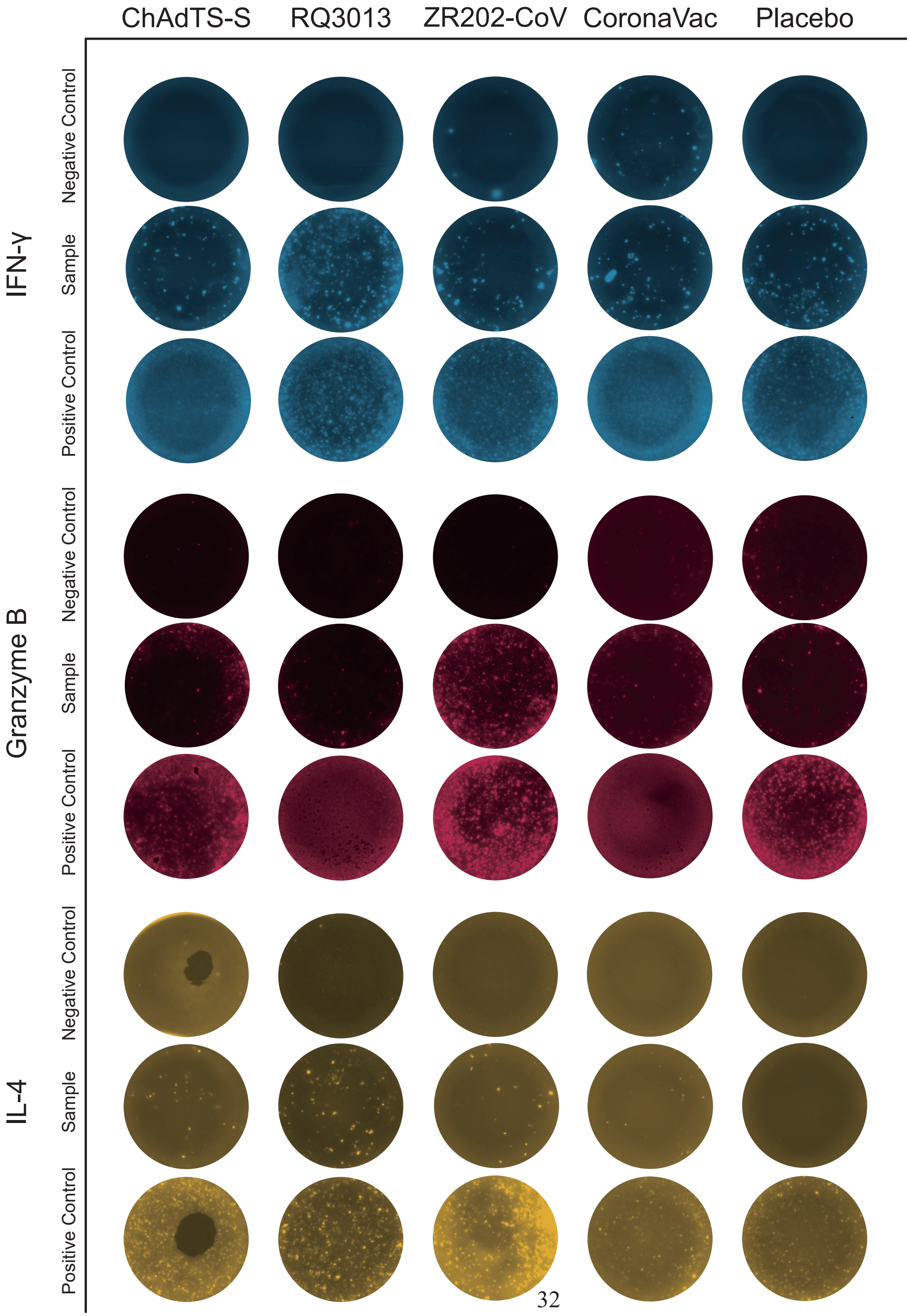
ChAdTS-S
RQ3013
ZR202-CoV
CoronaVac
Placebo
IFN-γ
Granzyme B
IL-4
Negative Control
Sample
Positive Control

Supplementary Figure 3: Correlations between neutralising antibody against wild-type SARS-CoV-2 and the prime-boost interval.

Neutralising antibody was determined with cytopathic effect (CPE)-based microneutralisation assay using the wild-type strain (Wuhan-1, GenBank: MT123291) of live SARS-CoV-2.

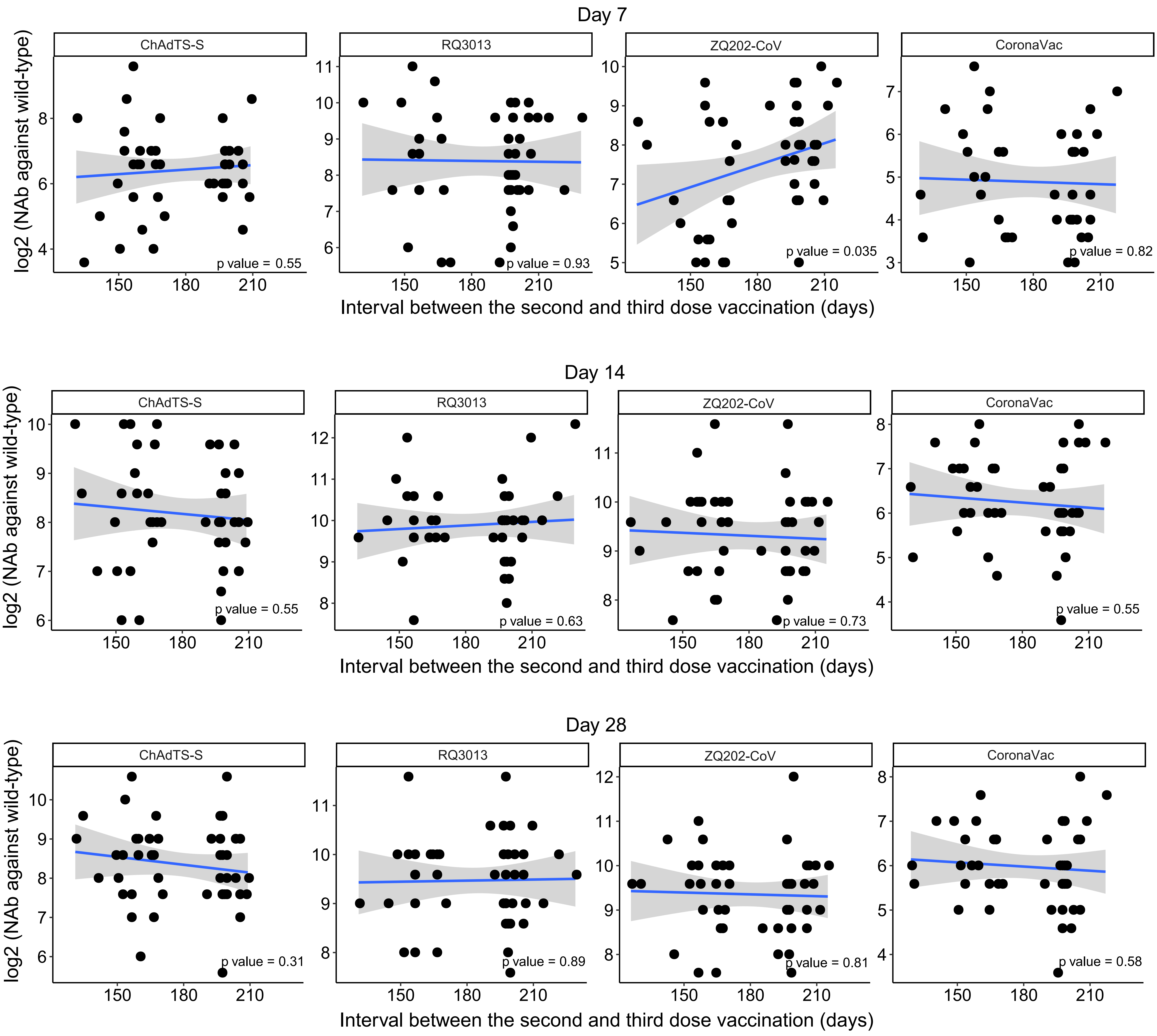

Day 7
ChAdTS-S
RQ3013
ZQ202-CoV
CoronaVac
log2 (NAb against wild-type)
p value = 0.55
p value = 0.93
p value = 0.035
p value = 0.82
Interval between the second and third dose vaccination (days)
Day 14
ChAdTS-S
RQ3013
ZQ202-CoV
CoronaVac
log2 (NAb against wild-type)
p value = 0.55
p value = 0.63
p value = 0.73
p value = 0.55
Interval between the second and third dose vaccination (days)
Day 28
ChAdTS-S
RQ3013
ZQ202-CoV
CoronaVac
log2 (NAb against wild-type)
p value = 0.31
p value = 0.89
p value = 0.81
p value = 0.58
Interval between the second and third dose vaccination (days)

Supplementary Figure 4: Correlations between neutralising antibody against the delta variant of SARS-CoV-2 and the prime-boost interval.

Neutralising antibody was determined with cytopathic effect (CPE)-based microneutralisation assay using the delta variant (B.1.617.2, IQTC-IM2175251) of live SARS-CoV-2.

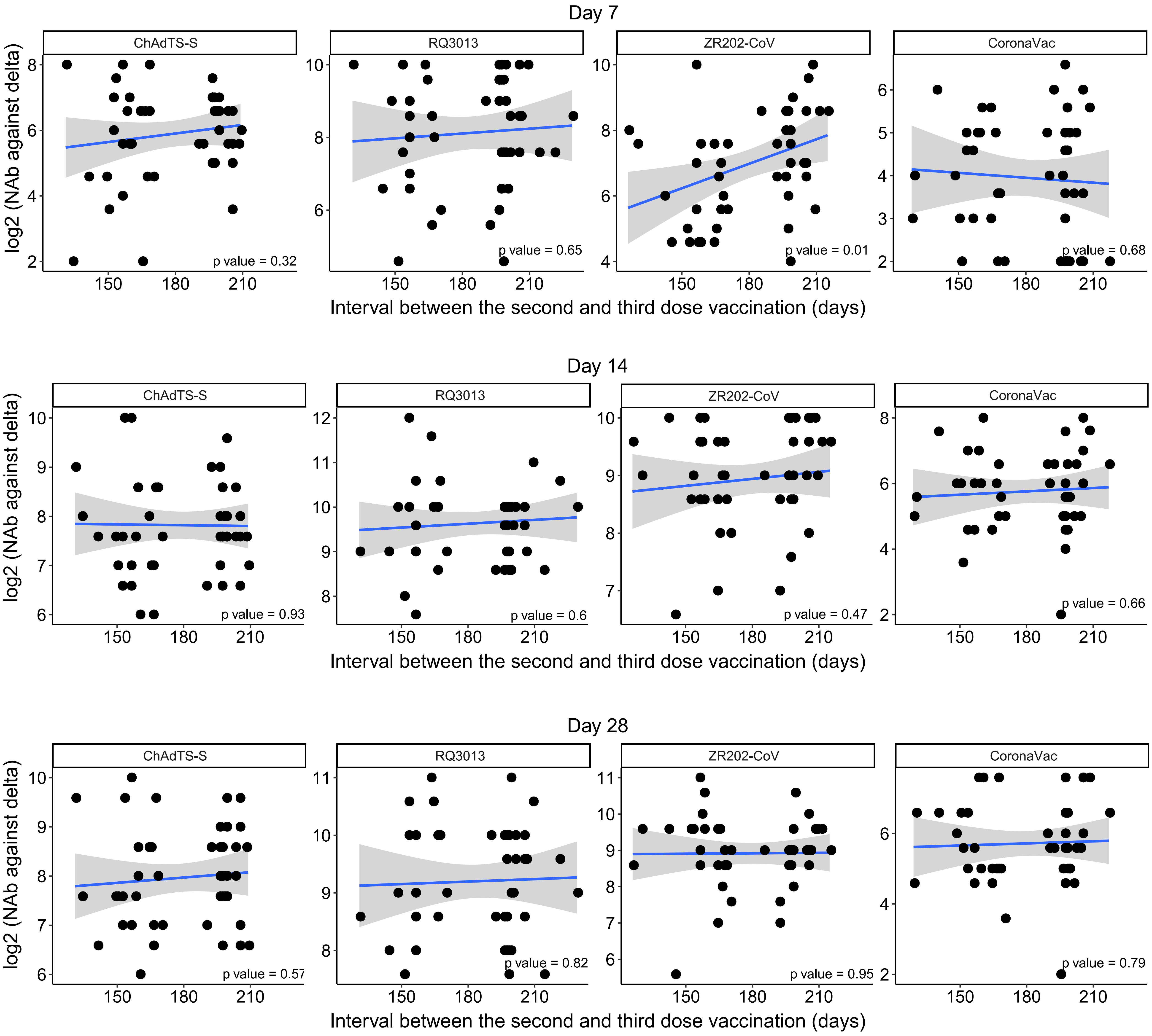
Day 7
ChAdTS-S
RQ3013
ZR202-CoV
CoronaVac
log2 (NAb against delta)
p value = 0.32
p value = 0.65
p value = 0.01
p value = 0.68
Interval between the second and third dose vaccination (days)
Day 14
ChAdTS-S
RQ3013
ZR202-CoV
CoronaVac
log2 (NAb against delta)
p value = 0.93
p value = 0.6
p value = 0.47
p value = 0.66
Interval between the second and third dose vaccination (days)
Day 28
ChAdTS-S
RQ3013
ZR202-CoV
CoronaVac
log2 (NAb against delta)
p value = 0.57
p value = 0.82
p value = 0.95
p value = 0.79
Interval between the second and third dose vaccination (days)

Supplementary Figure 5: Correlations between neutralising antibody against the omicron variant of SARS-CoV-2 and the prime-boost interval.

Neutralising antibody was determined with cytopathic effect (CPE)-based microneutralisation assay using the omicron variant (BA.1.1, IQTC-Y216017) of live SARS-CoV-2.

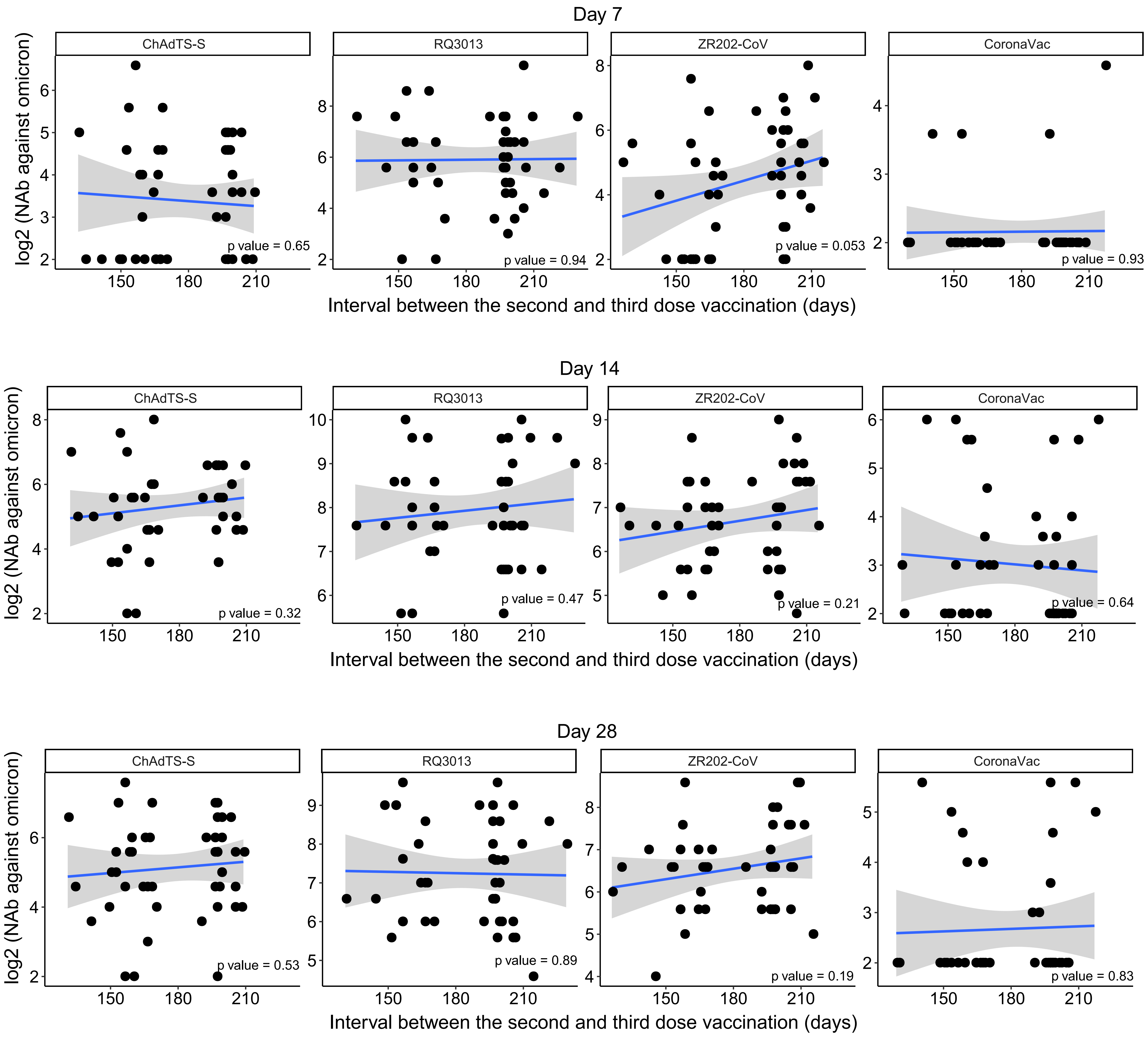
Day 7
ChAdTS-S
RQ3013
ZR202-CoV
CoronaVac
log2 (NAb against omicron)
p value = 0.65
p value = 0.94
p value = 0.053
p value = 0.93
Interval between the second and third dose vaccination (days)
Day 14
ChAdTS-S
RQ3013
ZR202-CoV
CoronaVac
log2 (NAb against omicron)
p value = 0.32
p value = 0.47
p value = 0.21
p value = 0.64
Interval between the second and third dose vaccination (days)
Day 28
ChAdTS-S
RQ3013
ZR202-CoV
CoronaVac
log2 (NAb against omicron)
p value = 0.53
p value = 0.89
p value = 0.19
p value = 0.83
Interval between the second and third dose vaccination (days)

Supplementary Figure 6: Subgroup analysis of neutralising antibodies against SARS-CoV-2 at day 7 after the third dose vaccination stratified by the prime-boost interval.

Neutralising antibodies were determined with cytopathic effect (CPE)-based microneutralisation assay using authentic SARS-CoV-2 virus, including the wild-type strain (Wuhan-1, GenBank: MT123291), the delta variant (B.1.617.2, IQTC-IM2175251) and the omicron variant (BA.1.1, IQTC-Y216017) as well as with competitive inhibition method.

	Geometric mean titre (95% CI)	n	Geometric mean ratio (95% CI)	p value for interaction
**Neutralising antibody against wild-type of live SARS-CoV-2**				
Placebo				
<180	4.9 (2.9-8.1)	17	Ref	
≥180	5.1 (3.6-7.4)	29	Ref	
ChAdTS-S				0.7088
<180	81.7 (52.3-127.4)	23	16.8 (10.1-28.1)	
≥180	93.1 (63.3-137.0)	25	18.1 (11.7-28.0)	
RQ3013				0.9221
<180	340.7 (211.7-548.1)	18	70.2 (40.8-120.7)	
≥180	353.0 (240.9-517.3)	29	68.8 (45.2-104.7)	
ZR202-CoV				0.0463
<180	118.7 (75.8-185.9)	22	24.5 (14.6-40.9)	
≥180	242.1 (163.5-358.5)	25	47.2 (30.6-72.7)	
CoronaVac				0.9167
<180	31.5 (19.7-50.4)	20	6.5 (3.8-11.0)	
≥180	30.3 (20.7-44.5)	26	5.9 (3.8-9.1)	
**Neutralising antibody against the delta variant of live SARS-CoV-2**				
Placebo				
<180	4 (2.2-7.0)	17	Ref	
≥180	4.8 (3.2-7.2)	29	Ref	
ChAdTS-S				0.3028
<180	48.2 (29.2-79.5)	23	12.2 (6.9-21.6)	
≥180	72.4 (46.9-111.7)	25	15.2 (9.3-24.7)	
RQ3013				0.4152
<180	231.5 (135.7-394.9)	18	58.5 (31.9-107.2)	
≥180	323.1 (210.6-495.6)	29	67.6 (42.3-108.2)	
ZR202-CoV				0.0092
<180	71 (42.9-117.4)	22	17.9 (10.1-31.9)	
≥180	203.1 (130.7-315.7)	25	42.5 (26.2-69.1)	
CoronaVac				0.6963
<180	14.1 (8.3-23.9)	20	3.6 (2.0-6.4)	
≥180	16.5 (10.7-25.4)	26	3.5 (2.1-5.6)	
**Neutralising antibody against the omicron variant of live SARS-CoV-2**				
Placebo				
<180	4.1 (2.4-7.1)	17	Ref	
≥180	3.9(2.7-5.8)	29	Ref	
ChAdTS-S				0.8919
<180	10.9 (6.8-17.4)	23	2.6 (1.5-4.5)	
≥180	10.3 (6.9-15.5)	25	2.6 (1.7-4.2)	
RQ3013				0.6756
<180	55.0 (33.3-90.8)	18	13.3 (7.5-23.5)	
≥180	64.6 (43.2-96.5)	29	16.4 (10.6-25.5)	
ZR202-CoV				0.0506
<180	14.5 (9.1-23.3)	22	3.5 (2.0-6.0)	
≥180	30.3 (20.0-45.9)	25	7.7 (4.9-12.2)	
CoronaVac				0.9242
<180	4.3 (2.6-7.0)	20	1.0 (0.6-1.8)	
≥180	4.4 (3.0-6.6)	26	1.1 (0.7-1.8)	
**Neutralising antibody measured by competitive inhibition method**				
Placebo				
<180	172.7 (102.1-292.1)	17	Ref	
≥180	141 (96.3-206.4)	29	Ref	
ChAdTS-S				0.5350
<180	4927.9 (3114.3-7797.7)	23	28.5 (16.8-48.4)	
≥180	6169.3 (4142.2-9188.5)	25	43.8 (27.9-68.7)	
RQ3013				0.7422
<180	20195.4 (12359.2-32999.9)	18	116.9 (66.8-204.5)	
≥180	22850.9 (15425.9-33849.8)	29	162.1 (105.1-250.0)	
ZR202-CoV				0.0567
<180	6539.3 (4121.6-10375.2)	22	37.9 (22.2-64.5)	
≥180	13208.5 (8802.3-19820.3)	25	93.7 (60.0-146.4)	
CoronaVac				0.2688
<180	1047.0 (644.7-1700.4)	20	6.1 (3.5-10.5)	
≥180	1579.8 (1064.8-2343.8)	26	11.2 (7.2-17.5)	

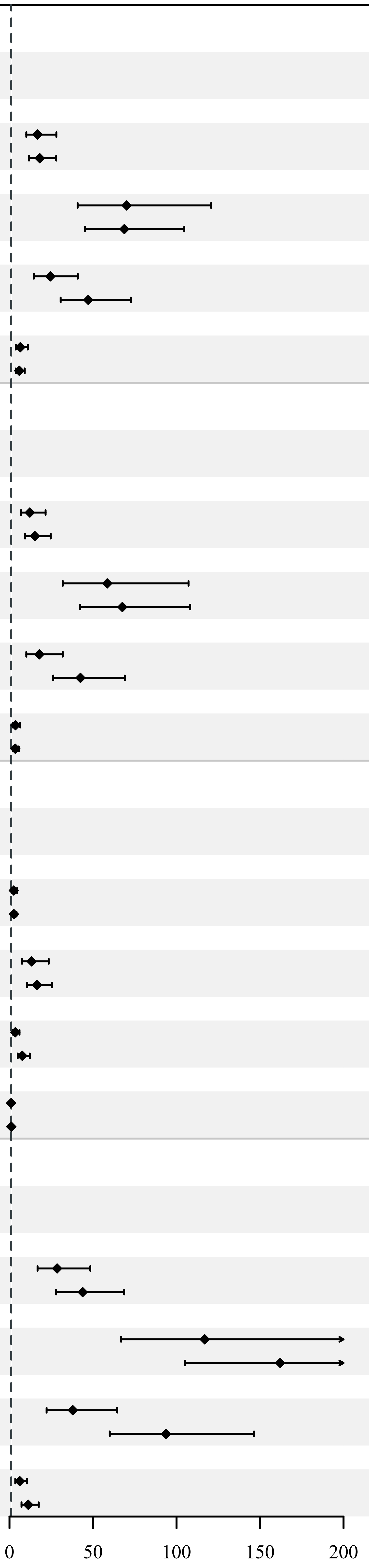

Supplementary Figure 7: Subgroup analysis of neutralising antibodies against SARS-CoV-2 at day 14 after the third dose vaccination stratified by the prime-boost interval.

Neutralising antibodies were determined with cytopathic effect (CPE)-based microneutralisation assay using authentic SARS-CoV-2 virus, including the wild-type strain (Wuhan-1, GenBank: MT123291), the delta variant (B.1.617.2, IQTC-IM2175251) and the omicron variant (BA.1.1, IQTC-Y216017) as well as with competitive inhibition method.

	Geometric mean titre (95% CI)	n	Geometric mean ratio (95% CI)	p value for interaction
**Neutralising antibody against wild-type of live SARS-CoV-2**				
Placebo				
<180	5.6 (3.7-8.5)	17	Ref	
≥180	4.8 (3.6-6.5)	29	Ref	
ChAdTS-S				0.2168
<180	355.7 (248.1-510.1)	23	63.3 (41.8-95.8)	
≥180	250.1 (182.8-342.1)	25	52.0 (36.5-74.0)	
RQ3013				0.3776
<180	1129.5 (768.4-1660.3)	18	200.9 (129.5-311.6)	
≥180	868.2 (633.1-1190.7)	29	180.5 (127.9-254.7)	
ZR202-CoV				0.1548
<180	778.7 (541.5-1119.8)	22	138.5 (91.3-210.2)	
≥180	516.0 (375.3-709.5)	25	107.3 (75.5-152.3)	
CoronaVac				0.1430
<180	99.5 (68.0-145.7)	20	17.7 (11.5-27.2)	
≥180	64.8 (47.4-88.5)	26	13.5 (9.5-19.1)	
**Neutralising antibody against the delta variant of live SARS-CoV-2**				
Placebo				
<180	4.4 (3.0-6.6)	17	Ref	
≥180	4.2 (3.2-5.7)	29	Ref	
ChAdTS-S				0.9342
<180	230.5 (162.6-326.7)	23	52.2 (35.0-77.8)	
≥180	225.3 (166.4-305.1)	25	53.1 (37.8-74.6)	
RQ3013				0.7895
<180	834.6 (574.9-1211.5)	18	189.0 (123.8-288.5)	
≥180	772.8 (569.9-1048.0)	29	182.1 (130.6-253.8)	
ZR202-CoV				0.8455
<180	477.0 (335.6-678.0)	22	108.0 (72.2-161.6)	
≥180	503.7 (370.0-685.7)	25	118.7 (84.5-166.6)	
CoronaVac				0.9786
<180	54.4 (37.6-78.7)	20	12.3 (8.2-18.6)	
≥180	54.0 (40.0-73.0)	26	12.7 (9.1-17.8)	
**Neutralising antibody against the omicron variant of live SARS-CoV-2**				
Placebo				
<180	5.1 (3.2-8.2)	17	Ref	
≥180	3.4 (2.4-4.8)	29	Ref	
ChAdTS-S				0.9064
<180	40.8 (26.9-61.9)	23	8.0 (5.0-12.9)	
≥180	42.4 (29.6-60.9)	25	12.4 (8.3-18.7)	
RQ3013				0.5429
<180	278.5 (178.6-434.4)	18	54.7 (33.0-90.4)	
≥180	226.0 (157.2-325.0)	29	66.3 (44.6-98.5)	
ZR202-CoV				0.7074
<180	111.3 (73.3-169.0)	22	21.8 (13.5-35.3)	
≥180	98.2 (68.0-141.8)	25	28.8 (19.2-43.2)	
CoronaVac				0.0911
<180	11.0 (7.1-17.1)	20	2.2 (1.3-3.5)	
≥180	6.2 (4.3-8.9)	26	1.8 (1.2-2.7)	
**Neutralising antibody measured by competitive inhibition method**				
Placebo				
<180	166.7 (103.5-268.5)	17	Ref	
≥180	136.0 (96.2-192.3)	29	Ref	
ChAdTS-S				0.5760
<180	22453.5 (14802.0-34060.2)	23	134.7 (83.4-217.5)	
≥180	18677.0 (12999.3-26834.7)	25	137.3 (91.2-206.9)	
RQ3013				0.8213
<180	98158.1 (62848.1-153306.2)	18	589.0 (354.5-978.5)	
≥180	90821.4 (63108.8-130703.4)	29	667.8 (448.3-994.8)	
ZR202-CoV				0.9115
<180	49878.8 (32805.0-75838.9)	22	299.3 (184.5-485.5)	
≥180	51763.3 (35789.0-74867.6)	25	380.6 (253.8-570.8)	
CoronaVac				0.9869
<180	4858.5 (3127.5-7547.5)	20	29.2 (17.8-47.8)	
≥180	4885.4 (3412.7-6993.6)	26	35.9 (24.0-53.9)	

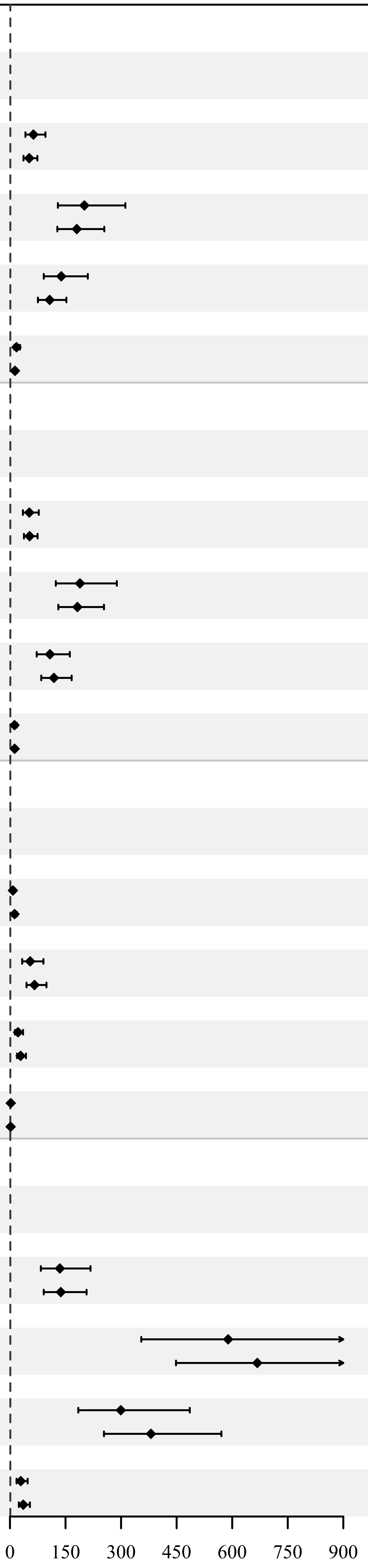

Supplementary Figure 8: Subgroup analysis of neutralising antibodies against SARS-CoV-2 at day 28 after the third dose vaccination stratified by the prime-boost interval.

Neutralising antibodies were determined with cytopathic effect (CPE)-based microneutralisation assay using authentic SARS-CoV-2 virus, including the wild-type strain (Wuhan-1, GenBank: MT123291), the delta variant (B.1.617.2, IQTC-IM2175251) and the omicron variant (BA.1.1, IQTC-Y216017) as well as with competitive inhibition method.

	Geometric mean titre (95% CI)	n	Geometric mean ratio (95% CI)	p value for interaction
**Neutralising antibody against wild-type of live SARS-CoV-2**				
Placebo				
<180	4.4 (2.9-6.5)	17	Ref	
≥180	5.8 (4.4-7.7)	29	Ref	
ChAdTS-S				0.7712
<180	340.0 (241.2-479.2)	23	78.1 (52.6-116.0)	
≥180	314.2 (233.3-423.3)	25	54.2 (38.8-75.8)	
RQ3013				0.7360
<180	685.3 (474.9-989.1)	18	157.5 (103.7-239.2)	
≥180	753.5 (561.2-1011.7)	29	130.0 (94.0-179.8)	
ZR202-CoV				0.9412
<180	633.9 (448.5-896.0)	22	145.6 (97.9-216.7)	
≥180	646.9 (477.9-875.7)	25	111.6 (79.9-155.9)	
CoronaVac				0.6976
<180	70.0 (48.7-100.6)	20	16.1 (10.7-24.2)	
≥180	62.8 (46.7-84.5)	26	10.8 (7.8-15.1)	
**Neutralising antibody against the delta variant of live SARS-CoV-2**				
Placebo				
<180	4.6 (3.0-7.0)	17	Ref	
≥180	4.4 (3.3-6.0)	29	Ref	
ChAdTS-S				0.9402
<180	248.9 (172.6-358.8)	23	54.3 (35.7-82.6)	
≥180	254.3 (185.2-349.3)	25	57.2 (40.0-81.8)	
RQ3013				0.5174
<180	658.1 (445.3-972.8)	18	143.6 (92.2-223.8)	
≥180	542.2 (396.4-741.5)	29	122.0 (86.5-172.0)	
ZR202-CoV				0.6091
<180	524.0 (362.4-757.7)	22	114.4 (75.0-174.4)	
≥180	451.0 (326.6-622.9)	25	101.5 (71.1-144.8)	
CoronaVac				0.8426
<180	55.0 (37.4-81.0)	20	12.0 (7.8-18.5)	
≥180	51.9 (37.8-71.1)	26	11.7 (8.2-16.6)	
**Neutralising antibody against the omicron variant of live SARS-CoV-2**				
Placebo				
<180	4.5 (2.8-7.2)	17	Ref	
≥180	3.7 (2.6-5.3)	29	Ref	
ChAdTS-S				0.7903
<180	34.1 (22.4-51.9)	23	7.6 (4.7-12.3)	
≥180	37.2 (25.9-53.6)	25	10.0 (6.6-15.1)	
RQ3013				0.6774
<180	164.8 (105.3-257.9)	18	36.9 (22.2-61.3)	
≥180	142.9 (99.8-204.6)	29	38.3 (25.9-56.9)	
ZR202-CoV				0.9399
<180	92.4 (60.7-140.9)	22	20.7 (12.8-33.6)	
≥180	94.8 (65.5-137.2)	25	25.4 (16.9-38.2)	
CoronaVac				0.7236
<180	6.8 (4.4-10.6)	20	1.5 (0.9-2.5)	
≥180	6.0 (4.2-8.6)	26	1.6 (1.1-2.4)	
**Neutralising antibody measured by competitive inhibition method**				
Placebo				
<180	149.1 (93.3-238.2)	17	Ref	
≥180	136.9 (97.5-192.4)	29	Ref	
ChAdTS-S				0.9132
<180	23039.5 (15298.1-34698.5)	23	154.5 (96.5-247.5)	
≥180	23865.0 (16725.8-34051.4)	25	174.3 (116.5-260.7)	
RQ3013				0.5917
<180	66666.5 (43014.8-103323.1)	18	447.2 (271.5-736.5)	
≥180	55696.7 (39224.5-79086.2)	29	406.8 (276.3-598.9)	
ZR202-CoV				0.6696
<180	54727.7 (36252.4-82618.7)	22	367.1 (228.2-590.6)	
≥180	47582.6 (33126.6-68347)	25	347.5 (233.3-517.5)	
CoronaVac				0.8159
<180	4307.2 (2794.4-6638.9)	20	28.9 (17.8-47.0)	
≥180	4652.7 (3272.2-6615.8)	26	34.0 (22.8-50.6)	

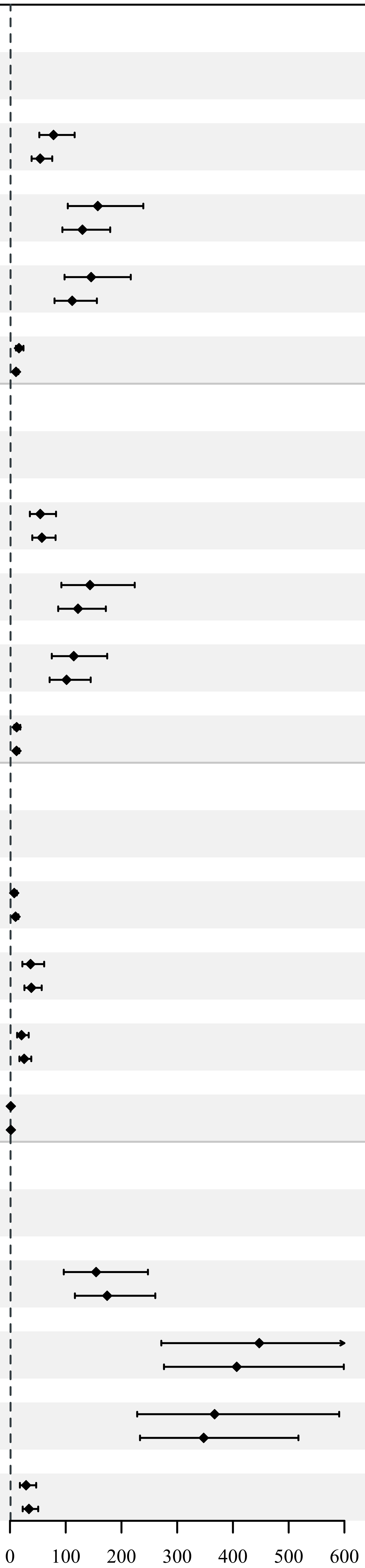

Supplementary Figure 9: Subgroup analysis of neutralising antibodies against SARS-CoV-2 at day 90 after the third dose vaccination stratified by the prime-boost interval.

Neutralising antibodies were determined with cytopathic effect (CPE)-based microneutralisation assay using authentic SARS-CoV-2 virus, including the wild-type strain (Wuhan-1, GenBank: MT123291), the delta variant (B.1.617.2, IQTC-IM2175251) and the omicron variant (BA.1.1, IQTC-Y216017) as well as with competitive inhibition method.

	Geometric mean (95% CI)	n	pvalue forinteraction	Geometric mean ratio (95% CI)
**Wild-Type**				
Placebo				
<180	4.7 (3-7.4)	17	Ref	
≥180	3.6 (2.6-5)	29	Ref	
ChAdTS-S				0.8196
<180	74.1 (49.4-111)	23	15.7 (10.0-24.6)	
≥180	79.6 (56.6-111.9)	25	22.1 (15.0-32.4)	
RQ3013				0.6207
<180	136.4 (89.1-208.9)	18	28.9 (18.0-46.5)	
≥180	116.2 (83.1-162.3)	29	32.2 (22.3-46.5)	
ZR202-CoV				0.9742
<180	177.2 (119.7-262.6)	22	37.6 (24.1-58.7)	
≥180	175.5 (123.3-249.6)	25	48.6 (32.9-72.0)	
CoronaVac				0.9237
<180	18.4 (12.1-28.0)	20	3.9 (2.4-6.2)	
≥180	19.0 (13.5-26.7)	26	5.3 (3.6-7.7)	
**Delta-Type**				
Placebo				
<180	5.2 (3.1-8.8)	17	Ref	
≥180	3.6 (2.5-5.3)	29	Ref	
ChAdTS-S				0.8100
<180	130.3 (82.1-206.8)	23	25.0 (14.8-42.3)	
≥180	119.3 (79.6-178.8)	25	33.0 (20.9-52.0)	
RQ3013				0.1645
<180	293.4 (177.2-485.6)	18	56.4 (32.1-99.1)	
≥180	171.5 (114.9-256.1)	29	47.4 (30.6-73.5)	
ZR202-CoV				0.4082
<180	350.3 (219.9-558.1)	22	67.3 (39.7-114.3)	
≥180	257.7 (171.0-388.3)	25	71.2 (45.2-112.2)	
CoronaVac				0.3490
<180	24.7 (15.2-40.2)	20	4.8 (2.7-8.2)	
≥180	17.4 (11.7-26.0)	26	4.8 (3.1-7.5)	
**Omicron-Type**				
Placebo				
<180	5.2 (3.1-8.8)	17	Ref	
≥180	3.3 (2.3-4.9)	29	Ref	
ChAdTS-S				0.7981
<180	13.3 (8.4-21.1)	23	2.6 (1.5-4.3)	
≥180	12.2 (8.1-18.2)	25	3.6 (2.3-5.7)	
RQ3013				0.1315
<180	52.4 (31.8-86.4)	18	10.1 (5.8-17.6)	
≥180	29.5 (19.9-43.7)	29	8.8 (5.7-13.6)	
ZR202-CoV				0.3508
<180	54.9 (34.7-87.0)	22	10.5 (6.2-17.8)	
≥180	38.9 (25.7-58.8)	25	11.6 (7.4-18.3)	
CoronaVac				0.2910
<180	5.9 (3.6-9.5)	20	1.1 (0.7-1.9)	
≥180	4.0 (2.7-5.9)	26	1.2 (0.8-1.9)	
**Neutralisation Antibody**				
Placebo				
<180	5.7 (2.8-11.5)	17	Ref	
≥180	4.2 (2.5-7.0)	29	Ref	
ChAdTS-S				0.0172
<180	1786.4 (970.3-3289.1)	23	312.5 (154.4-632.3)	
≥180	558.2 (325.1-958.6)	25	134.1 (72.7-247.4)	
RQ3013				0.1970
<180	1691.6 (880.1-3251.2)	18	295.9 (140.2-624.5)	
≥180	884.0 (521.3-1499.0)	29	212.4 (118.5-380.6)	
ZR202-CoV				0.3551
<180	1447.8 (783.5-2675.4)	22	253.3 (124.3-516.1)	
≥180	919.0 (530.7-1591.3)	25	220.8 (120.2-405.5)	
CoronaVac				0.0001
<180	899.2 (471.8-1714.0)	20	157.3 (76.0-325.7)	
≥180	115.7 (68.1-196.6)	26	27.8 (15.3-50.6)	

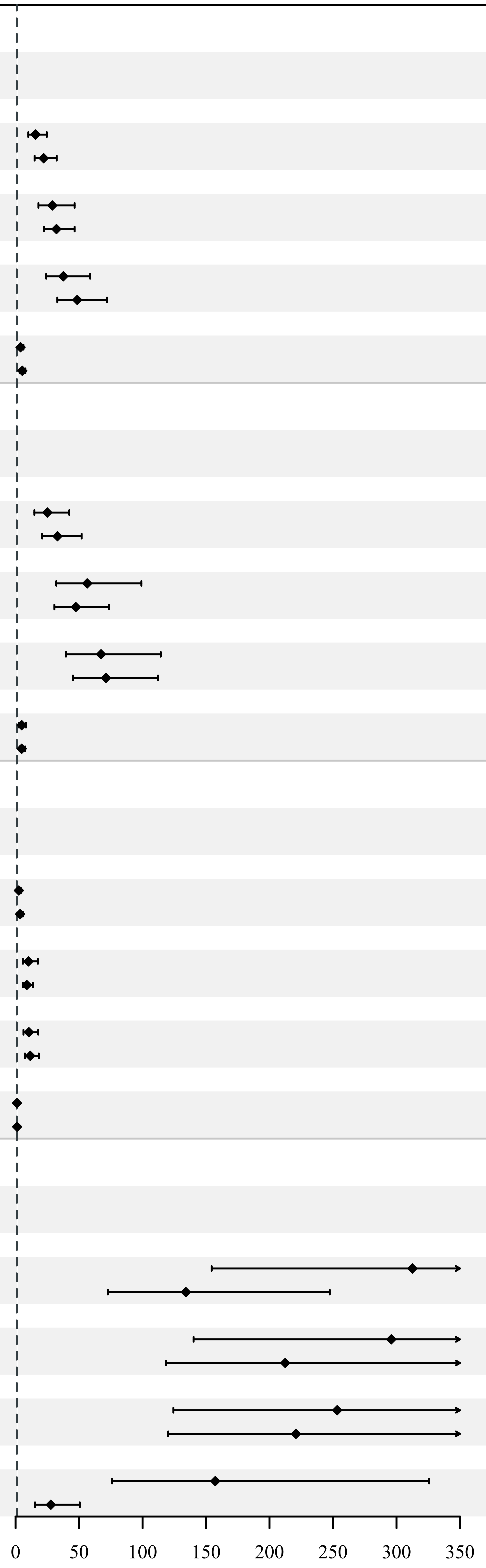

Supplementary Figure 10: Subgroup analysis of neutralising antibodies against SARS-CoV-2 at day 7 after the third dose vaccination stratified by sex.

Neutralising antibodies were determined with cytopathic effect (CPE)-based microneutralisation assay using authentic SARS-CoV-2 virus, including the wild-type strain (Wuhan-1, GenBank: MT123291), the delta variant (B.1.617.2, IQTC-IM2175251) and the omicron variant (BA.1.1, IQTC-Y216017) as well as with competitive inhibition method.

	Geometric mean (95% CI)	n	Geometric mean ratio (95% CI)	p value for interaction
**Neutralising antibody against wild-type of live SARS-CoV-2**				**1**
Placebo				
Female	5.2 (3.8-7.0)	28	Ref	
Male	4.8 (3.4-7.0)	18	Ref	
ChAdTS-S				
Female	71.7 (52.0-98.8)	24	13.9 (9.0-21.4)	0.0841
Male	107.3 (78.0-147.6)	24	22.2 (13.7-36.0)	
RQ3013				
Female	432.8 (319.5-586.5)	26	83.8 (54.6-128.6)	0.0290
Male	260.8 (186.1-365.4)	21	54.0 (32.8-88.8)	
ZR202-CoV				
Female	246.8 (182.7-333.4)	27	47.8 (31.5-72.4)	0.0006
Male	108.9 (76.8-154.3)	20	22.5 (13.7-37.2)	
CoronaVac				
Female	28.5 (20.3-40.0)	21	5.5 (3.5-8.7)	0.5300
Male	33.0 (24.2-45.0)	25	6.8 (4.2-11.0)	
**Neutralising antibody against the delta variant of live SARS-CoV-2**				
Placebo				
Female	4.6 (3.3-6.5)	28	Ref	
Male	4.1 (2.7-6.2)	18	Ref	
ChAdTS-S				
Female	62.6 (43.4-90.2)	24	13.5 (8.2-22.1)	0.8095
Male	58.7 (40.9-84.4)	24	14.3 (8.3-24.9)	
RQ3013				
Female	316.5 (223.6-448.0)	26	68.2 (42.1-110.2)	0.2838
Male	238.2 (161.8-350.7)	21	58.2 (32.9-102.9)	
ZR202-CoV				
Female	187.0 (133-263.1)	27	40.3 (25.0-64.8)	0.0005
Male	73.0 (49.1-108.7)	20	17.8 (10.1-31.6)	
CoronaVac				
Female	15.4 (10.5-22.7)	21	3.3 (2.0-5.5)	0.9865
Male	15.5 (10.9-22.0)	25	3.8 (2.2-6.5)	
**Neutralising antibody against the omicron variant of live SARS-CoV-2**				
Placebo				
Female	4.0 (3.0-5.4)	28	Ref	
Male	4.0 (2.7-5.8)	18	Ref	
ChAdTS-S				
Female	11.1 (8.0-15.4)	24	2.8 (1.8-4.3)	0.7010
Male	10.1 (7.3-14.0)	24	2.6 (1.6-4.2)	
RQ3013				
Female	76.7 (56.1-104.7)	26	19.1 (12.4-29.4)	0.0264
Male	45.1 (31.9-63.9)	21	11.4 (6.8-19.0)	
ZR202-CoV				
Female	36.6 (27.0-49.8)	27	9.1 (5.9-14.0)	<0.0001
Male	10.5 (7.3-15.0)	20	2.6 (1.6-4.4)	
CoronaVac				
Female	3.8 (2.7-5.4)	21	0.9 (0.6-1.5)	0.3056
Male	4.9 (3.6-6.7)	25	1.2 (0.8-2.0)	
**Neutralising antibody measured by competitive inhibition method**				
Placebo				
Female	145.0 (106.1-198.1)	28	Ref	
Male	163.3 (111.0-240.1)	18	Ref	
ChAdTS-S				
Female	4726.7 (3377.5-6614.7)	24	32.6 (20.6-51.5)	0.1771
Male	6569.7 (4698.7-9185.7)	24	40.2 (24.1-67.1)	
RQ3013				
Female	24203.6 (17590.5-33302.7)	26	167.0 (107.1-260.3)	0.2695
Male	18492.5 (12946.1-26415.2)	21	113.3 (66.8-192)	
ZR202-CoV				
Female	12810.3 (9323.3-17601.6)	27	88.4 (57.0-136.9)	0.0069
Male	6450.3 (4456.5-9336.2)	20	39.5 (23.2-67.3)	
CoronaVac				
Female	1168.1 (818.2-1667.7)	21	8.1 (5.0-12.9)	0.3441
Male	1474.6 (1063.0-2045.6)	25	9.0 (5.4-15.0)	

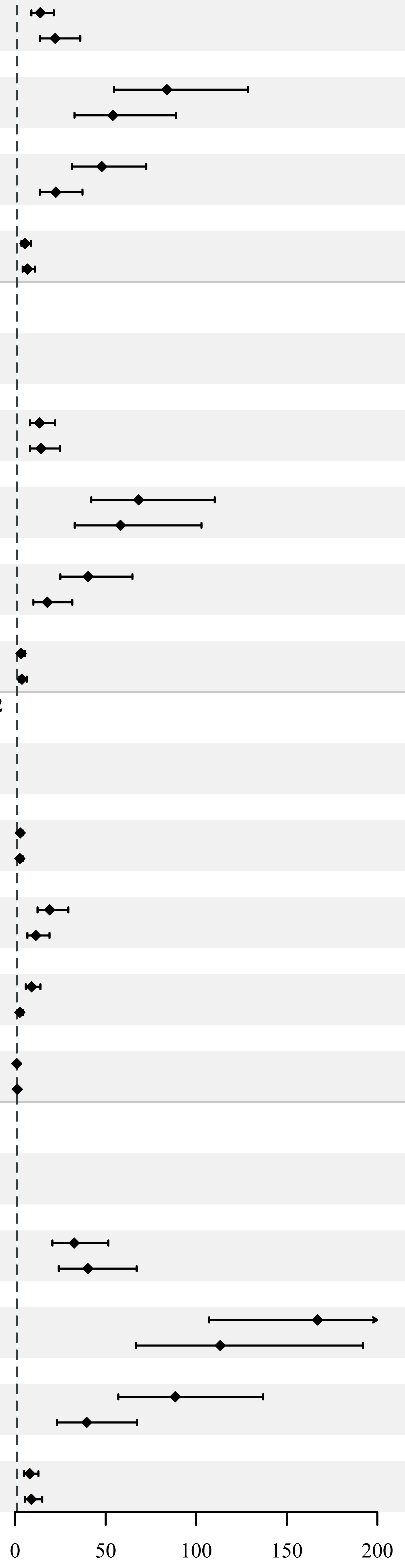

Supplementary Figure 11: Subgroup analysis of neutralising antibodies against SARS-CoV-2 at day 14 after the third dose vaccination stratified by sex.

Neutralising antibodies were determined with cytopathic effect (CPE)-based microneutralisation assay using authentic SARS-CoV-2 virus, including the wild-type strain (Wuhan-1, GenBank: MT123291), the delta variant (B.1.617.2, IQTC-IM2175251) and the omicron variant (BA.1.1, IQTC-Y216017) as well as with competitive inhibition method.

	Geometric mean titre (95% CI)	n	Geometric mean ratio (95% CI)	p value for interaction
**Neutralising antibody against wild-type of live SARS-CoV-2**				
Placebo				
Female	5.5 (4.3-7.0)	28	Ref	
Male	4.6 (3.4-6.2)	18	Ref	
ChAdTS-S				0.0040
Female	220.6 (169.6-286.8)	24	40.2 (28.2-57.4)	
Male	384.1 (295.8-498.7)	24	83.1 (56-123.4)	
RQ3013				0.7628
Female	1007.6 (781.7-1298.7)	26	183.8 (128.9-262.1)	
Male	951.3 (721.7-1254.0)	21	205.9 (136.9-309.7)	
ZR202-CoV				0.4000
Female	664.9 (519.7-850.7)	27	121.3 (86.3-170.6)	
Male	564.7 (424.3-751.5)	20	122.2 (81.1-184.2)	
CoronaVac				0.3271
Female	70.6 (53.5-93.2)	21	12.9 (8.9-18.7)	
Male	85.1 (66.0-109.7)	25	18.4 (12.5-27.3)	
**Neutralising antibody against the delta variant of live SARS-CoV-2**				
Placebo				
Female	4.5 (3.6-5.7)	28	Ref	
Male	4.0 (3.0-5.4)	18	Ref	
ChAdTS-S				0.0637
Female	191.8 (149.0-246.9)	24	42.5 (30.2-60.0)	
Male	269.2 (209.6-345.8)	24	66.9 (45.7-98.0)	
RQ3013				0.3760
Female	740.4 (579.5-946.1)	26	164.2 (117.4-229.8)	
Male	872.0 (667.4-1139.2)	21	216.7 (146.1-321.5)	
ZR202-CoV				0.4291
Female	522.4 (412.6-661.5)	27	115.9 (83.3-161.1)	
Male	451.1 (342.6-594.1)	20	112.1 (75.4-166.7)	
CoronaVac				0.0808
Female	45.5 (34.8-59.4)	21	10.1 (7.1-14.4)	
Male	62.8 (49.2-80.1)	25	15.6 (10.7-22.8)	
**Neutralising antibody against the omicron variant of live SARS-CoV-2**				
Placebo				
Female	4.0 (3.0-5.2)	28	Ref	
Male	4.0 (2.9-5.7)	18	Ref	
ChAdTS-S				0.0036
Female	29.9 (22.2-40.3)	24	7.6 (5-11.3)	
Male	56.3 (41.9-75.6)	24	14.0 (8.9-21.9)	
RQ3013				0.7940
Female	242.2 (181.4-323.3)	26	61.2 (41.1-90.9)	
Male	256.3 (187.0-351.3)	21	63.6 (39.9-101.3)	
ZR202-CoV				0.1069
Female	120.3 (91.1-158.9)	27	30.4 (20.6-44.8)	
Male	84.6 (61.2-117.0)	20	21.0 (13.2-33.5)	
CoronaVac				0.0087
Female	5.8 (4.3-8.0)	21	1.5 (1.0-2.2)	
Male	10.4 (7.8-13.8)	25	2.6 (1.6-4.0)	
**Neutralising antibody measured by competitive inhibition method**				
Placebo				
Female	144.7 (109.8-190.8)	28	Ref	
Male	146.2 (103.9-205.7)	18	Ref	
ChAdTS-S				0.0013
Female	14321.0 (10638.0-19279.0)	24	99.0 (66.0-148.3)	
Male	28941.9 (21512.6-38936.7)	24	197.9 (125.9-311.3)	
RQ3013				0.2048
Female	83116.0 (62329.5-110834.7)	26	574.3 (386.1-854.1)	
Male	109474.6 (79858.6-150073.8)	21	748.7 (469.4-1194.2)	
ZR202-CoV				0.9764
Female	50838.3 (38376.2-67347.3)	27	351.3 (238.4-517.5)	
Male	50503.8 (36412.2-70048.7)	20	345.4 (215.5-553.7)	
CoronaVac				0.0430
Female	3861.8 (2817.6-5292.8)	21	26.7 (17.5-40.6)	
Male	6012.9 (4501.2-8032.2)	25	41.1 (26.2-64.5)	

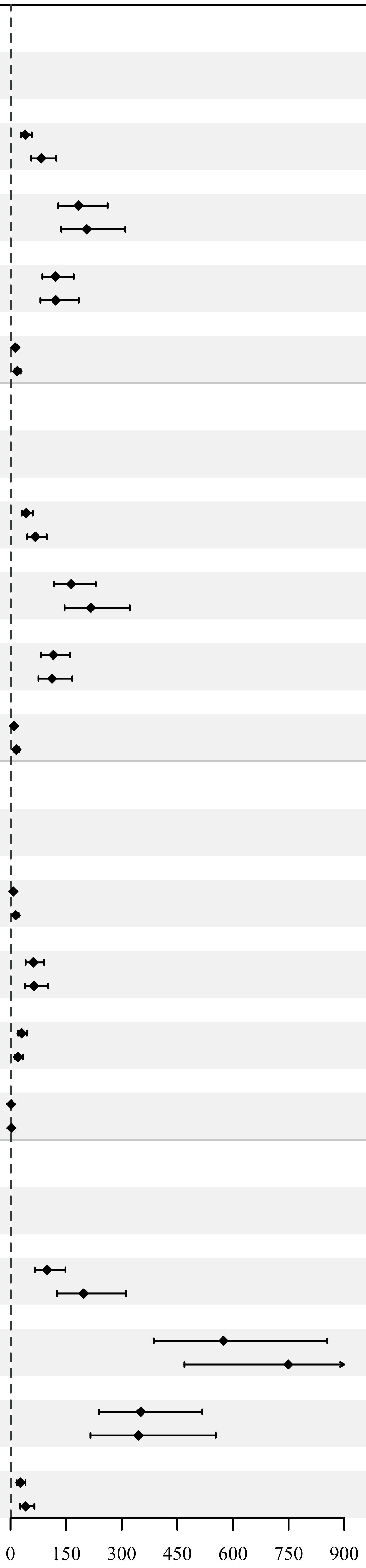

Supplementary Figure 12: Subgroup analysis of neutralising antibodies against SARS-CoV-2 at day 28 after the third dose vaccination stratified by sex.

Neutralising antibodies were determined with cytopathic effect (CPE)-based microneutralisation assay using authentic SARS-CoV-2 virus, including the wild-type strain (Wuhan-1, GenBank: MT123291), the delta variant (B.1.617.2, IQTC-IM2175251) and the omicron variant (BA.1.1, IQTC-Y216017) as well as with competitive inhibition method.

	Geometric mean (95% CI)	n	Geometric mean ratio (95% CI)	p value for interaction
**Neutralising antibody against wild-type of live SARS-CoV-2**				
Placebo				
Female	5.6 (4.4-7.1)	28	Ref	
Male	4.7 (3.5-6.2)	18	Ref	
ChAdTS-S				0.0240
Female	265.0 (205.9-341.2)	24	47.4 (33.7-66.7)	
Male	402.0 (312.8-516.6)	24	86.3 (59.1-126.2)	
RQ3013				0.8569
Female	736.4 (579.8-935.4)	26	131.7 (94.1-184.5)	
Male	712.7 (546.5-929.5)	21	153.1 (103.4-226.7)	
ZR202-CoV				0.7747
Female	655.7 (517.4-830.8)	27	117.3 (84.5-162.8)	
Male	621.6 (472.3-818.1)	20	133.5 (90.0-198.1)	
CoronaVac				0.6832
Female	63.4 (48.6-82.8)	21	11.3 (7.9-16.3)	
Male	68.3 (53.5-87.2)	25	14.7 (10.1-21.4)	
**Neutralising antibody against the delta variant of live SARS-CoV-2**				
Placebo				
Female	4.7 (3.7-6.0)	28	Ref	
Male	4.2 (3.1-5.7)	18	Ref	
ChAdTS-S				0.0241
Female	201.7 (154.9-262.8)	24	42.8 (29.9-61.3)	
Male	310.8 (239.1-403.8)	24	74.0 (49.6-110.3)	
RQ3013				0.2243
Female	528.8 (411.2-680.1)	26	112.2 (79.2-158.8)	
Male	667.8 (504.8-883.5)	21	159.0 (105.2-240.2)	
ZR202-CoV				0.7323
Female	496.7 (388.0-636.0)	27	105.3 (74.6-148.7)	
Male	464.8 (348.5-620.0)	20	110.7 (73.1-167.6)	
CoronaVac				0.1073
Female	44.9 (34.0-59.5)	21	9.5 (6.6-13.8)	
Male	61.3 (47.5-79.2)	25	14.6 (9.8-21.7)	
**Neutralising antibody against the omicron variant of live SARS-CoV-2**				
Placebo				
Female	4.0 (3.0-5.2)	28	Ref	
Male	4.0 (2.9-5.7)	18	Ref	
ChAdTS-S				0.0311
Female	28.1 (20.8-37.9)	24	7.1 (4.7-10.6)	
Male	44.8 (33.3-60.2)	24	11.1 (7.1-17.5)	
RQ3013				0.9279
Female	153.1 (115.2-203.4)	26	38.5 (26.0-57.1)	
Male	150.1 (109.4-205.9)	21	37.3 (23.4-59.5)	
ZR202-CoV				0.0259
Female	115.2 (87.1-152.2)	27	29.0 (19.6-42.8)	
Male	70.6 (51.0-97.7)	20	17.5 (11.0-28.0)	
CoronaVac				0.0112
Female	4.7 (3.4-6.4)	21	1.2 (0.8-1.8)	
Male	8.2 (6.1-10.9)	25	2.0 (1.3-3.2)	
**Neutralising antibody measured by competitive inhibition method**				
Placebo				
Female	149.6 (113.8-196.7)	28	Ref	
Male	126.6 (90.3-177.5)	18	Ref	
ChAdTS-S				0.0060
Female	17442.3 (12991.2-23418.6)	24	116.6 (78.1-174.1)	
Male	31495.2 (23476.2-42253.5)	24	248.8 (158.9-389.6)	
RQ3013				0.2178
Female	53345.5 (40326.4-70567.9)	26	356.5 (241.5-526.2)	
Male	69425.6 (50787.7-94903.3)	21	548.4 (345.3-870.9)	
ZR202-CoV				0.5261
Female	47723.0 (36120.3-63052.7)	27	318.9 (217.3-468.2)	
Male	54898.9 (39698.6-75919.2)	20	433.6 (271.7-692.1)	
CoronaVac				0.1101
Female	3752.6 (2746.4-5127.5)	21	25.1 (16.5-38.0)	
Male	5302.5 (3979.9-7064.6)	25	41.9 (26.8-65.4)	

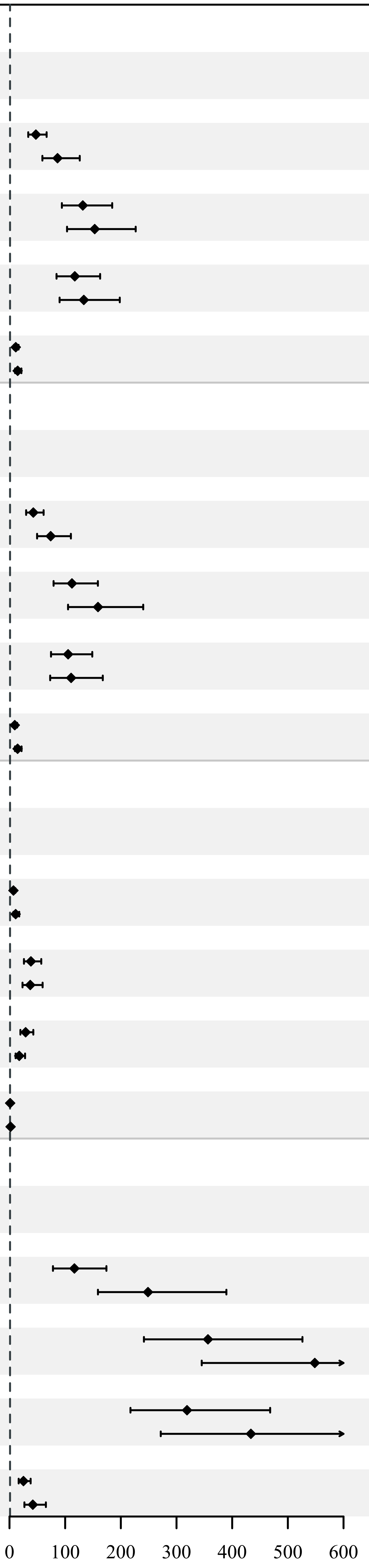

Supplementary Figure 13: Subgroup analysis of neutralising antibodies against SARS-CoV-2 at day 90 after the third dose vaccination stratified by sex.

Neutralising antibodies were determined with cytopathic effect (CPE)-based microneutralisation assay using authentic SARS-CoV-2 virus, including the wild-type strain (Wuhan-1, GenBank: MT123291), the delta variant (B.1.617.2, IQTC-IM2175251) and the omicron variant (BA.1.1, IQTC-Y216017) as well as with competitive inhibition method.

	Geometric mean (95% CI)	n	Geometric mean ratio (95% CI)	p value for interaction
**Wild-Type**				
Placebo				
Female	3.7 (2.9-4.9)	28	Ref	
Male	4.4 (3.2-6.1)	18	Ref	
ChAdTS-S				0.0087
Female	57.5 (42.8-77.1)	24	15.4 (10.4-22.7)	
Male	100.8 (75.7-134.1)	24	22.8 (14.8-35.2)	
RQ3013				0.6179
Female	194.8 (148.5-255.5)	26	31.8 (21.9-46.2)	
Male	152.7 (111.6-209.0)	21	29.8 (19.0-46.6)	
ZR202-CoV				0.2530
Female	118.7 (91.0-154.8)	27	52.2 (36.0-75.7)	
Male	131.4 (97.2-177.7)	20	34.6 (22.1-54.3)	
CoronaVac				0.8811
Female	18.5 (13.6-25.3)	21	5.0 (3.3-7.5)	
Male	19.1 (14.5-25.2)	25	4.3 (2.8-6.7)	
**Delta-Type**				
Placebo				
Female	4.2 (3.1-5.7)	28	Ref	
Male	4.1 (2.8-6.2)	18	Ref	
ChAdTS-S				0.2651
Female	106.8 (76.2-149.6)	24	25.4 (16.1-40.2)	
Male	140.6 (99.8-197.9)	24	33.9 (20.1-57.2)	
RQ3013				0.2778
Female	191.2 (138.7-263.6)	26	45.5 (29.2-71.0)	
Male	251.2 (172.7-365.5)	21	60.6 (35.0-104.8)	
ZR202-CoV				0.5600
Female	313.4 (228.6-429.5)	27	74.6 (48.1-115.8)	
Male	270.6 (185.4-394.8)	20	65.2 (37.9-112.4)	
CoronaVac				0.5707
Female	18.8 (13.2-26.9)	21	4.5 (2.8-7.2)	
Male	21.7 (15.5-30.2)	25	5.2 (3.1-8.8)	
**Omicron-Type**				
Placebo				
Female	4.0 (2.9-5.4)	28	Ref	
Male	4.0 (2.7-6.0)	18	Ref	
ChAdTS-S				0.0389
Female	9.7 (7.0-13.5)	24	2.5 (1.6-3.9)	
Male	16.1 (11.5-22.5)	24	4.0 (2.4-6.6)	
RQ3013				0.9222
Female	37.1 (27.1-50.8)	26	9.4 (6.0-14.5)	
Male	38.0 (26.5-54.4)	21	9.4 (5.5-16.0)	
ZR202-CoV				0.1392
Female	52.9 (38.6-72.5)	27	13.3 (8.6-20.7)	
Male	36.6 (25.2-53.1)	20	9.1 (5.3-15.5)	
CoronaVac				0.2111
Female	4.0 (2.8-5.7)	21	1.0 (0.6-1.6)	
Male	5.4 (3.9-7.5)	25	1.3 (0.8-2.2)	
**Neutralisation Antibody**				
Placebo				
Female	5.9 (3.8-9.1)	28	Ref	
Male	3.4 (2.0-5.9)	18	Ref	
ChAdTS-S				0.5534
Female	853.2 (533.0-1365.7)	24	145.4 (76.6-275.8)	
Male	1046.6 (648.1-1690.3)	24	307.2 (147.7-638.8)	
RQ3013				0.6462
Female	1127.1 (720.9-1762.2)	26	192.1 (103.1-357.6)	
Male	1318 (800.0-2171.3)	21	386.8 (182.7-818.7)	
ZR202-CoV				0.4666
Female	994.9 (637.6-1552.4)	27	169.5 (91.8-312.9)	
Male	1291.2 (759.2-2196.1)	20	378.9 (176.4-813.9)	
CoronaVac				0.1577
Female	365.2 (221.8-601.2)	21	62.2 (32-120.9)	
Male	224.2 (141.8-354.6)	25	65.8 (31.9-135.6)	

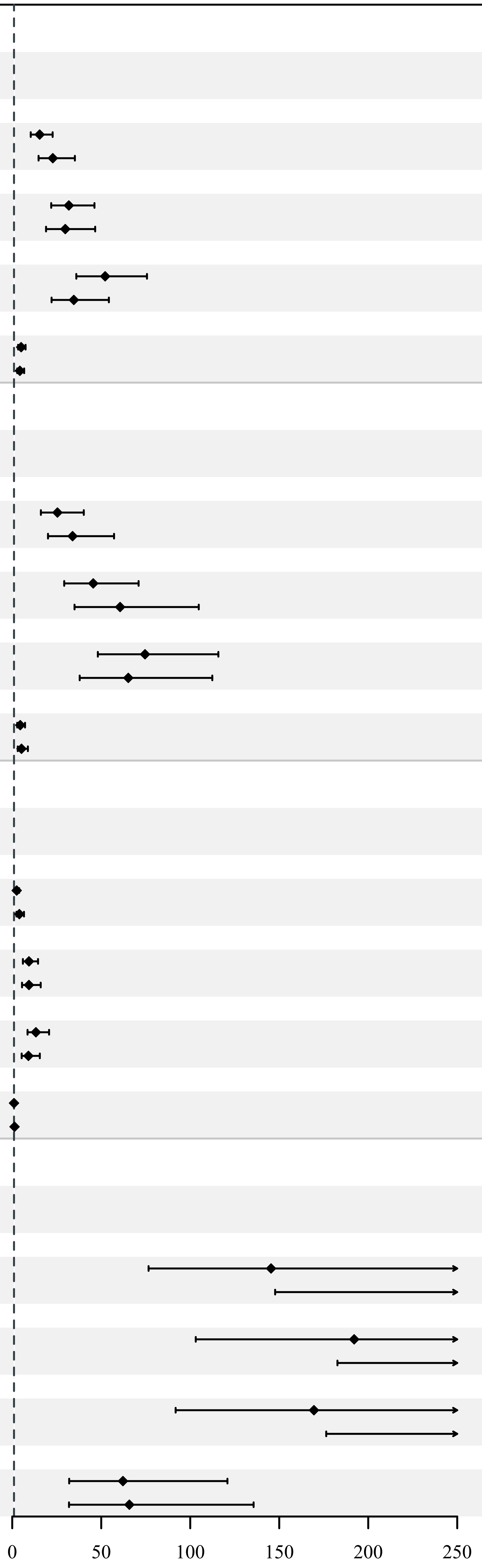

Supplementary Figure 14: Subgroup analysis of T cell responses against wild-type of SARS-CoV-2 Spike protein at day 7 after the third dose vaccination stratified by the prime-boost interval.

The interferon (IFN)-γ, interleukin (IL)-4 and granzyme B-secreting T cells after stimulating peripheral blood mononuclear cells (PBMCs) with peptides of whole Spike protein epitopes designed based on the wild-type of SARS-CoV-2 were measured by FluoroSpot assay.

	n	Median(IQR)	p value
**IFN-γ⁺ SFC per million PBMCs against wild-type of SARS-CoV-2**			
Placebo			0.8906
<180	14	396.0 (118.0,892.0)	
≥180	27	272.0 (126.0,574.0)	
ChAdTS-S			0.5587
<180	20	1298.0 (603.0,1557.0)	
≥180	18	1150.0 (570.0,1434.0)	
RQ3013			0.8541
<180	18	2384.0 (837.0,3392.0)	
≥180	23	2404.0 (1634.0,3008.0)	
ZR202-CoV			0.8783
<180	18	1054.0 (436.0,1543.0)	
≥180	21	932.0 (524.0,1540.0)	
CoronaVac			0.8540
<180	18	308.0 (173.0,993.0)	
≥180	23	352.0 (164.0,668.0)	
**Granzyme B⁺ SFC per million PBMCs against wild-type of SARS-CoV-2**			
Placebo			0.3477
<180	13	888.0 (140.0,1188.0)	
≥180	22	374.0 (145.0,632.0)	
ChAdTS-S			0.7588
<180	18	1072.0 (475.0,1359.0)	
≥180	20	846.0 (439.0,1342.0)	
RQ3013			0.7986
<180	14	1518.0 (966.0,3275.0)	
≥180	26	1898.0 (1092.0,2214.0)	
ZR202-CoV			0.1052
<180	18	1084.0 (604.0,1447.0)	
≥180	21	580.0 (288.0,1204.0)	
CoronaVac			0.9646
<180	16	364.0 (191.0,1309.0)	
≥180	22	432.0 (168.0,704.0)	
**IL-4⁺ SFC per million PBMCs against wild-type of SARS-CoV-2**			
Placebo			0.0307
<180	17	40.0 (0.0,96.0)	
≥180	28	0.0 (0.0,31.0)	
ChAdTS-S			0.0430
<180	21	132.0 (44.0,172.0)	
≥180	25	60.0 (20.0,112.0)	
RQ3013			0.9190
<180	18	432.0 (142.0,607.0)	
≥180	24	374.0 (103.0,650.0)	
ZR202-CoV			0.0364
<180	20	130.0 (40.0,257.0)	
≥180	24	46.0 (15.0,98.0)	
CoronaVac			0.0224
<180	20	32.0 (0.0,102.0)	
≥180	23	0.0 (0.0,24.0)	

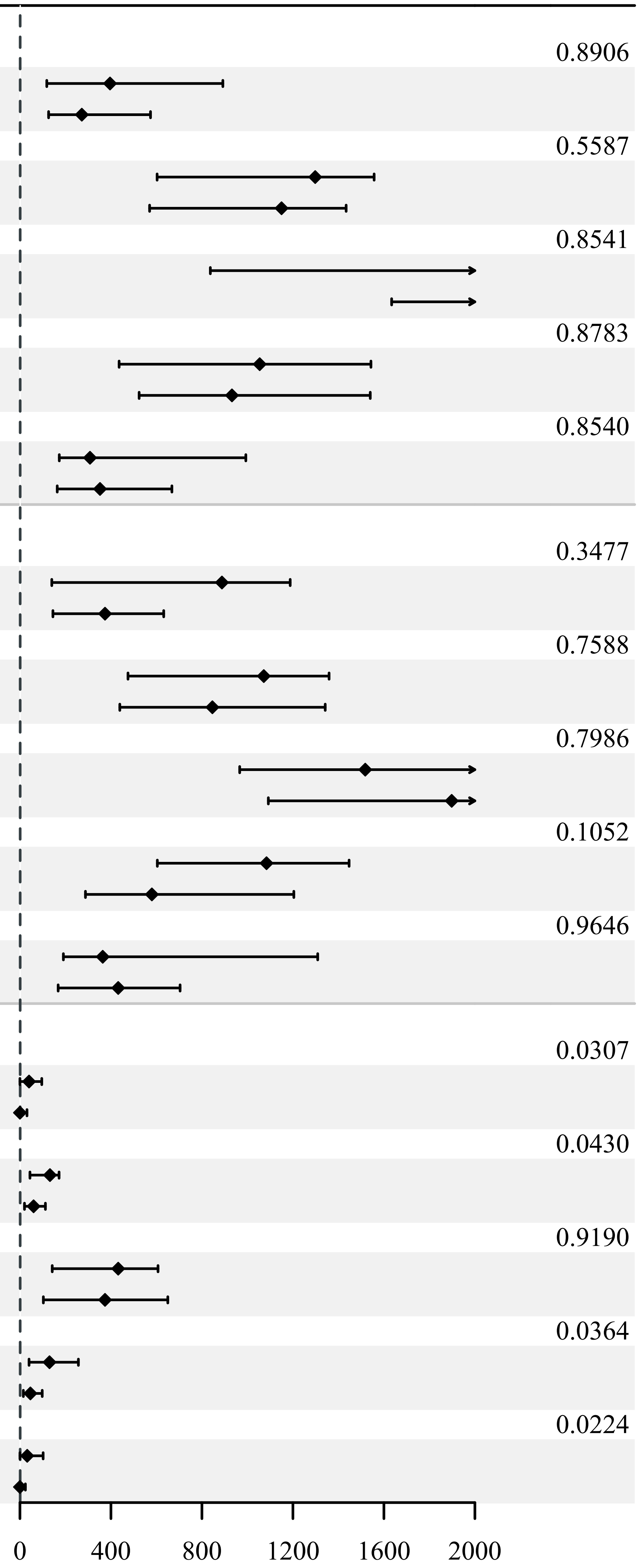

Supplementary Figure 15: Subgroup analysis of T cell responses against wild-type of SARS-CoV-2 Spike protein at day 14 after the third dose vaccination stratified by the prime-boost interval.

The interferon (IFN)-γ, interleukin (IL)-4 and granzyme B-secreting T cells after stimulating peripheral blood mononuclear cells (PBMCs) with peptides of whole Spike protein epitopes designed based on the wild-type of SARS-CoV-2 were measured by FluoroSpot assay.

	n	Median(IQR)	p value
**IFN-γ⁺ SFC per million PBMCs against wild-type of SARS-CoV-2**			
Placebo			0.6434
<180	15	464.0 (234.0,890.0)	
≥180	23	376.0 (124.0,828.0)	
ChAdTS-S			0.7737
<180	19	832.0 (380.0,1414.0)	
≥180	22	954.0 (693.0,1084.0	
RQ3013			0.462
<180	18	1076.0 (424.0,2435.0)	
≥180	23	1304.0 (974.0,2488.0)	
ZR202-CoV			0.8769
<180	18	1242.0 (345.0,1833.0)	
≥180	21	664.0 (540.0,1464.0)	
ZR202-CoV			0.8528
<180	16	524.0 (237.0,947.0)	
≥180	23	472.0 (212.0,956.0)	
**Granzyme B⁺ SFC per million PBMCs against wild-type of SARS-CoV-2**			
Placebo			0.1436
<180	12	762.0 (315.0,948.0)	
≥180	20	246.0 (42.0,938.0)	
ChAdTS-S			0.2457
<180	13	1404.0 (748.0,1548.0)	
≥180	22	880.0 (596.0,1316.0)	
RQ3013			0.4789
<180	17	1384.0 (572.0,2804.0)	
≥180	22	1170.0 (633.0,1630.0)	
ZR202-CoV			0.9878
<180	15	1004.0 (360.0,2134.0)	
≥180	22	944.0 (557.0,1649.0)	
CoronaVac			0.8387
<180	14	472.0 (160.0,1290.0)	
≥180	23	564.0 (180.0,1094.0)	
**IL-4⁺ SFC per million PBMCs against wild-type of SARS-CoV-2**			
Placebo			0.0663
<180	17	36.0 (0.0,72.0)	
≥180	28	0.0 (0.0,25.0)	
ChAdTS-S			0.0918
<180	22	90.0 (33.0,145.0)	
≥180	25	36.0 (0.0,80.0)	
RQ3013			0.7194
<180	18	170.0 (80.0,280.0)	
≥180	27	168.0 (78.0,368.0)	
ZR202-CoV			0.4852
<180	22	84.0 (45.0,160.0)	
≥180	23	72.0 (0.0,132.0)	
CoronaVac			0.2573
<180	19	60.0 (26.0,100.0)	
≥180	24	32.0 (0.0,104.0)	

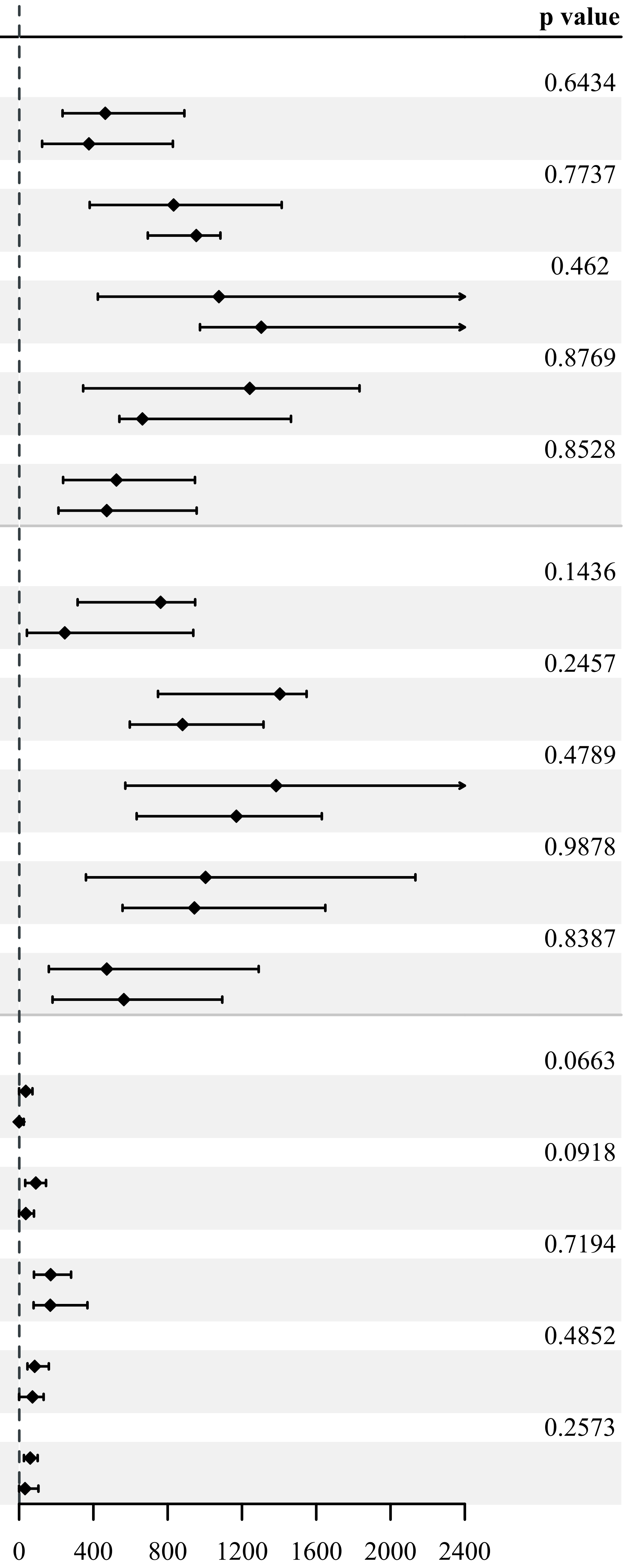

Supplementary Figure 16: Subgroup analysis of T cell responses against wild-type of SARS-CoV-2 Spike protein at day 28 after the third dose vaccination stratified by the prime-boost interval.

The interferon (IFN)-γ, interleukin (IL)-4 and granzyme B-secreting T cells after stimulating peripheral blood mononuclear cells (PBMCs) with peptides of whole Spike protein epitopes designed based on the wild-type of SARS-CoV-2 were measured by FluoroSpot assay.

	n	Median(IQR)	p value
**IFN-γ$^+$ SFC per million PBMCs against wild-type of SARS-CoV-2**			
Placebo			0.3240
<180	10	206.0 (79.0,643.0)	
≥180	11	508.0 (222.0,1206.0)	
ChAdTS-S			0.7987
<180	12	366.0 (270.0,641.0)	
≥180	12	354.0 (216.0,932.0)	
RQ3013			0.3410
<180	12	474.0 (214.0,1074.0)	
≥180	17	680.0 (504.0,1052.0)	
ZR202-CoV			0.5512
<180	12	340.0 (203.0,795.0)	
≥180	12	466.0 (346.0,768.0)	
ZR202-CoV			0.8767
<180	10	262.0 (197.0,372.0)	
≥180	13	156.0 (100.0,748.0)	
**Granzyme B$^+$ SFC per million PBMCs against wild-type of SARS-CoV-2**			
Placebo			0.8870
<180	9	440.0 (136.0,772.0)	
≥180	16	294.0 (120.0,763.0)	
ChAdTS-S			0.6485
<180	10	278.0 (93.0,491.0)	
≥180	18	384.0 (203.0,573.0)	
RQ3013			0.8046
<180	11	428.0 (248.0,806.0)	
≥180	18	556.0 (210.0,1006.0)	
ZR202-CoV			0.6140
<180	13	428.0 (164.0,760.0)	
≥180	16	646.0 (327.0,864.0)	
CoronaVac			0.9585
<180	11	204.0 (90.0,372.0)	
≥180	15	216.0 (60.0,474.0)	
**IL-4$^+$ SFC per million PBMCs against wild-type of SARS-CoV-2**			
Placebo			0.0615
<180	15	32.0 (0.0,62.0)	
≥180	21	0.0 (0.0,28.0)	
ChAdTS-S			0.0301
<180	16	44.0 (0.0,99.0)	
≥180	16	0.0 (0.0,29.0)	
RQ3013			0.7269
<180	15	72.0 (34.0,156.0)	
≥180	19	92.0 (10.0,132.0)	
ZR202-CoV			0.7867
<180	16	32.0 (0.0,53.0)	
≥180	19	36.0 (0.0,56.0)	
CoronaVac			0.0186
<180	15	40.0 (10.0,88.0)	
≥180	17	0.0 (0.0,28.0)	

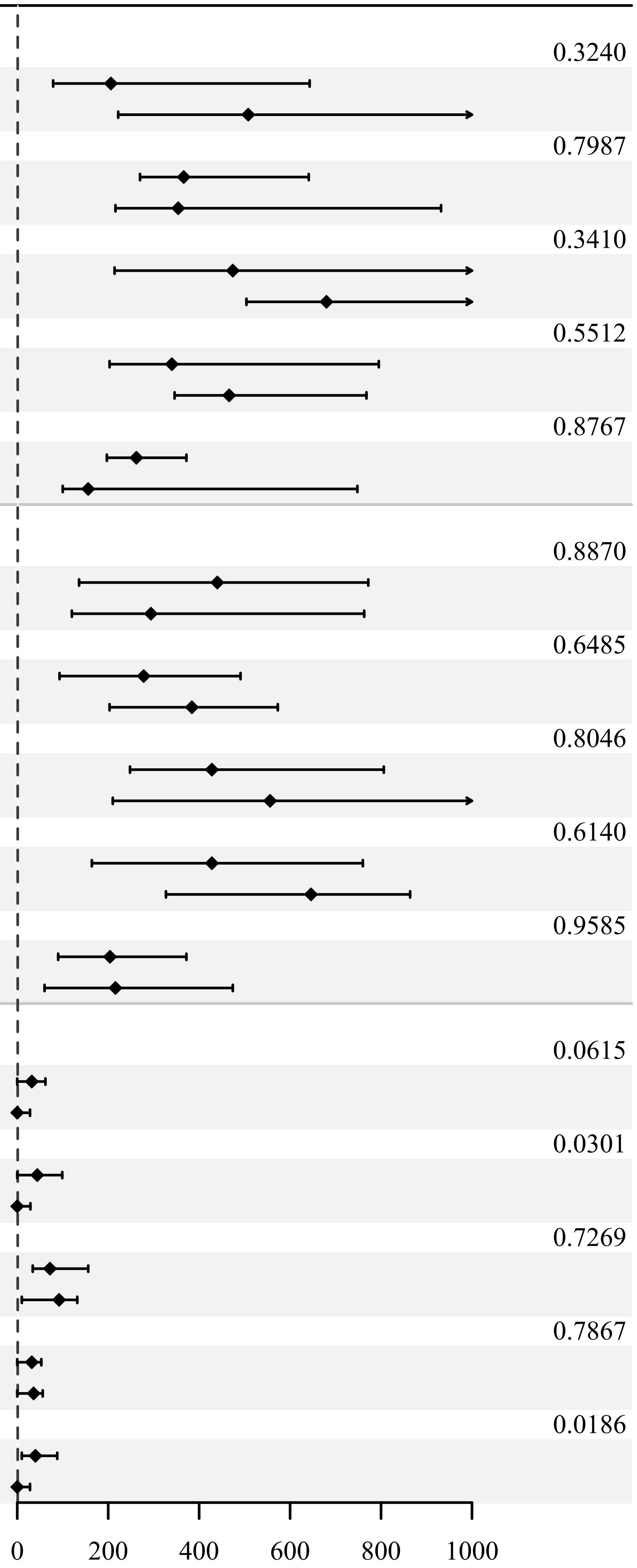

Supplementary Figure 17: Subgroup analysis of T cell responses against wild-type of SARS-CoV-2 Spike protein at day 7 after the third dose vaccination stratified by sex.

The interferon (IFN)-γ, interleukin (IL)-4 and granzyme B-secreting T cells after stimulating peripheral blood mononuclear cells (PBMCs) with peptides of whole Spike protein epitopes designed based on the wild-type of SARS-CoV-2 were measured by FluoroSpot assay.

	n	Median(IQR)	p value
**IFN-γ^+ SFC per million PBMCs against wild-type of SARS-CoV-2**			
Placebo			0.5037
Female	25	344.0 (148.0,856.0)	
Male	16	232.0 (99.0,712.0)	
ChAdTS-S			0.4385
Female	20	1212.0 (630.0,1693.0)	
Male	18	1162.0 (511.0,1441.0)	
RQ3013			0.1361
Female	22	2468.0 (2024.0,3372.0)	
Male	19	2044.0 (1110.0,2898.0)	
ZR202-CoV			0.8307
Female	24	1018.0 (512.0,1670.0)	
Male	15	960.0 (670.0,1502.0)	
CoronaVac			0.0483
Female	19	272.0 (78.0,506.0)	
Male	22	436.0 (209.0,1244.0)	
**Granzyme B$^+$ SFC per million PBMCs against wild-type of SARS-CoV-2**			
Placebo			0.8295
Female	19	412.0 (144.0,1036.0)	
Male	16	544.0 (125.0,963.0)	
ChAdTS-S			1.0000
Female	20	934.0 (442.0,1422.0)	
Male	18	1040.0 (508.0,1329.0)	
RQ3013			0.2132
Female	23	2044.0 (1268.0,2722.0)	
Male	17	1504.0 (948.0,2136.0)	
ZR202-CoV			0.2790
Female	24	754.0 (249.0,1285.0)	
Male	15	912.0 (590.0,1426.0)	
CoronaVac			0.0111
Female	15	220.0 (50.0,420.0)	
Male	23	508.0 (292.0,1208.0)	
**IL-4$^+$ SFC per million PBMCs against wild-type of SARS-CoV-2**			
Placebo			0.3527
Female	27	0.0 (0.0,40.0)	
Male	18	10.0 (0.0,113.0)	
ChAdTS-S			0.6354
Female	24	66.0 (35.0,123.0)	
Male	22	120.0 (26.0,170.0)	
RQ3013			0.7046
Female	23	368.0 (118.0,590.0)	
Male	19	404.0 (140.0,712.0)	
ZR202-CoV			0.8183
Female	27	72.0 (26.0,144.0)	
Male	17	60.0 (24.0,188.0)	
CoronaVac			0.1389
Female	20	0.0 (0.0,32.0)	
Male	23	24.0 (0.0,64.0)	

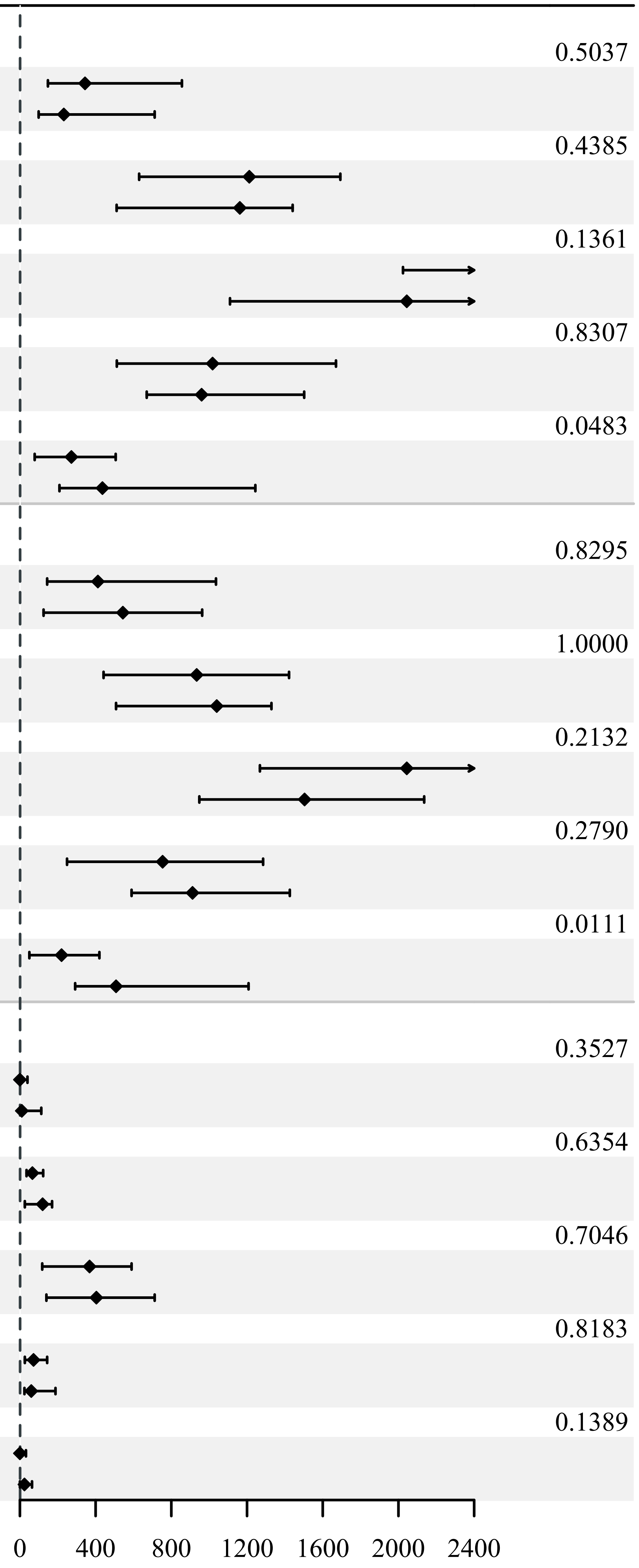

Supplementary Figure 18: Subgroup analysis of T cell responses against wild type of SARS-CoV-2 Spike protein at day 14 after the third dose vaccination stratified by sex.

The interferon (IFN)-γ, interleukin (IL)-4 and granzyme B-secreting T cells after stimulating peripheral blood mononuclear cells (PBMCs) with peptides of whole Spike protein epitopes designed based on the wild-type of SARS-CoV-2 were measured by FluoroSpot assay.

	n	Median(IQR)	p value
**IFN-γ^+ SFC per million PBMCs against wild-type of SARS-CoV-2**			
Placebo			0.3702
Female	23	536.0 (222.0,976.0)	
Male	15	356.0 (148.0,648.0)	
ChAdTS-S			0.7143
Female	22	922.0 (591.0,1279.0)	
Male	19	904.0 (656.0,1224.0)	
RQ3013			0.046
Female	20	1608.0 (1037.0,2630.0)	
Male	21		
ZR202-CoV			0.2475
Female	23	1216.0 (552.0,1836.0)	
Male	16	580.0 (393.0,1440.0)	
CoronaVac			0.4639
Female	18	656.0 (303.0,951.0)	
Male	21	364.0 (172.0,1092.0)	
**Granzyme B^+ SFC per million PBMCs against wild-type of SARS-CoV-2**			
Placebo			0.5201
Female	17	284.0 (56.0,936.0)	
Male	15	340.0 (220.0,932.0)	
ChAdTS-S			0.6679
Female	18	1016.0 (509.0,1614.0)	
Male	17	1092.0 (748.0,1332.0)	
RQ3013			0.1189
Female	20	1474.0 (738.0,2504.0)	
Male	19	1152.0 (400.0,1588.0)	
ZR202-CoV			0.4171
Female	22	1126.0 (474.0,1775.0)	
Male	15	596.0 (380.0,1574.0)	
CoronaVac			0.7220
Female	15	732.0 (246.0,1094.0)	
Male	22	388.0 (170.0,1290.0)	
**IL-4^+ SFC per million PBMCs against wild-type of SARS-CoV-2**			
Placebo			0.4969
Female	28	0.0 (0.0,37.0)	
Male	17	24.0 (0.0,60.0)	
ChAdTS-S			0.5971
Female	24	60.0 (0.0,106.0)	
Male	23	56.0 (12.0,124.0)	
RQ3013			0.8199
Female	24	160.0 (71.0,292.0)	
Male	21	168.0 (100.0,344.0)	
ZR202-CoV			0.1179
Female	26	88.0 (47.0,195.0)	
Male	19	64.0 (0.0,96.0)	
CoronaVac			0.6239
Female	20	56.0 (24.0,105.0)	
Male	23	56.0 (10.0,100.0)	

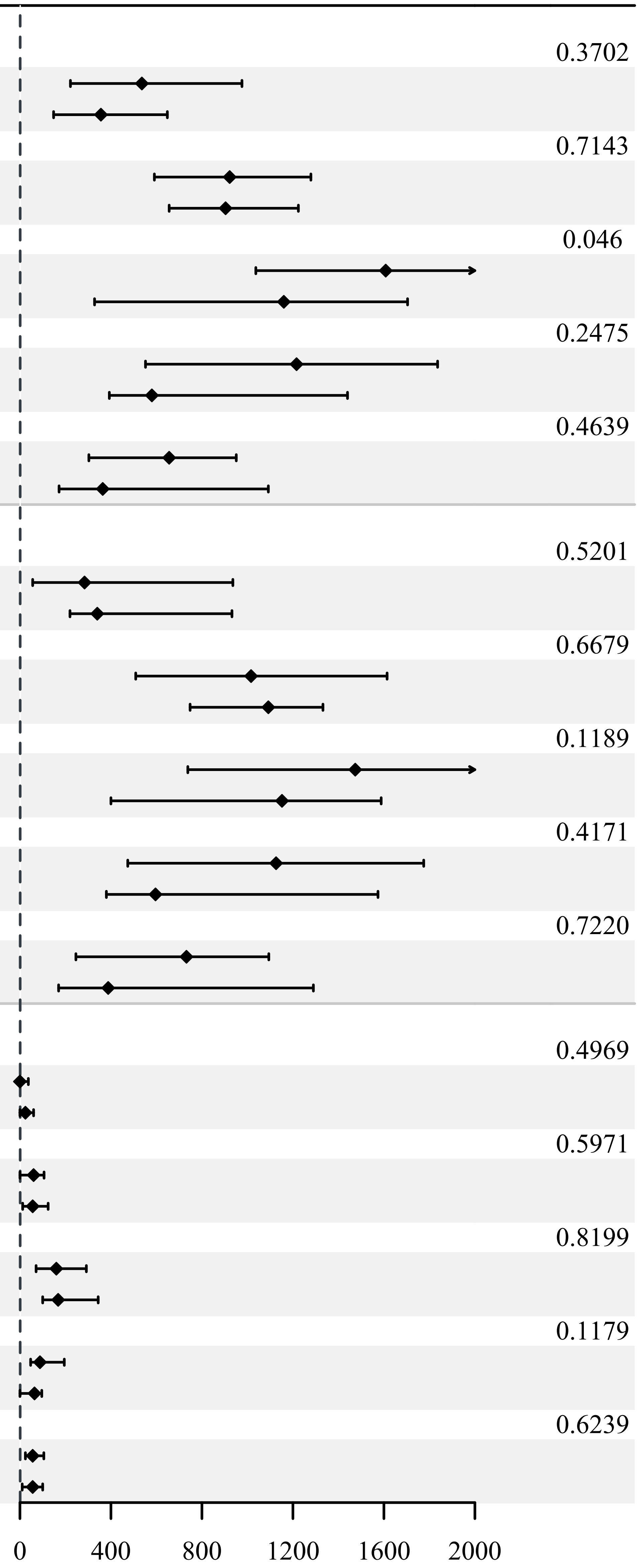

Supplementary Figure 19: Subgroup analysis of T cell responses against wild-type of SARS-CoV-2 Spike protein at day 28 after the third dose vaccination stratified by sex.

The interferon (IFN)-γ, interleukin (IL)-4 and granzyme B-secreting T cells after stimulating peripheral blood mononuclear cells (PBMCs) with peptides of whole Spike protein epitopes designed based on the wild-type of SARS-CoV-2 were measured by FluoroSpot assay.

	n	Median(IQR)	p value
**IFN-γ$^+$ SFC per million PBMCs against wild-type of SARS-CoV-2**			
Placebo			0.1551
Female	12	700.0 (163.0,1699.0)	
Male	9	256.0 (112.0,508.0)	
ChAdTS-S			0.0410
Female	11	284.0 (210.0,366.0)	
Male	13	728.0 (340.0,968.0)	
RQ3013			0.0016
Female	16	998.0 (602.0,1437.0)	
Male	13	320.0 (172.0,508.0)	
ZR202-CoV			0.1005
Female	12	618.0 (379.0,1076.0)	
Male	12	332.0 (218.0,497.0)	
CoronaVac			0.1134
Female	8	168.0 (72.0,264.0)	
Male	15	356.0 (110.0,732.0)	
**Granzyme B$^+$ SFC per million PBMCs against wild-type of SARS-CoV-2**			
Placebo			0.2404
Female	12	542.0 (186.0,993.0)	
Male	13	140.0 (128.0,752.0)	
ChAdTS-S			0.4904
Female	14	302.0 (111.0,463.0)	
Male	14	384.0 (140.0,598.0)	
RQ3013			0.5684
Female	16	606.0 (200.0,1174.0)	
Male	13	436.0 (208.0,564.0)	
ZR202-CoV			0.1478
Female	16	704.0 (414.0,864.0)	
Male	13	328.0 (164.0,672.0)	
CoronaVac			0.0344
Female	10	70.0 (0.0,193.0)	
Male	16	262.0 (172.0,504.0)	
**IL-4$^+$ SFC per million PBMCs against wild-type of SARS-CoV-2**			
Placebo			0.6068
Female	21	0.0 (0.0,28.0)	
Male	15	20.0 (0.0,42.0)	
ChAdTS-S			0.2475
Female	15	0.0 (0.0,44.0)	
Male	17	28.0 (0.0,64.0)	
RQ3013			0.4848
Female	19	100.0 (26.0,180.0)	
Male	15	72.0 (14.0,116.0)	
ZR202-CoV			0.0987
Female	20	44.0 (0.0,73.0)	
Male	15	20.0 (0.0,40.0)	
CoronaVac			0.4977
Female	15	32.0 (0.0,44.0)	
Male	17	0.0 (0.0,52.0)	

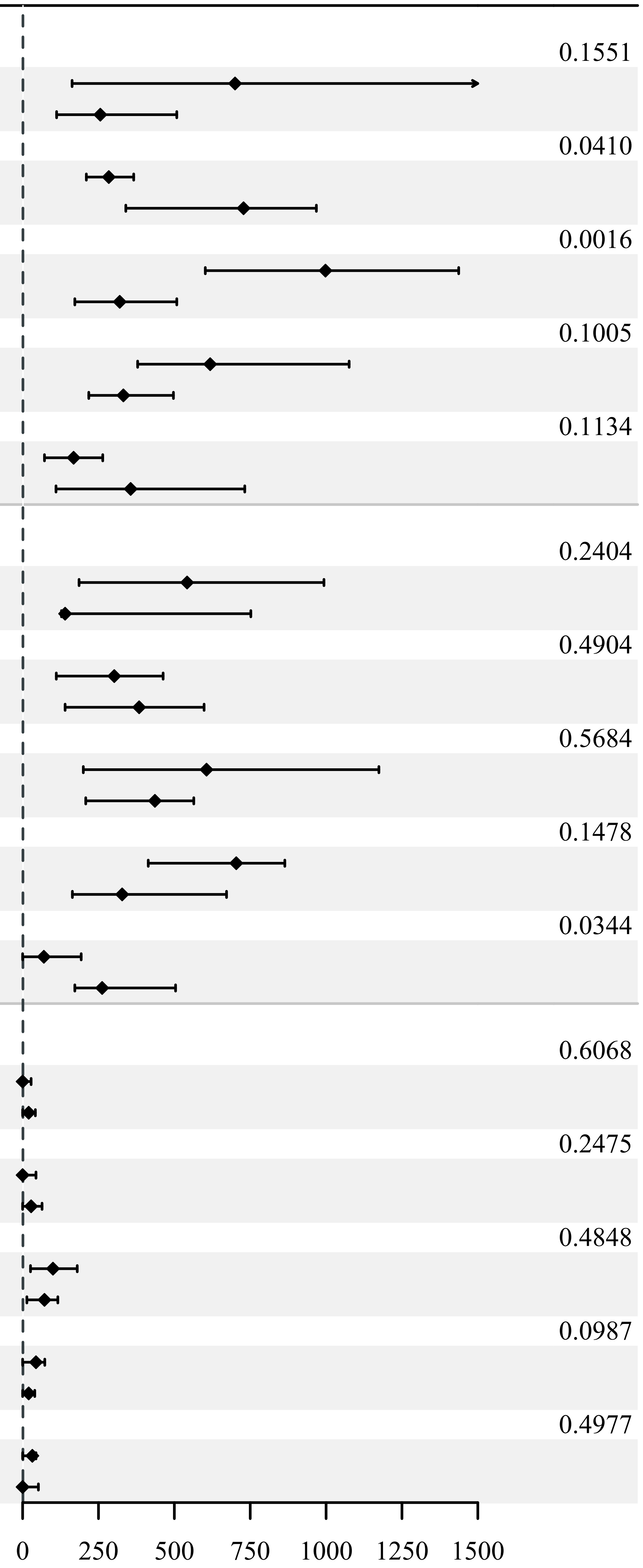

Supplementary Figure 20: Subgroup analysis of T cell responses against omicron variant of SARS-CoV-2 Spike protein at day 7 after the third dose vaccination stratified by sex.

The interferon (IFN)-γ, interleukin (IL)-4 and granzyme B-secreting T cells after stimulating peripheral blood mononuclear cells (PBMCs) with peptides of whole Spike protein epitopes designed based on the omicron variant (B.1.1.529) of SARS-CoV-2 were measured by FluoroSpot assay. T cell responses against omicron variant of SARS-CoV-2 were determined in samples from a random subset of the 234 participants (n=23 for ChAdTS-S, n=21 for RQ3013, n=22 for ZR202-CoV, n=21 for CoronaVac, and n=21 for placebo).

	n	Median(IQR)	p value
**IFN-γ⁺ SFC per million PBMCs against omicron variant of SARS-CoV-2**			
Placebo			0.7618
Female	8	764.0 (476.0,1342.0)	
Male	10	880.0 (383.0,1210.0)	
ChAdTS-S			0.0659
Female	11	1628.0 (1402.0,2178.0)	
Male	11	940.0 (700.0,1376.0)	
RQ3013			0.1487
Female	10	2746.0 (1650.0,2952.0)	
Male	11	2388.0 (660.0,2552.0)	
ZR202-CoV			0.0434
Female	8	1680.0 (1317.0,2112.0)	
Male	10	602.0 (358.0,898.0)	
CoronaVac			0.5148
Female	10	230.0 (186.0,1380.0)	
Male	8	758.0 (521.0,1015.0)	
**Granzyme B⁺ SFC per million PBMCs against omicron variant of SARS-CoV-2**			
Placebo			0.2523
Female	7	1500.0 (948.0,1720.0)	
Male	9	996.0 (284.0,1352.0)	
ChAdTS-S			0.6607
Female	10	1424.0 (1284.0,1960.0)	
Male	9	1392.0 (732.0,1728.0)	
RQ3013			0.2581
Female	9	2184.0 (1164.0,2300.0)	
Male	9	1916.0 (416.0,2168.0)	
ZR202-CoV			0.1220
Female	8	1276.0 (1146.0,1590.0)	
Male	10	680.0 (419.0,1159.0)	
CoronaVac			0.0604
Female	9	356.0 (324.0,596.0)	
Male	8	814.0 (692.0,1286.0)	
**IL-4⁺ SFC per million PBMCs against omicron variant of SARS-CoV-2**			
Placebo			0.5498
Female	9	24.0 (0.0,28.0)	
Male	11	24.0 (0.0,62.0)	
ChAdTS-S			0.9019
Female	12	88.0 (39.0,156.0)	
Male	11	92.0 (42.0,140.0)	
RQ3013			0.4384
Female	10	246.0 (204.0,450.0)	
Male	11	208.0 (98.0,474.0)	
ZR202-CoV			0.0552
Female	9	200.0 (72.0,468.0)	
Male	11	20.0 (10.0,74.0)	
CoronaVac			0.0094
Female	12	0.0 (0.0,26.0)	
Male	9	40.0 (28.0,52.0)	

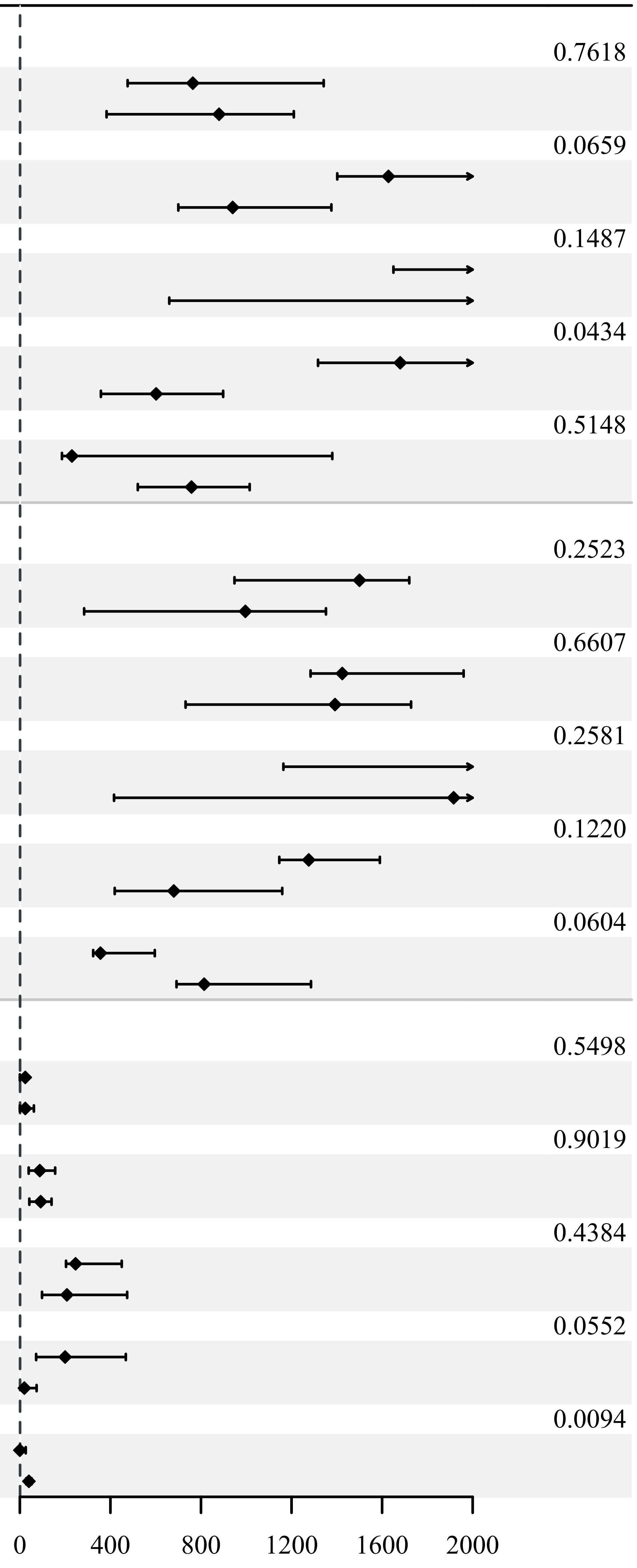

Supplementary Figure 21: Subgroup analysis of T cell responses against omicron variant of SARS-CoV-2 Spike protein at day 14 after the third dose vaccination stratified by sex.

The interferon (IFN)-γ, interleukin (IL)-4 and granzyme B-secreting T cells after stimulating peripheral blood mononuclear cells (PBMCs) with peptides of whole Spike protein epitopes designed based on the omicron variant (B.1.1.529) of SARS-CoV-2 were measured by FluoroSpot assay. T cell responses against omicron variant of SARS-CoV-2 were determined in samples from a random subset of the 234 participants (n=23 for ChAdTS-S, n=21 for RQ3013, n=22 for ZR202-CoV, n=21 for CoronaVac, and n=21 for placebo).

	n	Median(IQR)	p value
**IFN-γ⁺ SFC per million PBMCs against omicron variant of SARS-CoV-2**			
Placebo			0.3562
Female	10	966.0 (572.0,1221.0)	
Male	9	524.0 (300.0,996.0)	
ChAdTS-S			0.5999
Female	11	1252.0 (908.0,1516.0)	
Male	8	1334.0 (1027.0,1884.0)	
RQ3013			0.4119
Female	9	1908.0 (1716.0,2052.0)	
Male	11	908.0 (414.0,2424.0)	
ZR202-CoV			0.2743
Female	8	1866.0 (985.0,2661.0)	
Male	10	1144.0 (534.0,1710.0)	
CoronaVac			0.8286
Female	10	950.0 (318.0,1520.0)	
Male	8	878.0 (648.0,1782.0)	
**Granzyme B⁺ SFC per million PBMCs against omicron variant of SARS-CoV-2**			
Placebo			0.6510
Female	6	1284.0 (603.0,1293.0)	
Male	8	1004.0 (537.0,1106.0)	
ChAdTS-S			0.5672
Female	7	1620.0 (1076.0,2194.0)	
Male	6	1324.0 (876.0,1787.0)	
RQ3013			0.2743
Female	8	2114.0 (1494.0,2548.0)	
Male	10	1086.0 (357.0,2443.0)	
ZR202-CoV			0.8596
Female	6	1586.0 (845.0,2177.0)	
Male	9	776.0 (552.0,1740.0)	
CoronaVac			0.3969
Female	8	806.0 (268.0,1705.0)	
Male	7	1276.0 (912.0,1858.0)	
**IL-4⁺ SFC per million PBMCs against omicron variant of SARS-CoV-2**			
Placebo			0.5323
Female	10	0.0 (0.0,30.0)	
Male	11	0.0 (0.0,44.0)	
ChAdTS-S			0.9262
Female	12	38.0 (26.0,136.0)	
Male	11	44.0 (28.0,116.0)	
RQ3013			0.5032
Female	10	120.0 (54.0,183.0)	
Male	11	88.0 (74.0,130.0)	
ZR202-CoV			0.2243
Female	9	80.0 (0.0,192.0)	
Male	13	36.0 (20.0,48.0)	
CoronaVac			0.1334
Female	12	32.0 (0.0,65.0)	
Male	9	52.0 (44.0,68.0)	

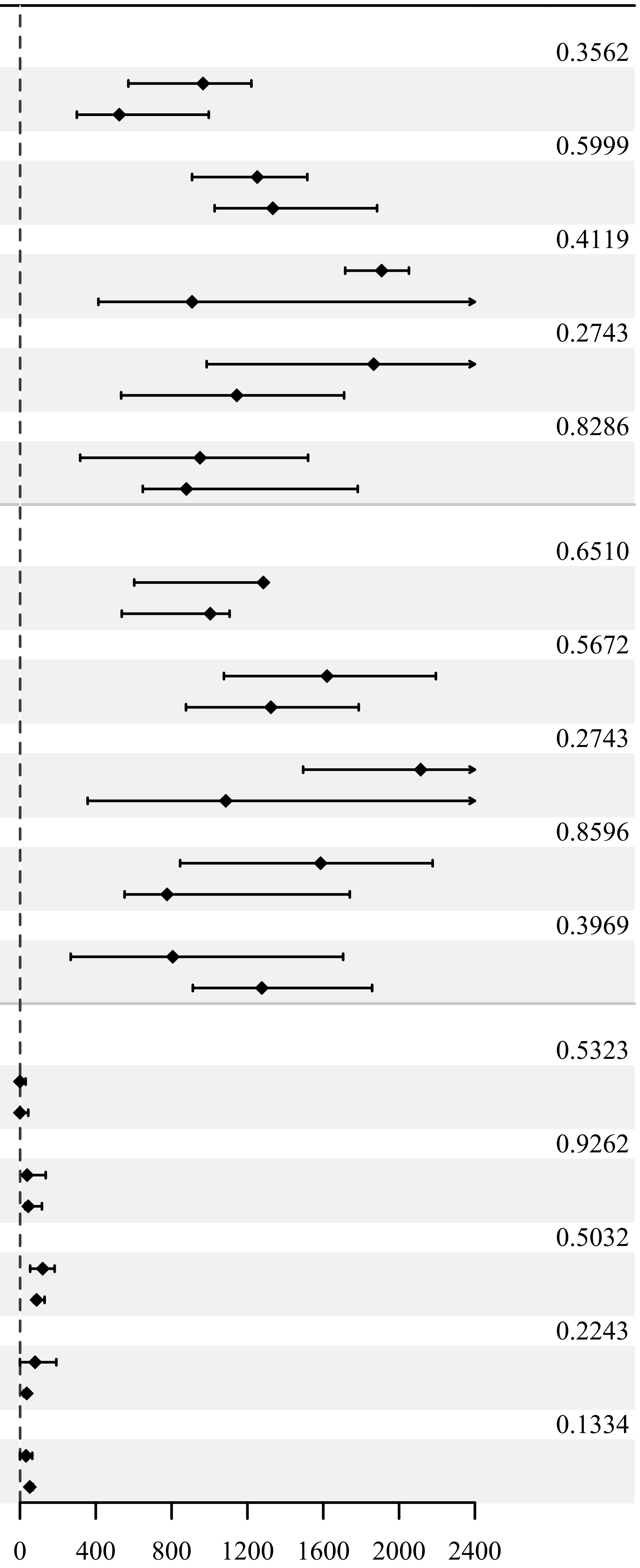

Supplementary Figure 22: Subgroup analysis of T cell responses against omicron variant of SARS-CoV-2 at day 28 after the third dose vaccination stratified by sex.

The interferon (IFN)-γ, interleukin (IL)-4 and granzyme B-secreting T cells after stimulating peripheral blood mononuclear cells (PBMCs) with peptides of whole Spike protein epitopes designed based on the omicron variant (B.1.1.529) of SARS-CoV-2 were measured by FluoroSpot assay. T cell responses against omicron variant of SARS-CoV-2 were determined in samples from a random subset of the 234 participants (n=23 for ChAdTS-S, n=21 for RQ3013, n=22 for ZR202-CoV, n=21 for CoronaVac, and n=21 for placebo).

	n	Median(IQR)	p value
**IFN-γ⁺ SFC per million PBMCs against omicron variant of SARS-CoV-2**			
Placebo			0.5887
Female	6	804.0 (252.0,1062.0)	
Male	6	524.0 (264.0,865.0)	
ChAdTS-S			0.3450
Female	6	1386.0 (861.0,1581.0)	
Male	8	1776.0 (730.0,2223.0)	
RQ3013			0.3357
Female	7	1188.0 (536.0,1734.0)	
Male	8	688.0 (305.0,1201.0)	
ZR202-CoV			1.0000
Female	4	1156.0 (680.0,1708.0)	
Male	8	1102.0 (555.0,2077.0)	
CoronaVac			0.1797
Female	6	472.0 (413.0,603.0)	
Male	6	976.0 (513.0,1583.0)	
**Granzyme B⁺ SFC per million PBMCs against omicron variant of SARS-CoV-2**			
Placebo			0.8307
Female	4	878.0 (147.0,1565.0)	
Male	6	846.0 (375.0,1104.0)	
ChAdTS-S			0.3290
Female	5	748.0 (496.0,988.0)	
Male	6	1168.0 (889.0,1486.0)	
RQ3013			0.6042
Female	4	1178.0 (1005.0,1479.0)	
Male	9	948.0 (440.0,1336.0)	
ZR202-CoV			0.4140
Female	4	1530.0 (746.0,2108.0)	
Male	9	868.0 (356.0,1700.0)	
CoronaVac			0.0152
Female	6	200.0 (84.0,286.0)	
Male	6	786.0 (367.0,1169.0)	
**IL-4⁺ SFC per million PBMCs against omicron variant of SARS-CoV-2**			
Placebo			0.5090
Female	10	0.0 (0.0,21.0)	
Male	9	0.0 (0.0,36.0)	
ChAdTS-S			0.8305
Female	7	52.0 (24.0,66.0)	
Male	9	28.0 (20.0,68.0)	
RQ3013			1.0000
Female	8	26.0 (0.0,127.0)	
Male	9	36.0 (20.0,56.0)	
ZR202-CoV			1.0000
Female	6	14.0 (0.0,73.0)	
Male	10	22.0(5.0,50.0)	
CoronaVac			0.8715
Female	9	0.0 (0.0,36.0)	
Male	8	16.0 (0.0,50.0)	

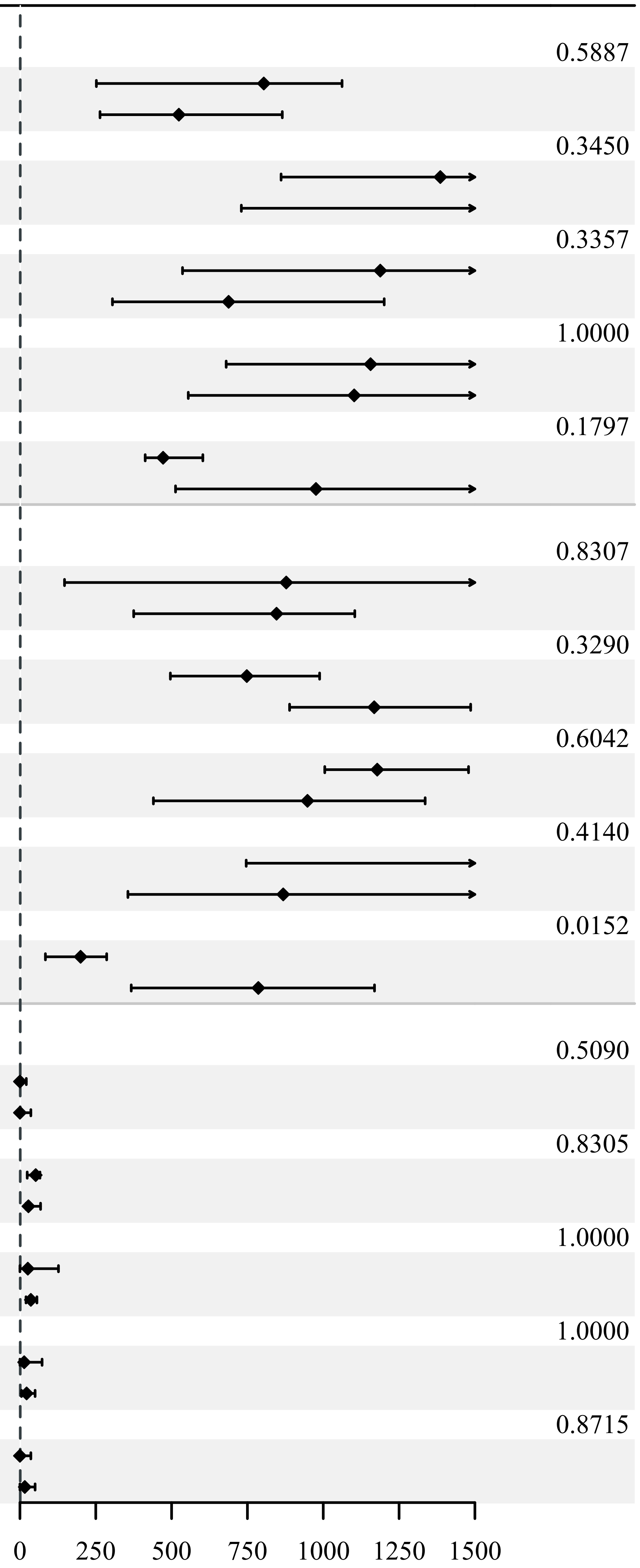

Supplementary Figure 23: Correlations between neutralising antibodies against SARS-CoV-2 after the third dose vaccination by booster schedules.

Neutralising antibodies (NAb) were determined with cytopathic effect (CPE)-based microneutralisation assay using authentic SARS-CoV-2 virus, including the wild-type strain (Wuhan-1, GenBank: MT123291), the delta variant (B.1.617.2, IQTC-IM2175251) and the omicron variant (BA.1.1, IQTC-Y216017) * indicated that the NAb was measured by competitive inhibition method.

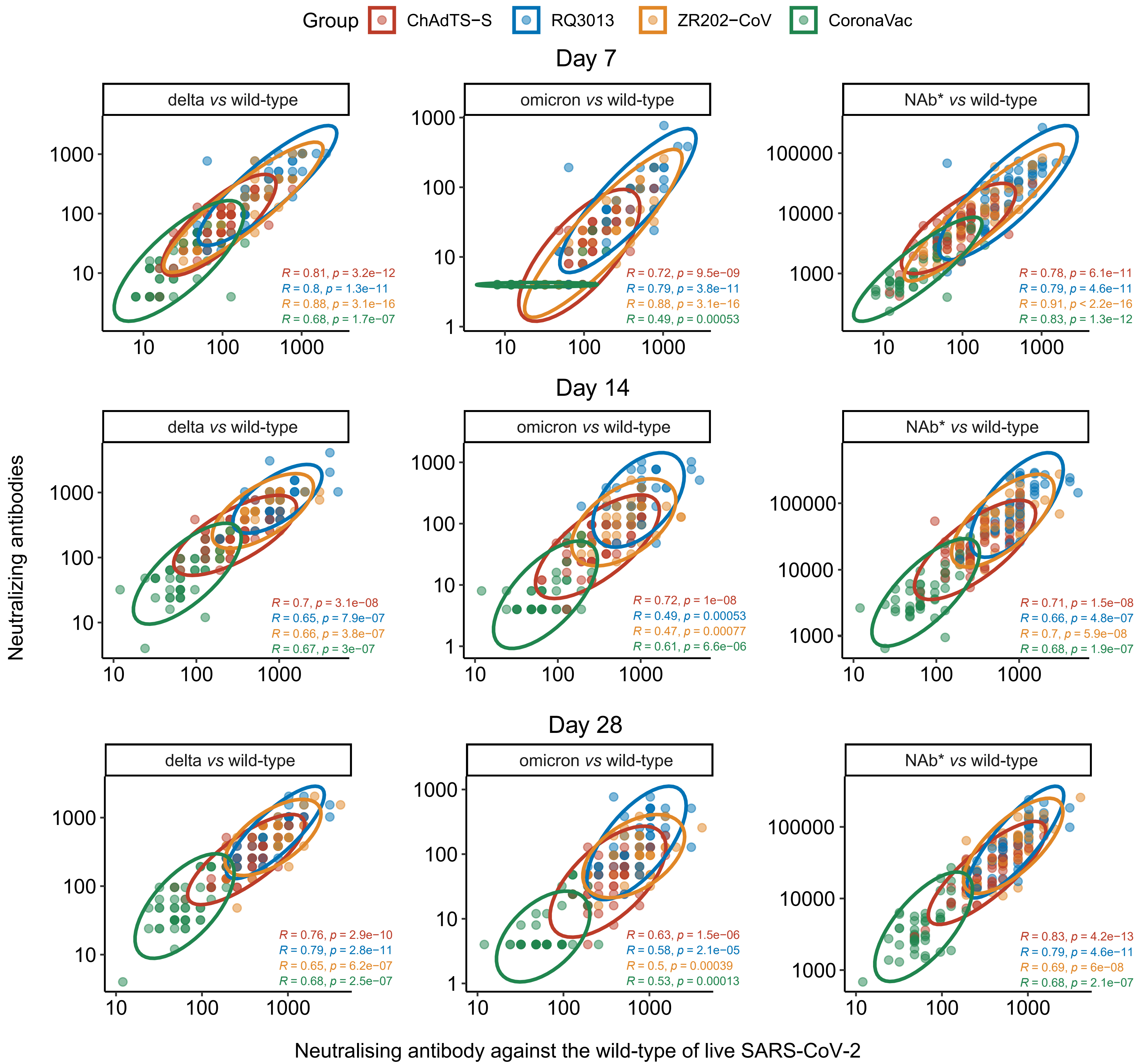
Group
ChAdTS−S
RQ3013
ZR202−CoV
CoronaVac
Day 7
delta vs wild-type
R = 0.81, p = 3.2e−12
R = 0.8, p = 1.3e−11
R = 0.88, p = 3.1e−16
R = 0.68, p = 1.7e−07
omicron vs wild-type
R = 0.72, p = 9.5e−09
R = 0.79, p = 3.8e−11
R = 0.88, p = 3.1e−16
R = 0.49, p = 0.00053
NAb* vs wild-type
R = 0.78, p = 6.1e−11
R = 0.79, p = 4.6e−11
R = 0.91, p < 2.2e−16
R = 0.83, p = 1.3e−12
Day 14
delta vs wild-type
R = 0.7, p = 3.1e−08
R = 0.65, p = 7.9e−07
R = 0.66, p = 3.8e−07
R = 0.67, p = 3e−07
omicron vs wild-type
R = 0.72, p = 1e−08
R = 0.49, p = 0.00053
R = 0.47, p = 0.00077
R = 0.61, p = 6.6e−06
NAb* vs wild-type
R = 0.71, p = 1.5e−08
R = 0.66, p = 4.8e−07
R = 0.7, p = 5.9e−08
R = 0.68, p = 1.9e−07
Day 28
delta vs wild-type
R = 0.76, p = 2.9e−10
R = 0.79, p = 2.8e−11
R = 0.65, p = 6.2e−07
R = 0.68, p = 2.5e−07
omicron vs wild-type
R = 0.63, p = 1.5e−06
R = 0.58, p = 2.1e−05
R = 0.5, p = 0.00039
R = 0.53, p = 0.00013
NAb* vs wild-type
R = 0.83, p = 4.2e−13
R = 0.79, p = 4.6e−11
R = 0.69, p = 6e−08
R = 0.68, p = 2.1e−07
Neutralizing antibodies
Neutralising antibody against the wild-type of live SARS-CoV-2

Supplementary Figure 24: Correlations between T cell responses against SARS-CoV-2 after the third dose vaccination by booster schedules.

The interferon (IFN)-γ, interleukin (IL)-4 and granzyme B-secreting T cells after stimulating peripheral blood mononuclear cells (PBMCs) with peptides of whole Spike protein epitopes designed based on the wild-type or the omicron variant (B.1.1.529) of SARS-CoV-2 were measured by FluoroSpot assay. T cell responses against omicron variant of SARS-CoV-2 were determined in samples from a random subset of the 234 participants (n=23 for ChAdTS-S, n=21 for RQ3013, n=22 for ZR202-CoV, n=21 for CoronaVac, and n=21 for placebo).

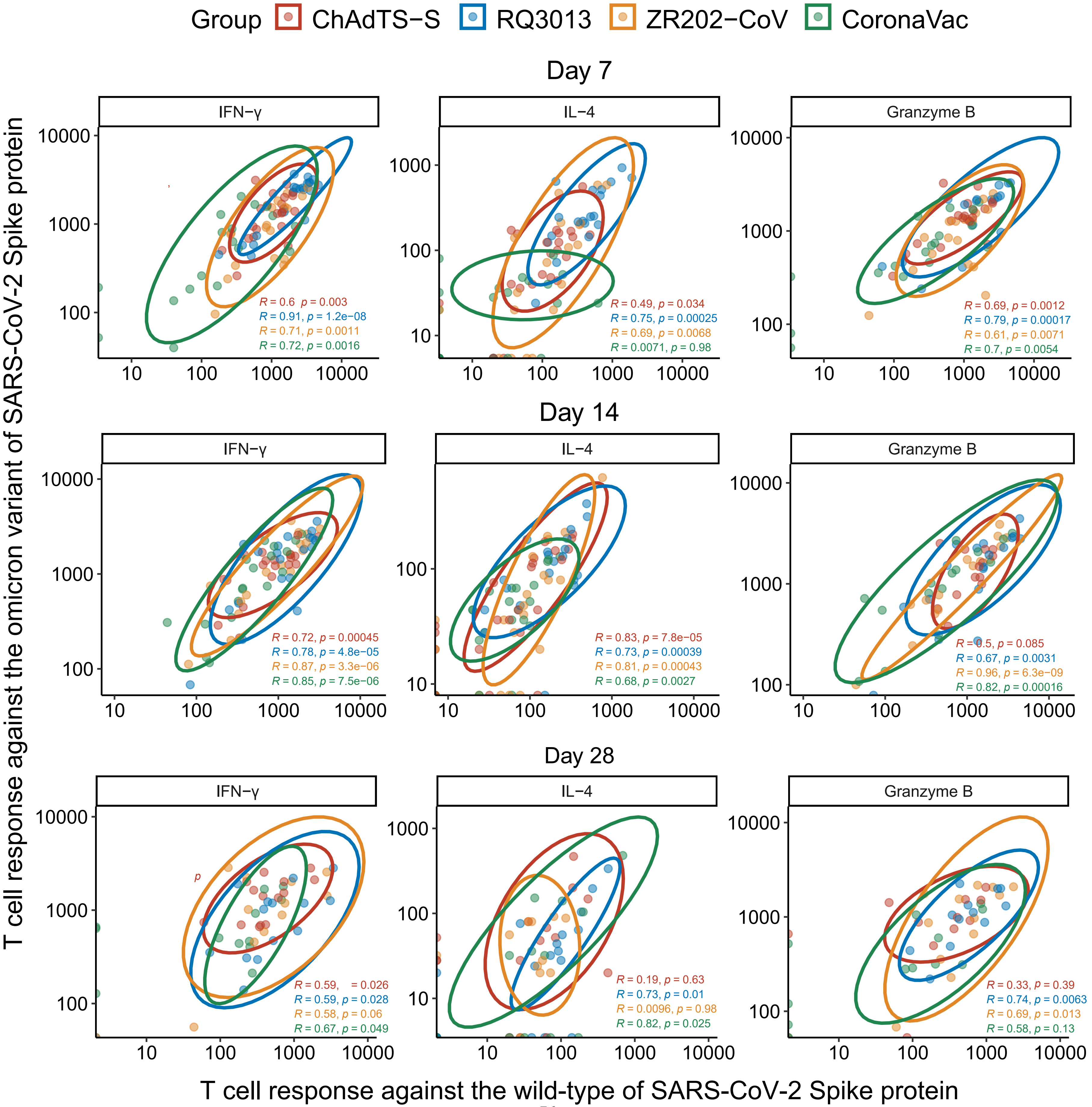
Group ChAdTS−S RQ3013 ZR202−CoV CoronaVac
Day 7
IFN−γ
IL−4
Granzyme B
R = 0.6 p = 0.003
R = 0.91, p = 1.2e−08
R = 0.71, p = 0.0011
R = 0.72, p = 0.0016
R = 0.49, p = 0.034
R = 0.75, p = 0.00025
R = 0.69, p = 0.0068
R = 0.0071, p = 0.98
R = 0.69, p = 0.0012
R = 0.79, p = 0.00017
R = 0.61, p = 0.0071
R = 0.7, p = 0.0054
Day 14
IFN−γ
IL−4
Granzyme B
R = 0.72, p = 0.00045
R = 0.78, p = 4.8e−05
R = 0.87, p = 3.3e−06
R = 0.85, p = 7.5e−06
R = 0.83, p = 7.8e−05
R = 0.73, p = 0.00039
R = 0.81, p = 0.00043
R = 0.68, p = 0.0027
R = 0.5, p = 0.085
R = 0.67, p = 0.0031
R = 0.96, p = 6.3e−09
R = 0.82, p = 0.00016
Day 28
IFN−γ
IL−4
Granzyme B
R = 0.59, = 0.026
R = 0.59, p = 0.028
R = 0.58, p = 0.06
R = 0.67, p = 0.049
R = 0.19, p = 0.63
R = 0.73, p = 0.01
R = 0.0096, p = 0.98
R = 0.82, p = 0.025
R = 0.33, p = 0.39
R = 0.74, p = 0.0063
R = 0.69, p = 0.013
R = 0.58, p = 0.13
T cell response against the omicron variant of SARS-CoV-2 Spike protein
T cell response against the wild-type of SARS-CoV-2 Spike protein

Supplementary Figure 25: Correlations between neutralising antibodies and T cell responses against wild-type SARS-CoV-2 after the third dose vaccination by booster schedules.

Neutralising antibodies were determined with cytopathic effect (CPE)-based microneutralisation assay using the wild-type strain (Wuhan-1, GenBank: MT123291). The interferon (IFN)-γ, interleukin (IL)-4 and granzyme B-secreting T cells after stimulating peripheral blood mononuclear cells (PBMCs) with peptides of whole Spike protein epitopes designed based on the wild-type SARS-CoV-2 were measured by FluoroSpot assay.

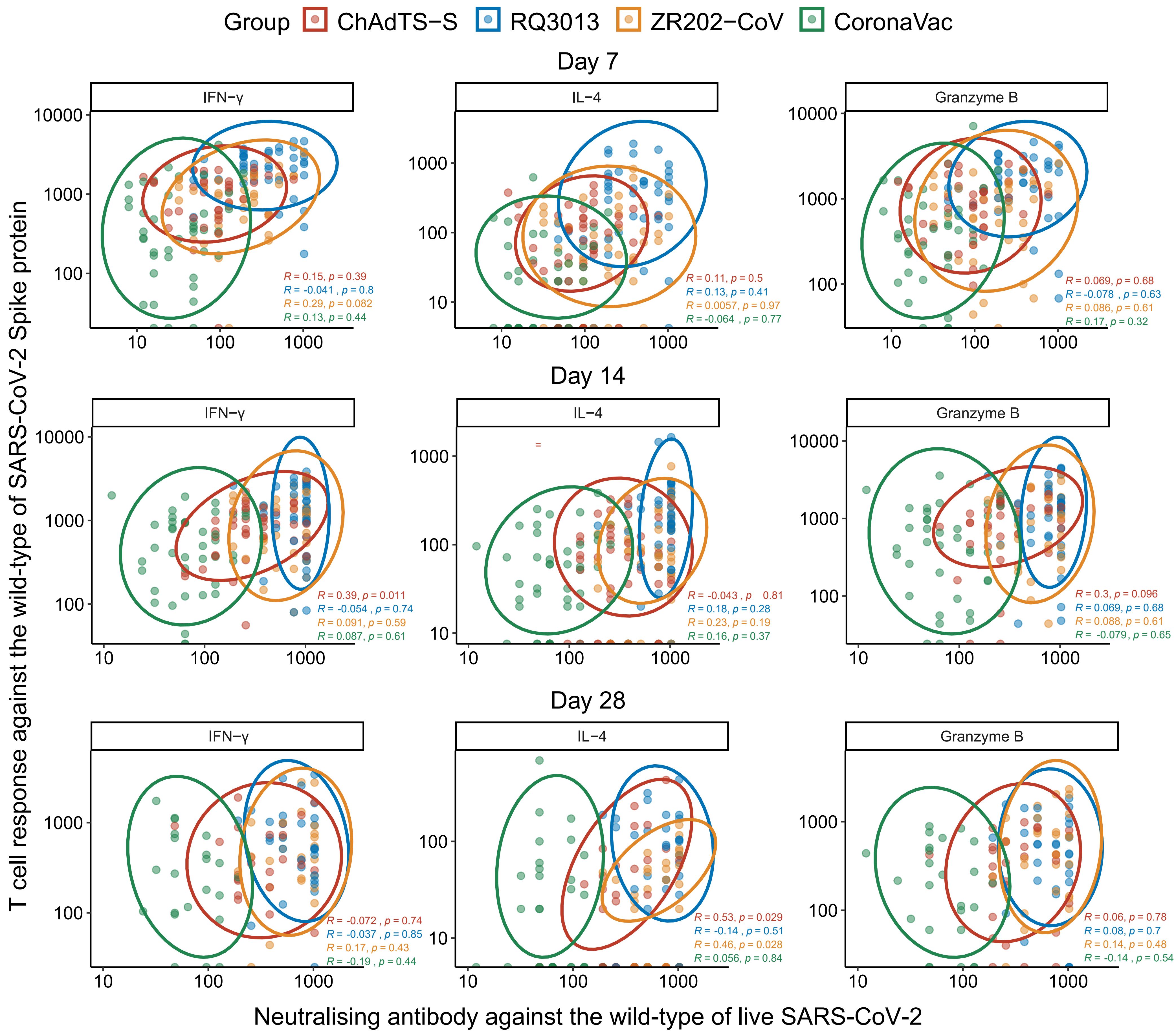

Group ChAdTS-S RQ3013 ZR202-CoV CoronaVac
Day 7
IFN-γ
IL-4
Granzyme B
R = 0.15, p = 0.39
R = -0.041 , p = 0.8
R = 0.29, p = 0.082
R = 0.13, p = 0.44
R = 0.11, p = 0.5
R = 0.13, p = 0.41
R = 0.0057, p = 0.97
R = -0.064 , p = 0.77
R = 0.069, p = 0.68
R = -0.078 , p = 0.63
R = 0.086, p = 0.61
R = 0.17, p = 0.32
Day 14
R = 0.39, p = 0.011
R = -0.054 , p = 0.74
R = 0.091, p = 0.59
R = 0.087, p = 0.61
R = 0.18, p = 0.28
R = 0.23, p = 0.19
R = 0.16, p = 0.37
R = 0.3, p = 0.096
R = 0.069, p = 0.68
R = 0.088, p = 0.61
R = -0.079, p = 0.65
Day 28
R = -0.072 , p = 0.74
R = -0.037 , p = 0.85
R = 0.17, p = 0.43
R = -0.19 , p = 0.44
R = 0.53, p = 0.029
R = -0.14 , p = 0.51
R = 0.46, p = 0.028
R = 0.056, p = 0.84
R = 0.06, p = 0.78
R = 0.08, p = 0.7
R = 0.14, p = 0.48
R = -0.14 , p = 0.54
T cell response against the wild-type of SARS-CoV-2 Spike protein
Neutralising antibody against the wild-type of live SARS-CoV-2

Supplementary Figure 26: Correlations between neutralising antibodies against wild-type SARS-CoV-2 and T cell responses against omicron variant of SARS-CoV-2 after the third dose vaccination by booster schedules.

Neutralising antibodies were determined with cytopathic effect (CPE)-based microneutralisation assay using the wild-type strain (Wuhan-1, GenBank: MT123291). The interferon (IFN)-γ, interleukin (IL)-4 and granzyme B-secreting T cells after stimulating peripheral blood mononuclear cells (PBMCs) with peptides of whole Spike protein epitopes designed based on the omicron variant (B.1.1.529) of SARS-CoV-2 were measured by FluoroSpot assay. T cell responses against omicron variant of SARS-CoV-2 were determined in samples from a random subset of the 234 participants (n=23 for ChAdTS-S, n=21 for RQ3013, n=22 for ZR202-CoV, n=21 for CoronaVac, and n=21 for placebo)

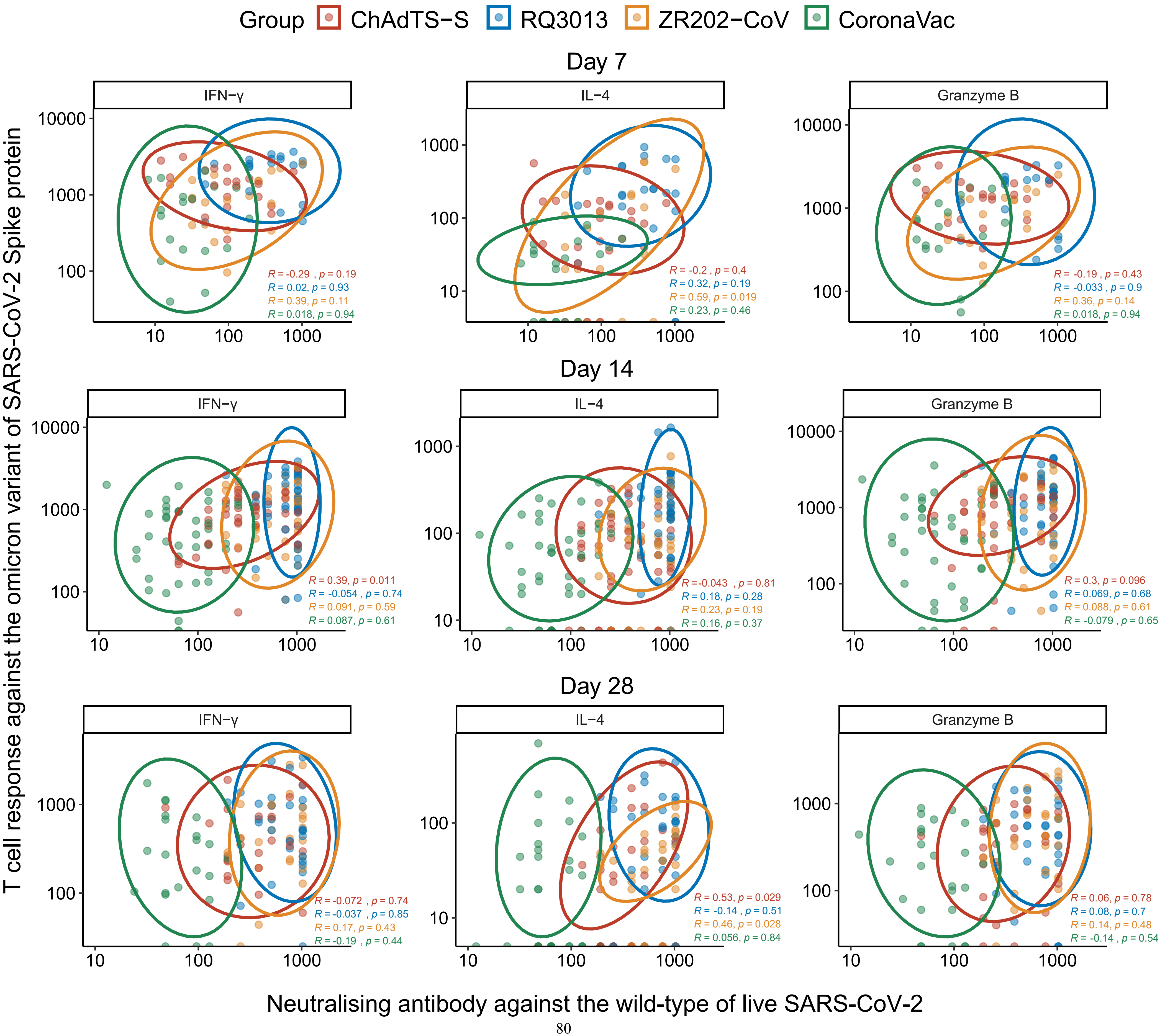
Group ChAdTS−S RQ3013 ZR202−CoV CoronaVac
Day 7
IFN−γ
R = -0.29 , p = 0.19
R = 0.02, p = 0.93
R = 0.39, p = 0.11
R = 0.018, p = 0.94
IL−4
R = -0.2 , p = 0.4
R = 0.32, p = 0.19
R = 0.59, p = 0.019
R = 0.23, p = 0.46
Granzyme B
R = -0.19 , p = 0.43
R = -0.033 , p = 0.9
R = 0.36, p = 0.14
R = 0.018, p = 0.94
Day 14
IFN−γ
R = 0.39, p = 0.011
R = -0.054 , p = 0.74
R = 0.091, p = 0.59
R = 0.087, p = 0.61
IL−4
R = -0.043 , p = 0.81
R = 0.18, p = 0.28
R = 0.23, p = 0.19
R = 0.16, p = 0.37
Granzyme B
R = 0.3, p = 0.096
R = 0.069, p = 0.68
R = 0.088, p = 0.61
R = -0.079 , p = 0.65
Day 28
IFN−γ
R = -0.072 , p = 0.74
R = -0.037 , p = 0.85
R = 0.17, p = 0.43
R = -0.19 , p = 0.44
IL−4
R = 0.53, p = 0.029
R = -0.14 , p = 0.51
R = 0.46, p = 0.028
R = 0.056, p = 0.84
Granzyme B
R = 0.06, p = 0.78
R = 0.08, p = 0.7
R = 0.14, p = 0.48
R = -0.14 , p = 0.54
T cell response against the omicron variant of SARS-CoV-2 Spike protein
Neutralising antibody against the wild-type of live SARS-CoV-2

Supplementary Figure 27: SARS-CoV-2 evolution and vaccine design.

Blue indicates wild-type of SARS-CoV-2, while red indicates the alpha and beta variants of SARS-CoV-2. Solid box shows the four COVID-19 vaccines involved in this study and dashed box shows the reported COVID-19 vaccines in the published studies.

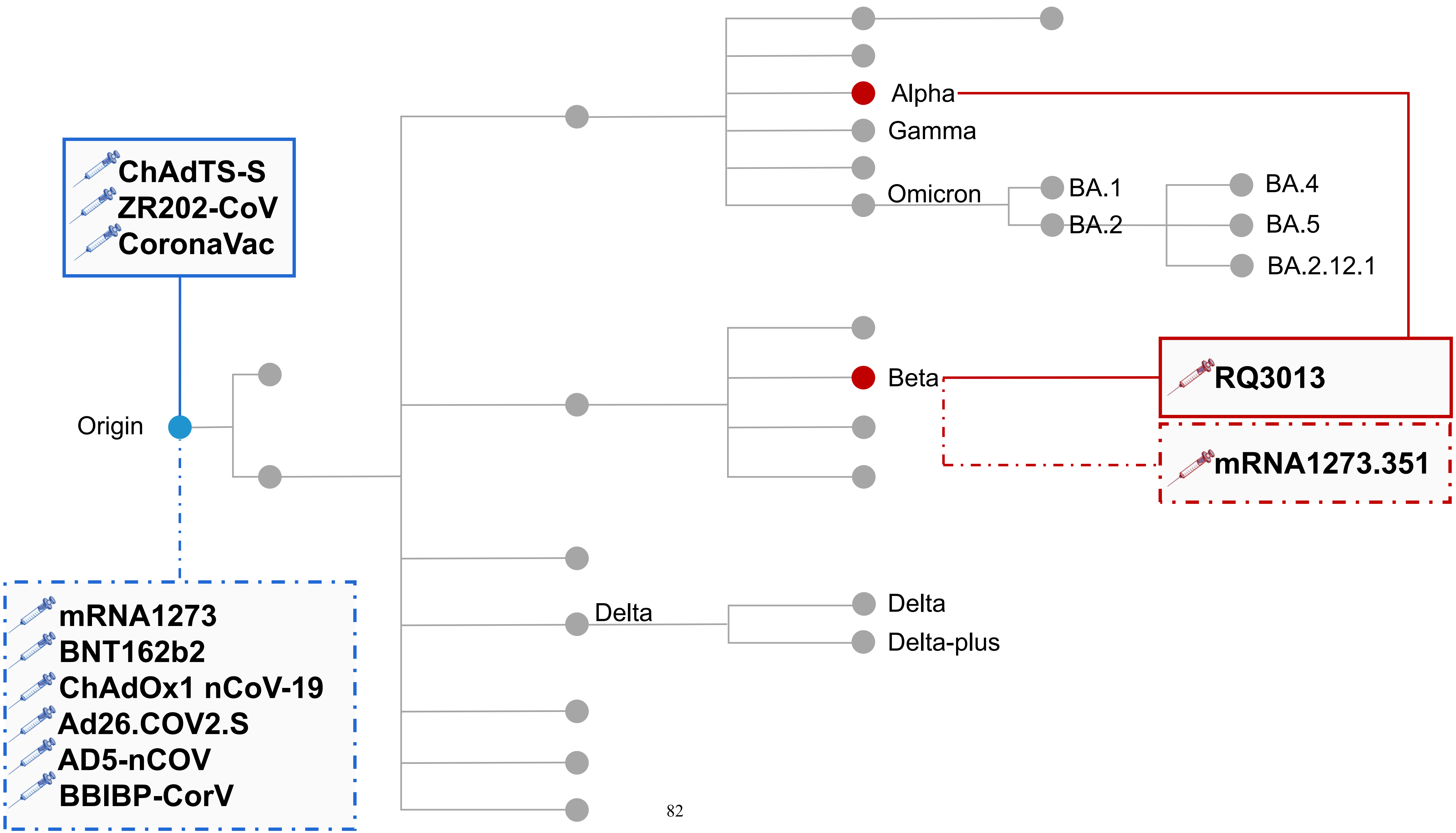
ChAdTS-S
ZR202-CoV
CoronaVac
Origin
mRNA1273
BNT162b2
ChAdOx1 nCoV-19
Ad26.COV2.S
AD5-nCOV
BBIBP-CorV
Alpha
Gamma
Omicron
BA.1
BA.2
BA.4
BA.5
BA.2.12.1
Beta
RQ3013
mRNA1273.351
Delta
Delta
Delta-plus

Supplementary Figure 28: Characteristics of neutralisation, T cell response and reactogenicity profiles of the four COVID-19 vaccines and placebo in this trial.

For neutralisation, we included neutralising antibodies against the wild-type and the omicron variant of SARS-CoV-2 at day 14 after the third dose vaccination to indicate the humoral response magnitude and broadness. Specifically, the geometric mean titers (GMT) of neutralising antibodies for each intervention regimen were normalised by referring the GMT of RQ3013 as 1. For T cell responses, we included IFN-γ-secreting Th1 cells and granzyme B-secreting cytotoxic cells against wild-type of SARS-CoV-2 Spike protein at day 7 after vaccination as indexes and their median values were normalised by referring the median value of RQ3013 as 1. For reactogenicity, we included incident rates of any systemic adverse events or local adverse events within 14 days for comparison.

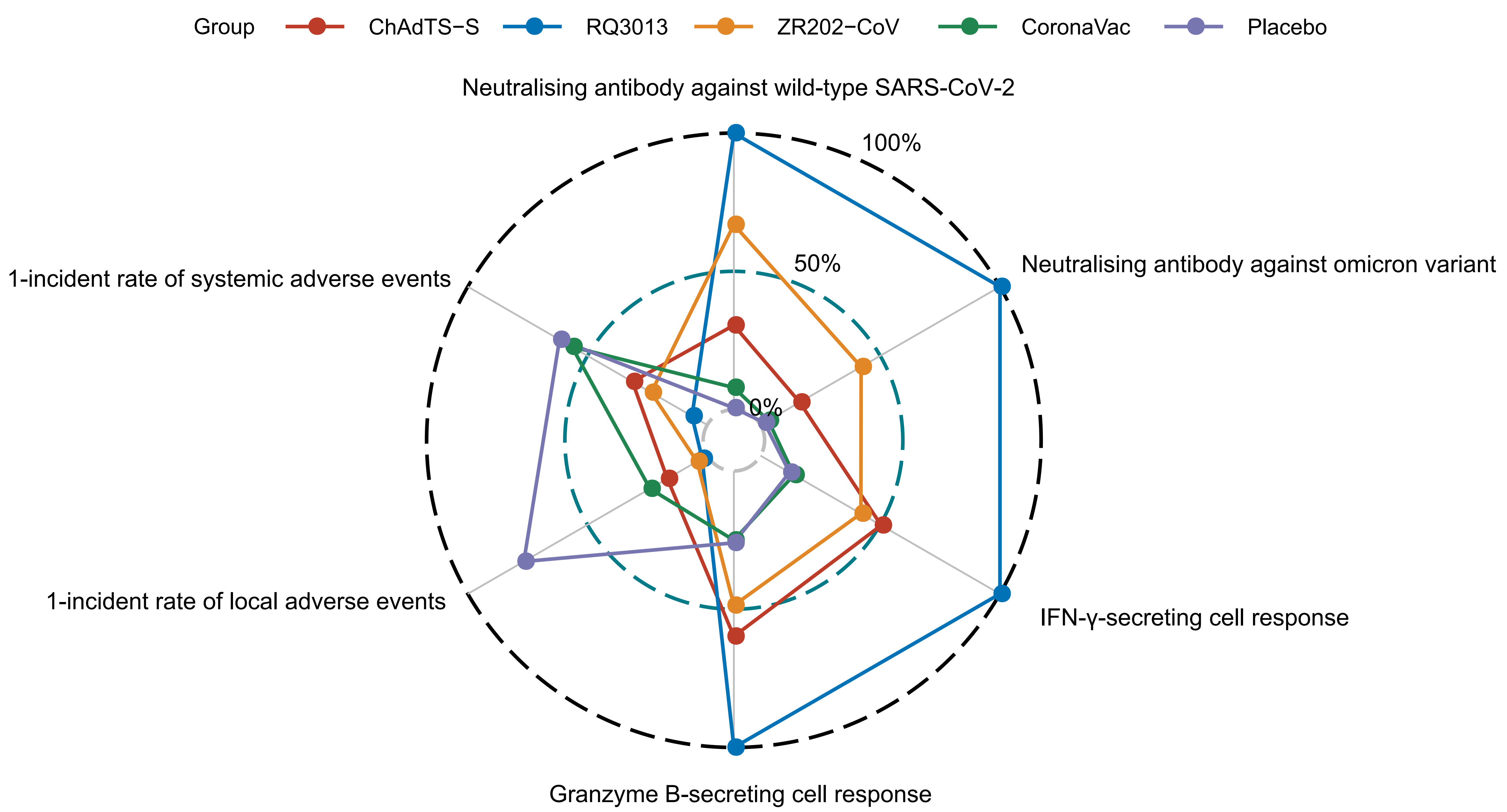
Group
ChAdTS−S
RQ3013
ZR202−CoV
CoronaVac
Placebo
Neutralising antibody against wild-type SARS-CoV-2
Neutralising antibody against omicron variant
IFN-γ-secreting cell response
Granzyme B-secreting cell response
1-incident rate of local adverse events
1-incident rate of systemic adverse events
100%
50%
0%

Immunogenicity, durability, and safety of an mRNA and three platform-based COVID-19 vaccines as a third dose following two doses of CoronaVac in China: a randomised, double-blinded, placebo-controlled, phase 2 trial

Appendix 2

This supplement contains the following items:

1. Study protocol (page 1-55)
2. Statistical analysis plan (page 56-63)

CLINICAL TRIAL PROTOCOL

Clinical Evaluation of Immunogenicity and Safety of Adenovirus-vectored, mRNA, Recombinant Protein and Inactivated Vaccine Booster Dose among Adults Who Have Received Two Doses of Inactivated COVID-19 Vaccine

Protocol Number: YNUVC-2022001
Version Number: 1.0

Sponsor: The Affiliated Hospital of Yunnan University

Research organizations: Yunnan University

The Affiliated Hospital of Yunnan University

Statistical Organization: Kunming Medical University

Southern Medical University

Protocol Approver Signature

Name (print): Jia Wei

Signature: Jia Wei

Date of Signature: 2021.12.15

Study Team & Contact Details

Sponsor

The Affiliated Hospital of Yunnan University

Add.: 176th Qingnian Rd, Kunming, Yunnan, China

Tel.: 0871-65815279 Zip Code: 650021

Contact Person: Jia Wei E-mail: weijia19631225@163.com

Study Institute

The Affiliated Hospital of Yunnan University & Yunnan State Key Laboratory of Conservation and Utilisation of Bioresources, Yunnan University

Add.: 176th Qingnian Rd, Kunming, Yunnan, China & 2nd North Cuihu Rd, Kunming, Yunnan, China

Tel.: 0871-65815279 Zip Code: 650021

Contact Person: Zijie Zhang E-mail: zijiezhang@ynu.edu.cn

Contact Person: Taicheng Zhou E-mail: zhoutc@ynshhyy.com

Clinical Trial Site

Person in Charge of Monitoring

Name: Zijie Zhang

Yunnan University

Add.: 2nd North Cuihu Rd, Kunming, Yunnan, China

Tel.: 0871-65033362 Zip Code: 650091

E-mail: zijiezhang@ynu.edu.cn

Institution Responsible for Humoral and T cell Immunity Testing

Laboratory 1: responsible for blood sample processing, T-cell detection

Institution: State Key Laboratory for Conservation and Utilisation of Bio-resource, Yunnan University

Add.: 2nd North Cuihu Rd, Kunming, Yunnan, China

Tel.: 0871-65033362 Zip Code: 650091

Laboratory 2: responsible for MCLIA antibody and neutralising antibody detection
Institution: Central Lab and Liver Disease Research centre, The Affiliated Hospital of Yunnan University

Add.: 176th Qingnian Rd, Kunming, Yunnan, China

Tel.: 0871-65815279 Zip Code: 650021

Laboratory 3: responsible for the against live virus antibody detection in P3 laboratory

Institution: State Key Laboratory of Respiratory Disease

Add.: 195th Dongfeng west Rd, Guangzhou, Guangdong, China

Tel.: 020-83205074 Zip Code: 510182

Data Management Institution

Institution: State Key Laboratory for Conservation and Utilisation of Bio-resource, Yunnan University

Add.: 2nd North Cuihu Rd, Kunming, Yunnan, China

Contact Person: Zijie Zhang

Tel.: 0871-65033362 Zip Code: 650091 E-mail: zijiezhang@ynu.edu.cn

Statistical Analysis Institution

Institution 1: Kunming Medical University

Add.: 1168th Chunrongxi Road Chenggong District Kunming Yunnan Province

Contact Person: Dingyun You

Tel.: 0871-65922623 Zip Code: 650500 E-mail: youdingyun@kmmu.edu.cn

Institution 2: Guangdong Provincial Key Laboratory of Tropical Disease Research, Department of Biostatistics, School of Public Health, Southern Medical University

Add.: No.1838 Guangzhou Road, Guangzhou City, Guangdong Province, China.

Contact Person: Ying Wu

Tel.: +8602062789194 Zip Code: 510515 E-mail: wuying19890321@gmail.com

External Contract Research Organisation (CRO)

Institution: Yunnan Precise Medical Science Ltd.

Add.: 101st Keyuan Rd, Kunming, Yunnan, China

Contact Person: Zhouxian Li

Zip Code: 650091 E-mail: 283881377@qq.com

Data Safety Monitoring Board (DSMB)

Prof. Longding Liu (Chair)

Institute of Medical Biology, Chinese Academy of Medical Sciences & Peking Union Medical College

E-mail: longdingl@gmail.com

Voting right: Yes

Dr. Xiaoqiang Liu (Clinical vaccine expert)

Centre for vaccine clinical study, Yunnan Centres for Disease Control and Prevention

E-mail: lxq7611@126.com

Voting right: Yes

Prof. Yuanyuan Xiao (Statistician)

School of public health, Kunming Medical University

E-mail: 33225647@qq.com

Voting right: Yes

Principal Investigator Agreement Page

I agree:

- Assume the responsibility for properly instructing this clinical trial.
- Ensure that the trial is conducted is carried out in accordance with the Trial Protocol and standard operating procedure for clinical research.
- Ensure that personnel involved in this trial are fully aware of the research product information, as well as other responsibilities and obligations in connection with the Research as specified in the Trial Protocol.
- Ensure that no changes to the trial protocol are made without review and written approval of the sponsor and the Independent Ethics Committee (IEC), unless necessary to eliminate immediate harm to subjects or as required by the registration authority (e.g., administration of the Project).
- I am fully familiar with the proper use of the vaccine as described in the trial protocol and am fully aware of other information provided by the sponsor, including but not limited to the following: the current Investigator's Brochure (IB) or equivalent document and supplementary documents to the IB (if any).
- I am familiar with and will comply with Good Clinical Practice (GCP), Guidelines for the Quality Management of Vaccine Clinical Trials (trial implementation) and all current regulatory requirements.

Investigator's Name (print): Jia Wei

Investigator's Signature: Jiawei

Date of Signature: 2021、12.15

Abbreviations

ACE2	Angiotensin-Converting-Enzyme 2
ASaT	All Subjects as Treated
BAU	Binding Antibody Unit
BUN	Blood Urea Nitrogen
CI	Confidence Interval
COVID-19	Corona Virus Disease 2019
CPE	Cytopathic Effect
CRF	Case Report Form
CRO	Contract Research Organisation
DMEM	Dulbecco's Modified Eagle Medium
DMSO	Dimethylsulfoxide
DSMB	Data and Safety Monitoring Board
e-diary	electronic diary
FAS	Full Analysis Set
GCP	Good Clinical Practice
GMR	Geometric Mean Ratio
GMT	Geometric Mean Titre
HIV	Human Immunodeficiency Virus
HPF	High Power Field
HREC	Human Research Ethics Committee
HRP	Horseradish Peroxidase
IB	Investigator's Brochure
IEC	Independent Ethics Committee
ICH	International Conference of Harmonisation
ICU	Intensive Care Unit
IFN-γ	Interferon-γ
IL-2	Interleukin-2
IL-4	Interleukin-4
IL-13	Interleukin-13
IQR	Inter-quartile range
ITT	Intention-to-Treat

IU	International Unit
MCLIA	Magnetic particle ChemiLuminescence ImmunoAssay
mRNA	messenger Ribonucleic Acid
NA	Not Available
NAb	Neutralizing Antibody
NTD	N-Terminal Domain
PBMC	Peripheral Blood Mononuclear Cell
PPS	Per Protocol Set
RBC	Red Blood Cell
RBD	Receptor Binding Domain
RCT	Randomised Controlled Trial
RPMI	Roswell Park Memorial Institute (RPMI)
S	Spike
SARS-CoV-2	Severe Acute Respiratory Syndrome Coronavirus 2
S/CO	chemiluminescence values divided by the cutoff (S/CO)
SFC	Spot Forming Cell
TCID50	Tissue Culture Infective Dose 50
TNF-α	Tumour Necrosis Factor-α
WBC	White Blood Cell

Table of Contents

1. Overview of the Study 12
Figure 1. Study Design. 15
2. Background & Rationale 16
2.1 COVID-19 16
2.2 Vaccination 16
Table 1. Different Platforms of COVID-19 Vaccine Development. 17
Table 2. Characteristics of the Booster Regimen and Immunogenecity in the Published Studies. 19
2.3 Rationale and Significance of this Study 21
3. Trial Objectives 21
3.1 Primary Immunogenecity Objectives (Blinded) 21
3.2 Secondary Immunogenecity Objectives 22
3.3 Primary Safety Objectives (Blinded) 22
3.4 Secondary Safety Objectives 22
4. Trial Design 23
4.1 Pre-randomisation Period (3 weeks) 23
4.2 Randomisation and Vaccination Period (1 day, day 0) 23
4.3 Follow-up Period (180 days, day 1 ~ day 180) 24
Figure 2. Study Profile. 25
5. Trial Intervention 26
5.1 Investigational Vaccines 26
Table 3. Vaccines under Investigation. 26
5.2 Preparation/Handling/Storage/Accountability 27
5.3 Administration 28
6. Selection and Withdrawal of Participants 28
6.1 Target Population 28
6.2 Inclusion Criteria 28
6.3 Exclusion Criteria 29
6.4 Screening and Selection of Participants 29
6.5 Screening Log 30
6.6 Randomisation & Blinding 30
6.7 Breaking the Blinding 31
6.8 Withdrawal of Participants 32
6.9 Expected Duration of Trial 32

7. Study Procedures and Assessments 32
7.1 By Visit 32
Table 4. Schedule of Study Tests, Procedures and Clinic Visits. 34
7.2 Physical Examination & Screening 35
7.3 Vaccine Distribution and Inoculation 35
7.4 Sampling, Processing and Preservation 35
7.4.1 Blood Sampling 35
Table 5. Maximum Blood Sampling Volumes per Participant by Visits. 36
7.4.2 Sample Processing and Preservation 36
7.5 Immunogenicity Assessment 37
7.5.1 Live Virus Neutralizing Antibody against Wild-type SARS-CoV-2, the delta and omicron Variants 37
7.5.2 anti-SARS-CoV-2 RBD-specific IgG, IgM and IgA, and Neutralizing Antibody using Competitive Inhibition Method 37
7.5.3 Cellular Immune Responses 38
7.6 Safety Assessment 39
7.6.1 Duration of Observation 39
7.6.2 Laboratory Evaluations 40
7.6.3 e-diary 40
7.6.4 Safety Observation Contents and Indicators 41
Table 6. Grading of Injection Site (Local) Adverse Events 41
Table 7. Grading of Systemic Adverse Events 42
7.6.5 Outcomes of Adverse Events 43
7.6.6 Relationship between Adverse Events and Vaccination 43
7.6.7 Documentation and Reporting of Adverse Events 44
7.7 Pregnancy 44
7.8 Unscheduled Visits 44
7.9 Data Handling & Management 44
8. Statistics 45
8.1 Sample Size Calculation and Reasoning 45
8.2 Statistical Analyses 46
9. Participant Confidentiality & Record Keeping 46
9.1 Participant Confidentiality 46
9.2 Investigator's Files /Source Documents/ Retention of Documents 47
10. Quality Assurance Procedures 47

10.1 Obtaining Informed Consent 47
10.2 Delegation of Investigator Duties 48
10.3 Ethics and Regulatory Approvals 49
10.4 Management of Protocol Deviations 49
10.5 GCP Training and Site Monitoring 49
10.6 Audits and Inspections 50
10.7 Executive Committee and Steering Committee 50
10.8 Data and Safety Monitoring Board (DSMB) 51
10.9 Termination of the Study 51
11. Publication Policy 51
Appendix 1: Definitions of Adverse Events 52
Appendix 2: Specification of Source Data 53
References 54
STATISTICAL CONSIDERATIONS 57
Part A: Sample Size Calculation and Reasoning 57
Table 1. Probability of Testing Vaccination Safety. 57
Part B: Statistical Analyses 58
1. Selection of Analysis Data Sets 58
1.1 Immunogenicity Data Set 58
1.2 Safety Data Set 58
1.3 General Principles 58
2. Baseline Demographics 59
3. The Primary Immunogenicity Outcome Analysis 59
3.1 Definition of Outcome 59
3.2 Population for Analysis 59
3.3 Statistical Analysis 60
3.4 Subgroup Analyses 60
3.5 Missing Data 61
4. Secondary Immunogenicity Outcome Analysis 61
4.1 Definition of Outcomes 61
4.2 Population for Analysis 61
4.3 Statistical Analysis 61
5. Safety Outcome Analysis 62
5.1 Definition of Outcomes 62
5.2 Population for Analysis 62

5.3 Statistical Analysis 63
Part C: Initial Analysis 63

1. Overview of the Study

Title	Clinical evaluation of immunogenicity and safety of adenovirus-vectored, mRNA, recombinant protein and inactivated vaccine booster dose among adults who have received two doses of inactivated COVID-19 vaccine
Sponsor	The Affiliated Hospital of Yunnan University
Investigational Vaccines	Vaccine 1: ChAdTS-S (Spike adenovirus-vectored)<br>Vaccine 2: RQ3013 (mRNA encoding a mutated Spike protein)<br>Vaccine 3: ZR202-CoV (Spike ectodomain trimer)<br>Vaccine 4: CoronaVac (inactivated whole virus)<br>Placebo: normal saline (0.9% sodium chloride solution for injection)
Study Purpose	This study will evaluate the immunogenicity and safety of a third dose of ChAdTS-S, RQ3013, ZR202-CoV, CoronaVac or placebo on participants vaccinated with two doses of inactivated COVID-19 vaccine (CoronaVac).
Study Design	A multi-arm, double-blinded, randomised, placebo-controlled study
Target Population	The target population will consist of healthy adults vaccinated with 2 doses of inactivated COVID-19 vaccine (CoronaVac).
Planned Sample Size	250 participants
Inclusion Criteria	◆ 18-59 years old, male or female.<br>◆ Completed two doses of inactivated COVID-19 vaccine (CoronaVac) with a 3-5 weeks interval.<br>◆ It is now in day 100 to day 270 after the second dose of vaccination.<br>◆ Healthy participants who are determined according to medical history, physical examination, and clinical judgment by the investigator to be eligible for inclusion in the study.<br>◆ Participants who can comply with the study protocol in the view of the treating physician.<br>◆ Participants who are capable of giving personal signed informed consent.

Exclusion Criteria	◆ Previous infection history of SARS-CoV-2.<br>◆ History of severe adverse reaction and/or allergic reaction associated with a vaccine or any vaccine components.<br>◆ Pregnancy or planned pregnancy.<br>◆ Breastfeeding.<br>◆ Medical history of uncontrolled chronic diseases, such as coronary heart disease, hypertension, diabetes, chronic respiratory disease, tumours.<br>◆ Severe psychiatric disorders.<br>◆ Individuals who received treatment with immunosuppressive therapy.<br>◆ Receipt of blood/plasma products or immunoglobulin from 60 days before the study or planned receipt throughout the study.<br>◆ Previous or current participation in other studies involving study intervention that could disturb the safety and efficiency assessment in this study.<br>◆ Participants who have any inapplicable factors according to the study protocol in the view of investigator.
Blood Sampling	At day 0 (before the booster vaccination), day 1, day 4, day 7, day 14, day 28, day 90 and day 180 after the booster dose
Immunogenicity Outcomes	Primary outcomes:<br>● Geometric mean titres (GMTs) of serum neutralizing antibody against the wild-type, delta and omicron variants of live SARS-CoV-2 at day 0 (before the booster vaccination), and day 7, day 14 and day 28 after the booster dose.<br>Secondary outcomes:<br>● GMTs of serum neutralizing antibody against the wild-type, delta and omicron variants of live SARS-CoV-2 at month 3 after the booster dose.<br>● GMTs of serum neutralizing antibody against the wild-type, delta and omicron variants of live SARS-CoV-2 at month 6 after the booster dose.<br>● GMTs of serum neutralizing antibody measured by competitive inhibition method at day 0 (before the booster vaccination), and day 1, day 4, day 7, day 14, day 28, day 90 and day 180 after the booster dose.<br>● GMTs of serum SARS-CoV-2 receptor binding domain (RBD)-specific IgG, IgM and IgA at day 0 (before the booster vaccination), and day 1, day 4, day 7, day 14, day 28, day 90 and day 180 after the booster dose.<br>● The proportions of SARS-CoV-2 S-protein-specific interferon (IFN)-γ, interleukin (IL)-4- and granzyme B-secreting T cells at day 0 (before the booster vaccination), and day 7, day 14 and day 28 after the booster dose (FluoroSpot [Mabtech]).

	● The proportions of SARS-CoV-2 S-protein-specific CD4+ and CD8+ T cells secreting IFN-γ, tumour necrosis factor (TNF)-α, IL-4, IL-13, IL-2 at day 0 (before the booster vaccination), and day 7, day 14 and day 28 after the booster dose (flowcytometry method).
Safety outcomes:	Primary outcomes:<br>● The incidence of adverse events in each group within 14 days after the booster vaccination.<br>● The incidence of Grade 3 or higher adverse event rates within 14 days after the booster vaccination.<br>Secondary outcomes:<br>● Adverse events, especially the adverse event rates, within 7 days after the booster vaccination.<br>● Adverse events, especially the adverse event rates, within 28 days after the booster vaccination.<br>● Adverse events, especially the adverse event rates, within 3 months after the booster vaccination.<br>● Adverse events, especially the adverse event rates, within 6 months after the booster vaccination.<br>● Pregnancy events within 6 months after the booster vaccination.

Figure 1. Study Design.

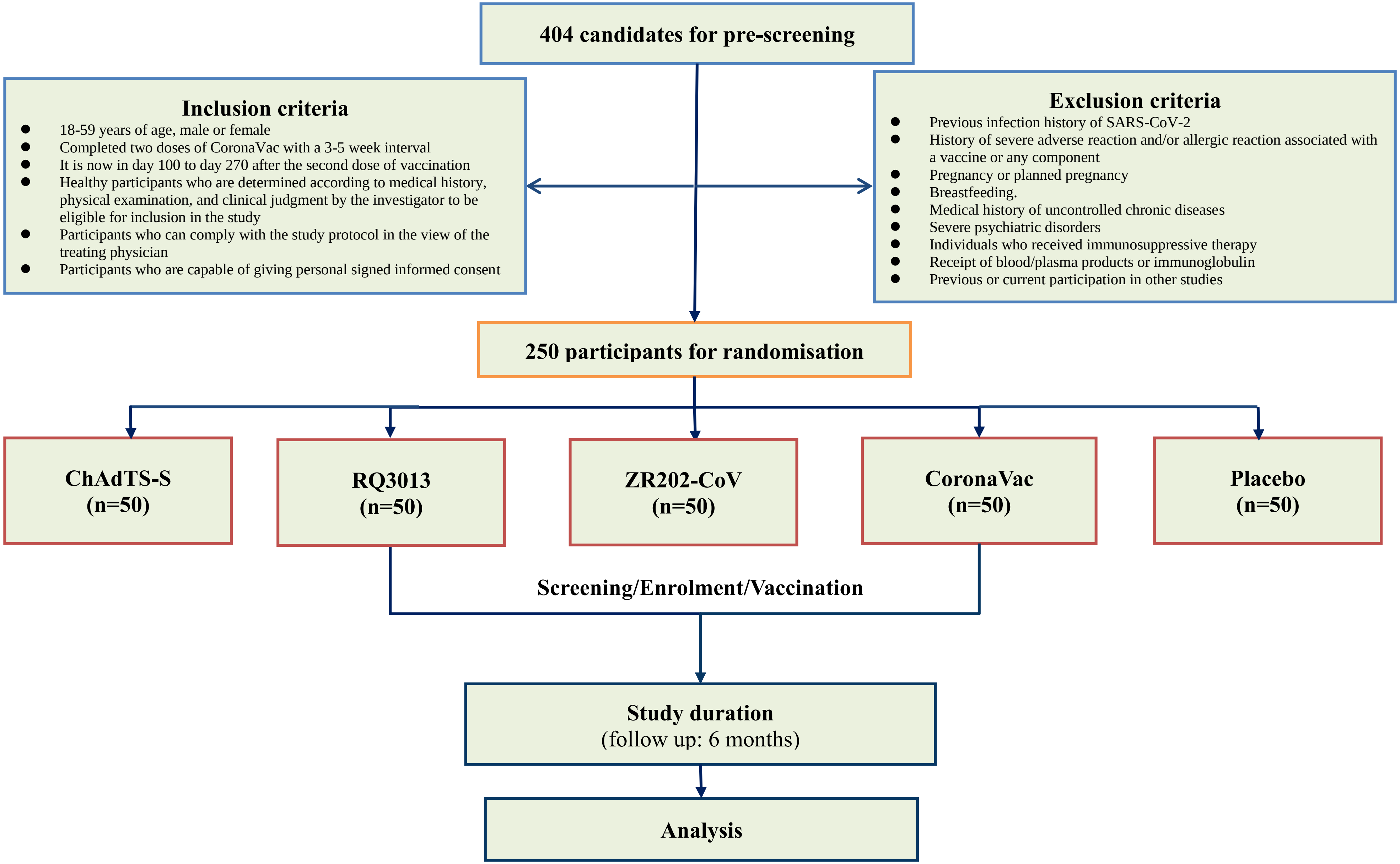

2. Background & Rationale

2.1 COVID-19

Coronavirus disease 2019 (COVID-19) caused by severe acute respiratory syndrome coronavirus 2 (SARS-CoV-2) has become a pandemic since March 11, 2020, which remains to be a global public health threat with continuous evolving variants. Among proteins encoded by SARS-CoV-2 genome, Spike glycoprotein (S protein) is the most important surface protein of coronavirus, containing two subunits: S1 and S2. S1 mainly contains receptor binding domain (RBD), which is responsible for the recognition of cellular receptors, such as angiotensin-converting enzyme 2 (ACE2), and related to the transmission ability of the virus. S2 contains the basic elements for the membrane fusion process. In the development of COVID-19 vaccines, S protein is the most widely used candidate immunogen while some vaccines use RBD domain as the immunogen.

The emerging and spreading of SARS-CoV-2 variants kept raising new concerns about the pandemic. Till now, 7 SARS-CoV-2 variants have been isolated and detected, including alpha (B.1.1.7), beta (B.1.351), epsilon (B.1.429), gamma (P.1 V2), kappa (B.1.617.1), delta (B.1.617.2) and omicron (B.1.1.529 and the BA.1 and BA.2 sublineages). These mutations result in the high transmission rate and potential for immune evasion. Among them, the omicron variant, which is now being reported in epidemiological studies, harbours up to 59 mutations throughout its genome, with as many as 37 of these occurring within the S protein, the mediator of host cell entry and the main target of neutralizing antibodies. Thus, how to broaden neutralizing antibody responses against highly divergent SARS-CoV-2 variants remains to be important for controlling the pandemic.

2.2 Vaccination

Vaccine is one of the most effective ways to control the COVID-19 global pandemic. Till now, a variety of vaccine platforms have been developed and are being deployed globally. At the time of proposal preparing, there are three major research and development technology routes of COVID-19 vaccines being approved by World Health Organisation (WHO), namely inactivated vaccine, adenovirus-vectored vaccine, and mRNA vaccine (**Table 1**).[1] Numbers of clinical trials have shown the good protective efficacy and safety of these COVID-19 vaccines.[2-5]

Table 1. Different Platforms of COVID-19 Vaccine Development.

R&D platforms	Mechanism of Action	Vaccines given Emergency Use Listing by WHO	Approved Date
mRNA vaccine	mRNA vaccine encoding SARS-CoV-2 spike glycoprotein	BNT162b2; mRNA1273	31 December 2020 30 April 2021
Adenovirus-vectored vaccine	Replication-deficient chimpanzee adenovirus-vectored vaccine, expressing the SARS-CoV-2 spike glycoprotein	Ad26.COV2.S ChAdOx1-S	12 March 2021 16 February 2021
Inactivated vaccine	Whole, inactivated SARS-CoV-2 virus	Sinopharm COVID-19 vaccine; CoronaVac	7 May 2021; 1 June 2021

Note: The approved COVID-19 vaccines were searched before protocol inception (October 2021) (https://www.who.int/).

However, like all other vaccines, there is evidence that immunogenicity of COVID-19 vaccines has waned with time, similar to post-infection. This together with the increased transmission capability of the omicron variant (partially attributed to its strong immune escape) resulted in increasing numbers of breakthrough infections in fully vaccinated individuals. Concerns about waning immunity and the potential for existing schedules to protect against new SARS-CoV-2 variants of concern have led to questions on the optimisation of vaccine schedules. Booster vaccinations, both homologous and heterologous schedules, have received emergency use authorisation.

We searched PubMed for randomised controlled trials (RCTs) published during protocol preparation (December 2021) using terms “(COVID-19 OR SARS-CoV-2) AND (vaccine) AND (booster OR third dose)” with no language restrictions. There are four RCTs evaluating the boosting effects of a third dose of COVID-19 vaccine (**Table 2**). Homologous schedule studies include booster doses with ChAdOx1-S,[6] BNT162b2[7] or CoronaVac,[8] while the heterologous schedule study (COV-BOOST) includes booster doses with multi-platform vaccines in individuals primed with ChAdOx1-S or BNT162b2.[9] Specifically, the COV-BOOST study is a multicentre trial aiming to evaluate safety and immunogenicity of seven COVID-19 vaccines as a third dose (booster) following two doses of ChAdOx1 nCov-19 or BNT162b2 in the UK. It was shown that all studied vaccines, except for VLA2001 (an inactivated whole COVID-19 vaccine) in the two-dose-BNT162b2-primed participants, boosted humoral immune responses, with no safety concerns. Substantial differences in humoral and cellular responses were observed for different vaccines. Overall, the available evidence suggests that a third dose vaccination is effective and well tolerated:

- **Efficacy**: Nearly all studies observed a significant increase in neutralizing antibodies. Among all combinations of vaccine schedules, mRNA vaccine (BNT162b2 or mRNA1273) appeared to induce higher neutralizing antibody titres despite of higher but well-tolerated reactogenicity than other vaccines.
- **Safety**: The booster dose vaccination was found to be well tolerated.
- **Boosting interval**: Longer time interval would further enhance the immune responses.
- **Sequential vaccination**: Heterologous boosting schedule is a strategy that could enhance both immunogenicity and deployment flexibility to improve accessibility to vaccines. Evidence to support the use of mixed schedules is rapidly evolving.

The inactivated COVID-19 vaccine is one of the most widely used COVID-19 vaccine in China and many other countries with over 3 billion doses administrated. However, as far as we known, there is only two small prospective studies evaluating the booster effect of ZF2001[10] and ChAdOx1 nCoV-19[11] in participants primed with two-dose inactivated COVID-19 vaccines (CoronaVac or BBIBP-CorV). Both of them showed that a third heterologous booster of protein subunit vaccine or adenovirus-vectored vaccine was safe and highly immunogenic. However, although these studies brought important insights into the value of a booster vaccination on the basis of two-dose inactivated vaccine immunisation, several limitations should also be noted:

- None or only a small number of participants in these studies tested the live virus neutralizing antibody, especially against for the omicron variant of SARS-CoV-2.
- Most studies only focused on the neutralizing antibodies while less attention was on the cellular immune response. The paucity of T-cell response data makes it unable to systemically evaluate the immunogenic effect of the booster dose vaccination.
- Till now, only one animal study evaluated all the four platform vaccines (mRNA, adenovirus-vectored, inactivated, and recombinant protein) as booster vaccines based on CoronaVac/CoronaVac prime immunisation.[12] It is still lacking a formal clinical trial to evaluate the immunogenicity and safety of multi-platform vaccines for those primarily vaccinated with inactivated vaccines. A well-designed RCT is necessary to optimise the booster schedule.

Table 2. Characteristics of the Booster Regimen and Immunogenicity in the Published Studies.

Date	PMID	Prime Vaccination	The first-second dose interval	Booster Vaccination	The prime-boost dose interval	NAb against the wild-type variant	NAb against the delta variant	NAb against the omicron variant	Detection time point	Method	T cell
**Randomised Controlled Trials of a Booster Dose Vaccination**											
2021 Sep 1	34480858	2-ChAdOx1 nCoV-19	8-16 weeks	NA	NA	NA	78 (Ref)	NA	28 days after vaccination	Focus reduction neutralisation assays	√
			8-16 weeks	ChAdOx1 nCoV-19	28-38 weeks	NA	206 (2.64)	NA	28 days after vaccination		
2021 Sep 15	34525276	2-BNT162b2	21 days	NA	NA	83 (Ref)	NA	NA	7.9-8.8 months after vaccination	plaque-reduction neutralisation testing	×
			21 days	BNT162b2	7.9-8.8 months	1754 (21.13)/2119 (25.53)	NA	NA	day 7 and day 30 after vaccination		
2021 Dec 2	34863358	2-ChAdOx1 nCoV-19	NA	Control	>70 days	Ref	Ref	NA	28 days after vaccination	pseudotype virus neutralisation assays	√
			NA	ChAdOx1 nCoV-19	>70 days	193 (2.47)	48.9 (2.58)	NA	28 days after vaccination		
			NA	mRNA1273	>70 days	2368 (26.98)	559.7 (27.17)	NA	28 days after vaccination		
			NA	BNT162b2	>70 days	1621 (21.58)	315 (14.43)	NA	28 days after vaccination		
			NA	CVnCoV	>70 days	373 (5.06)	64.5 (3.76)	NA	28 days after vaccination		
			NA	NVX-CoV2373	>70 days	727 (8.86)	124 (6.25)	NA	28 days after vaccination		
			NA	VLA2001	>70 days	202 (2.68)	35.2 (1.65)	NA	28 days after vaccination		
			NA	Ad26.COV2.S	>70 days	563 (6.85)	125 (5.33)	NA	28 days after vaccination		
		2-BNT162b2	NA	Control	>84 days	Ref	Ref	NA	28 days after vaccination		
			NA	BNT162b2	>84 days	1789 (8.35)	392 (6.60)	NA	28 days after vaccination		
			NA	mRNA1273	>84 days	2019 (12.04)	508.7 (12.58)	NA	28 days after vaccination		
			NA	ChAdOx1 nCoV-19	>84 days	950 (6.01)	260 (6.84)	NA	28 days after vaccination		
			NA	CVnCoV	>84 days	487 (2.57)	119.1 (2.59)	NA	28 days after vaccination		
			NA	NVX-CoV2373	>84 days	766 (5.39)	165 (4.94)	NA	28 days after vaccination		

			NA	VLA2001	>84 days	289 (1.38)	67.1 (1.19)	NA	28 days after vaccination		
			NA	Ad26.COV2.S	>84 days	1441 (7.84)	418 (8.02)	NA	28 days after vaccination		
2021 Dec 8	34890537	2-CoronaVac	14 days	NA	NA	3.9 (Ref)	NA	NA	6 months after vaccination	microcyte pathogenic effect assay	×
			14 days	CoronaVac	2 months	45.8 (11.74)	NA	NA	28 days after vaccination		
			14 days	CoronaVac	8 months	137.9 (35.35)	NA	NA	14 days after vaccination		
			28 days	NA	NA	6.8 (Ref)	NA	NA	6 months after vaccination		
			28 days	CoronaVac	2 months	49.7 (7.31)	NA	NA	28 days after vaccination		
			28 days	CoronaVac	8 months	143.1 (21.04)	NA	NA	28 days after vaccination		
**None-Randomised Controlled Trials of a Booster Dose Vaccination in Participants primed with 2 doses of Inactivated COVID-19 Vaccines**											
2021 Nov 23	34815511	2-CoronaVac/BBIBP-CorV, 71	NA	ZF2001	4-8 months	24.89 (Ref)/1881.01 (75.57)	22.56 (Ref)/1944.15 (86.18)	NA	day 0 and day 14 after vaccination	pseudovirus neutralisation test	√
2021 Dec 3	34893344	2-CoronaVac, 36	3-4 weeks	NA	NA	66.6 (Ref)	48.93 (Ref)	NA	21-49 days after vaccination	surrogate virus neutralisation test	×
		2-CoronaVac, 36	3-4 weeks	ChAdOx1 nCoV-19	1-2 months	97.76 (1.47)	97.22 (1.99)	NA	14-35 days after vaccination	surrogate virus neutralisation test	

Note: NA, not available; NAb, neutralizing antibody.

2.3 Rationale and Significance of this Study

The inactivated COVID-19 vaccine, CoronaVac (Sinovac Life Sciences, Beijing, China), is the most widely administrated vaccine type in China and many other countries. A third dose (booster) has been approved in China to combat waning immunity and evolved virus variants. All reported prime-boost vaccinations elicited humoral and cellular immune responses, but with substantial differences in strength. Accumulating evidence, particularly a few real-world test-negative studies in Brazil, Chile and Singapore as well as high numbers of breakthrough cases at regional outbreaks in China, suggest that homologous booster dose with inactivated COVID-19 vaccine provides limited protection against the omicron variant. Thus, boosting strategy with higher immunogenicity is demanded. However, there is still no study systematically evaluating vaccines of four major platforms (mRNA, adenovirus-vectored, recombinant protein, inactivated virus) in the large inactivated-vaccine-primed population.

Given the importance of flexible deployment of different COVID-19 vaccines as a third dose with more choices to further enhance the immunity against the currently circulating variants, this study aims to investigate the immunogenicity and safety of three new COVID-19 vaccine candidates (ChAdTS-S [AdC68-19S, replication-deficient chimpanzee adenovirus-vectored vaccine], RQ3013 [mRNA vaccine] and ZR202-CoV [recombinant protein vaccine]) and CoronaVac [inactivated virus vaccine]) in people who have received two doses of CoronaVac. We believe our study will address important clinical questions involving a significant proportion of vaccinated people worldwide. Through the successful completion of the present study, we expect to add more immunogenic vaccines to the booster vaccination campaign toolbox and inform the public health community more precise vaccination strategies according to properties of each vaccine platform.

3. Trial Objectives

This study aims to evaluate the short-term and long-term immunogenicity and safety of a booster immunisation using four vaccines (ChAdTS-S, RQ3013, ZR202-CoV, and CoronaVac) or placebo on a background of two-dose inactivated SARS-CoV-2 vaccination (CoronaVac) in healthy adults aged 18-59 years.

3.1 Primary Immunogenicity Objectives (Blinded)

To determine the GMTs of neutralizing antibody against live SARS-CoV-2 wild-type, delta and omicron variants at day 0 (before vaccination), and day 7, day 14 and day 28 after a third dose

vaccination in participants vaccinated with two-dose CoronaVac.

3.2 Secondary Immunogenicity Objectives

- GMTs of serum neutralizing antibody against live SARS-CoV-2 wild-type, delta and omicron variants at month 3 after the booster dose (open label).
- GMTs of serum neutralizing antibody against live SARS-CoV-2 wild-type, delta and omicron variants at month 6 after the booster dose (open label).
- GMTs of serum neutralizing antibody measured by competitive inhibition method at day 0 (before the booster vaccination), and day 1, day 4, day 7, day 14, day 28, day 90 and day 180 after the booster dose (blinded before day 28).
- GMTs of serum SARS-CoV-2 receptor binding domain (RBD)-specific IgG, IgM and IgA at day 0 (before the booster vaccination), and day 1, day 4, day 7, day 14, day 28, day 90 and day 180 after the booster dose.
- The proportions of SARS-CoV-2 S-protein-specific interferon (IFN)-γ, interleukin (IL)-4- and granzyme B-secreting T cells at day 0 (before the booster vaccination), and day 7, day 14 and day 28 after the booster dose (FluoroSpot [Mabtech]) (open label).
- The proportions of SARS-CoV-2 S-protein-specific CD4+ and CD8+ T cells secreting IFN-γ, tumor necrosis factor (TNF)-α, IL-4, IL-13, IL-2 at day 0 (before the booster vaccination), and day 7, day 14 and day 28 after the booster dose (flowcytometry method) (open label).

3.3 Primary Safety Objectives (Blinded)

- The incidence of adverse events in each group within 14 days after the booster vaccination.
- The incidence of Grade 3 or higher adverse event within 14 days after the booster vaccination.

3.4 Secondary Safety Objectives

- Adverse events, especially the adverse event rates, within 7 days after the booster vaccination (blinded).
- Adverse events, especially the adverse event rates, within 28 days after the booster vaccination (blinded).
- Adverse events, especially the adverse event rates, within 3 months after the booster

vaccination (open label).

- Adverse events, especially the adverse event rates, within 6 months after the booster vaccination (open label).
- Pregnancy events within 6 months after the booster vaccination (open label).

4. Trial Design

This study is a double-blinded, randomised, placebo-controlled clinical trial. We will screen about 250 healthy subjects aged 18-59 years, who have been vaccinated with two doses of inactivated COVID-19 vaccine (CoronaVac). They will be recruited and randomised at a 1:1:1: 1:1 ratio to receive a booster dose of ChAdTS-S, RQ3013, ZR202-CoV, CoronaVac or placebo at an interval of 100-270 days from the second dose. After randomisation, participants will undertake a third dose intervention and then be followed up regularly for 6 months. This study comprises 3 study phases.

4.1 Pre-randomisation Period (3 weeks)

We will recruit the target population by telephone survey and introduce the research protocol to them. For those who intend to participate in clinical trial, we will inform them the time, place, benefits and risks.

During the screening period, the participant's eligibility for randomisation into the trial will be evaluated. This includes checking the detailed information of the candidate participants to make sure that all inclusion criteria are fulfilled and all exclusion criteria are unfulfilled.

4.2 Randomisation and Vaccination Period (1 day, day 0)

At randomisation, participants who fulfill all eligibility criteria will be asked to sign the consent form and be randomly assigned to either vaccine group of one platform or placebo in a double-blinded fashion by unblined statisticians. Considering the number of participants vaccinated each time, we use a block randomisation strategy with a block size of 10 where we randomly assign 10 participants to each group of adenovirus-vectored, mRNA, recombinant protein and inactivated vaccines or placebo. Participants are ranked by the interval between the second and third doses before being assigned to each block so that people in each block have similar interval between the second and third doses. This strategy is to balance the influence of booster dose interval on vaccine evaluations.

Prior to the vaccination, a brief physical examination will be performed by physicians. The physical examination includes vital signs (temperature, blood pressure, heart rate). Participants found to meet exclusion criteria onsite (e.g., blood pressure too high) will be excluded from the study. Blood samples will be collected for eligible participants before vaccination for baseline immune response assessment. Then, they will be administered for a third dose vaccine by appropriately trained clinical nurses at the Department of Preventive and Health Care of the Affiliated Hospital of Yunnan University. Both the observer and the administered participants are blinded for the types of vaccination. They are only informed of the random assignment numbers until unblinding after data of primary objectives is obtained and locked.

Baseline urinary, hematological and biochemical blood tests will be taken before vaccination at day 0. Participants are observed for at least 45 minutes for anaphylaxis or other acute adverse reactions after vaccination. During this time period, they are given a thermometer, tape measure, and diary card (electronic) to record solicited and unsolicited adverse events within 14 days daily. Participants are asked to record for one time for any occurrence of adverse events during 15-28 days. The study physicians review the diary card regularly to record adverse events and contact the participants immediately once serious adverse events are reported. During 29-180 days adverse events or pregnant events are asked to be reported spontaneously by participants. The grading of adverse event severity and the standard for determining the causality between adverse events and SARS-CoV-2 vaccination are described in Section 7.6.4.

4.3 Follow-up Period (180 days, day 1 ~ day 180)

Participants will continue to be followed at regular intervals (day 1, 4, 7, 14, 28, 90, 180 after vaccination, see section '7.1 By Visit' below) for a planned 6-month period.

At day 1 after the third dose vaccination, the urinary, hematological and biochemical blood tests are performed for safety assessment. In addition, blood samples are taken at day 1, 4, 7, 14, 28, 90, and 180 to explore the kinetics of the humoral and/or cellular responses. At the end of the study, those participants in placebo group will be asked for the intention of vaccinating. If yes, unscheduled visits will be arranged (see Section 7.8). An overview of the study profile is shown in **Figure 2**.

Figure 2. Study Profile.

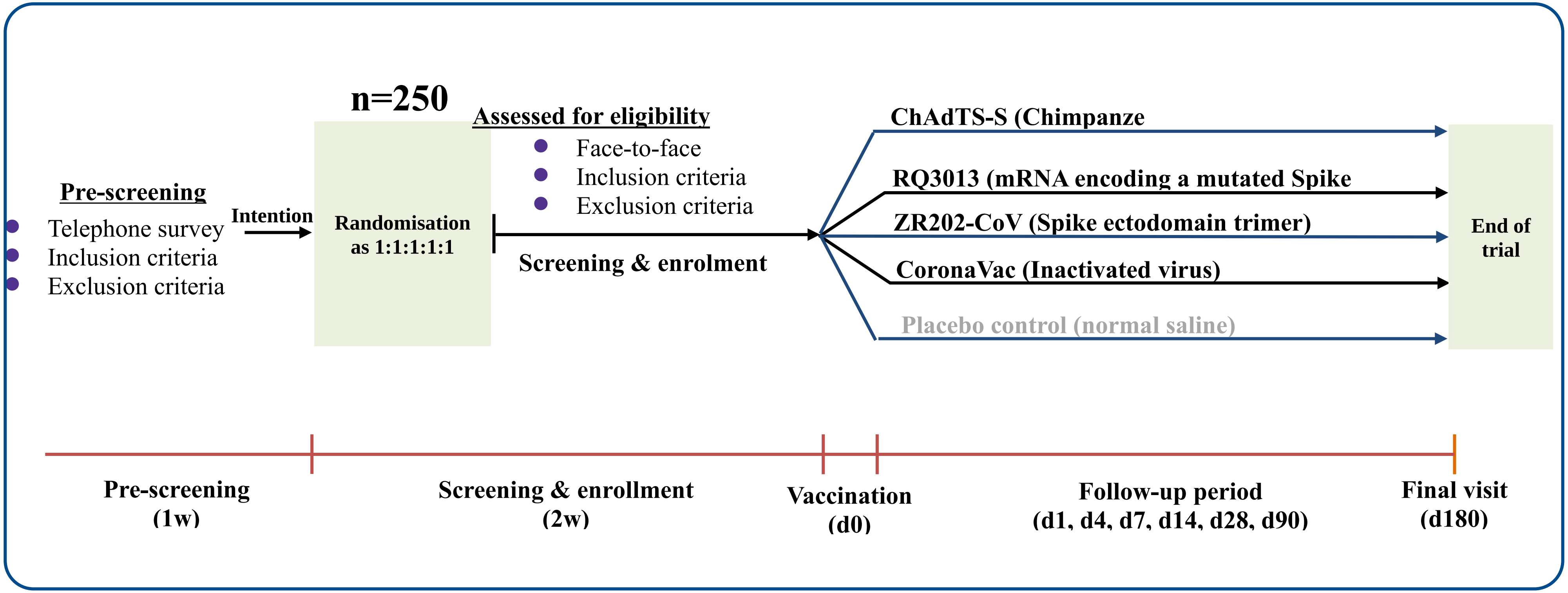

Note: d, day.

5. Trial Intervention

5.1 Investigational Vaccines

The study will evaluate the immunogenicity and safety of a booster dose of four investigational COVID-19 vaccines developed by different platforms. Three newly developed vaccine candidates, with the addition of CoronaVac and saline placebo, are five study intervention options that may be administered to a study participant. The three COVID-19 vaccine candidates developed by different platforms used in this trial include ChAdTS-S (Chimpanzee adenovirus-vectored),[13] RQ3013 (mRNA encoding a mutated Spike)[14] and ZR202-CoV (Spike ectodomain trimer)[15]. Unlike ZF2001 using the SARS-CoV-2 RBD as the immunogen, we use vaccines which express SARS-CoV-2 S protein as immunogen. The rationale for choosing the S protein as immunogen rather than RBD in each vaccine platform was based on previous study that antibodies specific to other domains of the SARS-CoV-2 S protein (e.g. N-terminal domain, NTD) could contribute to the neutralisation to SARS-CoV-2 virus and thus broaden the spectrum of induced neutralizing antibodies.[16] Details of the four investigational COVID-19 vaccines are listed in **Table 3**. All of them are administered via intramuscular injection into the upper arm.

Table 3. Vaccines under Investigation.

Intervention Name	ChAdTS-S	RQ3013	ZR202-CoV	CoronaVac	Placebo
**Mechanism of Action**	Replication-deficient chimpanzee adenovirus-vectored vaccine, expressing spike protein of wild-type SARS-CoV-2	mRNA vaccine encoding SARS-CoV-2 S protein of the Alpha and Beta variants	Nanoparticle vaccine containing purified Spike ectodomain trimer of wild-type SARS-CoV-2	Whole, inactivated wild-type SARS-CoV-2 virus	-
**Dose Formulation**	Adenovirus-vectored	mRNA	Protein	Inactivated SARS-CoV-2	0.9% sodium chloride solution for injection
**Unit Dose Strength(s)**	5×10^{10} viral particles/0.5mL	30 µg mRNA/0.15mL	25 µg Spike ectodomain trimer, 500 µg aluminum hydroxide-based adjuvant and 500 µg CpG ODN/0.5mL	600 SARS-CoV-2 antigen units/0.5 mL	0.5mL
**Dosage Level(s)**	0.5mL/dose	0.15mL/dose	0.5mL/dose	0.5mL/dose	0.5mL/dose
**Manufacturer(s)**	Walvax Biotechnology Co., Ltd	RNAcure-Walvax Biotechnology Co., Ltd	Zerun-Walvax Biotechnology Co., Ltd	Sinovac Biotechnology Co., Ltd	Walvax Biotechnology Co., Ltd

**Route of Administration**	Inject intramuscularly into the deltoid muscle	Inject intramuscularly into the deltoid muscle	Inject intramuscularly into the deltoid muscle	Inject intramuscularly into the deltoid muscle	Inject intramuscularly into the deltoid muscle
**Use**	Experimental	Experimental	Experimental	Experimental	Placebo
**Sourcing**	Provided centrally by the sponsor	Provided centrally by the sponsor	Provided centrally by the sponsor	Provided centrally by the sponsor	Provided centrally by the sponsor

5.2 Preparation/Handling/Storage/Accountability

The study vaccines will be packaged and supplied by the manufacturer. There will be extra regimens to be used in case of loss during intervention and supplementary vaccination for participants assigned to placebo control.

As the package for ChAdTS-S, RQ3013, ZR202-CoV, CoronaVac and placebo (0.9% sodium chloride solution) are different, designated unblinded statisticians will be responsible for vaccine preparation. The original labels on the syringes for injection will be concealed with a label before use. Names (with additional identifier if duplicated) of designated recipient will be used as unique vaccine label by the unblinded statistician for the easiness of handle by the vaccine nurse. The unblinded statisticians will not participant in any other process of the study and are forbidden to reveal the identity of the study vaccine to any other investigators until unblinding.

All clinicians involved in the prescription of study treatment must read the Summary of Vaccine Information which provides detailed information about the composition, indications, side effects, suggested dosage and contraindications of the study interventions.

The investigational vaccines must be kept in a locked area at the vaccine storage room of the department of preventive and health care of the Affiliated Hospital of Yunnan University with restricted access and must be stored and handled in accordance with the manufacturer's instructions. The investigator or pharmacist will also keep accurate records of the quantities of the investigational vaccines dispensed, used, and returned by each participant.

Final enrolment and vaccination will be divided into four batches. The study monitor will check the supplies of investigational vaccines held by the investigator or pharmacist to verify

accountability of all investigational vaccines used after each batch of vaccination (intervention).

For reasons of safety, institutional regulations and storage capacity at trial site, at the conclusion of the study, all used and unused investigational vaccines at the site will be destroyed or return to the manufacture by investigational site staff according to local guidelines following monitoring inspection unless prior arrangements have been approved by the Investigators.

5.3 Administration

Participants will receive the third dose vaccination as randomised at each batch of intervention. Study intervention should be administered intramuscularly into the deltoid muscle, preferably of the non-dominant arm. Administration of study interventions should be performed by well-trained professional nurses at the department of preventive and health care of the Affiliated Hospital of Yunnan University. Appropriate medication and other supportive measures for management of an acute hypersensitivity reaction should be available in accordance with local guidelines for standard immunisation practices.

6. Selection and Withdrawal of Participants

6.1 Target Population

Target population consists of participants aged 18-59 years who have been vaccinated with two-dose inactivated COVID-19 vaccine (CoronaVac).

6.2 Inclusion Criteria

Participants who do not meet any of the following inclusion criteria will not be included in the trial:

- The participants are 18 to 59 years old, male or female;
- Two doses of inactivated COVID-19 vaccine CoronaVac were administered and the interval between two doses was 3-5 weeks;
- It is now in day 100 to day 270 after administering of the second dose of the inactivated COVID-19 vaccine CoronaVac;
- Healthy participants who are determined by medical history, physical examination, and clinical judgment of the investigator to be eligible for inclusion in the study;

- Participants can follow the researchers' guidance to accomplish the research process;
- Participants who are capable of giving personal signed informed consent.

6.3 Exclusion Criteria

Participants who meet any of the following exclusion criteria will not be included in the trial:

- The history of SARS-CoV-2 infection;
- History of severe adverse reaction and/or allergic reaction associated with a vaccine or any vaccine components;
- Pregnancy or planned pregnancy;
- Breastfeeding;
- Medical history of uncontrolled common diseases, such as coronary heart disease, hypertension, diabetes, chronic respiratory disease, tumors;
- Severe psychiatric disorders;
- Receive treatment with immunosuppressants;
- Receipt of blood/plasma products or immunoglobulin from 60 days before enrolment or planned receipt throughout the study;
- Previous or current participation in other studies which could disturb the immunogenicity and safety assessment in this study;
- Participants who have any inapplicable factors according to the study protocol in the view of researcher.

Note: Healthy participants with preexisting stable disease, defined as disease not requiring significant change in therapy or hospitalisation for worsening disease during the 6 weeks before enrolment, can be included.

6.4 Screening and Selection of Participants

All eligible participants who provide informed consent will be invited for randomisation.

There will be 2 study visits during the screening period.

Visit 1: The participant will be provided with information regarding the trial and offered an opportunity to consider and discuss this information with investigators.

Visit 2: Those individuals who provide written informed consent will have eligibility for enrolment into the trial assessment. The screening procedures to be performed are described in Section 4.1.

6.5 Screening Log

The screening log is designed to monitor participant recruitment at the study centre. A screening log of all participants evaluated for enrolment in the study will be recorded in a case record form (CRF). The log will record all screened items, whether they are randomised into the study or considered ineligible for the study. Additionally, the reason participants are excluded or the reasons eligible participants are not enrolled will be recorded in the log. All screening processed will be confirmed by written signature of the physician performing on-site physical exam. A copy of the log should be retained in the investigator's study files.

6.6 Randomisation & Blinding

Participants meeting all inclusion criteria, not meeting any exclusion criteria, and providing informed consent will be randomised to either the vaccine group or placebo group in a 1:1:1:1:1 ratio. The blinding code is generated separately by the randomisation code administrator at Kunming Medical School by the method of block randomisation (block length of 10) using SAS software (version 9.4). An unblinded statistician creates the computer-generated randomisation list. Concealed random group allocations and blinding codes are kept in signed and sealed envelopes.

Participants, laboratory staff, and the clinical study team, including those undertaking adverse event assessment, not delivering the vaccines are blind to treatment allocation. Participant blinding to vaccines is maintained by concealing randomisation pages, preparing vaccines out of sight, and applying masking tape to vaccine syringes to conceal dose, volume, and appearance. The analyzing statisticians remains blind until the statistical analysis plan is signed off.

Randomisation data are kept confidential until the time of unblinding, and will not be accessible by anyone else involved in the study with the exception of the members of the DSMB and the independent bio-statistician who will perform the preliminary analysis. Unblinding of participants should only be performed when knowledge of the treatment allocation will influence the participant's management in a significant fashion. For example, as per regulatory reporting requirement, the principle investigator will unblind the identity of the study medication for all unexpected serious adverse events that are considered by the investigator to be related to study vaccine. The precise reason for unblinding must always be provided, together with details of the name of the clinician making the decision, the date and time the decision is made and any supporting documentation that supports the decision (such as laboratory reports). In any case of unblinding, the follow-up schedule of data collection should be maintained to enable full analysis of all participant data on an intention-to-treat basis.

6.7 Breaking the Blinding

The investigator must not disrupt the blind study of the vaccine unless the treatment allocation information is medically necessary for the subjects in emergency. In the case of a medical emergency, the principal investigator should determine need for an urgent unblinding.

Blinding will be uncovered after cleaning and locking the safety and immunogenicity data at day 28 after the boost dose, but the subjects and safety observers will remain blinded. Before unblinding, ensure that the blind base is well preserved (statisticians keep the blind base), and the unblinding document shall be signed by the principal investigator, the sponsor, and the statistician.

In case of an emergency, the investigator has the sole responsibility for determining if unblinding of a participant's study intervention assignment is warranted. An emergency unlinding envelope containing the randomised grouping for each participant will be prepared together with the blinding code and kept by the statistician. Participant safety must always be the first consideration in making the determination of emergency unblinding. If the investigator decides that unblinding is warranted, the investigator should make every effort to contact the sponsor prior to unblinding a participant's vaccine assignment unless this could delay further management of the participant. If a participant's vaccine assignment is unblinded, the sponsor must be notified within 24 hours after breaking the blind. The date and reason that the blind was broken must be recorded in the source documentation and CRF.

6.8 Withdrawal of Participants

Participants have the right to withdraw from the study at any time for any reason. The investigator also has the right to withdraw participants from the study if they believe that is in the best interests of the participant due to intercurrent illness, severe adverse event, treatment failure, protocol violations, non-compliance, administrative reasons or other reasons.

If the reason for removal of a participant from the study is an adverse event or an abnormal laboratory test result, the principal specific event or test will be recorded on the CRF.

Should a participant decide to withdraw consent or if they are withdrawn by the investigator for reasons mentioned above, all efforts will be made to complete and report the observations prior to withdrawal as thoroughly as possible. A complete final evaluation at the time of the participant’s withdrawal should be made with an explanation of why the participant is withdrawing from the study.

An excessive rate of withdrawals may make study interpretation difficult; therefore, unnecessary withdrawal of participants should be avoided.

6.9 Expected Duration of Trial

The total duration of this study is expected to be at least 7 months with recruitment of at least 1 month and a subsequent follow up of at least 6 months. All randomised participants will participate in the active study phase of up to 6-month duration.

The actual overall study duration or participant recruitment period may vary.

7. Study Procedures and Assessments

7.1 By Visit

Table 4 lists all of the assessments and indicates with an “X” the visits (data and sample collection) when they are performed.

All the scheduled visits are conducted face-to-face, with the exception of visit 1 which is

primarily a telephone visit, but may be conducted face-to-face. A CRF should be completed for every scheduled assessment. All data obtained from the assessments listed in **Table 4** must be supported in the participant's source documentation (e.g., medical charts, participant notes or electronic data).

Whenever possible, study assessments will be made by the same person, at the same time of day, at each study visit. For face-to-face visits, each evaluation will be conducted in the morning wherever possible. Please note that if circumstances exist where the study participant is unable to attend morning site visits (i.e., evening shift worker, etc.), afternoon evaluations are permitted. If possible, participants should present for laboratory evaluations in a fasted state. Visit dates should be adhered to as closely as possible.

If one visit is postponed, brought forward or missed, it should not result in the next visit being postponed or brought forward. The next visit, if at all possible, should adhere to the original time schedule. On occasion, a visit will be missed and this will be captured in the CRF.

Table 4. Schedule of Study Tests, Procedures and Clinic Visits.

Background vaccination (two-dose of CoronaVac)										
**Phase**	**Pre-Screening**	**Screening & enrolment**	**Follow-up**							
**Time (days)**	-7 ☎	0	1	4	7	14	28	90	180	End of Study
**Visit**	1	2	3	4	5	6	7	8	9	10
**Informed consent form**		x								
**Healthy status**		x	x	x	x	x	x	x	x	x
**Inclusion/exclusion criteria**	x	x								
**Medical history/ demography**	x	x								
**Height**		x								
**Weight**		x								
**Vital signs**		x								
**Physical examination**		x								
**Randomisation**		x								
**Urinary routine test**		x	x							
**Haematology**		x	x	x						
**Blood chemistry (fasting)[b]**		x	x							
**Blood Sampling**		x	x	x	x	x	x	x	x	
**Adverse events**		x	x	x	x	x	x	x	x	
**Supplementary vaccination (if applicable)**										x

7.2 Physical Examination & Screening

The subjects' body temperature and blood pressure will be measured before enrolment. According to the "inclusion and exclusion criteria", the interviewers conduct medical history inquiry and screening. Only those who pass the pre-screening could be enrolled and participate in the randomisation. A complete physical examination will be performed at Visit 2 (**Table 4**). It will include the examination of general appearance, skin, lungs and heart. Additional physical examinations may be performed whenever clinically indicated. Vital signs – temperature, blood pressure and heart rate will also be measured at Visit 2 (Screening). Height in centimeters (cm) and body weight (to the nearest 0.1 kilogram [kg] in indoor clothing, but without shoes) will be measured. Only those who passed the final on-site physical examination will be giving intervention.

Information about the all-physical examinations must be present in the CRF, which will act as source data for the purpose of this study. Significant findings that are present prior to the start of the third dose vaccination must be included in the participant's CRF. Significant findings made after the third dose vaccination, which meet the definition of a suspected, unexpected serious adverse event must be recorded on the Serious Adverse Event screen of the participant's CRF.

7.3 Vaccine Distribution and Inoculation

An independent statistical party will be responsible for vaccine preparation and assign the allocated treatment to the subjects according to the random number generated. After the preparation of the vaccine, they hand the ready-to-use syringes to the vaccination nurse, who will administrate the vaccination. The inoculation site is the deltoid muscle of the lateral upper arm and the inoculation route is intramuscular injection.

7.4 Sampling, Processing and Preservation

7.4.1 Blood Sampling

At each visit of V2-V9, 20 mL of venous blood will be collected using two 10-mL purple anticoagulation blood collection tubes (actual blood collection 7-8 mL/tube) and one 5-mL

yellow blood collection tube (actual blood collection was about 2-3 mL/tube). Each sample will be labeled with a code so that the laboratory personnel testing the samples will not know the participant's identity. Blood samples from this clinical trial will be used to test the humoral and cellular responses specified in the protocol, and the use of other studies will require the approval of the IEC.

Table 5. Maximum Blood Sampling Volumes per Participant by Visits.

Study Visit Day	Day 0	Day 1	Day 4	Day 7	Day 14	Day 28	Day 90	Day 180	Total
**Safety laboratory tests**	6 mL	6 mL	-	-	-	-	-	-	12 mL
**Humoral antibodies**	3 mL	3 mL	3 mL	3 mL	3 mL	3 mL	3 mL	3 mL	24 mL
**Cellular responses**	16 mL	16 mL	16 mL	16 mL	16 mL	16 mL	16 mL	16 mL	128 mL
**Total**	25 mL	25 mL	19 mL	19 mL	19 mL	19 mL	19 mL	19 mL	164 mL

7.4.2 Sample Processing and Preservation

Serum

Serum samples will be obtained by centrifugation of the 5 mL venous blood collected by Vacutainer Rapid Serum Tube (BD, USA) and stored at -80°C for humoral antibodies determination.

PBMCs

We preserve whole blood using Vacutainer tubes (BD, USA) containing EDTA at room temperature for no longer than 5 hours before isolation. Peripheral blood mononuclear cells (PBMCs) are then separated by density-gradient sedimentation. In brief, whole blood is diluted by Roswell Park Memorial Institute (RPMI) 1640 (VIVACELL, China) in a 1: 1 ratio, layered over Ficoll (STEMCELL, Canada), and then followed by a centrifugation of 30 minutes at 1455 g with no brake. The PBMC buffy coat and plasma are then collected and washed by adding equal volume RPMI 1640 (VIVACELL, China). Then the liquid supernatant is removed after centrifugation for 10 minutes at 524 g. Cells are aliquot by pre-cooled cryopreservation solution containing 10% Dimethylsulfoxide (DMSO) (Solarbio, China) and 90% fetal bovine serum (VIVACELL, China) and transferred into cryopreservation tubes, placed in a pre-cooled Mr. Frosty freezing container (Thermo Scientific). All containers are then moved into -80°C freezer

overnight before transferring to liquid nitrogen until laboratory immunology assays.

7.5 Immunogenicity Assessment

Serum and cellular samples will be obtained for immunogenicity testing at the visits specified in the **Table 4**. The following assays will be performed.

7.5.1 Live Virus Neutralizing Antibody against Wild-type SARS-CoV-2, the delta and omicron Variants

We will use the gold standard of antibody titration cytopathic effect (CPE)-based microneutralisation assay with authenitic SARS-CoV-2 virus, including wild-type strain (Wuhan-1, GenBank: MT123291), delta variant (B.1.617.2, IQTC-IM2175251) and omicron variant (BA.1.1, IQTC-Y216017), to quantify the neutralizing antibody titres after the booster vaccination (Guangzhou Customs Technology centre, Guangzhou, China). Briefly, serum is first inactivated at 56℃ for 30 minutes and then is 2-fold serially diluted, from 1:4 to 1: 512, in serum free dulbecco's modified eagle medium (DMEM) and seropositivity is defined as titre ≥ 1:8. The diluted serum is then incubated with equal volume of SARS-CoV-2 wild-type virus or variants containing 100 median tissue culture infective dose (TCID50) of virus/well in 96-well plate for 2 hours at 37℃ and 5% CO2. Hereafter, 12,000 Vero E6 cells are added to the serum-virus mix and incubate at 37℃ for 4 days. Images of whole wells are captured using a Celigo Imaging Cytometer (Nexcelom Bioscience, Lawrence, MA, USA). The neutralisation is determined by the appearance of CPE. The neutralizing antibody titre is calculated as the reciprocal of the highest serum dilution showing more than 50% of inhibition. A positive control (plasma sample with known SARS-CoV-2 neutralizing activity) and negative control (human serum sample negative for SARS-CoV-2 extracted before COVID-19 pandemic) are performed as well in each assay to control the quality of the microcytopathogenic effect assay. For all assays, values above the upper limit will be tested series-diluted samples until the measured values fall within the detection range.

7.5.2 anti-SARS-CoV-2 RBD-specific IgG, IgM and IgA, and Neutralizing Antibody using Competitive Inhibition Method

We will use magnetic particle chemiluminescence immunoassay (MCLIA) (Bioscience Co.,

China) to measure anti-SARS-CoV-2 RBD-specific IgG, IgM as well as IgA, and use competitive inhibition-based method to measure the neutralizing antibody tires.[17,18] Antibody tires are presented as the measured chemiluminescence values divided by the cutoff (S/CO). The cutoff value of this test is defined by the receiver operating characteristic curves. We test the antibody tire serum sample except for samples that exhibit neutralizing level higher than 30 S/CO (the upper bound of linearity for antibody detection). For these samples, we will test series-diluted samples until the measured values fall within the detection range. Seroconversion cutoff is defined as 1 S/CO for IgG, IgM and IgA, and 2 S/CO for neutralizing antibody according to the kit manufacturer.

The following formula may be used for converting concentration units from S/CO to binding antibody unit (BAU)/mL for anti-SARS-CoV-2 RBD-specific IgG:
Result (BAU/mL) = Result (S/CO) × 5

The following formula may be used for converting concentration units from S/CO to international unit (IU)/mL for neutralizing antibody measured by competitive inhibition method:
Result (IU/mL) = Result (S/CO) × 25

7.5.3 Cellular Immune Responses

The FluoroSpot assay is a modified version of ELISpot assay that combines the sensitivity of ELISpot with the capacity to study secretion of several analyses simultaneously, enabling studies of cell populations with different functional profiles. FluoroSpot uses fluorescence label instead of Horseradish Peroxidase (HRP) label so that multiple secreted proteins can be detected at the same time. To perform FluoroSpot assay, we will thaw each vial of cryopreserved cells by diluting them in 13.5 mL pre-warmed incubating medium containing 10% fetal calf serum and spinning at 500 g for 10 minutes. Supernatant is carefully removed by toppling and falling. Then we resuspend cells in 500 μL warm incubating medium. To remove cell debris and ensure a stable cellular state for counting, all cells will be rested at 37℃ in a humidified incubator with 5% CO2 for 1 hour. We evaluate the IFN-γ-, granzyme B- and IL-4-secreting cells under the manufacture's instruction of FluoroSpot kit (Mabtech, Sweden), but with the following modifications. Cells are plated in a 96-well plate at 2.5×10^5 cells in 110 μL per well followed by stimulation with whole S protein epitopes designed based on the wild-type SARS-CoV-2 sequence or the omicron variant (B.1.1.529) at a final concentration of 2.0 μg/mL of each peptide, or positive control (anti-CD3

at 0.1 μg/mL), or negative control (DMSO). All the assays are co-stimulated with anti-CD28 (0.1 μg/mL). Then they are incubated at 37°C in a humidified incubator with 5% CO2 for 48 hours.

We will count the number of spots (cytokine-secreting cells) by Mabtech IRIS FluoroSpot/ELISpot Reader. Positive results are defined as follows: the difference of spot-forming units between sample and negative control is more than 10 for IFN-γ and granzyme B, and 5 for IL-4, and difference between negative and positive controls accounts for more than 50% of the negative control. To quantify antigen-specific responses, we subtracted the spot counts of negative control wells from peptide stimulation wells. Finally, T-cell frequencies are reported as spot forming cells (SFC) per 250 000 PBMCs, and these results will be multiplied by four to express frequencies per million PBMCs.

7.6 Safety Assessment

7.6.1 Duration of Observation

After vaccination, the participants will stay at the trial site for at least 45 minutes for acute reaction observation, which will be documented in the adverse event CRF.

At day 1 after vaccination, haematology, blood chemistry and urinary analysis are tested to evaluate the acute organ injury. Clinically significant abnormal laboratory findings are those which are not associated with the underlying disease, unless judged by the investigator to be more severe than expected for the participant's condition. Unscheduled clinical laboratory measurements may be obtained at any time during the study if necessary to assess any perceived safety issues.

Then participants are asked to record the solicited and unsolicited injection site and systemic adverse events (including fever) by themselves daily through an electronic diary (e-diary) till 14 days after the vaccination. From day 15 to day 28 after vaccination, the adverse events are recorded for once. In order to record information on fever, a thermometer will be given to participants with instructions on how to measure axillary temperature at home. Temperature will be collected in the e-diary in the evening daily during the e-diary reporting period and the highest temperature for each day will be recorded in the e-diary. It will also be collected at any time during the e-diary data collection periods when fever is suspected. From day 29 to month 6 after

vaccination, only unsolicited adverse events or pregnant events are asked to be reported by participants spontaneously.

Planned time points for all safety assessments are provided in the Table 4 and the injection site and systemic adverse events are graded as described below in Section 7.6.4.

7.6.2 Laboratory Evaluations

Haematology

Haemoglobin, white blood cell count, lymphocyte and platelet count will be measured.

Blood chemistry

Blood Urea Nitrogen (BUN), creatinine, total bilirubin, alkaline phosphatase, total protein, albumin and uric acid will be measured.

Urinary analysis

Semi quantitative proteinuria and a qualitative microscopic determination - white blood cells per high power field (WBCs/HPF) and red blood cells per high power field (RBCs/HPF) will be performed.

7.6.3 e-diary

Participants will be required to complete an e-diary through an application installed on the participant's own personal device. Investigators will review the e-diary online every day as part of the ongoing safety review.

Adverse events reported intermittently but with less than 3-day interval should be recorded as one adverse event. For any reported solicited adverse events, the investigator must obtain stop dates from the participant for any ongoing local or systemic adverse events on the last day that adverse event is completed.

If a Grade 3 local or systemic event is reported in the e-diary, a telephone contact should occur to ascertain further details and determine whether a site visit is clinically indicated. Only an

investigator or medically qualified person is able to classify a participant's adverse events as Grade 4. If a participant experiences a confirmed Grade 4 local or systemic adverse event, the investigator must immediately notify the sponsor.

7.6.4 Safety Observation Contents and Indicators

Participants are required to record local adverse events (pain, scleroma/swelling and redness), or systemic adverse events (acute allergic reaction, nausea/vomiting, joint pain, muscular pain, headache, chill, fatigue and fever) on e-diary daily for the first 14 days after their third dose vaccination. For days 15-28, they need only to record the solicited adverse events for once. During day 29 to month 6, unsolicited adverse events are collected by spontaneous reporting from participants. Reported adverse events are graded according to China National Medical Products Administration guidelines (China National Medical Products Administration Guidelines for grading standards of adverse events in clinical trials of preventive vaccines. 2019. https://www.nmpa.gov.cn/xxgk/ggtg/qtggtg/20191231111901460.html). The existence of causal associations between adverse events and vaccination is determined by the investigators according to section 7.6.6.

Table 6. Grading of Injection Site (Local) Adverse Events

	**Grade 1**	**Grade 2**	**Grade 3**	**Grade 4**
**Pain**	Having no or marginal effect on limb activity	Having an effect on limb activity	Having an effect on daily life	Loss of basic self-care ability or hospitalisation
**Scleroma/Swelling*#**	Diameter 2.5~<5 cm or area 6.25~<25 cm^2 and having no or marginal effect on daily life	Diameter 5~<10 cm or area 25~<100 cm^2 or having an effect on daily life	Diameter ≥10 cm or area ≥100 cm^2 or fester or secondary infection or phlebitis or sterile abscesses or wound drainage or having serious effect on daily life	Abscess, exfoliative dermatitis, dermal or deep tissue necrosis
**Redness#**	Diameter 2.5～<5 cm or area 6.25～<25 cm^2 and having no or marginal effect on daily life	Diameter 5~<10 cm or area 25~<100 cm^2 or having an effect on daily life	Diameter ≥10 cm or area ≥100 cm^2 or fester or secondary infection or phlebitis or sterile abscesses or wound drainage or having serious effect on daily life	Abscess, exfoliative dermatitis, dermal or deep tissue necrosis

* Scleroma/Swelling: In addition to the grading and evaluation by measuring the diameter directly, the change of measurements should also be recorded.

Scleroma/Swelling and redness: The maximum measured diameter or area should be used; The grading and

evaluation should be based on the function grade and actual measurements and indicators with a higher grade should be chosen.

Table 7. Grading of Systemic Adverse Events

	Grade 1	Grade 2	Grade 3	Grade 4
**Acute allergic reaction***	Local urticaria (blister), no treatment required	Local urticaria, requiring for treatment or mild angioedema, no treatment required	Extensive urticaria or angioedema requiring for treatment or mild bronchospasm	Allergic shock or life-threatening bronchospasm or laryngeal edema
**Nausea/Vomiting**	1~2 times/24 hours and daily activities not affected	3~5 times/24 hours or limited activity or persistent nausea leads to reduced food intake (24-48 hours)	Over 6 times within 24 hours or requiring intravenous infusion or persistent nausea leads to almost no food intake (>48 hours)	Hospitalisation or other nutrition channels indicated due to hypotensive shock
**Joint pain**	Daily activities not affected	Daily activities marginally affected	Severe joint pain, and daily activities severely affected	Urgent intervention or hospitalisation indicated
**Muscular pain (not at the inoculation site)**	Daily activities not affected	Daily activities marginally affected	Severe muscle pain, and daily activities severely affected	Urgent intervention or hospitalisation indicated
**Headache**	Daily activities not affected, and treatment not required	Transient, daily activities marginally affected, and treatment or intervention probably required	Daily activities severely affected, treatment or intervention required	Refractory, urgent intervention or hospitalisation indicated
**Chill**	Transient, daily activities not affected, and treatment not required	Persistent, daily activities marginally affected, and treatment probably required	Daily activities severely affected; treatment required	Urgent intervention or hospitalisation indicated
**Fatigue**	Hypoergia < 48 hours, no impact on activity	Hypoergia for 20%~50%>48 hours, with slight impact on activity	Hypoergia for >50%, with heavy impact on activity and stop working	Incapable of taking care of oneself, and urgent intervention or hospitalisation indicated
**Fever (axillary temperature)**	37.3~<38.0	38.0~<38.5	38.5~<39.5	≥39.5, lasting over 3 days

* Indicates type I hypersensitivity.

Adverse events not included in the above grading tables (unsolicited adverse events) should be graded and evaluated according to the following criteria:

- **Grade 1 Mild**: Short-term (<48 hours) or slight discomfort, no effect on activities, treatment not required;
- **Grade 2 Moderate**: Mild or moderate restricted activities, presentation probably required, treatment not required or mild treatment required;
- **Grade 3 Severe**: Significant restricted activities, presentation and treatment required, hospitalisation probably required;

- **Grade 4 Critical**: Life-threatening possibly, severely restricted activities, intensive care required;
- **Grade 5 Death**.

7.6.5 Outcomes of Adverse Events

The outcomes of adverse events include: Recovery; Not yet recovered; Recovered but sequelae; Death or Loss of visit.

7.6.6 Relationship between Adverse Events and Vaccination

Definitively unrelated: The subject has not used the investigational vaccine; or the adverse event occurs in an implausible time relationship to administration of the investigational vaccine; or there are other significant reasons that may result in the adverse event.

Unlikely related: There is evidence of administration of the investigational vaccine; the adverse event is more likely to be caused by other reasons; a negative or uncertain result is observed after re-administration of the investigational vaccine.

Possibly related: There is evidence of administration of the investigational vaccine; the adverse event occurs in a plausible time relationship to administration of the investigational vaccine; administration of the investigational vaccine cannot be ruled out as a cause of the adverse event, but other reasons may be the cause.

Probably related: There is evidence of administration of the investigational vaccine; the adverse event occurs in a plausible time relationship to administration of the investigational vaccine; the occurrence of the adverse events is explained by the investigational vaccine more reasonably than other reasons.

Definitely related: There is evidence of administration of the investigational vaccine; the adverse event occurs in a plausible time relationship to administration of the investigational vaccine; the occurrence of the adverse events is explained by the investigational vaccine more reasonably than other reasons; a positive result is observed after re-administration of the investigational vaccine; the adverse events are consistent with previous knowledge of this or this type of vaccine.

7.6.7 Documentation and Reporting of Adverse Events

All reportable adverse events that occur during the observation period set in this protocol will be reported by the investigator on the adverse event log of the CRF.

Serious adverse events and adverse events that fulfill a reason for expedited reporting must be documented in the CRF within 24 hours and notification will be sent to the primary investigators (including the medical monitor).

The investigator must also inform the study monitor in all cases. The initial report must be as complete as possible, including details of the current illness and (serious) adverse event, and an assessment of the causal relationship between the event and the study intervention. The investigator will submit reportable adverse events to the relevant ethics committees in accordance with local ethics committee reporting requirements.

7.7 Pregnancy

All female participants of childbearing potential will have a consultation screening whether they are preparing for pregnancy currently or in the coming 6 months at Visit 1 to evaluate eligibility for the trial. Pregnant events will be collected during the 6-month monitoring.

7.8 Unscheduled Visits

The CRF will have the capacity to capture data for unscheduled visits that may occur after the end of this study. After breaking blinding, those who injected with placebo would give a chance to vaccinate with one of the vaccines according to their intention.

7.9 Data Handling & Management

Data for this study will be captured via CRF and e-diary. The investigator should ensure the accuracy, completeness and timeliness of the data.

For each participant

led, a CRF must be completed. The participants will be identified by names and a participant

ID number/identification code on the CRF. The access of the information recorded in the CRF will be restricted to related research personnel and kept confidential.

Data will be validated for accuracy and reliability using a comprehensive validation check program, which will centrally verify the data according to the Data Management Document and automatically generate discrepancies for resolution by the investigator. Manual discrepancies can also be raised if necessary.

An electronic audit trail will maintain a record of initial entries and changes made; reasons for change; time and date of entry; and name of person who made the change.

8. Statistics

8.1 Sample Size Calculation and Reasoning

The sample calculation for this trial is based on the following assumptions:

- The standard deviation of the GMT on log scale (base 10) is 0.4 based on the current available data.
- The minimum clinical difference to detect is 3-fold difference in GMT between the three heterologous boosting vaccines and inactivated vaccine control, i.e., 0.477 on log scale (base 10).
- The minimum clinical difference to detect is 5-fold difference in GMT between the four study vaccines and placebo control, i.e., 0.699 on log scale (base 10).
- Since we will make, at most, four comparisons for each index, using a Bonferroni correction would need to adjust for a significance level of 0.05/4=0.0125. To account for multiple testing, a conservative two-sided significance level of 0.01 will be used.

Based on the above assumptions, the study will need to recruit at least 23 participants in each group to achieve 90% power with a two-sided significance level of 0.01. Taking into account of an attrition rate of 20%, a sample size of 29 participants in each group is required.

However, according to guidelines by regulatory agencies, to ensure the safety assessment 40-50 participants per arm are usually required in the phase I/II clinical trials.

Therefore, a sample size of 50 per group is finally chosen and the required sample size in total is

250.

8.2 Statistical Analyses

We will assess immunogenicity outcomes in the modified intention-to-treat (mITT) population which includes all participants who complete their booster doses and have at least one post-dose immunogenicity data. Summaries of baseline characteristics will be reported for all enrolled participants in the mITT analysis. The primary immunogenicity outcome of neutralizing antibodies against SARS-CoV-2 (wild-type, delta and omicron) will be reported as GMT and 95% confidence intervals (CI). Generalised linear model will be fitted to log-transformed antibody titres to calculate GMRs between groups by adjusting for baseline antibody titres, intervals between the first and second vaccine doses, and intervals between the second and third doses. Safety outcomes will be assessed in the safety population, which includes all participants who receive the booster vaccine. The proportion of participants with at least one adverse event will be reported by the vaccine schedules.

All statistical analyses will be done using R (version 4.2.1). More details are shown in the Statistical Analysis Plan.

9. Participant Confidentiality & Record Keeping

9.1 Participant Confidentiality

Participants will be assigned a unique study numerical identifier. Initially a separate list with participant identifier and study number will be kept in order to contact the participant arrange for blood collection post vaccination and for regular follow-up to ascertain if they developed COVID-19 infection or adverse reaction despite vaccination. The investigator and trial staff must ensure that participants' anonymity will be maintained, that their identities are protected from unauthorised parties, and take measures to prevent accidental or premature destruction of these documents. When the study is complete, this will be destroyed. Samples will only contain study number and date. Samples will be kept until the study is complete.

All records and documents pertaining to the study will be retained by the study trial site at the Affiliated Hospital of Yunnan University for up to 25 years from the completion of the study. Paper records will be stored in a secure filing cabinet in a locked office. Baseline demographic data will be collected and then stored in an electronic database. The database will be stored on the Affiliated Hospital of Yunnan University network accessed from a password protected

computer located in a secure location in the hospital.

9.2 Investigator's Files /Source Documents/ Retention of Documents

The Investigator must maintain adequate and accurate records to enable the conduct of the study to be fully documented and the study data to be subsequently verified. These documents should be classified into two separate categories (1) investigator's Study File and (2) participant clinical source documents.

The Investigator's Study File will contain the protocol/amendments, schedule of assessments, Independent Ethics Committee/Institutional Review Board, sample informed consent, vaccine records, staff curriculum vitae and authorisation forms and other appropriate documents/correspondence, etc. In addition, at the end of the study the investigator will receive the participant data, which includes an audit trail containing a complete record of all changes to data, query resolution correspondence and reasons for changes in readable format on CD, which also has to be kept with the Investigator's Study File.

For this trial, the CRF will serve as source data, but some hard-copy source data must also be maintained as shown in Appendix 2 below. Participant clinical source documents could include hospital/clinic records, physician's and nurse's notes, appointment book, original laboratory reports, special assessment reports, signed informed consent forms, consultant letters, and participant screening and enrolment logs. The investigator must keep these two categories of documents on file for at least 25 years after completion or discontinuation of the study. After that period of time the documents may be destroyed, subject to local regulations.

10. Quality Assurance Procedures

The study will be conducted in accordance with the current approved protocol, ICH Guidelines for Good Clinical Practice (CPMP/ICH/135/95) July 1996 (ICH GCP), Declaration of Helsinki, relevant regulations and standard operating procedures.

10.1 Obtaining Informed Consent

The participant must personally sign and date the latest approved version of the informed consent form before any study specific procedures are performed.

Written and verbal versions of the participant information and informed consent will be presented to the participants detailing no less than: the exact nature of the study; the implications and constraints of the protocol; the known side effects and any risks involved in taking part. It will be clearly stated that the participant is free to withdraw from the study at any time for any reasons without prejudice to future care, and with no obligation to give the reason for withdrawal.

The participant will be allowed as much time as they require to consider the information, and the opportunity to question the investigator, their GP or other independent parties to decide whether they will participate in the study. Written Informed Consent will then be obtained by means of participant dated signature and dated signature of the person who presented and obtained the informed consent. The person who obtained the consent must be suitably qualified and experienced, and have been authorised to do so by the Principal Investigator. A copy of the signed Informed Consent will be given to the participants. The original signed form will be retained at the study site.

If the participant is unable to read, oral presentation and explanation of the written informed consent form and information to be supplied to participants must take place in the presence of an impartial witness. Consent must be confirmed at the time of consent orally and by the personally dated signature of the participant or by a local legally recognised alternative (e.g., the participant’s thumbprint or mark). The witness and the person conducting the informed consent discussions must also sign and personally date the consent document.

The investigator should inform the participant’s primary physician about the participant’s participation in the trial if the participant has a primary physician and if the participant agrees to the primary physician being informed.

10.2 Delegation of Investigator Duties

The investigator should ensure that all persons assisting with the trial are adequately qualified, informed about the protocol, any amendments to the protocol, the study treatments and their trial-related duties and functions. The investigator should maintain a list of sub-investigators and other appropriately qualified persons to whom he or she has delegated significant trial-related duties.

10.3 Ethics and Regulatory Approvals

Before the start of the study, the protocol, informed consent document, any proposed advertising material and any other appropriate documents will be submitted to the appropriate Human Research Ethics Committee (HREC) for written approval. The investigator will submit and, where necessary, obtain approval from the above parties for all subsequent and substantial amendments to the original approved documents.

Safety reports, preliminary reports and a final report at conclusion of the trial will be submitted to the Regulatory Authorities, research ethics committees and if applicable, to the study treatment manufacturer within the timelines defined in the Regulations.

10.4 Management of Protocol Deviations

A protocol deviation is an unanticipated or unintentional departure from the expected conduct of an approved study that is not consistent with the current research protocol or consent document. A protocol deviation may be an omission, addition or change in any procedure described in the protocol.

The investigator should not implement any deviation from or changes of the protocol without agreement by the Steering Committee and documented approval from the Independent Ethics Committee of the amendment, except where necessary to eliminate an immediate hazard(s) to trial participants. In the event of an emergency intended to eliminate an apparent immediate hazard to participants, the Investigator may implement any medical procedure deemed appropriate.

Deviations from the protocol must be documented and promptly reported to the investigator and the Independent Ethics Committee (if applicable). The report should summarised the event and actions taken.

10.5 GCP Training and Site Monitoring

Study monitors from the CRO team will conduct a site initiation visit prior to the start of the

study to ensure that proper study-related documentation exists, assist in training investigators and other site personnel in study procedures and GCP guidelines, confirm receipt of study supplies, and ensure that acceptable facilities are available to conduct the study.

The monitors will verify that the clinical trial procedures are being conducted and data are generated, documented and reported in compliance with the protocol, ICH GCP and the applicable regulatory requirements. Data recorded in the CRF will be evaluated for compliance with the protocol and accuracy in relation to source documents.

On completion of all participant treatments and evaluations, the monitor will conduct a closure visit at the trial site.

10.6 Audits and Inspections

The investigator should permit auditing by or on the behalf of the Sponsor and inspection by regulatory authorities. The investigator agrees to allow the auditors/inspectors to have direct access to his/her study records for review, being understood that these personnel is bound by professional secrecy, and as such will not disclose any personal identity or personal medical information. The investigator will make every effort to help with the performance of the audits and inspections.

As soon as the investigator is notified of a planned inspection by the authorities, he/she will inform the Sponsor and authorise the Sponsor to participate in this inspection. Any result or information arising from the inspections by the regulatory authorities will be immediately communicated by the investigator to the Sponsor. The investigator shall take appropriate measures required by the Sponsor to take corrective actions for all problems found during the audit or inspections.

10.7 Executive Committee and Steering Committee

The study will be conducted under leadership of a Steering Committee that has overall responsibility for protocol design, study conduct and publication. The members of the Steering Committee have accountable experience in managing patients with SARS-CoV-2 vaccination, and have demonstrated experience and expertise in designing, conducting and analysing

clinical studies. Members of this committee includes vaccine clinicians at the department of preventive and health care, clinicians at the intensive care unit (ICU), clinician at the department of infectious disease of Affiliated Hospital of Yunnan University and researchers at the Yunnan University.

10.8 Data and Safety Monitoring Board (DSMB)

An independent DSMB has been established to review the progress of the study and monitor adherence to the protocol, participant recruitment, outcomes, complications and other issues related to participant safety. They will also monitor the assumptions underlying sample size calculations for the study and alert the investigators if they see substantial departures as the data accumulate.

The DSMB consists of vaccine expert, trialists and statisticians who are not involved in managing the trial. The independent DSMB will review safety data on an on-demand basis and may recommend the investigators to stop or amend the study based on safety findings. Since the planned sample size of this study is not very big, there will no interim analysis. The planned DSMB meeting will be held after all the safety data up-to 28 days post vaccination are collected.

10.9 Termination of the Study

The study must be closed at the site on completion of all participant treatment and evaluations. Furthermore, the study may be closed at any time at the request of the Steering Committee, the investigator, or a regulatory authority, with proper and timely notification of all parties concerned. As far as possible, early closure should occur after mutual consultation.

11. Publication Policy

It is intended that the results of the study will be reported and disseminated at conferences and in peer-reviewed scientific journals. Both positive and negative results will be provided.

Appendix 1: Definitions of Adverse Events

Adverse Events

According to the International Conference of Harmonisation [ICH], an adverse event is any untoward medical occurrence in a participant or clinical investigation subject administered a pharmaceutical product, which does not necessarily have a causal relationship with this treatment. An adverse event can therefore be any unfavourable and unintended sign or symptom or abnormal laboratory tests, or disease temporally associated with the use of a medicinal [investigational] product, whether or not considered related to the medicinal [investigational] product. Pre-existing conditions, which worsen during a study, are adverse events.

All reportable adverse events encountered during the clinical study will be reported on the adverse event electronic form (e-diary). Intensity of adverse events will be graded on a four-point scale [grade 1-4] as mentioned in Section 7.6.4.

Serious Adverse Events

Serious adverse events are defined as any untoward medical occurrence that meets one of more of the following criteria:

- Results in death
- Is life-threatening
- Requires inpatient hospitalisation or prolongation of existing hospitalisation
- Results in persistent or significant disability/incapacity
- Is a congenital anomaly/birth defect

The classification of 'serious adverse event' is not related to the assessment of the severity of the adverse event. An event that is mild in severity may be classified as a serious adverse event based on the above criteria. If there is any doubt whether an event constitutes a severe adverse event, this event should be considered a severe adverse event.

Adverse Events of Special Interest

Adverse events of special interest are defined as new COVID-19 infection during the study.

Appendix 2: Specification of Source Data

Assessment	What will function as Source Data
Informed consent form	Individual consent form
In/exclusion criteria	CRF
Screening log	Screening log maintained at trial site
Randomisation	Randomisation form
Study vaccine dispensation	Drug accountability logs maintained at trial site
Study vaccine accountability	Drug accountability logs maintained at trial site
Med History/ Demography	CRF, and copies of documents/letters where available to be filed in participant file
Height and Weight	CRF
Vital signs	CRF
Physical Exam	CRF
Short physical exam	CRF
Urinary analysis	Lab report – filed in the participant file signed and dated by the responsible clinician
Haematology	Lab report – filed in the participant file signed and dated by the responsible clinician
Blood chemistry	Lab report – filed in the participant file signed and dated by the responsible clinician
Adverse events	e-diary
Serious and reportable adverse events	Written information on diagnosis, hospital discharge summaries etc. – filed in the participant file

References

1. Tregoning JS, Flight KE, Higham SL, Wang Z, Pierce BF. Progress of the COVID-19 vaccine effort: viruses, vaccines and variants versus efficacy, effectiveness and escape. Nat Rev Immunol 2021; 21: 626-36.
2. Polack FP, Thomas SJ, Kitchin N, et al. Safety and Efficacy of the BNT162b2 mRNA Covid-19 Vaccine. N Engl J Med 2020; 383: 2603-15.
3. Baden LR, El Sahly HM, Essink B, et al. Efficacy and Safety of the mRNA-1273 SARS-CoV-2 Vaccine. N Engl J Med 2021; 384: 403-16.
4. Tanriover MD, Doganay HL, Akova M, et al. Efficacy and safety of an inactivated whole-virion SARS-CoV-2 vaccine (CoronaVac): interim results of a double-blind, randomised, placebo-controlled, phase 3 trial in Turkey. Lancet 2021; 398: 213-22.
5. Folegatti PM, Ewer KJ, Aley PK, et al. Safety and immunogenicity of the ChAdOx1 nCoV-19 vaccine against SARS-CoV-2: a preliminary report of a phase 1/2, single-blind, randomised controlled trial. Lancet 2020; 396: 467-78.
6. Flaxman A, Marchevsky NG, Jenkin D, et al. Reactogenicity and immunogenicity after a late second dose or a third dose of ChAdOx1 nCoV-19 in the UK: a substudy of two randomised controlled trials (COV001 and COV002). Lancet 2021; 398: 981-90.
7. Falsey AR, Frenck RW, Jr., Walsh EE, et al. SARS-CoV-2 Neutralization with BNT162b2 Vaccine Dose 3. N Engl J Med 2021; 385: 1627-9.
8. Zeng G, Wu Q, Pan H, et al. Immunogenicity and safety of a third dose of CoronaVac, and immune persistence of a two-dose schedule, in healthy adults: interim results from two single-centre, double-blind, randomised, placebo-controlled phase 2 clinical trials. Lancet Infect Dis 2022; 22: 483-95.
9. Munro APS, Janani L, Cornelius V, et al. Safety and immunogenicity of seven COVID-19 vaccines as a third dose (booster) following two doses of ChAdOx1 nCov-19 or BNT162b2 in the UK (COV-BOOST): a blinded, multicentre, randomised, controlled, phase 2 trial. Lancet 2021; 398: 2258-76.
10. Ai J, Zhang H, Zhang Q, et al. Recombinant protein subunit vaccine booster following two-dose inactivated vaccines dramatically enhanced anti-RBD responses and neutralizing titers against SARS-CoV-2 and Variants of Concern. Cell Res 2022; 32: 103-6.
11. Yorsaeng R, Suntronwong N, Phowatthanasathian H, et al. Immunogenicity of a third dose viral-vectored COVID-19 vaccine after receiving two-dose inactivated vaccines in healthy adults. Vaccine 2022; 40: 524-30.
12. Zhang J, He Q, An C, et al. Boosting with heterologous vaccines effectively improves protective immune responses of the inactivated SARS-CoV-2 vaccine. Emerg Microbes Infect

2021; 10: 1598-608.
13. Li M, Guo J, Lu S, et al. Single-Dose Immunization With a Chimpanzee Adenovirus-Based Vaccine Induces Sustained and Protective Immunity Against SARS-CoV-2 Infection. Front Immunol 2021; 12: 697074.
14. Lu J, Lu G, Tan S, et al. A COVID-19 mRNA vaccine encoding SARS-CoV-2 virus-like particles induces a strong antiviral-like immune response in mice. Cell Res 2020; 30: 936-9.
15. Liu H, Zhou C, An J, et al. Development of recombinant COVID-19 vaccine based on CHO-produced, prefusion spike trimer and alum/CpG adjuvants. Vaccine 2021; 39: 7001-11.
16. Kang Wang YC, Zhou Y, Wu J, et al. A third dose of inactivated vaccine augments the potency, breadth, and duration of anamnestic responses against SARS-CoV-2. medRxiv 2021.
17. Long Q, Liu B, Deng H, et al. Antibody responses to SARS-CoV-2 in patients with COVID-19. Nat Med 2020; 26: 845-8.
18. Xu X, Sun J, Nie S, et al. Seroprevalence of immunoglobulin M and G antibodies against SARS-CoV-2 in China. Nat Med 2020; 26: 1193-5.

Statistical Analysis Plan

Clinical Evaluation of Immunogenicity and Safety of Adenovirus-vectored, mRNA, Recombinant Protein and Inactivated Vaccine Booster Dose among Adults Who Have Received Two Doses of Inactivated COVID-19 Vaccine

Protocol Number: YNUVC-2022001

Version Number: 1.0

Sponsor: The Affiliated Hospital of Yunnan University

Research organisations: Yunnan University

The Affiliated Hospital of Yunnan University

Statistical Organisation: Kunming Medical University

Southern Medical University

STATISTICAL CONSIDERATIONS

Part A: Sample Size Calculation and Reasoning

The sample size is calculated based on the following assumptions:

- The standard deviation of the GMT on log scale (base 10) is 0.4 based on the current available data.
- The minimum clinical difference to detect is 3-fold difference in GMT between the three heterologous boosting vaccines and inactivated vaccine control, i.e., 0.477 on log scale (base 10).
- The minimum clinical difference to detect is 5-fold difference in GMT between the four study vaccines and placebo control, i.e., 0.699 on log scale (base 10).
- Since we will make, at most, four comparisons for each index, using a Bonferroni correction would need to adjust for a significance level of 0.05/4=0.0125. To account for multiple testing, a conservative two-sided significance level of 0.01 will be used.

Based on the above assumptions, the study will need to recruit at least 23 participants in each group to achieve 90% power with a two-sided significance level of 0.01. Taking into account of an attrition rate of 20%, a sample size of 29 participants in each group is required.

However, according to guidelines by regulatory agencies, to ensure the safety assessment 40-50 participants per arm are usually required in the phase I/II clinical trials.

Therefore, a sample size of 50 per group is finally chosen and the required sample size in total is 250.

Based on sample size of 50 per group, the precision and 95% confidence intervals (CIs) of safety events are:

Table 1. Probability of Testing Vaccination Safety.

True safety event rate	Precision (normal approximation)	95% exact binomial CI
5%	±5.5%	0.8%-15.2%
10%	±8.3%	3.3%-21.8%
15%	±9.9%	6.5%-27.9%
20%	±11.1%	10.0%-33.7%
25%	±12.0%	13.8%-39.3%
50%	±13.9%	35.5%-64.5%

Part B: Statistical Analyses

1. Selection of Analysis Data Sets

1.1 Immunogenicity Data Set

Full Analysis Set (FAS): It is defined as an ideal participant population determined according to the intention-to-treat (ITT) analysis. All participants who meet the inclusion/exclusion criteria take the booster vaccination and have at least one post-dose immunogenicity data will be included in the FAS (modified ITT). The group of participants will be determined by the intervention they actually receive rather than they are allocated to.

Per-Protocol Set (PPS): It is a subset of FAS. Participants in this set are more compliant with the protocol, experience no major protocol violation, comply with all inclusion criteria/exclusion criteria, and complete the vaccination within the time window as required in the protocol and all blood samplings for primary outcome (day 0 before vaccination, and days 7, 14 and 28 after vaccination) are included in the PPS set. Participants who met one or more criteria below will not be included in this analysis set:

- Participants receive wrong vaccination;
- Participants receive vaccines or medicines that inhibited by this study proposal:

 1) Participants involved in another study;

 2) Participants use long-term (persistent over 14 days) immunosuppressants or other immune regulatory drugs (inhaled or local use corticosteroids is allowed);

 3) Participants receive immunoglobulin and/or other blood products;

- New onset autoimmune diseases;
- Other situations which disturb the assessment of vaccination immunogenicity.

1.2 Safety Data Set

The safety evaluation should be conducted for all participants who receive vaccines after randomisation. Data violating the protocol should not be eliminated. In accordance with ASaT (All Subjects as Treated) principle, the group of participants will be determined by the intervention they actually receive rather than they are allocated to.

1.3 General Principles

The primary outcome analyses will be carried out once the immunogenicity and safety data within 28 days become available.

Censored data are expected for immunogenicity outcomes as these assays normally have a lower limit of detection. Data below the lower limit of detection/quantification will be imputed

by a value half the lower limit of detection, prior to log10 transformation. For data above the high limit of detection/quantification, we will test series-diluted samples until the measured values fall within the detection range. Missing data will not be imputed.

Histograms and boxplots will be used to check the distribution and for possible outliers for continuous variables. Outliers will be checked to confirm the validity of the data. Mathematical transformations (log10) will be applied, where appropriate, in order to render a normal distribution.

Continuous variables that follow an approximately normal distribution will be summarised using means, standard deviations and range values, and number of missing values. Skewed continuous variables will be summarised using medians/geometric mean (where appropriate), inter-quartile ranges (IQRs) and range values, and number of missing values. Categorical/binary variables will be summarised using frequencies and percentages.

Baseline characteristics will be summarised for each arm to describe the study population. This will contain the numbers of participants randomly assigned to each group, receiving vaccination, completing the study and analysed for the primary outcome. It will also include a breakdown of reasons for withdrawal and their relative time points.

For the primary analyses on immunogenicity (comparing each study vaccine arm with inactivated vaccine or placebo as controls), the statistical tests will be two-sided and a p value less than 0.01 will be considered significant. The significance level for all the other secondary analyses will be 2-sided 0.05, unless specified otherwise in the analysis section below.

2. Baseline Demographics

Baseline characteristics will be summarised and compared by study vaccines.

3. The Primary Immunogenicity Outcome Analysis

3.1 Definition of Outcome

A superiority test by comparing the GMTs of neutralizing antibody against the wild-type SARS-CoV-2 and delta and omicron variants between each COVID-19 vaccine group with the inactivated vaccine group or the placebo control.

3.2 Population for Analysis

The modified ITT population include all participants who meet the inclusion/exclusion criteria

take the booster vaccination and have at least one post-dose immunogenicity data. The group of participants will be determined by the intervention they actually receive rather than they are allocated to.

3.3 Statistical Analysis

The primary outcome is the live virus neutralizing antibody GMTs against the wild-type SARS-CoV-2, the delta and omicron variants at day 7, 14, and 28 post vaccination. The GMTs will be compared between each of the vaccine arms and the placebo arm under the hypothesis:

H0: GMT vaccine / GMT placebo =1 or log GMT vaccine -log GMT placebo = 0;

H1: GMT vaccine / GMT placebo ≥ 5 or log GMT vaccine - log GMT placebo ≥ 0.699.

The GMTs will also be compared between each of the heterologous schedules with ChAdTS-S, RQ3013 or ZR202-CoV, and the homologous schedule with CoronaVac under the hypothesis:

H0: GMT heterologous / GMT homologous =1 or log GMT heterologous -log GMT homologous = 0;

H1: GMT heterologous / GMT homologous ≥ 3 or log GMT heterologous - log GMT homologous ≥ 0.477.

The GMTs of each arm will be calculated as the antilogarithm of sum (log10 transformed titre)/n, i.e., as the antilogarithm transformation of the mean of the log10 transformed titre, where n is the number of participants in that arm. The 95% CI will be calculated as the anti-logarithm transformation of the upper and lower limits for a two-sided CI for the mean of the log10 transformed titres. Data reported as lower than the detection threshold will be imputed with a value equal to half of the threshold before the transformation.

The Geometric Mean Ratio (GMR) will be calculated as antilogarithm of the difference between the mean of the log10 transformed titre in the vaccine arm and that in the control arm (as the reference), after adjusting for baseline immunogenicity, interval between the first and second doses, and interval between the second and third doses, in a linear mixed-effect regression model. The two-sided 95% CI of the adjusted GMR will be calculated as the antilogarithm transformation of the upper and lower 95% CI limits of the adjusted difference of the log10 transformed means.

3.4 Subgroup Analyses

Subgroup analyses for the primary outcome will be conducted by adding interaction terms to

the primary model. The adjusted GMR and two-sided 95% CI will be presented for each group. The subgroup analyses will be performed for all participants and include:

- Sex (male and female)
- Vaccination interval between the second and third doses (< 180 days or >= 180 days)
- Comorbidity, if applicable (with or without comorbidities at baseline, such as cardiovascular diseases, respiratory diseases, and diabetes)

3.5 Missing Data

Missing data will not be imputed, and the analysis participate number will be informed.

4. Secondary Immunogenicity Outcome Analysis

4.1 Definition of Outcomes

Secondary outcomes include:

- Live virus neutralizing antibodies against the wild-type, delta and omicron variants of live SARS-CoV-2 at day 90 and day 180 after booster vaccination.
- Neutralizing antibodies measured by competitive inhibition method at day 0 before the booster dose, and at days 1, 4, 7, 14, 28, 90 and 180 after the booster vaccination.
- Anti-SARS-CoV-2 RBD-specific IgG, IgM and IgA at day 0 before the booster dose, and at days 1, 4, 7, 14, 28, 90 and 180 after the booster vaccination.
- Cellular immune responses by FluoroSpot (Mabtech) at day 0 before the booster dose, and at days 7, 14 and 28 after the booster vaccination.
- Cellular immune responses by Flowcytometry at day 0 before the booster dose, and at days 7, 14 and 28 after the booster vaccination.

4.2 Population for Analysis

The modified ITT population include all participants who meet the inclusion/exclusion criteria take the booster vaccination and have at least one post-dose immunogenicity data. The group of participants will be determined by the intervention they actually receive rather than they are allocated to.

4.3 Statistical Analysis

The analyses for all secondary outcomes will follow the same principle and test the same

hypothesis as set out in the primary immunogenicity analysis. We will use transformations and comparing the GMRs where suitable.

Secondary outcome analyses will focus on the description of immunogenicity kinetics between different arms. A line plot of the GMTs with a measure of precision (95% CI) or median with IQR will be plotted across available time points for each arm within a group.

5. Safety Outcome Analysis

5.1 Definition of Outcomes

These outcomes comprise of both solicited adverse events over the first 14 days of local and systemic reactions (reactogenicity outcomes), and spontaneously reported (unsolicited) adverse events, serious adverse events, adverse events of special interest. Adverse events will also be detected through routine clinical and blood screening tests before and 1 day after the booster vaccination.

Solicited adverse events are named in the study protocol Section 7.6.4.

Unsolicited adverse events are all events that have been spontaneously reported (unsolicited) or adverse event detected through routine clinical and blood screening tests.

Serious adverse events is any untoward medical occurrence that:

- Results in death.
- Is life-threatening.
- Requires inpatient hospitalisation or prolongation of existing hospitalisation.
- Results in persistent or significant disability/incapacity.
- Consists of a congenital anomaly or birth defect.

Adverse events of special interest are defined as any adverse event identified as being of particular relevance to the investigational vaccines.

5.2 Population for Analysis

The safety evaluation should be conducted for all participants who receive vaccines after randomisation. Data violating the protocol should not be eliminated. In accordance with ASaT (All Subjects as Treated) principle, the group of participants will be determined by the intervention they actually receive rather than they are allocated to.

5.3 Statistical Analysis

The proportion of any local or systemic adverse events within 7 days, 14 days and 28 days will be presented in boxplot showing vaccine arm, with one plot per time window.

The proportion with at least one severe local or systemic adverse events within 28 days will be presented showing vaccine arm.

The median duration (days until resolution) of local or systemic adverse events within 7 days, 14 days and 28 days will be presented showing vaccine arm, with one table per time window.

An additional view of reactogenicity outcomes will be provided by priming vaccine in line with prior published vaccine trials, i.e., the information will be visualised using a stacked bar chart by severity and day (x-axis) for each solicited event in one row (one row per vaccine arm).

Part C: Initial Analysis

An initial analysis of immunogenicity and safety will be performed after completing all the data collection within 28 days after the booster vaccination.

Appendix 3
Precise-CoVaccine study group members

First and middle names	Surnames
Zijie	Zhang
Jia	Wei
Tai-Cheng	Zhou
Ying	Wu
Dingyun	You
Guo-Dong	Wang
Zhenwang	Fu
Yuemiao	Zhang
Chunmei	Li
Yanli	Chen
Wei	Yang
Zhongfang	Wang
Xupu	Ma
Guanghong	Yan
Hanfang	Bi
Ao	Li
Na	Wan
Jun	Hu
Fan	Yang
Wei	Su
Tianpei	Shi
Mei	Yang
Rong	Wang
Wanting	Qin
Xuanjing	Yu
Hong-Yi	Zheng
Zumi	Zhou
Yong-Tang	Zheng
Muxian	Dai
Fengwei	Liu
Muhua	Feng
Gangxu	Xu
Yajing	Wang
Lihong	Zhang
Liang	Zhang
Xinshuai	Zhao
Naiyin	Mao
Wenbo	Xu
