# Supplementary material for: Immunogenicity, durability, and safety of an mRNA and three platform-based COVID-19 vaccines as a third dose following two doses of CoronaVac in China: A randomised, double-blinded, placebo-controlled, phase 2 trial
Source: eClinicalMedicine. 2022 Sep 28;54:101680. doi: 10.1016/j.eclinm.2022.101680 (PMC9517939; doi:10.1016/j.eclinm.2022.101680)
Supplement: Supplementary file 2 [file mmc2.docx]

**Immunogenicity, durability, and safety of an mRNA and three platform-based COVID-19 vaccines as a third dose following two doses of CoronaVac in China: a randomised, double-blinded, placebo-controlled, phase 2 trial**

Appendix 1: Supplementary Tables and Figures

Appendix 2: Trial Protocol and Statistical Analysis Plan

Appendix 3: Precise-CoVaccine study group members
